# Degradable Alternating Copolymers With Strained Cyclobutane Backbones via Photocontrolled RAFT Polymerization

**DOI:** 10.1002/anie.7228967

**Published:** 2026-08-08

**Authors:** Xia Hu, Hongsik Kim, Lianqian Wu, Yi Jiang, Tae‐Lim Choi

**Affiliations:** ^1^ Department of Materials ETH Zurich Zurich Switzerland

**Keywords:** alternating copolymer, degradable, photocontrolled RAFT polymerization, radical polymerization

## Abstract

In this study, we report a reversible addition‐fragmentation chain transfer (RAFT) alternating copolymerization of 1,3‐disubstituted bicyclo[1.1.0]butanes (BCBs) with maleimides, maleic anhydride, or bio‐based fumarate derivatives, affording a class of well‐defined copolymers with strained cyclobutane backbones. Under visible‐light irradiation, a chain transfer agent (CTA)‐mediated photoiniferter process delivers BCB‐derived copolymers with a high degree of alternation, tunable molecular weights, and excellent end‐group fidelity. Our design leverages electronically and sterically tuned BCB monomers bearing an aryl substituent at one bridgehead and an electron‐withdrawing group at the other, facilitating precise control over radical reactivity and selective cross‐propagation. The resulting polymers exhibit high thermal stability with decomposition temperature up to 429°C, yet undergo highly efficient degradation under mechanical stimulation.

## Graphical abstract

A photocontrolled RAFT strategy enables the alternating copolymerization of 1,3‐disubstituted bicyclo[1.1.0]butanes with electron‐deficient comonomers, affording well‐defined copolymers bearing strained cyclobutane backbones. A high degree of alternation, tunable molecular weights, and excellent end‐group fidelity are achieved through a CTA‐mediated photoiniferter process. The resulting polymers exhibit high thermal stability, yet undergo efficient degradation under mechanical stimulation.

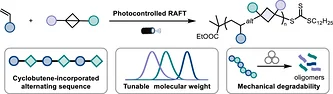

## 1 Introduction

Inspired by the exquisite precision of natural macromolecules, sequence‐controlled polymerization has emerged as a powerful platform for constructing synthetic polymers with diverse and advanced functionalities [1, 2, 3, 4, 5]. Among various sequence‐defined polymers, alternating copolymers are of particular interest due to their facile synthesis [6, 7] and the resulting unique physical [8, 9, 10, 11, 12] and biological properties [13, 14]. To date, significant progress has been made in the synthesis of alternating polymers via radical [15, 16, 17, 18, 19, 20, 21, 22, 23], cationic [24, 25], and ring‐opening metathesis polymerization (ROMP) [26, 27, 28]. Through the judicious design of monomer pairs, these strategies have enabled the precise installation of a wide range of functional groups along polymer backbones, such as esters, amides, fluorinated units, aromatic motifs, and alkenyl groups. More recently, Hong [29, 30] and our group [31, 32] have expanded structural diversity by integrating bio‐derived building blocks, enabling the synthesis of degradable, bio‐based materials. Despite these advances, the scope of compatible monomers remains largely confined to vinyl‐based systems, which restrict backbone diversity and material property space (Figure 1A). Consequently, the development of new monomer classes to access unconventional alternating architecture remains a formidable challenge.

**FIGURE 1:**
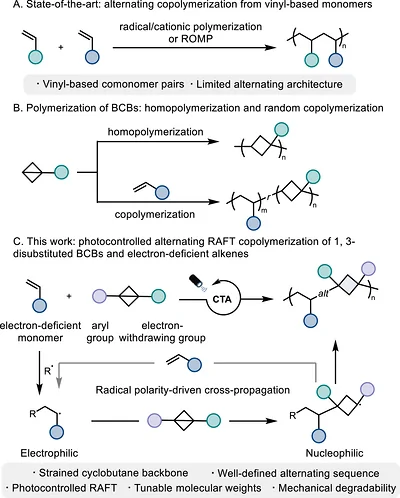
Alternating copolymer synthesis. (A). State‐of‐the‐art alternating copolymers from vinyl‐based monomers. (B). Bicyclo[1.1.0]butanes (BCBs) in homopolymerization and random copolymerization. (C). This work: photocontrolled RAFT copolymerization of 1,3‐disubstituted BCBs and electron‐deficient alkenes.

Bicyclo[1.1.0]butanes (BCBs) represent a compelling monomer class for expanding this landscape. Their exceptionally high ring‐strain energy (ca. 63.9 kcal/mol) promotes efficient ring‐opening processes [33], offering a unique opportunity to incorporate strained cyclobutane frameworks into polymer backbones. Beyond small‐molecule synthesis [34, 35, 36, 37, 38, 39], investigations into BCB‐based polymers have primarily centered on homopolymerization [40, 41, 42, 43, 44, 45, 46] and random copolymerization [47] (Figure 1B). However, sequence‐controlled radical alternating copolymerization of BCBs remains elusive. This limitation originates from a fundamental kinetic challenge in radical alternating copolymerization, where a delicate balance between monomer reactivity and selective cross‐propagation is required [6]. Previous studies have predominantly focused on mono‐substituted, electron‐deficient BCB derivatives, whose intrinsically high reactivity tends to preclude the fine kinetic control required for alternating propagation and instead favors homopolymerization.

To address these limitations, two general strategies have proven effective: (i) enhancing the electronic disparity between comonomers to promote polarity‐driven cross‐propagation, and (ii) introducing steric effects to suppress undesired homopropagation [22, 48, 49]. Guided by these principles, we envisioned that 1,3‐disubstituted BCBs bearing an aryl substituent at one bridgehead and an electron‐withdrawing group at the other are expected to modulate both the electronic properties and steric environment.

In this study, we report a photocontrolled RAFT alternating copolymerization of well‐designed 1,3‐disubstituted bicyclo[1.1.0]butanes (BCBs) with electron‐deficient alkenes (e.g., maleimides, maleic anhydride, and fumaric acid derivatives), providing access to an unprecedented class of well‐defined copolymers featuring strained cyclobutane backbones via polarity‐matched cross‐propagation between electrophilic and nucleophilic radical intermediates. By employing a CTA as a photoiniferter, a library of seventeen BCB‐derived alternating copolymers was readily obtained, exhibiting a high degree of alternation, tunable molecular weights, and excellent end‐group fidelity. Importantly, incorporation of rigid cyclobutane motifs into the polymer backbone imparts remarkable thermal stability, high glass transition temperature (*T*_g_), along with excellent mechanically triggered degradability (Figure 1C).

## 2 Results and Discussion

Based on our design, methyl 3‐phenylbicyclo[1.1.0]butane‐1‐carboxylate (**BCB1**) and *N*‐ethylmaleimide (**EtMI**) were selected as a representative comonomer pair due to their complementary electronic properties. Specifically, this aryl substituent can provide a suitable balance between radical reactivity and stability through resonance effects, which is critical for improving alternation tendency. **BCB1** should form a nucleophilic benzylic radical capable of undergoing selective cross‐addition with **EtMI** [50], which is analogous to that observed in cinnamate ester copolymerization, where an acrylate‐derived radical preferentially attacks the vinyl carbon on the carbonyl‐substituted side of a cinnamate ester to generate a styrenic propagating radical [51]. Initially, thermally initiated free radical polymerization (FRP) was conducted using azobisisobutyronitrile (AIBN) as the initiator at 60°C under equimolar monomer feed conditions. To our delight, the copolymerization of **BCB1** and **EtMI** proceeded smoothly via strain‐release‐driven ring‐opening copolymerization process, affording the copolymer **P(BCB1‐*alt*‐EtMI)** with a number‐average molecular weight (*M*_n_) of 85.7 kDa and a unimodal size‐exclusion chromatography (SEC) trace (Figure 2A, top), albeit with a relatively broad molecular weight distribution (*Đ* = 3.25). Notably, the conversions of **BCB1** and **EtMI** were nearly identical, reaching 60% and 59%, respectively (Scheme S1). Moreover, the **BCB1** content in the resulting copolymer was determined to be 50% (Figure S46), implying an alternating copolymerization process. As expected, monosubstituted BCB (**BCB2**) exhibited high reactivity, leading to nonselective random copolymers with **EtMI** (Figure 2A, middle). While replacing the bridgehead phenyl substituent of BCB with a methyl group (**BCB3**) resulted exclusively in homopolymerization of **EtMI** (Figure 2A, bottom). This highlights the essential role of the phenyl group in finely tuning reactivity and achieving selective cross‐propagation.

**FIGURE 2:**
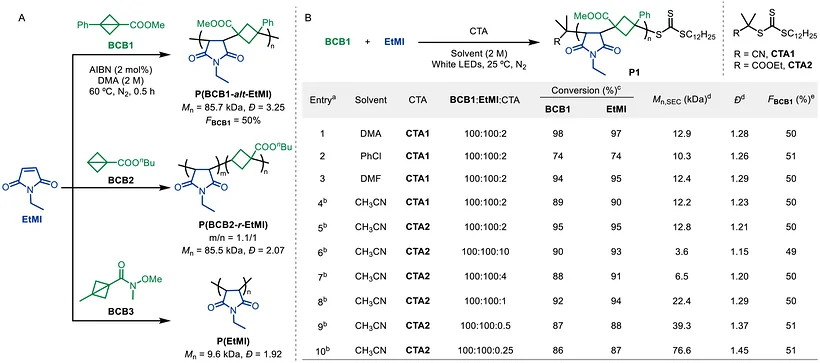
Investigation of RAFT alternating copolymerization of **BCB1** with *N*‐ethylmaleimide (**EtMI**). (A). Top: Thermally initiated alternating copolymerization of **BCB1** and **EtMI**. Middle: copolymerization of **BCB2** and **EtMI**. Bottom: copolymerization of **BCB3** and **EtMI**. (B). Development of photoiniferter‐mediated RAFT alternating copolymerization of **BCB1** and **EtMI**. ^a^ Reaction conditions: **BCB1** (0.2 mmol, 1.0 equiv), **EtMI** (0.2 mmol, 1.0 equiv), CTA (0.004 mmol, 2 mol%), solvent = 100 µL, white LEDs, N_2_, 25°C, 6 h. ^b^ Reaction time = 20 h. ^c^ Monomer conversions were determined by GC using naphthalene as an internal standard. ^d^ *M*_n_ and *Đ* were determined by SEC in THF calibrated with polystyrene (PS) standards. ^e^ The **BCB1** incorporation ratio (*F*_**BCB1**_) was determined by ^1^H NMR of isolated polymer.

We next explored a controlled radical copolymerization using the RAFT strategy [52]. To our delight, the copolymer **P1** (*M*_n_ = 11.2 kDa) was obtained in a controlled manner with markedly reduced dispersity (*Đ* = 1.28) when a trithiocarbonate‐type chain transfer agent **CTA1** was employed (Scheme S1). Given the milder nature of photopolymerization, we further evaluated the feasibility of the copolymerization under photoiniferter‐mediated RAFT processes. Using **CTA1** as a photoiniferter at 25°C under white light‐emitting diodes (LEDs) irradiation, copolymer **P1** was obtained with a *M*_n_ of 12.9 kDa and a *Đ* of 1.28 (Figure 2B, entry 1). Subsequent evaluation of solvent effects (Figure 2B, entries 2–4) revealed that acetonitrile led to a lower dispersity (*Đ* = 1.23). Replacement of **CTA1** with **CTA2** further improved the dispersity to 1.21 and a conversion of up to 95%. (Figure 2B, entry 5). The measured molecular weight (*M*_n, SEC_ = 12.8 kDa) showed good agreement with the theoretical value (*M*_n, theo_ = 14.9 kDa). Notably, Both **EtMI** and **BCB1** showed limited radical homopolymerization (Scheme S14), with only 10% and less than 5% conversion, respectively.

To clarify the photoactivation mechanism of **CTA2**, radical trapping experiments and UV‐vis absorption measurements were performed. Upon irradiation of **CTA2** with white LEDs in the presence of 2,2,6,6‐tetramethyl‐1‐piperidinyloxy (TEMPO, 2.0 equiv), but in the absence of either BCB or maleimide monomers, a **CTA2**‐derived TEMPO adduct was detected by HR‐MS (Figure S3). Furthermore, UV‐vis analysis showed that **CTA2** absorbs within the emission range of the white‐light source, whereas neither **BCB1** nor **EtMI** exhibits absorption in this region (Figure S4). Mixing **CTA2** with either one or both monomers caused no noticeable change in its absorption spectrum (Figure S5). Collectively, these results indicate that **CTA2** can be activated through direct photoinduced homolysis without requiring photosensitization by monomers.

Leveraging the characteristic features of RAFT copolymerization, alternating copolymers with tunable molecular weights (*M*_n_ = 3.6−76.6 kDa) and low to moderate dispersities (*Đ* = 1.15−1.45) can be readily achieved by varying the monomer‐to‐CTA ratio ([M]_0_:[CTA]_0_ = 10−400:1) (Figure 2B, entries 5−10). The slightly higher dispersity values observed at high [M]_0_:[CTA]_0_ ratios may be attributed to reduced chain‐transfer efficiency at low CTA concentrations. Notably, a consistent 1:1 incorporation ratio of **BCB1** and **EtMI** was maintained across all polymerizations, further supporting the robust alternating nature of this system. More importantly, kinetic studies revealed that both monomers were consumed at nearly identical rates, while the **BCB1** content remained approximately 50% throughout the polymerization (Figure 3A). Finally, synchronous incorporation was further validated by Mayo−Lewis analysis [53], where reactivity ratios of **BCB1** and **EtMI** were determined to be *r* ***_BCB1_*** = 0.014 *and r* ***_EtMI_*** = 0.011, respectively. These near‐zero values confirm a strong preference for cross‐propagation over homopropagation (Figure 3B). By contrast, for the monosubstituted **BCB2**/**EtMI** system, kinetic studies under the same photopolymerization conditions showed that **BCB2** was consumed slightly faster than **EtMI** throughout the polymerization (Table S6 and Figure S16). The reactivity ratios were further measured under thermal free‐radical conditions to obtain polymers with sufficient molecular weight for purification and reliable Mayo‐Lewis analysis, giving *r* **_BCB2_** = 0.356 and *r* ***_EtMI_*** = 0.436, respectively (Figure S19). These significantly larger values than those of **BCB1**/**EtMI** system indicate its low tendency toward alternation, highlighting the important role of disubstituted BCBs in promoting selective cross‐propagation.

**FIGURE 3:**
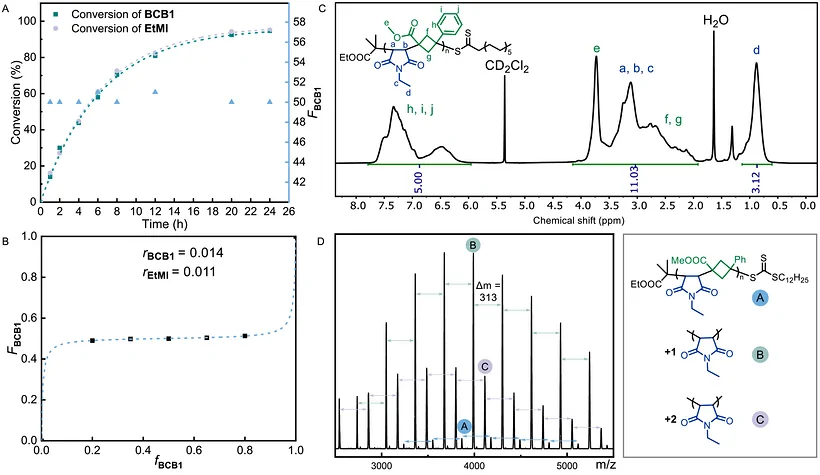
Characterization of alternating copolymerization of **BCB1** and **EtMI**. (A). Plots of monomer conversions and *F*_**BCB1**_ versus time. (B). Mayo−Lewis plot of the **BCB1** incorporation ratio (*F* **_BCB1_**) versus **BCB1** feed composition (*f* **_BCB1_**). (C). ^1^H NMR spectrum of **P1**. (D). MALDI‐TOF mass spectrum of **P1**.

The ^1^H nuclear magnetic resonance (NMR) spectrum of the resulting polymer displayed well‐resolved resonances corresponding to **BCB1** and **EtMI** segments (Figure 3C). To further confirm the alternation, matrix‐assisted laser desorption/ionization time‐of‐flight mass spectrometry (MALDI‐TOF MS) analysis was performed on a low‐molecular‐weight sample (Figure 3D), exhibiting three distinct peak series with a repeating unit mass of 313 Da, consistent with the combined mass of **BCB1** and **EtMI**. Ion series A was assigned to a perfectly alternating sequence with equal incorporation of two monomers, whereas series of B and C were attributed to copolymers containing one and two additional **EtMI** units, respectively. These extra **EtMI** units likely originated from the initiation or termination stages rather than the propagation step, as the latter would lead to more complex mass spectral patterns.

In addition to the well‐controlled *M*_n_ and low *Đ* (Figure 4A), first‐order kinetic behavior (Figure S13) and the linear growth in molecular weight over monomer conversion (Figure 4B) further supported the controlled polymerization. The relatively broad molecular weight distribution observed at the early stage may stem from the slow activation of the CTA under light irradiation, as an induction period is typically required to establish a dynamic equilibrium between dormant and active species. To validate the livingness and the excellent *ω*‐end group fidelity of the copolymers, we further investigated the synthesis of block‐alternating copolymers. To a macroinitiator **P1** (*M*_n_ = 6.5 kDa, *Đ* = 1.20) prepared from **BCB1** and **EtMI** at a M/I ratio of 25, subsequent chain extension with **BCB1** and maleimide derivative **S15** successfully afforded the desired diblock copolymer **P1‐*b*‐P15** (*M*_n_ = 18.4 kDa, *Đ* = 1.37). The successful chain extension was confirmed not only by a complete shift of the SEC trace to a shorter retention time (Figure 4C), but also by a single diffusion signal in the diffusion‐ordered spectroscopy (DOSY) NMR analysis (Figure S21). Moreover, addition of methyl acrylate (MA) produced another diblock copolymer, **P1**‐***b***‐**PMA**, under modified conditions using DMA as the solvent and blue LEDs as the light source (Scheme S17; Figures S22 and S23).

**FIGURE 4:**
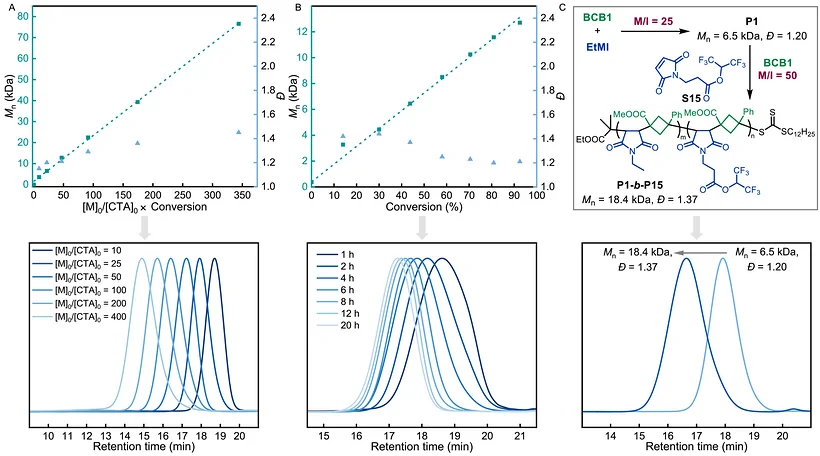
Characteristics of the RAFT copolymerization. (A). Plots of *M*_n_ and *Đ* as a function of [M]_0_/[CTA]_0_ × conversion and the corresponding SEC traces. (B). Plots of *M*_n_ and *Đ* as a function of monomer conversion and the corresponding SEC traces. (C). Synthesis of diblock alternating copolymer.

To test the generality of the established alternating copolymerization method, various BCBs and maleimide derivatives were evaluated (Figure 5). BCB monomers bearing methyl, ethyl, and isopropyl ester functionalities underwent efficient RAFT alternating copolymerization with **EtMI**, affording the corresponding copolymers **P1–P3** with molecular weight ranging from 12.8 to 14.7 kDa and low dispersity (*Đ* = 1.17–1.22). Notably, MALDI‐TOF MS analysis of **P2** revealed a dominant alternating sequence distribution (Figure S70), further supporting its high alternation. Beyond ester‐substituted BCBs, the scope could be readily extended to Weinreb amide‐ and ketone‐functionalized BCBs (**P4** and **P5**), both of which exhibited approximately 50% BCB incorporation. We next turned our attention to the scope of maleimides using an amide‐substituted BCB as the comonomer. A wide variety of maleimide derivatives bearing linear alkyl substituents, such as *n*‐butyl and benzyl groups, as well as branched alkyl substituents, including 2‐ethylhexyl and cyclohexyl groups, were all well tolerated under optimized conditions (**P6–P9**). In addition, phenyl‐substituted maleimide also proved to be a suitable comonomer (**P10**). Encouraged by these results, we further explored maleimides featuring polar and multifunctional side chains. Notably, maleimides containing alkyl hydroxyl, oligo(ethylene glycol) (OEG), and poly(ethylene glycol) (PEG) moieties were successfully incorporated (**P11–P13**), while maintaining narrow *Đ*. Furthermore, considering the importance of fluorinated polymers in advanced materials science [54], the monomer scope was further expanded to maleimides bearing fluoroalkyl substituents and hexafluoroisopropyl (HFIP) ester functionalities, affording copolymers **P14** and **P15** with low dispersity of 1.16 and 1.22, respectively. Importantly, this protocol is not restricted to maleimides, as maleic anhydride was also found to be compatible under standard conditions (**P16**). More significantly, the biomass‐derived monomer dimethyl fumarate could also be successfully copolymerized with BCBs, leading to the formation of a bio‐based alternating copolymer (**P17**) under modified conditions at 60°C using either AIBN/**CTA2** or white LEDs/**CTA2** system (Table S2). It is worth noting that the conversion of both monomers remained below 5% upon irradiation with white LEDs at room temperature, presumably due to the sterically hindered structure of dimethyl fumarate, which renders radical propagation kinetically unfavorable. The alternating sequence of all copolymers was confirmed by ^1^H NMR analysis, and further supported by MALDI‐TOF MS characterization for all samples except **P13** (Figures S69–S84).

**FIGURE 5:**
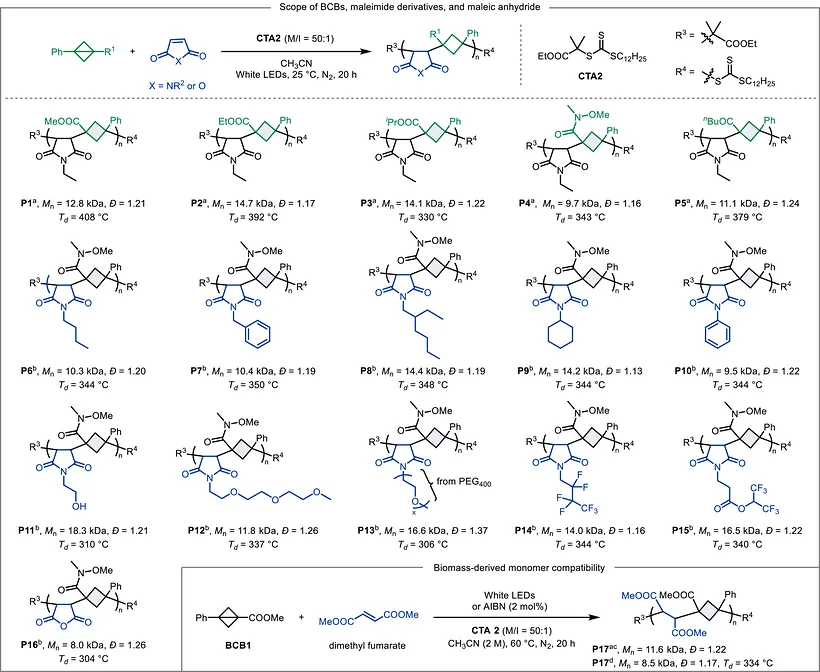
Monomer scope. ^a^ Reaction conditions: BCBs (0.2 mmol, 1.0 equiv), comonomer (maleimide derivatives, maleic anhydride or dimethyl fumarate; 0.2 mmol, 1.0 equiv), **CTA2** (0.004 mmol, 2 mol%), acetonitrile = 100 µL, white LEDs, N_2_, 25°C, 20 h. ^b^ Acetonitrile = 150 µL was used. ^c^ 60°C was used. ^d^ Reaction conditions: **BCB1** (0.2 mmol, 1.0 equiv), dimethyl fumarate (0.2 mmol, 1.0 equiv), AIBN (0.004 mmol, 2 mol%), **CTA2** (0.004 mmol, 2 mol%), acetonitrile = 100 µL, N_2_, 60°C, 20 h.

The thermal properties of these BCB‐derived alternating copolymers were investigated using thermogravimetric analysis (TGA) and differential scanning calorimetry (DSC). The resulting copolymers exhibited excellent thermal stability, with onset decomposition temperatures (*T*_d_, defined by the temperature of 5% weight loss) ranging from 304°C to 408°C (Figures 5 and S106–S123). TGA curves of representative copolymers (**P1–P5**) were displayed in Figure 6A, **P1** with a higher *M*_n_ of 76.6 kDa showed even better thermal stability with *T*_d_ of 429°C (Figure S107). As expected, relatively high glass‐transition temperatures (*T*_g_) were observed, ranging from 186°C to 233°C (**P1**, **P2**, **P3**, and **P5**, Figure 6B), which can be attributed to the inherent rigidity and steric hindrance imparted by the strained‐ring units in the polymer backbone.

**FIGURE 6:**
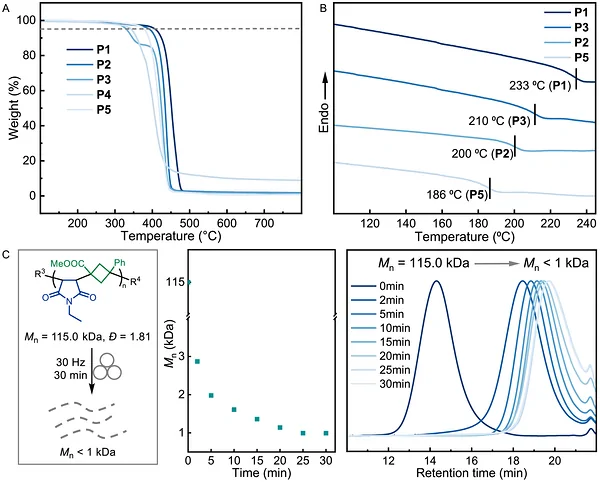
Material properties of BCB‐based alternating copolymers. (A). Overlay of TGA curves of representative alternating copolymers. (B). Overlay of DSC curves of representative alternating copolymers. (C). Ball‐mill grinding degradation of high‐molecular‐weight **P1**, molecular weight of **P1** and the resulting oligomers as a function of grinding time, and the corresponding SEC traces.

Mechanically triggered degradation in the bulk state offers an attractive strategy for addressing plastic waste while avoiding the use of additional chemical reagents [55]. Given the high efficiency of ball‐mill grinding (BMG) for mechanophore activation, we further explored the possibility of bulk mechanical degradation of the resulting alternating copolymers (Figure 6C). Notably, a high‐molecular‐weight sample (*M*_n, SEC_ = 115.0 kDa) exhibited rapid chain scission under BMG conditions. Time‐resolved degradation studies revealed a significant reduction in molecular weight from 115 to 2.9 kDa within 2 min of ball milling at 30 Hz. Prolonged treatment for 30 min yielded low‐molecular‐weight oligomers (*M*_n, SEC_ < 1 kDa, more than 100‐fold *M*_n_ reduction). MALDI‐TOF MS analysis of the resulting oligomers suggested that mechanical force facilitates cleavage of the carbon–carbon backbone bonds between repeating units, along with fragmentation of the RAFT‐derived chain‐end groups (Figure S25). Other copolymers bearing ethyl ester, isopropyl ester, and Weinreb amide‐functionalized side chains also underwent similar BMG degradation (Tables S8–S10 and Figures S26–S28). These results demonstrate that efficient backbone degradation is maintained across a range of structurally distinct side chains. To better understand this exceptionally fast degradation despite its high thermal stability, we evaluated the intrinsic mechanical strength of the polymer backbone via density functional theory (DFT) and relaxed potential energy surface (PES) scans. By systematically elongating specific bonds, we found that the **P(BCB1‐*alt*‐EtMI)** backbone can be cleaved at a critical force of 4.31 nN. which is significantly lower than that of a commodity polymer, polystyrene (4.97 nN) [56]. It is interesting to note that polymers with sterically crowded main‐chain containing strained rings exhibit great thermal stability, but also excellent degradation under mechanical stress.

## 3 Conclusion

In summary, we have demonstrated the successful incorporation of strained cyclobutane units into alternating copolymer backbones through photocontrolled RAFT copolymerization. The key to this achievement lies in the rational design of BCB monomers bearing an aryl bridgehead and an electron‐withdrawing substituent at the opposite position, which enables effective modulation of radical reactivity and propagation behavior. Comprehensive spectroscopic analysis and kinetic studies further confirmed the highly alternating sequence along the polymer main chains. In addition, the well‐controlled molecular weights, low dispersity, and successful synthesis of block alternating copolymers highlight the living nature of this polymerization system. Importantly, this strategy exhibits broad monomer scope and excellent functional group compatibility, enabling the synthesis of a library of seventeen alternating copolymers. The resulting polymers display excellent thermal stability, high glass transition temperatures, but at the same time, efficient mechanically induced degradability. This work expands the scope of alternating copolymerization beyond conventional vinyl‐based systems, providing a robust platform for precision polymer sequence engineering.

## Author Contributions

**Xia Hu**: data curation, writing – review and editing, formal analysis, supervision, project administration, validation, methodology, conceptualization, investigation, writing – original draft. **Hongsik Kim**: validation, formal analysis, writing – review and editing, data curation. **Lianqian Wu**: writing – review and editing, validation. **Yi Jiang**: writing – review and editing, validation. **Tae‐Lim Choi**: conceptualization, writing – review and editing, funding acquisition, project administration, methodology, validation, investigation, formal analysis, supervision, data curation, resources, writing – original draft.

## Conflicts of Interest

The authors declare no conflicts of interest.

## Supporting information




**Supporting File**: anie74096‐sup‐0001‐SuppMat.pdf.

## Data Availability

The data that support the findings of this study are available in the Supporting Information of this article.
