# Supplementary material for: Degradable Alternating Copolymers With Strained Cyclobutane Backbones via Photocontrolled RAFT Polymerization
Source: Angew Chem Int Ed Engl. 2026 Aug 8;65(41):e7228967. doi: 10.1002/anie.7228967 (PMC13638088; doi:10.1002/anie.7228967)
Supplement: Supplementary file 1 [file ANIE-65-e7228967-s001.pdf]

Degradable alternating copolymers with strained cyclobutane backbones via photocontrolled RAFT polymerization

Xia Hu, Hongsik Kim, Lianqian Wu, Yi Jiang, Tae-Lim Choi*
Department of Materials, ETH Zürich, Zürich 8093, Switzerland.
*Email: tae-lim.choi@mat.ethz.ch

Table of content

1. General information 2
2. Investigation of RAFT Alternating Copolymerization of **BCB1** with **EtMI** 3
3. Experimental Procedures and Characterization Data of Polymers 4
4. Experimental Procedures and Characterization Data of BCBs 11
5. Experimental Procedures and Characterization Data of Maleimide Derivatives 15
6. Photoiniferter-mediated Homopolymerization of **BCB1** and **EtMI** 18
7. Radical Trapping Experiment 19
8. UV-vis Analysis 20
9. Solvent Effects on Photocontrolled RAFT Alternating Copolymerization 21
10. Molecular Weight Control Experiments 24
11. Kinetic Studies 25
   11-1. Kinetic studies of alternating copolymerization of **BCB1** and **EtMI** 25
   11-2. Kinetic studies of copolymerization of **BCB2** and **EtMI** 27
12. Determination of Reactivity Ratios 29
   12-1. Reactivity ratios of **BCB1**/**EtMI** system 29
   12-2. Reactivity ratios of **BCB2**/**EtMI** system 30
13. Chain Extension 30
14. Mechanochemical Degradation via Ball-mill Grinding 33
15. Computational Details 37
16. References 42
17. NMR spectra 44
18. MALDI-TOF MS spectra 64
19. SEC traces 80
20. TGA curves 90
21. DSC curves 100

1. General information

All manipulations of air- and moisture-sensitive materials were carried out under nitrogen in a glovebox or using the standard Schlenk line techniques. Starting materials and anhydrous solvents were purchased from Sigma-Aldrich or TCI companies. Anhydrous acetonitrile and DMA were purified by passage through basic aluminum oxide, degassed, and stored under nitrogen in a glovebox. Photochemical experiments have been performed using EvoluChem 18 W 6200K white LEDs. Gas chromatography (GC) measurements were conducted on a Shimadzu GC-2025 Series GC system. UV-vis absorption spectra were obtained using an Agilent Cary-60 UV-vis spectrometer. All NMR Spectra were recorded on Bruker Avance spectrometers operating at 300, 400, 500, or 500 (Cryo) MHz. Chemical shifts are given in ppm and the spectra are calibrated using the residual chloroform signals (7.26 ppm for 1H NMR and 77.0 ppm for ^{13}C NMR) and the residual dichloromethane signals (5.32 ppm for ^{1}H NMR and 53.84 ppm, for ^{13}C NMR). Multiplicities are abbreviated as follows: singlet (s), doublet (d), triplet (t), quartet (q), doublet-doublet (dd), quintet (quint), septet (sept), multiplet (m), and broad (b). Matrix-Assisted Laser Desorption/Ionization Time-of-Flight Mass Spectrometry (MALDI-TOF MS) analyses were performed on a Bruker Auto Flex MaX instrument (positive linear mode). DCTB was used as the matrix and NaTFA as the cation source. High-resolution mass spectrometry (HRMS) analysis was conducted using an ultrahighresolution ESI Q-TOF mass spectrometer (Bruker, Germany) at the MoBiAS, ETH Zurich. Size-exclusion chromatography (SEC) analyses were carried out using an Agilent 1260 Infinity II SEC system with a Wyatt OptiLab T-rEx refractive index (RI) detector. The SEC system was equipped with two Shodex LF-804 columns, eluted with THF at 35 °C at a rate of 1.0 mL/min and calibrated using polystyrene standards. For **P11**, molecular weights were determined by prominence series Shimadzu SEC using DMAc as the eluent at a flow rate of 1.0 mL/min through three KF-805L (300 × 8 mm, bead size 10 μm, pore size maximum: 5000 Å) columns at 40 °C with a RID-20A refractive index detector. The instrument was calibrated with poly(methyl methacrylate) external standards (Aglient Technologies, Polymethyl Methacrylate EasiVials, molecular weights range from 1.6 × 106 to 7 × 103 g/mol). Thermogravimetric Analysis (TGA) analysis was obtained using a Waters Discovery TGA 5500, with 5 mg of a given sample at a heating rate of 10 ºC/min from 30 to 800 ºC under nitrogen gas. Differential Scanning Calorimetry (DSC) analysis was recorded on a Waters Discovery DSC 2500. All T_g values were obtained from a second scan, and the heating/cooling rate was 30 ºC/min.

2. Investigation of RAFT Alternating Copolymerization of BCB1 with EtMI

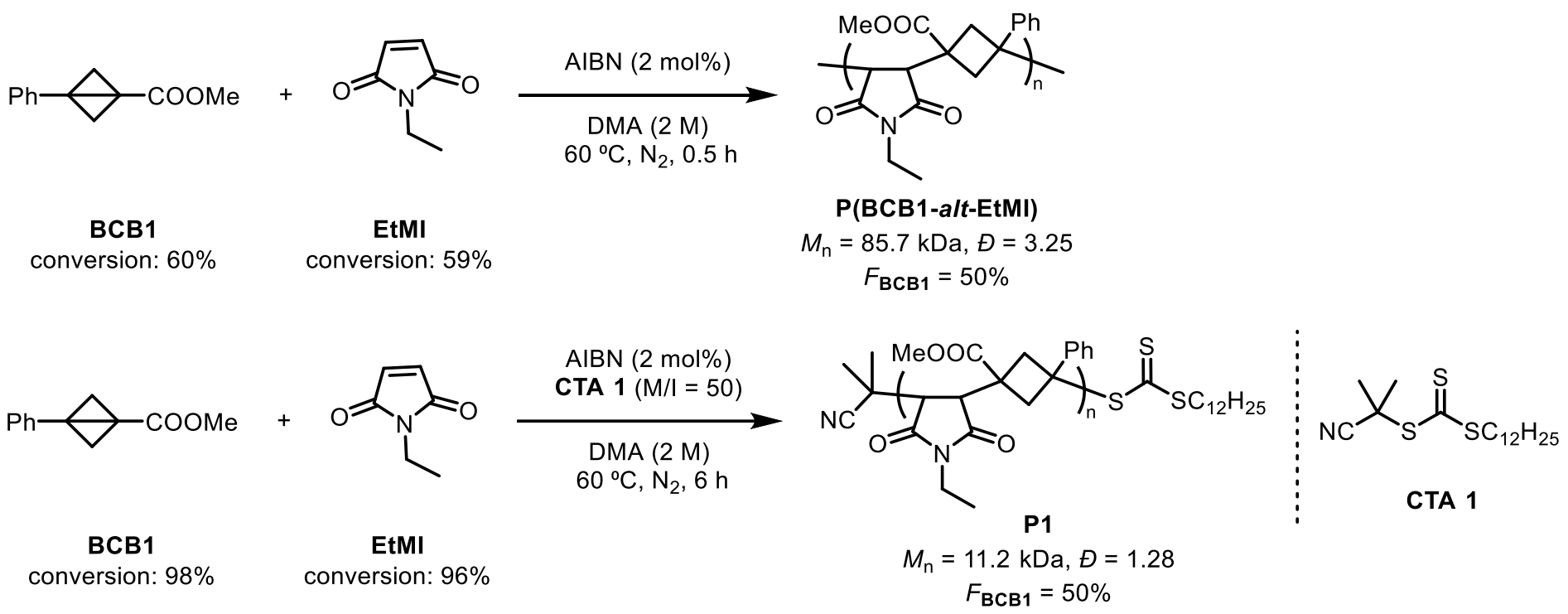

Scheme S1. Thermally initiated alternating copolymerization of BCB1 with EtMI.

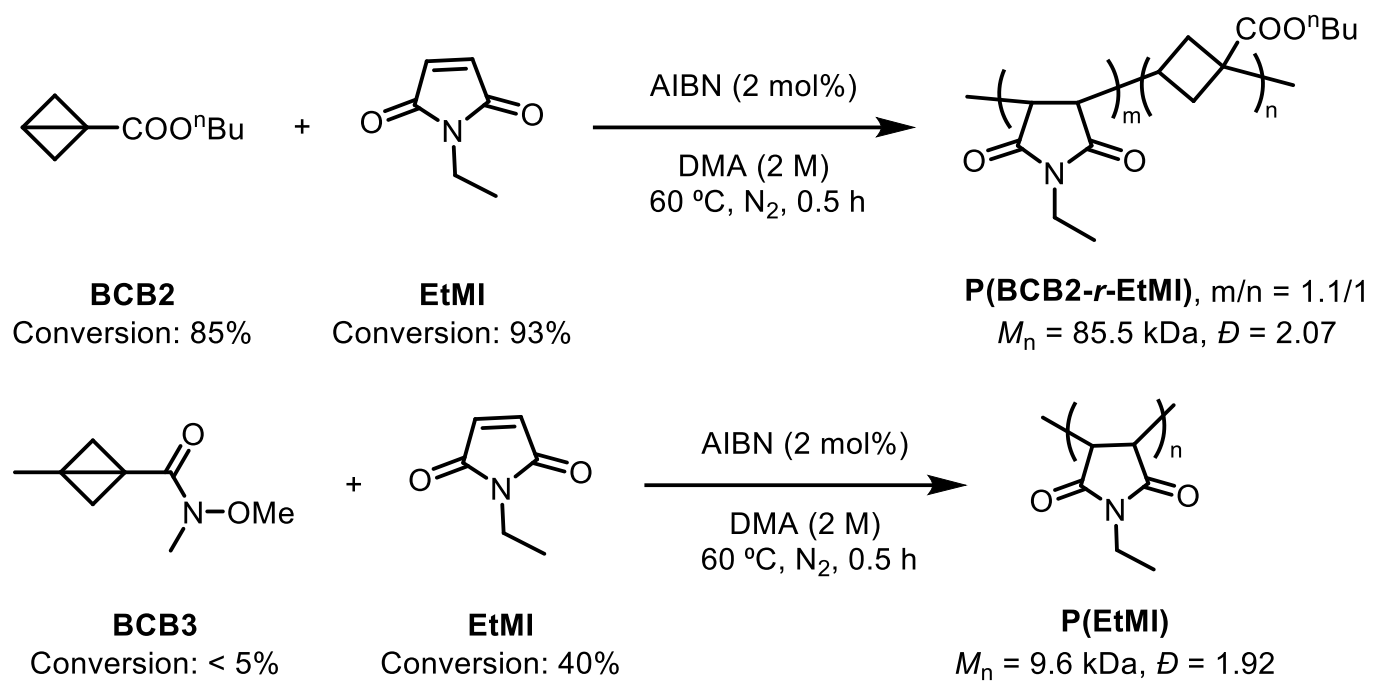

Scheme S2. Reactivity of BCB2 (or BCB3) with EtMI under Thermally initiated conditions.

Table S1. Condition optimization of photocontrolled RAFT alternating copolymerization of BCB1 with EtMI

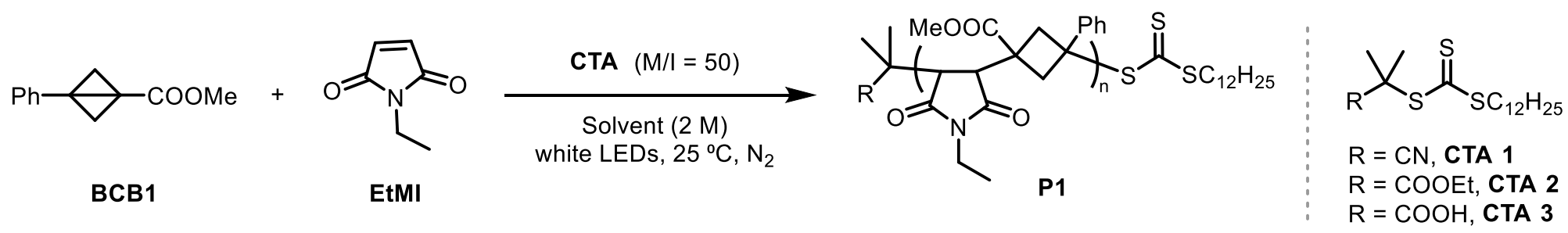

Entry[a]	Solvent	CTA	Conversion (%)[e] BCB1	Conversion (%)[e] EtMI	$M_{n,SEC}$ (kDa)[f]	$Đ$[f]	F_{BCB1} (%)[g]
1	DMA	**CTA1**	98	97	12.9	1.28	50
2	DMA	**CTA2**	87	88	11.7	1.28	50
3	DMA	**CTA3**	87	87	11.5	1.37	50
4	PhCl	**CTA1**	74	74	10.3	1.26	51
5	DMF	**CTA1**	94	95	12.4	1.29	50
6	CH_3CN	**CTA1**	61	64	8.7	1.20	50
7[b]	CH_3CN	**CTA1**	94	97	12.3	1.23	50
8[c]	CH_3CN	**CTA1**	>99	>99	16.9	1.43	50
9[d]	CH_3CN	**CTA1**	89	90	12.2	1.23	50
10[d]	Acetone	**CTA1**	85	87	10.6	1.23	50
11[d]	EtOAc	**CTA1**	92	95	12.6	1.26	50
12[d]	CH_3CN	**CTA2**	95	95	12.8	1.21	50

[a] Reaction conditions: **BCB1** (0.2 mmol, 1.0 equiv), **EtMI** (0.2 mmol, 1.0 equiv), **CTA** (0.004 mmol, 2 mol%), solvent = 100 μL, white LEDs, N_2, 25 °C, 6 h. [b] 4CzIPN (0.1 mmol%) was added as photocatalyst. [c] $Ir(ppy)_3$ (0.1 mmol%) was added as photocatalyst. [d] reaction time = 20 h. [e] Monomer conversions were determined by GC using naphthalene as an internal standard. [f] M_n and $Đ$ were determined by SEC in THF. [g] The **BCB1** incorporation ratio (F_{BCB1}) was determined by ^{1}H NMR of isolated polymer.

Table S2. Optimization of the alternating copolymerization conditions for dimethyl fumarate and BCB1

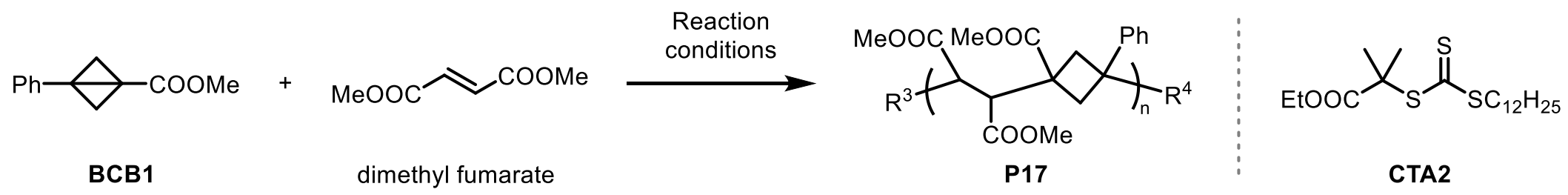

Entry	Reaction conditions	Conversion (%)[d] BCB1	Conversion (%)[d] dimethyl fumarate	$M_{n,SEC}$ (kDa)[e]	$Đ$[e]
1[a]	**CTA2** (M/I = 50:1)<br>CH_3CN (2 M)<br>White LEDs, 25 °C, N_2, 20 h	< 5	<5	--	--
2[ab]	**CTA2** (M/I = 50:1)<br>CH_3CN (2 M)<br>White LEDs, 60 °C, N_2, 20 h	61	58	11.6	1.22
3[c]	AIBN (2 mol%)<br>**CTA 2** (M/I = 50:1)<br>CH_3CN (2 M), 60 °C, N_2, 20 h	49	47	8.5	1.17

[a] Reaction conditions: **BCB1** (0.2 mmol, 1.0 equiv), dimethyl fumarate (0.2 mmol, 1.0 equiv), **CTA 2** (0.004 mmol, 2 mol%) , CH_3CN = 100 μL, white LEDs, N_2, 25 °C, 20 h. [b] 60 °C was used. [c] Reaction conditions: **BCB1** (0.2 mmol, 1.0 equiv), dimethyl fumarate (0.2 mmol, 1.0 equiv), AIBN (0.004 mmol, 2 mol%), **CTA2** (0.004 mmol, 2 mol %), acetonitrile = 100 μL, N_2, 60 °C, 20 h. [d] Monomer conversions were determined by GC using naphthalene as an internal standard. [e] M_n and $Đ$ were determined by SEC in THF.

3. Experimental Procedures and Characterization Data of Polymers

General Procedure A (GP-A):

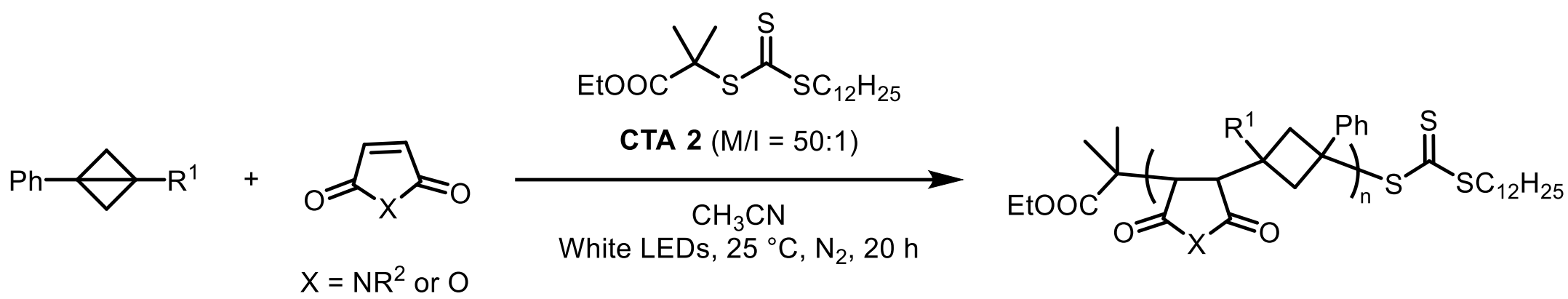

Scheme S3. General procedure A for the photoinduced RAFT alternating copolymerization of BCBs with electron-deficient alkenes.

GP-A: In a nitrogen-filled glovebox, a 5 mL reaction vial equipped with a magnetic stirring bar was charged with BCBs (0.2 mmol) and maleimide derivatives (0.2 mmol) or maleic anhydride (0.2 mmol), followed by addition of **CTA2** (2 mol%, 0.004 mmol, 100 μL of a 0.04 M solution in acetonitrile; for Weinreb amide-substituted BCBs, an additional 50 μL of acetonitrile was added). The vial was sealed, removed from the glovebox, and stirred under white light irradiation at 25 °C for 20 h. Upon completion, DCM was added to dissolve the reaction mixture, and naphthalene was added as an internal standard. Monomer conversion was determined by gas chromatography (an aliquot of the reaction mixture was subjected to precipitation treatment, and the supernatant was used for GC analysis). The polymer was then precipitated into Et_2O, or a pentane/Et_2O mixture, or MeOH, followed by centrifugation and decantation. The precipitated polymer was redissolved in DCM, and this precipitation/redissolution procedure was repeated three times to ensure complete removal of residual initiator, internal standard and unreacted monomer. The resulting polymer was dried in a high-vacuum oven for 12 h to constant weight. Note: polymerizations were conducted in an EvoluChem PhotoRedOx Box TC™ equipped with air cooling and illuminated by an EvoluChem 18 W 6200 K white light source.

General Procedure B (GP-B):

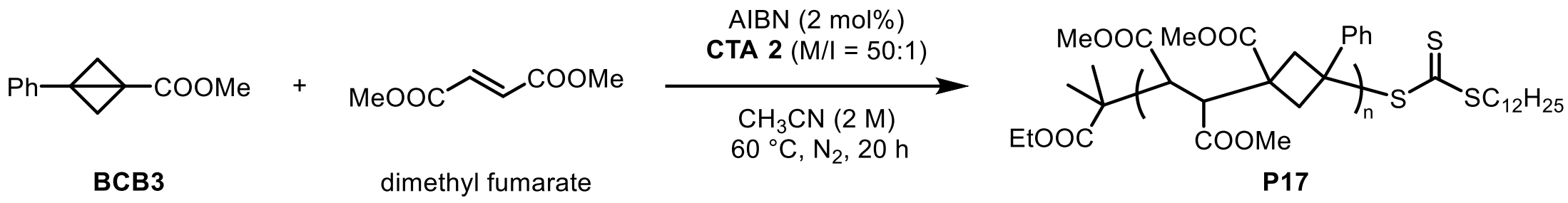

Scheme S4. General procedure B for the synthesis of **P17**.

GP-B: In a nitrogen-filled glovebox, a 5 mL reaction vial equipped with a magnetic stirring bar was charged with **BCB3** (0.2 mmol), dimethyl fumarate (0.2 mmol), AIBN (2 mol%, 0.004 mmol), followed by addition of **CTA2** (2 mol%, 0.004 mmol, 100 µL of a 0.04 M solution in acetonitrile). The vial was sealed, removed from the glovebox, and stirred at 60 °C for 20 h. Upon completion, DCM was added to dissolve the reaction mixture, and naphthalene was added as an internal standard. Monomer conversion was determined by gas chromatography (an aliquot of the reaction mixture was subjected to precipitation treatment, and the supernatant was used for GC analysis). The polymer was then precipitated into Et_2O, followed by centrifugation and decantation. The precipitated polymer was redissolved in DCM, and this precipitation/redissolution procedure was repeated three times to ensure complete removal of residual initiator, internal standard and unreacted monomer. The resulting polymer was dried in a high-vacuum oven for 12 h to constant weight.

General Procedure C (GP-C):

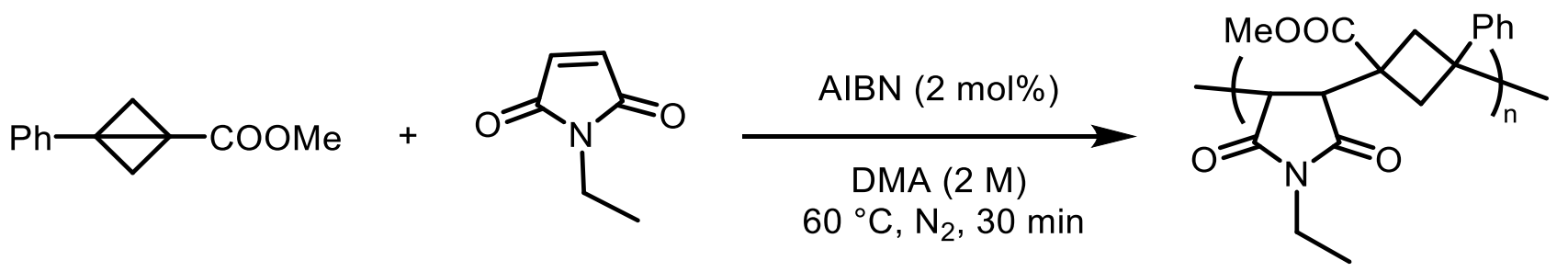

Scheme S5. General procedure C for the free radical copolymerization of **BCB3** with **EtMI**.

GP-C: In a nitrogen-filled glovebox, a 5 mL reaction vial equipped with a magnetic stirring bar was charged with **BCB3** (0.2 mmol), **EtMI** (0.2 mmol), AIBN (2 mol%, 0.004 mmol), and DMA (100 µL). The vial was sealed, removed from the glovebox, and stirred at 60 °C for 2 h. Upon completion, DCM was added to dissolve the reaction mixture, and naphthalene was added as an internal standard. Monomer conversion was determined by gas chromatography (an aliquot of the reaction mixture was subjected to precipitation treatment, and the supernatant was used for GC analysis). The polymer was then precipitated into Et_2O, followed by centrifugation and decantation. The precipitated polymer was redissolved in DCM, and this precipitation/redissolution procedure was repeated three times to ensure complete removal of residual initiator, internal standard and unreacted monomer. The resulting polymer was dried in a high-vacuum oven for 12 h to constant weight. Note: The reactivity between **BCB2** (or **BCB3**) and **EtMI** was also investigated following the same procedure. In addition, the homopolymerization of **BCB1** was performed under modified conditions, with the reaction time extended to 24 h.

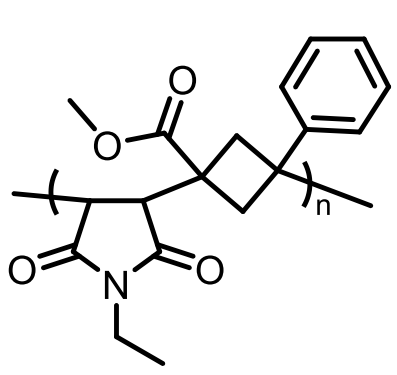

P(BCB1-*alt*-EtMI)

Prepared by **GP-C**. White powder, 53.0 mg, 85% yield. ^{1}H NMR (400 MHz, CD_2Cl_2) δ 7.76 – 5.90 (m, 5H), 4.09 – 1.81 (m, 11H), 1.12 – 0.52 (m, 3H). SEC (THF): M_n = 85.7 kDa, $Đ$ = 3.25. TGA: T_d = 429 ºC.

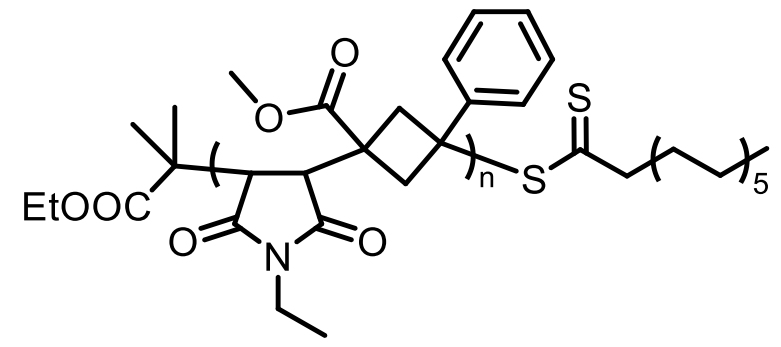

P1

Prepared by **GP-A**. White powder, 53.5 mg, 85% yield. ^{1}H NMR (400 MHz, CD_2Cl_2) δ 7.81 – 5.94 (m, 5H), 4.08 – 1.85 (m, 11H), 1.08 – 0.49 (m, 3H, the end-group signal peaks overlap). SEC (THF): M_n = 12.8 kDa, $Đ$ = 1.21. TGA: T_d = 408 ºC. DSC: T_g = 233 ºC.

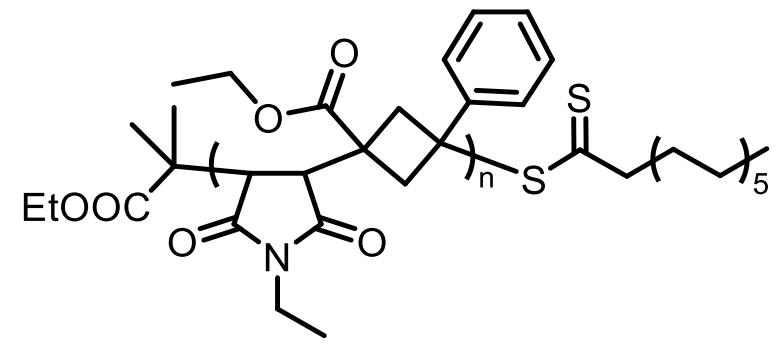

P2

Prepared by **GP-A**. White powder, 60.6 mg, 92% yield. ^{1}H NMR (500 MHz, CD_2Cl_2) δ 7.86 – 5.89 (m, 5H), 4.41 – 1.85 (m, 10H), 1.45 – 0.46 (m, 6H, the end-group signal peaks overlap). SEC (THF): M_n = 14.7 kDa, $Đ$ = 1.17. TGA: T_d = 392 ºC. DSC: T_g = 200 ºC.

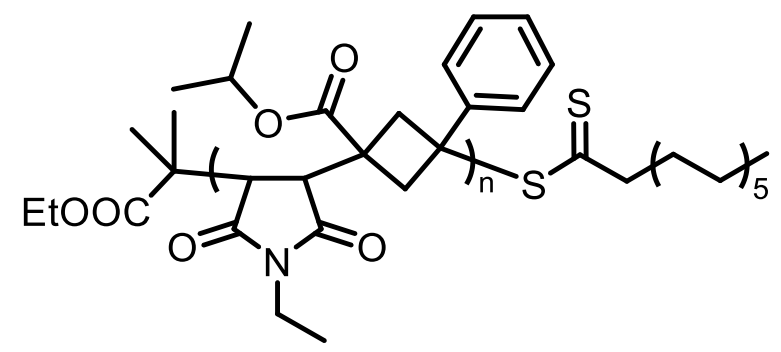

P3

Prepared by **GP-A**. White powder, 55.6 mg, 82% yield. ^{1}H NMR (400 MHz, CD_2Cl_2) δ 7.99 – 5.71 (m, 5H), 5.20 – 4.10 (m, 1H), 4.01 – 1.69 (m, 8H), 1.45 – 0.44 (m, 9H, the end-group signal peaks overlap). SEC (THF): M_n = 14.1 kDa, $Đ$ = 1.22. TGA: T_d = 330 ºC. DSC: T_g = 210 ºC.

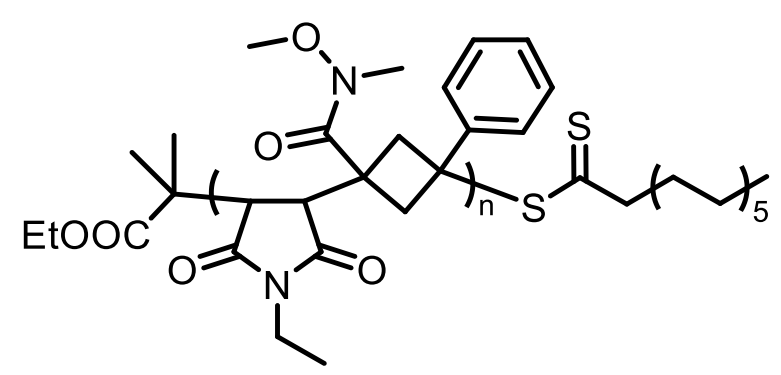

P4

Prepared by **GP-A**. White powder, 58 mg, 85% yield. ^{1}H NMR (400 MHz, CD_2Cl_2) δ 8.23 – 5.51 (m, 5H), 4.30 – 1.92 (m, 14H), 0.94 – 0.32 (m, 3H, the end-group signal peaks overlap). SEC (THF): M_n = 9.7 kDa, $Đ$ = 1.16. TGA: T_d = 343 ºC. The T_g could not be detected.

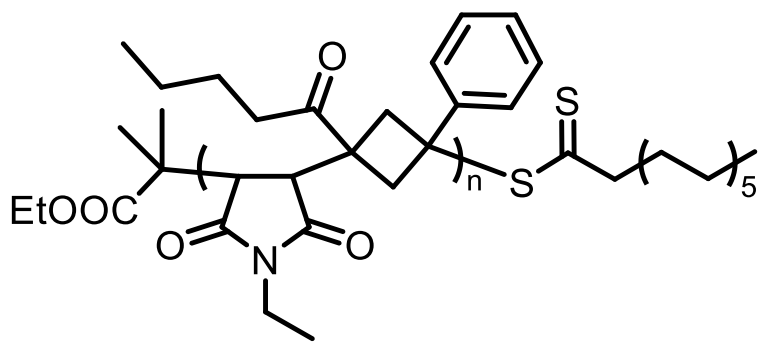

P5

Prepared by **GP-A**. White powder, 55.4 mg, 82% yield. ^{1}H NMR (400 MHz, CD_2Cl_2) δ 7.95 – 5.77 (m, 5H), 3.80 – 1.70 (m, 10H), 1.53 – 0.27 (m, 10H, the end-group signal peaks overlap). SEC (THF): M_n = 11.1 kDa, $Đ$ = 1.24. TGA: T_d = 379 ºC. DSC: T_g = 186 ºC.

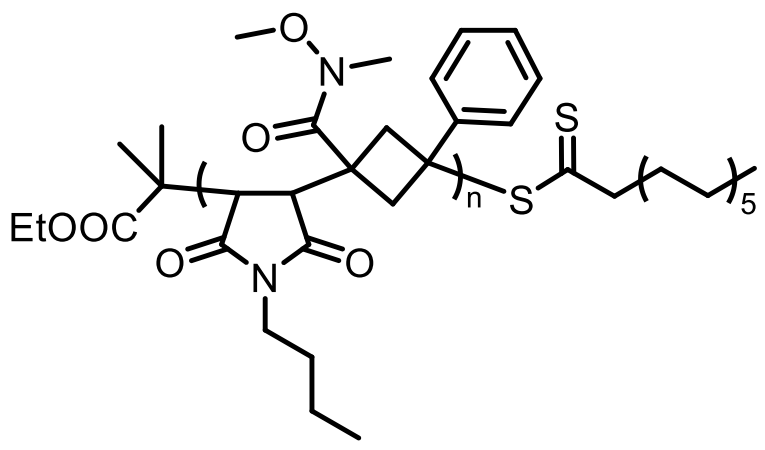

P6

Prepared by **GP-A**. White powder, 58 mg, 85% yield. ^{1}H NMR (400 MHz, CD_2Cl_2) δ 8.47 – 5.36 (m, 5H), 4.28 – 1.88 (m, 14H), 1.27 – 0.39 (m, 7H, the end-group signal peaks overlap). SEC (THF): M_n = 10.3 kDa, $Đ$ = 1.20. TGA: T_d = 344 ºC. The T_g could not be detected.

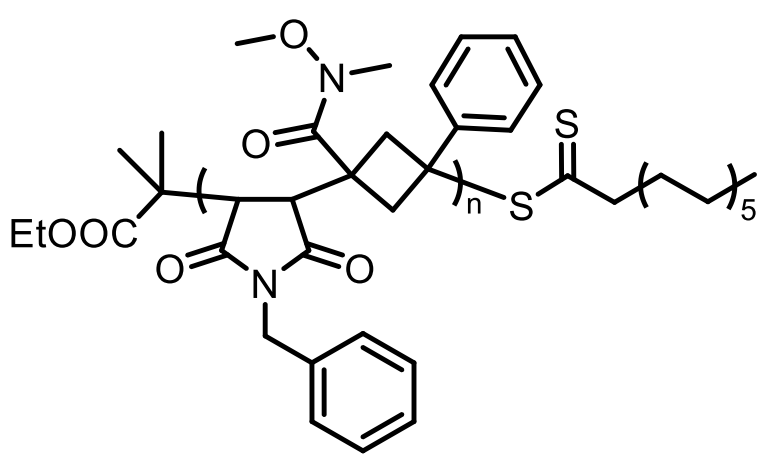

P7

Prepared by **GP-A**. White powder, 66.8 mg, 83% yield. ^{1}H NMR (400 MHz, CD_2Cl_2) δ 7.97 – 5.71 (m, 10H), 5.04 – 1.76 (m, 14H). SEC (THF): M_n = 10.4 kDa, $Đ$ = 1.19. TGA: T_d = 350 ºC. The T_g could not be detected.

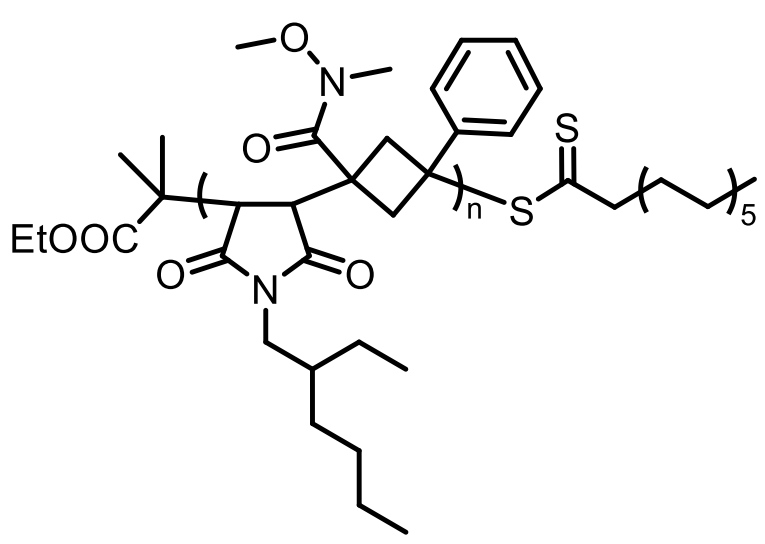

P8

Prepared by **GP-A**. White powder, 61 mg, 72% yield. ^{1}H NMR (400 MHz, CD_2Cl_2) δ 8.75 – 5.47 (m, 5H), 4.62 – 1.92 (m, 14H), 1.42 – 0.32 (m, 15H, the end-group signal peaks overlap). SEC (THF): M_n = 14.4 kDa, $Đ$ = 1.19. TGA: T_d = 328 ºC. The T_g could not be detected.

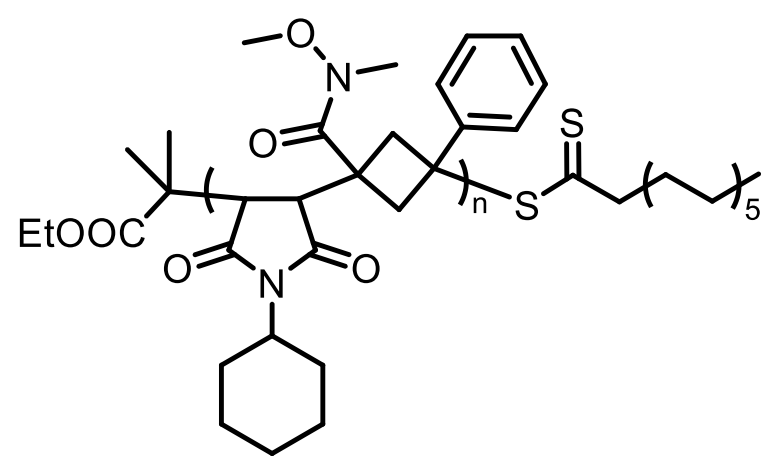

P9

Prepared by **GP-A**. White powder, 56.6 mg, 71% yield. ^{1}H NMR (400 MHz, CD_2Cl_2) δ 7.98 – 5.68 (m, 5H), 4.19 – 2.12 (m, 13H), 1.98 – 0.64 (m, 10H, the end-group signal peaks overlap). SEC (THF): M_n = 14.2 kDa, $Đ$ = 1.13. TGA: T_d = 344 ºC. The T_g could not be detected.

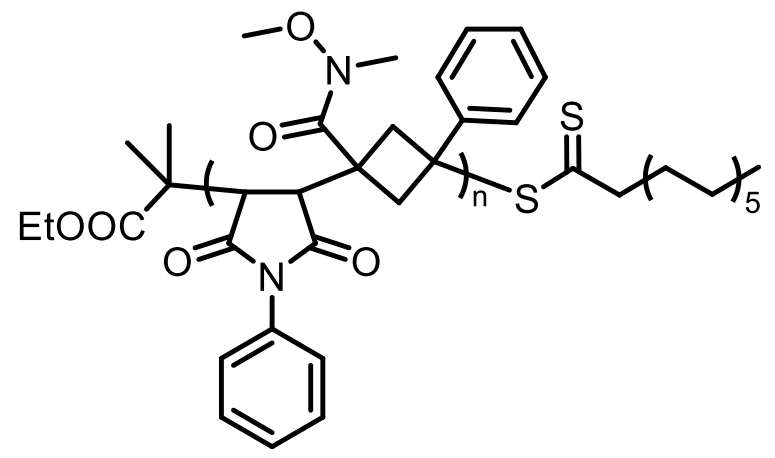

P10

Prepared by **GP-A**. White powder, 68.8 mg, 88% yield. ^{1}H NMR (400 MHz, CD_2Cl_2) δ 8.05 – 5.87 (m, 10H), 4.36 – 1.94 (m, 12H). SEC (THF): M_n = 9.5 kDa, $Đ$ = 1.22. TGA: T_d = 344 ºC. The T_g could not be detected.

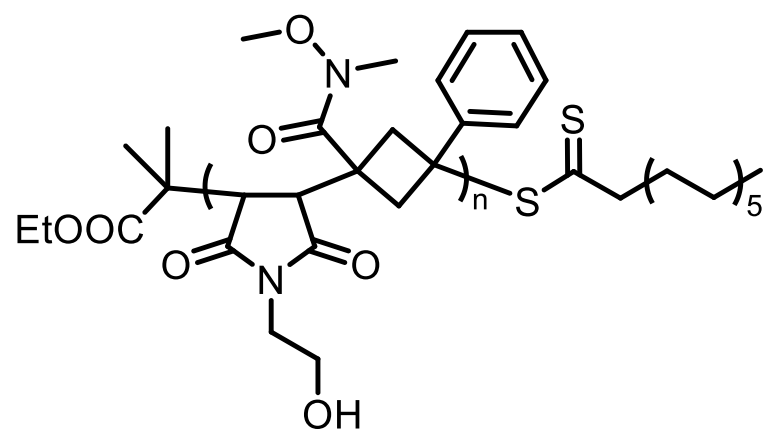

P11

Prepared by **GP-A**. White powder, 63.5 mg, 89% yield. ^{1}H NMR (400 MHz, CD_2Cl_2) δ 8.57 – 5.52 (m, 5H), 4.30 – 1.82 (m, 17H). SEC (DMA): M_n = 18.3 kDa, $Đ$ = 1.21. TGA: T_d = 310 ºC. The T_g could not be detected.

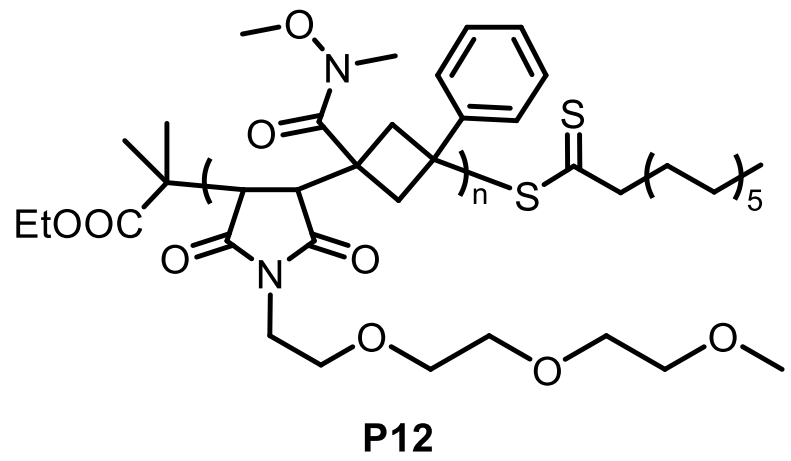

P12

Prepared by **GP-A**. White powder, 84.1 mg, 91% yield. ^{1}H NMR (400 MHz, CD_2Cl_2) δ 8.23 – 5.42 (m, 5H), 4.12 – 1.94 (m, 27H). SEC (THF): M_n = 11.8 kDa, $Đ$ = 1.26. TGA: T_d = 337 ºC. The T_g could not be detected.

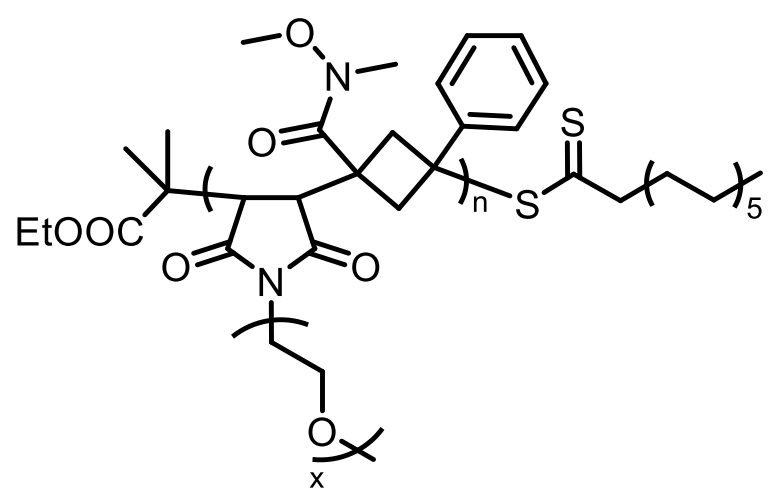

P13

Prepared by **GP-A**. Colorless viscous liquid, 115 mg, 81% yield. ^{1}H NMR (400 MHz, CD_2Cl_2) δ 8.20 – 5.50 (m, 5H), 4.46 – 1.86 (m, 52H). SEC (THF): M_n = 16.6 kDa, $Đ$ = 1.37. TGA: T_d = 306 ºC. The T_g could not be detected.

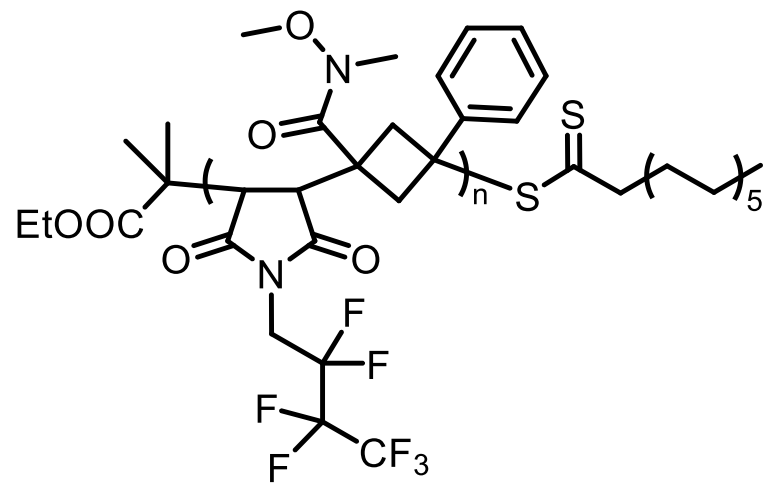

P14

Prepared by **GP-A**. White powder, 80.7 mg, 81% yield^{1}H NMR (400 MHz, CD_2Cl_2) δ 8.10 – 5.67 (m, 5H), 4.47 – 1.80 (m, 14H). ^{19}F NMR (377 MHz, CD_2Cl_2) δ -80.95, -114.76, -115.61, -117.28, -118.02, -128.04. SEC (THF): M_n = 14.0 kDa, $Đ$ = 1.16. TGA: T_d = 344 ºC. The T_g could not be detected.

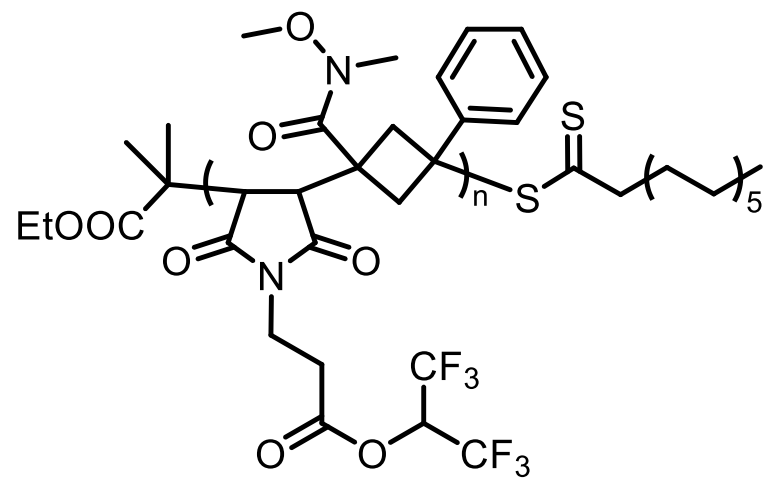

P15

Prepared by **GP-A**. White powder, 96.5 mg, 90% yield. ^{1}H NMR (400 MHz, CD_2Cl_2) δ 8.30 – 5.47 (m, 6H), 4.30 – 1.76 (m, 16H). ^{19}F NMR (377 MHz, CD_2Cl_2) δ -73.62. SEC (THF): M_n = 16.5 kDa, $Đ$ = 1.22. TGA: T_d = 340 ºC. The T_g could not be detected.

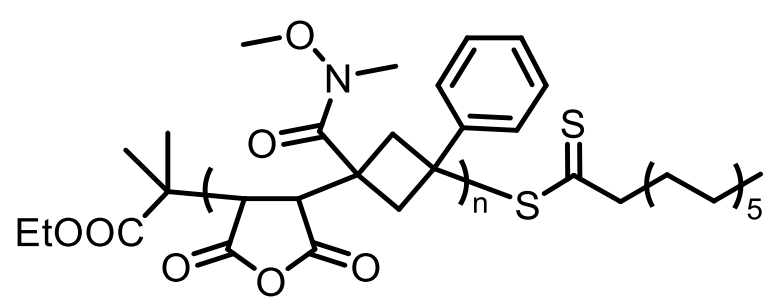

P16

Prepared by **GP-A**. White powder, 40.7 mg, 65% yield. ^{1}H NMR (400 MHz, CD_2Cl_2) δ 8.36 – 5.42 (m, 5H), 4.29 – 1.90 (m, 12H). SEC (THF): M_n = 8.0 kDa, $Đ$ = 1.26. TGA: T_d = 304 ºC. The T_g could not be detected.

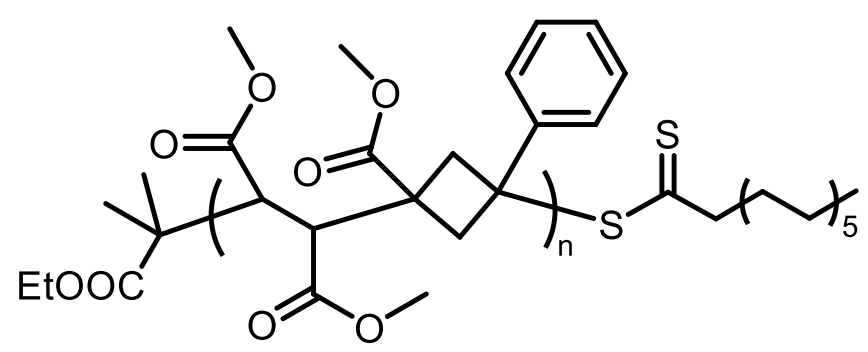

P17

Prepared by **GP-B**. White powder, 53.5 mg, 81% yield. ^{1}H NMR (400 MHz, CD_2Cl_2) δ 7.59 – 6.54 (m, 5H), 3.96 – 1.79 (m, 15H). SEC (THF): M_n = 8.5 kDa, $Đ$ = 1.17. TGA: T_d = 334 ºC. The T_g could not be detected.

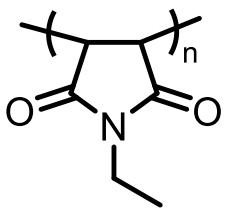

P(EtMI)

Prepared by **GP-C** (The polymer was obtained from the reaction between **BCB3** and **EtMI)**. White solid, 11.5 mg, 46% yield. ^{1}H NMR (400 MHz, $CDCl_3$) δ 4.14 – 2.94 (m, 4H), 1.51 – 0.60 (m, 3H). SEC (THF): M_n = 9.6 kDa, $Đ$ = 1.92. TGA: T_d = 353 ºC.

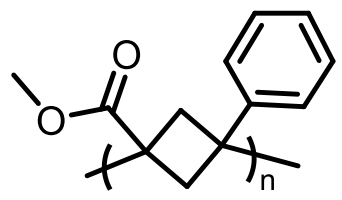

P(BCB1)

Prepared by **GP-C** (The reaction time extended to 24 h). White powder, 4.0 mg, 11% yield. ^{1}H NMR (400 MHz, CD_2Cl_2) δ 7.77 – 5.79 (m, 5H), 3.99 – 1.62 (m, 7H). SEC (THF): M_n = 9.0 kDa, $Đ$ =1.58. TGA: T_d = 362 ºC.

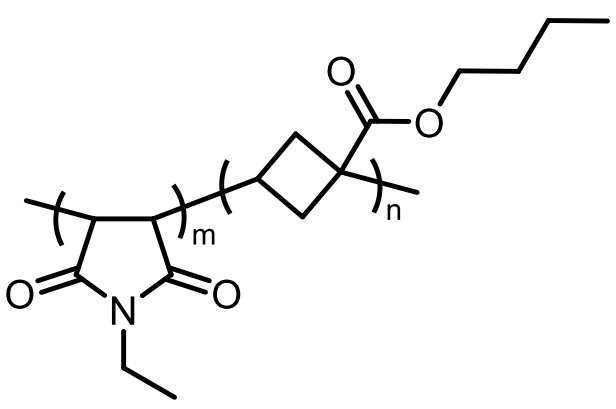

P(BCB2-*r*-EtMI)

Prepared by **GP-C**. Colorless transparent solid, 44.8 mg, 80% yield. ^{1}H NMR (400 MHz, CD_2Cl_2) δ 4.30 – 3.88 (m, 2H), 3.81 – 1.74 (m, 9.4H), 1.67 – 1.49 (m, 2H), 1.44 – 1.26 (m, 2H), 1.24 – 1.00 (m, 3.3H), 0.99 – 0.82 (m, 3H). SEC (THF): M_n = 85.4 kDa, $Đ$ = 2.07.

4. Experimental Procedures and Characterization Data of BCBs

General procedure D:

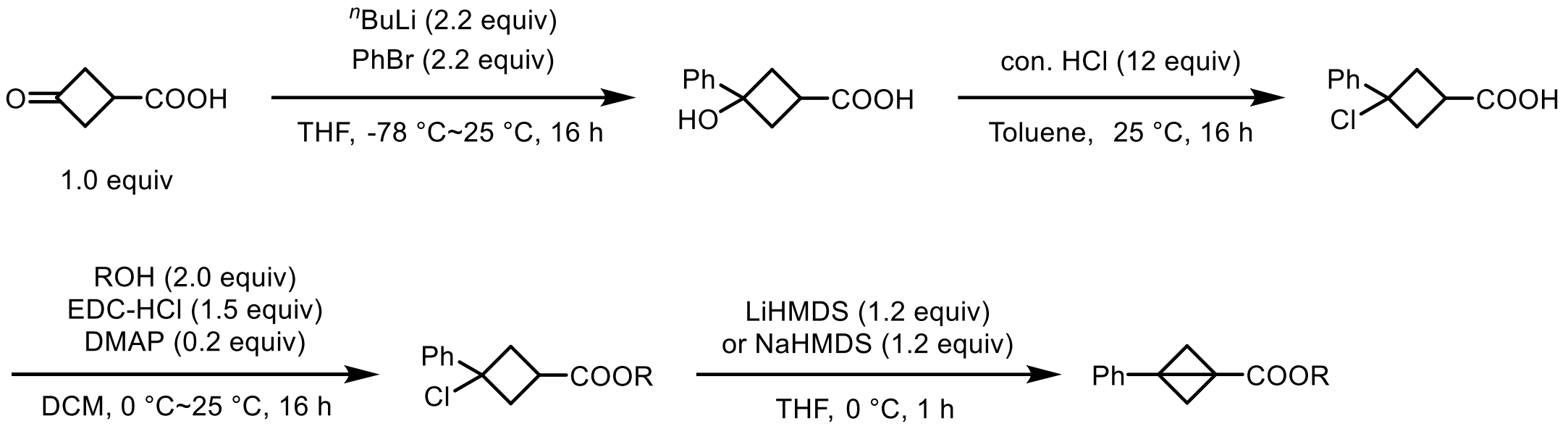

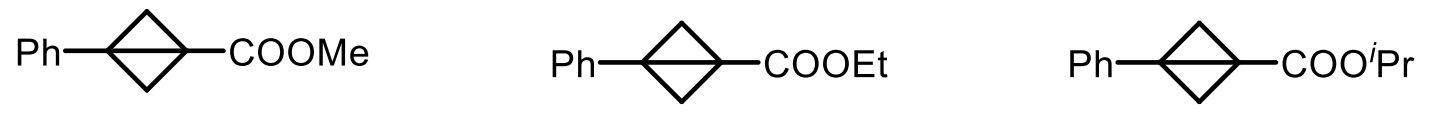

S1, BCB1 **S4, BCB4** **S5, BCB5**

Scheme S6. The synthesis of phenyl and ester-disubstituted BCBs.

Step 1: To an oven-dried flask was added phenyl bromide (2.2 equiv) and anhydrous THF (0.7 M) under N_2. The reaction was cooled to -78 °C and ^{n}BuLi (1.6 M, 2.2 equiv) was added dropwise. The reaction was stirred for 1 hour at -78 °C before a solution of 3-oxocyclobutane-1-carboxylic acid (1.0 equiv) in THF (2.0 M) was added. The mixture was allowed to slowly warm to room temperature and stirred at 25 °C for 16 hours. Upon completion, the reaction was quenched with saturated NH_4Cl before being basified with 4 M NaOH solution and washed with EtOAc twice. The aqueous layer was then acidified with 2 M HCl solution and extracted with EtOAc three times. The combined organic layers were washed with saturated brine and dried over $MgSO_4$, filtered, and concentrated under reduced pressure to afford 3-hydroxy-3-phenylcyclobutane-1-carboxylic acid as a white solid. This material was used directly in the next step without further purification.

Step 2: To an oven-dried flask was added phenylcyclobutane-1-carboxylic acid (1.0 equiv) and toluene (0.8 M), concentrated HCl (12 equiv) was added at 25 °C. The resulting mixture was stirred at 25 °C for 16 hours. The organic layer was separated, washed with H_2O and brine, dried over $MgSO_4$, filtered, and concentrated under reduced pressure to afford 3-chloro-3-phenylcyclobutane-1-carboxylic acid as a white solid. This material was used directly in the next step without further purification.

Step 3: To an oven-dried flask was added 3-chloro-3-phenylcyclobutane-1-carboxylic acid (1.0 equiv) and anhydrous DCM (0.2 M) under N_2. The reaction mixture was cooled to 0 °C, followed by the addition of 1-ethyl-3-(3-dimethylaminopropyl)carbodiimide hydrochloride (EDC-HCl, 1.5 equiv) and ROH (1.0 equiv). DMAP (0.2 equiv) was then added, and the reaction mixture was stirred at 25 °C for 16 h. Upon completion, the reaction was quenched with saturated NH_4Cl, and the aqueous layer was extracted with DCM three times. The combined organic layers were dried over $MgSO_4$, filtered, and concentrated under reduced pressure. The crude residue was purified by flash column chromatography on silica gel to afford the corresponding 3-chloro-3-phenylcyclobutane-1-carboxylate.

Step 4: To an oven-dried flask was added 3-chloro-3-phenylcyclobutane-1-carboxylate (1.0 equiv) and anhydrous THF (0.4 M) under N_2. The reaction was cooled to 0 °C and LiHMDS (for **S1** and **S4**, 1 M,1.2 equiv) or NaHMDS (for **S5**, 1 M,1.2 equiv) was added dropwise. The resulting mixture

was stirred for 1 hour at 0 °C. The reaction was then quenched with saturated NH_4Cl and extracted with EtOAc three times. The combined organic layers were washed brine, dried over Mg_2SO_4, filtered, and concentrated under reduced pressure. The crude reaction mixture was purified by flash column chromatography on silica gel to afford the corresponding ester-substituted BCBs.

BCB1 (S1)[1], **BCB4 (S4)**[1], and **BCB5 (S5)**[1] were prepared according to general procedure D and analytical data are in agreement with previously reported values.

Note: Disubstituted BCB monomers remained stable after storage in a fridge at 5 °C for more than six months.

The synthesis of BCB6:

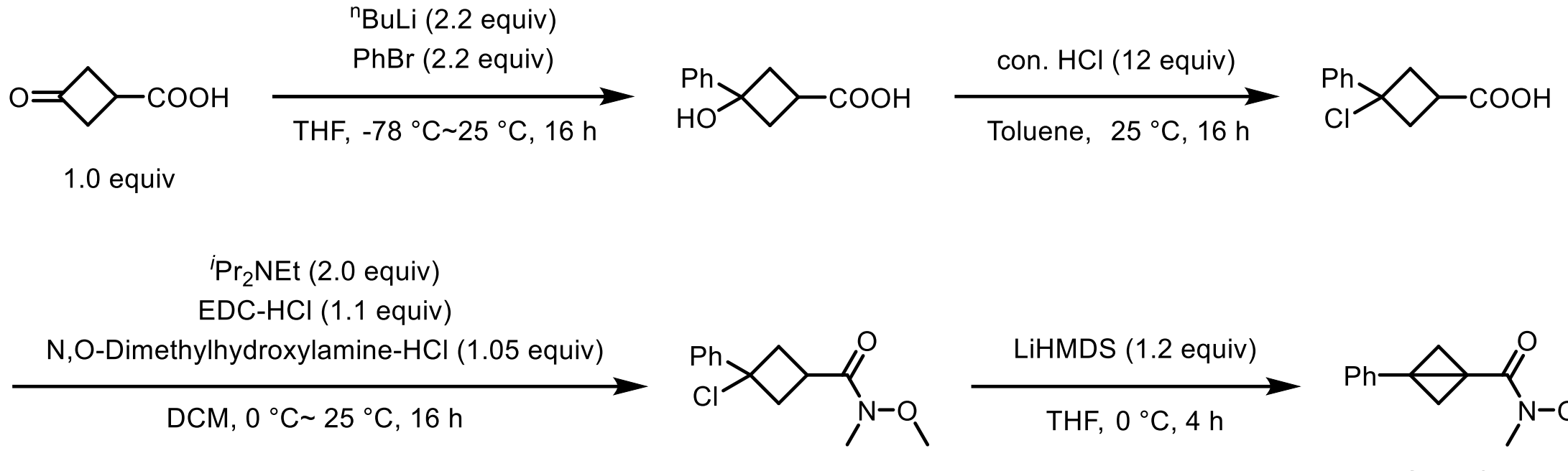

S6, BCB6

Scheme S7. The synthesis of **BCB6**.

Steps 1 and 2 were performed according to steps 1 and 2 of General Procedure A.

Step 3: To an oven-dried flask was added 3-chloro-3-phenylcyclobutane-1-carboxylic acid (1.0 equiv) and anhydrous DCM (0.2 M) under N_2. *N*, O-dimethylhydroxylamine hydrochloride (1.05 equiv) was added at 0 °C, followed by the slow addition of *N*, *N*-Diisopropylethylamine (2.0 equiv). After stirring at 0 °C for 5 minutes, the reaction was allowed to warm to room temperature and stir at 25 °C for 16 hours. Upon completion, the solution was diluted with H_2O and extracted with DCM three times. The combined organic layers were washed with brine, dried over $MgSO_4$, filtered, and concentrated under reduced pressure. The crude residue was purified by flash column chromatography on silica gel to give the corresponding 3-chloro-*N*-methoxy-*N*-methyl-3-phenylcyclobutane-1-carboxamide.

Step 4: To an oven-dried flask was added 3-chloro-*N*-methoxy-*N*-methyl-3-phenylcyclobutane-1-carboxamide (1.0 equiv) and anhydrous THF (0.4 M) under N_2. The reaction was cooled to 0 °C and LiHMDS (1 M,1.2 equiv) was added dropwise. The resulting mixture was stirred at 0 °C for 4 hours. The reaction was then quenched with saturated NH_4Cl and extracted with EtOAc three times. The combined organic layers were washed brine, dried over Mg_2SO_4, filtered, and concentrated under reduced pressure. The crude reaction mixture was purified by flash column chromatography on silica gel to afford **BCB6**. The analytical data of **BCB6 (S6)**[2] is in agreement with previously reported values.

The synthesis of BCB7:

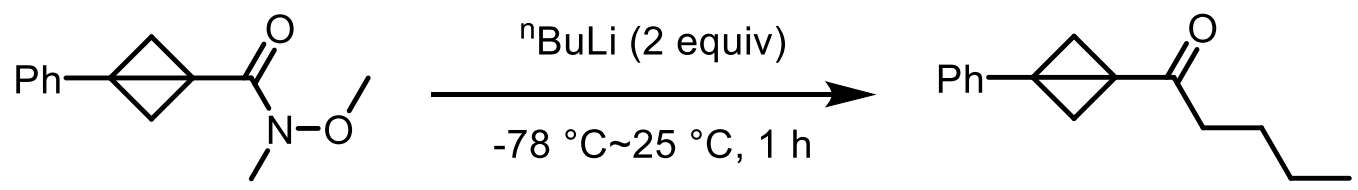

S7, BCB7

Scheme S8. The synthesis of **BCB7**.

To an oven-dried Schlenk tube was added **BCB6** (1.0 equiv) and anhydrous THF (0.5 M). The solution was cooled to -78 °C and ^{n}BuLi (1.6 M, 2.0 equiv) was added dropwise. The resulting mixture was allowed to warm to room temperature and stirred for 1 h. The reaction was quenched with saturated NH_4Cl and extracted with EtOAc three times. The combined organic layers dried over Mg_2SO_4, filtered, and concentrated under reduced pressure. The crude reaction mixture was purified by flash column chromatography on silica gel to afford **BCB7**. The analytical data of **BCB7 (S7)** [3] is in agreement with previously reported values.

The synthesis of BCB2:

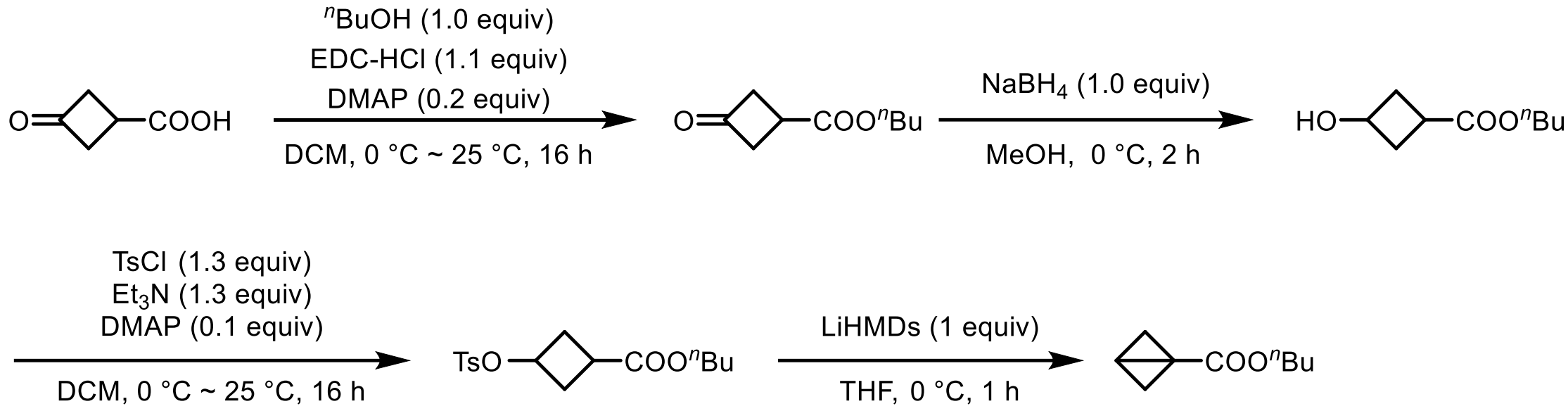

S2, BCB2

Scheme S9. The synthesis of **BCB2**.

Step 1: To an oven-dried flask was added 3-oxocyclobutane-1-carboxylic acid (1.0 equiv) and anhydrous DCM (0.2 M) under N_2. The reaction mixture was cooled to 0 °C, followed by the addition of 1-ethyl-3-(3-dimethylaminopropyl)carbodiimide hydrochloride (EDC-HCl, 1.5 equiv) and ^{n}BuOH (1.0 equiv). DMAP (0.2 equiv) was then added, and the reaction mixture was stirred at 25 °C for 16 hours. Upon completion, the reaction was quenched with saturated NH_4Cl, and the aqueous layer was extracted with DCM three times. The combined organic layers were dried over $MgSO_4$, filtered, and concentrated under reduced pressure. The crude residue was purified by flash column chromatography on silica gel to afford butyl 3-oxocyclobutane-1-carboxylate.

Step 2: To a stirred solution of butyl 3-oxocyclobutane-1-carboxylate (1.0 equiv) in MeOH (0.5 M) at 0 °C was added $NaBH_4$ (0.5 equiv) in portion. After stirred at 0 °C for 30 min, the reaction was quenched with saturated NH_4Cl and concentrated to remove MeOH. The resulting aqueous phase was extracted with DCM three times. The combined organic layer was washed with brine, dried over $MgSO_4$, filtered, concentrated under reduced pressure to afford butyl 3-hydroxycyclobutane-1-carboxylate. This material was used directly in the next step without further purification.

Step 3: To an oven-dried flask was added butyl 3-hydroxycyclobutane-1-carboxylate (1.0 equiv) and anhydrous DCM (0.5 M). The reaction mixture was cooled to 0 °C, followed by the addition of Et_3N (1.3 equiv), DMAP (0.1 equiv) and TsCl (1.3 equiv). The mixture was stirred at 25 °C for 16 h before being diluted with H_2O and DCM. The separated aqueous phase was extracted with DCM three times. The combined organic layers were washed with brine, dried over $MgSO_4$, filtered, and concentrated under reduced pressure. The crude residue was purified by flash column chromatography on silica gel to afford butyl 3-(tosyloxy)cyclobutane-1-carboxylate.

Step 4: To an oven-dried flask was added butyl 3-(tosyloxy)cyclobutane-1-carboxylate (1.0 equiv) and anhydrous THF (0.2 M) under N_2. The reaction was cooled to 0 °C and LiHMDS (1 M, 1.0 equiv) was added dropwise. The resulting mixture was stirred for 1 hour at 0 °C. The reaction was then quenched with saturated NH_4Cl and extracted with EtOAc three times. The combined organic layers were washed brine, dried over Mg_2SO_4, filtered, and concentrated under reduced pressure.

The crude reaction mixture was purified by flash column chromatography on silica gel to afford **BCB2**. The analytical data of **BCB2 (S2)** [4] is in agreement with previously reported values.

The synthesis of BCB3:

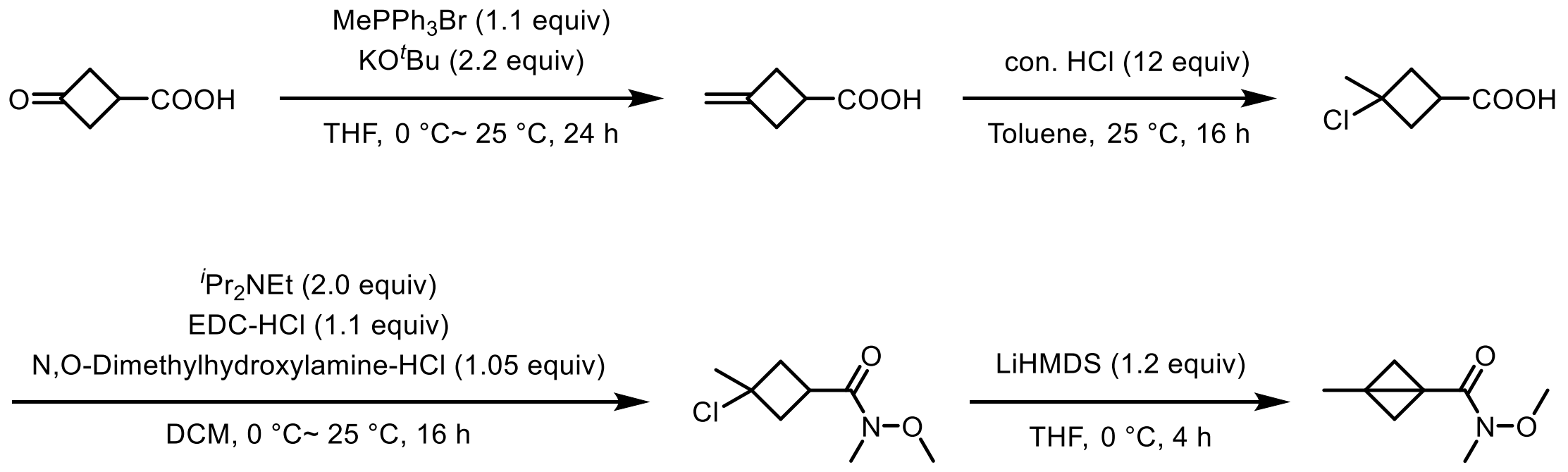

S3, BCB3

Scheme S10. The synthesis of **BCB3**.

Step 1: To an oven-dried flask was added $MePPh_3I$ (1.1 equiv) and ^{t}BuOK (2.2 equiv) under N_2. Anhydrous THF (0.2 M) was added at 0 °C, and the resulting mixture was stirred at 0 °C for 2 h. 3-Oxocyclobutane-1-carboxylic acid (1.0 equiv) in THF (2.0 M) was then added, and the reaction mixture was allowed to warm to room temperature and stirred at 25 °C for 16 h. Upon completion, the reaction was quenched with water and diluted with Et_2O. The resulting mixture was filtered through Celite, and the organic layer was separated. The aqueous layer was extracted with Et_2O twice, and the combined organic layers were washed with brine, dried over $MgSO_4$, filtered, and concentrated under reduced pressure to afford 3-methylenecyclobutane-1-carboxylic acid. This material was used directly in the next step without further purification.

Step 2: To an oven-dried flask was added 3-methylenecyclobutane-1-carboxylic acid (1.0 equiv) and toluene (0.5 M), concentrated HCl (12 equiv) was added at 25 °C. The resulting mixture was stirred at 25 °C for 16 hours. The organic layer was separated, and the aqueous layer was extracted with Et_2O twice. The combined organic layers were washed with H_2O and brine, dried over $MgSO_4$, filtered, and concentrated under reduced pressure to afford 3-chloro-3-methylcyclobutane-1-carboxylic acid. This material was used directly in the next step without further purification.

Step 3: To an oven-dried flask was added -chloro-3-methylcyclobutane-1-carboxylic acid (1.0 equiv) and anhydrous DCM (0.2 M) under N_2. *N*, O-dimethylhydroxylamine hydrochloride (1.05 equiv) was added at 0 °C, followed by the slow addition of *N*, *N*-Diisopropylethylamine (2.0 equiv). After stirring for 5 minutes at 0 °C, the reaction was allowed to warm to room temperature and stir at 25 °C for 16 hours. Upon completion, the solution was diluted with H_2O and extracted with DCM three times. The combined organic layers were washed with brine, dried over $MgSO_4$, filtered, and concentrated under reduced pressure. The crude residue was purified by flash column chromatography on silica gel to give the corresponding 3-chloro-*N*-methoxy-N,3-dimethylcyclobutane-1-carboxamide.

Step 4: To an oven-dried flask was added 3-chloro-N-methoxy-N,3-dimethylcyclobutane-1-carboxamide (1.0 equiv) and anhydrous THF (0.4 M) under N_2. The reaction was cooled to 0 °C and LiHMDS (1 M,1.2 equiv) was added dropwise. The resulting mixture was stirred for 4 hours at 0 °C. The reaction was then quenched with saturated NH_4Cl and extracted with EtOAc three times. The combined organic layers were washed brine, dried over Mg_2SO_4, filtered, and concentrated under reduced pressure. The crude reaction mixture was purified by flash column chromatography

on silica gel to afford **BCB3**. The analytical data of **BCB3 (S3)**[5] is in agreement with previously reported values.

5. Experimental Procedures and Characterization Data of Maleimide Derivatives

General procedure E:

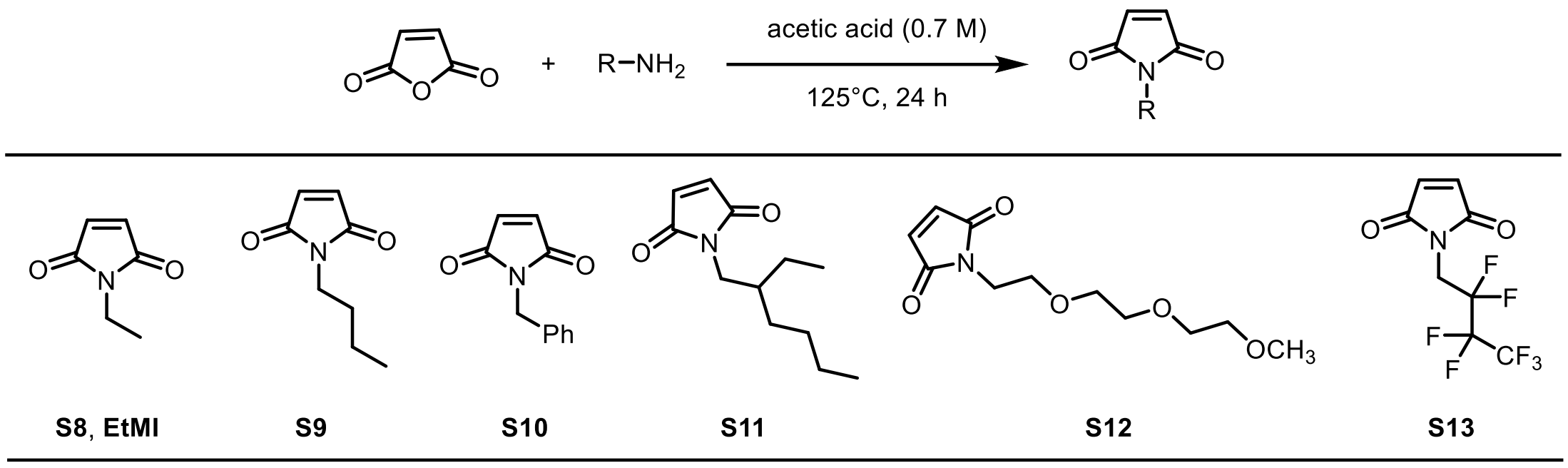

Scheme S11. General procedure A for the synthesis of substituted maleimides.

Add the amine (1 equiv) to a stirred solution of maleic anhydride (2.5 equiv) in acetic acid (0.7 M) in an oven-dried sealed pressure tube. The reaction was stirred at 125 °C for 24 hours. Upon completion, the acetic acid was removed under reduced pressure, and the residue was dissolved in EtOAc. The organic layer was washed successively with aqueous $NaHCO_3$ and brine, dried over $MgSO_4$, filtered, and concentrated under reduced pressure. The crude product was purified by flash column chromatography on silica gel to afford the corresponding substituted maleimides. **S8** [6], **S9, S10, S11, S12, S13** were prepared according to general procedure E.

The synthesis of S14:

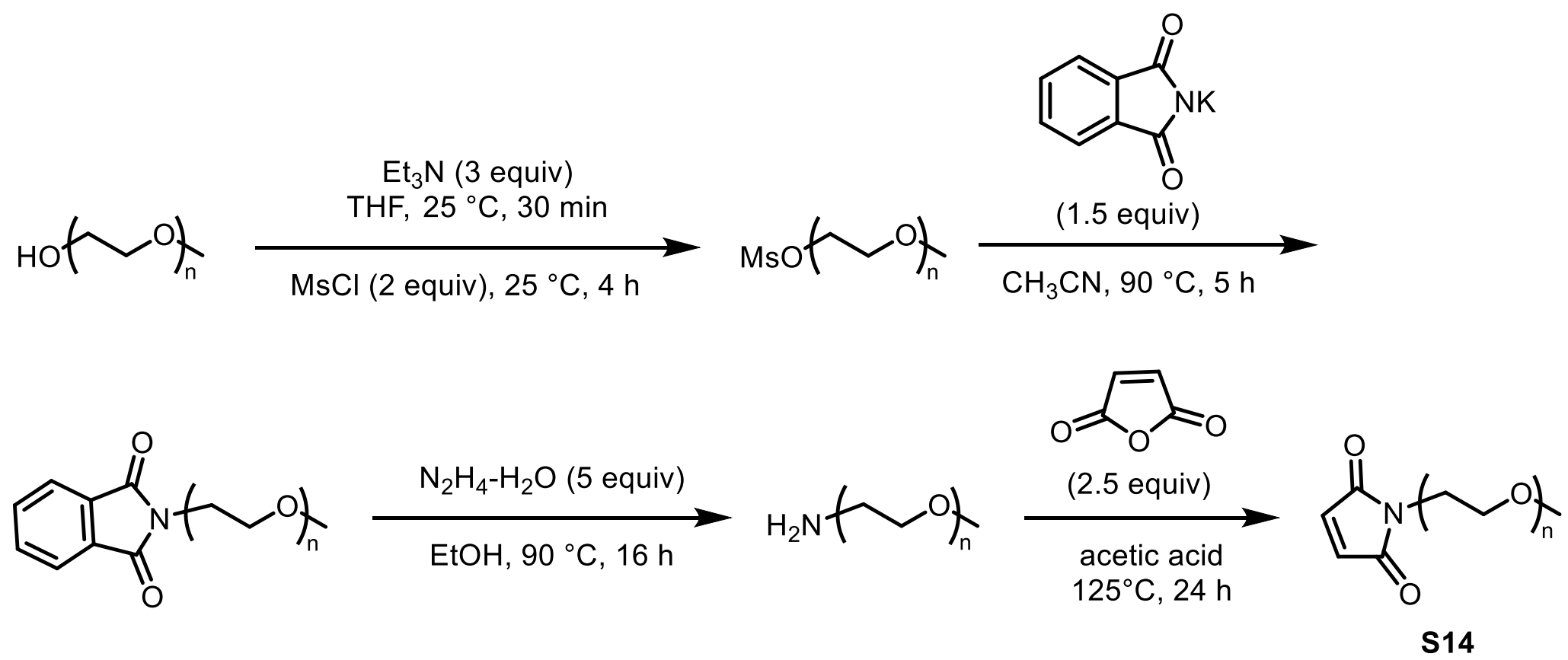

Scheme S12. The synthesis of **S14**.

Step 1: Add triethylamine (3.0 equiv) to a stirred solution of polyethylene glycol 400 (1.0 equiv) in anhydrous THF (0.2 M) under N_2. The reaction mixture was cooled to 0 °C and stirred for 30 min before methanesulfonyl chloride (2.0 equiv) in anhydrous THF (2.0 M) was added dropwise. After stirring at 0 °C for an additional 30 min, the reaction mixture was allowed to warm to room temperature and stirred at 25 °C for 4 h. Upon completion, the reaction mixture was washed successively with saturated aqueous $NaHCO_3$ solution (2 ×), H_2O (2 ×), and brine (1 ×). The organic layer was dried over $MgSO_4$, filtered, and concentrated under reduced pressure to afford the corresponding mesylate. The crude product was used directly in the next step without further purification.

Step 2: Add potassium phthalimide (1.5 equiv) to a stirred solution of the mesylate (1.0 equiv) obtained from the first step in CH_3CN (0.25 M). The reaction mixture was heated to reflux and stirred for 5 h. Upon completion, the reaction mixture was cooled to room temperature and filtered to remove excess potassium phthalimide. The filtrate was concentrated under reduced pressure to remove CH_3CN. Water was then added, and the aqueous layer was extracted with DCM (3 ×). The combined organic layers were washed with brine, dried over $MgSO_4$, filtered, and concentrated under reduced pressure to afford the corresponding PEG_{400}-derived phthalimide. The crude product was used directly in the next step without further purification.
Step 3: To an oven-dried flask was added the PEG_{400}-derived phthalimide, EtOH (0.1 M), and hydrazine hydrate (5.0 equiv). The reaction mixture was stirred at 90 °C for 16 h. Upon completion, the reaction mixture was cooled to room temperature and filtered. The filtrate was concentrated under reduced pressure to remove EtOH. The resulting residue was diluted with DCM and washed with 10% aqueous KOH solution. The aqueous layers were combined and extracted with DCM. The combined organic extracts were washed with brine, dried over $MgSO_4$, filtered, and concentrated under reduced pressure to afford the corresponding PEG_{400}-derived amine, which was used in the next step without further purification.
Step 4: Add the PEG_{400}-derived amine (1 equiv) to a stirred solution of maleic anhydride (2.5 equiv) in acetic acid (0.7 M) in an oven-dried sealed pressure tube. The reaction was stirred at 125 °C for 24 hours. Upon completion, the acetic acid was removed under reduced pressure, and the residue was dissolved in EtOAc. The organic layer was washed successively with aqueous $NaHCO_3$ (2 ×) and brine (2 ×), dried over $MgSO_4$, filtered, and concentrated under reduced pressure. The crude product was purified by flash column chromatography on silica gel to afford the corresponding PEG_{400}-derived maleimides **S14**.

The synthesis of S15:

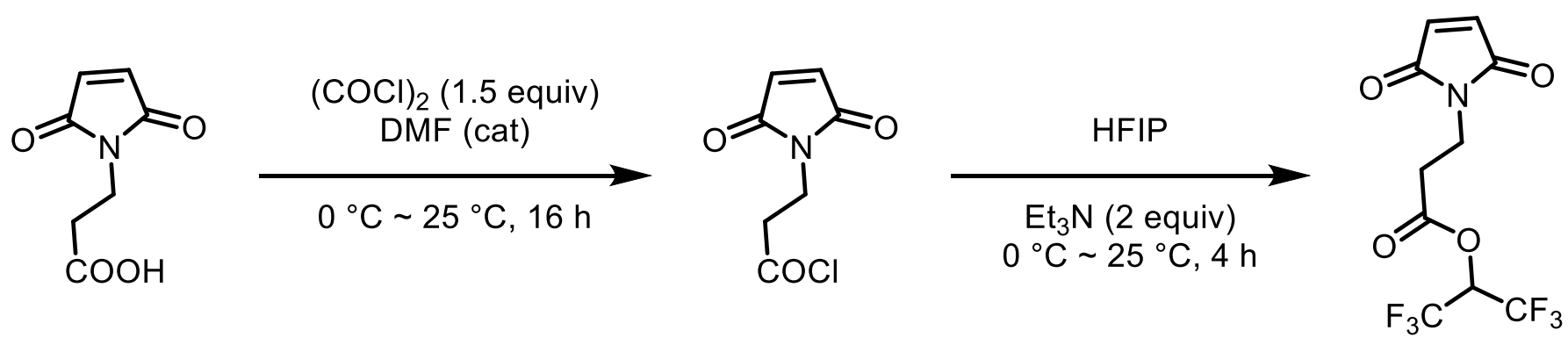

Scheme S13. The synthesis of **S15**.
Step 1: To an oven-dried flask was added 3-maleimidopropionic acid (1.0 equiv), anhydrous DCM (0.2 M), and a catalytic amount of DMF. The reaction mixture was cooled to 0 °C and stirred for 10 min. Oxalyl chloride ($(COCl)_2$, 1.5 equiv) was added dropwise, and the resulting mixture was allowed to warm to room temperature and stirred at 25 °C for 16 h. The reaction mixture was concentrated under reduced pressure to afford the corresponding acyl chloride, which was used directly in the next step without further purification.
Step 2: To an oven-dried flask was added hexafluoroisopropanol (HFIP, 1.0 equiv) and anhydrous DCM (0.5 M). The reaction mixture was cooled to 0 °C, followed by the addition of Et_3N (2.0 equiv), and stirred at 0 °C for 15 minutes. A solution of the acyl chloride (1.2 equiv) in anhydrous DCM (0.5 M) was then added dropwise. The reaction mixture was allowed to warm to room temperature and stirred at 25 °C for 4 h. The reaction was quenched with water and extracted with DCM three times. The combined organic layers were washed with brine, dried over $MgSO_4$, filtered, and

concentrated under reduced pressure. The crude product was purified by flash column chromatography on silica gel to afford **S15**.

1-butyl-1H-pyrrole-2,5-dione (**S9**)

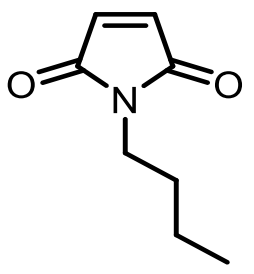

Colorless oil. ^{1}H NMR (400 MHz, $CDCl_3$) δ 6.65 (s, 2H), 3.48 (t, *J* = 7.3 Hz, 2H), 1.60 – 1.45 (m, 2H), 1.35 – 1.19 (m, 2H), 0.88 (t, *J* = 7.4 Hz, 3H); ^{13}C NMR (100 MHz, $CDCl_3$) δ 170.8, 134.0, 37.6, 30.5, 19.8, 13.5; HR-MS (ESI) m/z calcd for $C_8H_{12}NO_2$ [M+H$^+$] 154.08626, found 154.0862.

1-benzyl-1H-pyrrole-2,5-dione (**S10**)

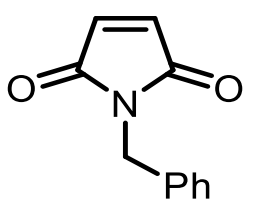

White solid. ^{1}H NMR (400 MHz, $CDCl_3$) δ 7.42 – 7.22 (m, 5H), 6.70 (s, 2H), 4.67 (s, 2H); ^{13}C NMR (100 MHz, $CDCl_3$) δ 170.4, 136.2, 134.2, 128.6, 128.3, 127.8, 41.4; HR-MS (ESI) m/z calcd for $C_{11}H_9NNaO_2$ [M+H$^+$] 210.0526, found 210.0525.

1-(2-ethylhexyl)-1H-pyrrole-2,5-dione (**S11**)

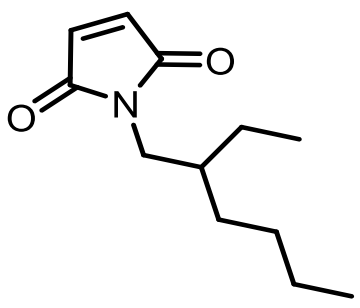

Colorless oil. ^{1}H NMR (400 MHz, $CDCl_3$) δ 6.66 (s, 2H), 3.38 (d, *J* = 7.3 Hz, 2H), 1.77 – 1.62 (m, 1H), 1.35 – 1.12 (m, 8H), 0.94 – 0.79 (m, 6H); ^{13}C NMR (100 MHz, $CDCl_3$) δ 171.1, 133.9, 41.7, 38.2, 30.4, 28.4, 23.7, 22.9, 14.0, 10.3; HR-MS (ESI) m/z calcd for $C_{12}H_{20}NO_2$ [M+H$^+$] 210.1489, found 210.1488.

1-(2-(2-(2-methoxyethoxy)ethoxy)ethyl)-1H-pyrrole-2,5-dione (**S12**)

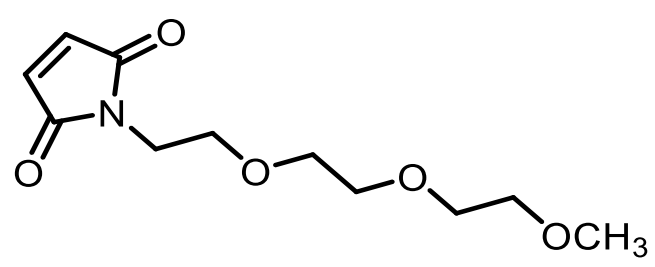

Colorless oil. ^{1}H NMR (400 MHz, $CDCl_3$) δ 6.65 (s, 2H), 3.69 – 3.63 (m, 2H), 3.61 – 3.51 (m, 8H), 3.49 – 3.43 (m, 2H), 3.30 (s, 3H); ^{13}C NMR (100 MHz, $CDCl_3$) δ 170.5, 134.0, 71.7, 70.4, 70.4, 69.9, 67.6, 58.8, 37.0; HR-MS (ESI) m/z calcd for $C_{11}H_{17}NNaO_5$ [M+H$^+$] 266.0999, found 266.0995.

1-(2,2,3,3,4,4,4-heptafluorobutyl)-1H-pyrrole-2,5-dione (**S13**)

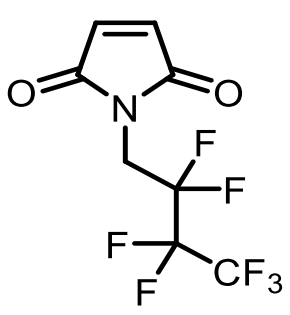

Colorless oil. ^{1}H NMR (400 MHz, $CDCl_3$) δ 6.84 (s, 2H), 4.18 (t, *J* = 15.3 Hz, 2H); ^{13}C NMR (100 MHz, $CDCl_3$) δ 168.9, 134.7, 122.1 – 105.3 (m, 3C), 36.9 (td, *J* = 23.4, 1.4 Hz); ^{19}F NMR (377 MHz, $CDCl_3$) δ -80.68 (t, *J* = 9.6 Hz), -117.21 – -117.41 (m, 2F), -127.73 (dd, *J* = 21.5, 4.9 Hz); HR-MS (ESI) m/z calcd for $C_8H_5F_7NO_2$ [M+H$^+$] 280.0203, found 280.0202.

1,1,1,3,3,3-hexafluoropropan-2-yl 3-(2,5-dioxo-2,5-dihydro-1H-pyrrol-1-yl)propanoate (**S13**)

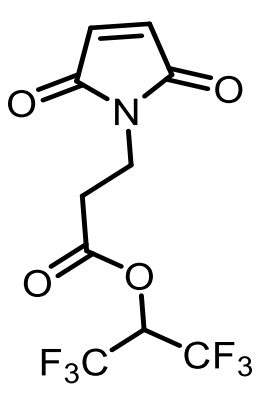

White solid. ^{1}H NMR (400 MHz, $CDCl_3$) δ 6.72 (s, 2H), 5.74 (hept, *J* = 6.1 Hz, 1H), 3.88 (t, *J* = 7.1 Hz, 2H), 2.89 (t, *J* = 7.1 Hz, 2H). ^{13}C NMR (100 MHz, $CDCl_3$) δ 170.0, 167.6, 134.3, 124.4 – 116.0 (m), 66.7 (q, *J* = 34.7 Hz), 32.9, 31.8; ^{19}F NMR (377 MHz, $CDCl_3$) δ -73.28, -73.30; HR-MS (ESI) m/z calcd for $C_{10}H_7F_6NNaO_4$ [M+H$^+$] 342.0712, found 342.0171.

***N*-(methoxytriethylene glycol) maleimide** (**S12**)

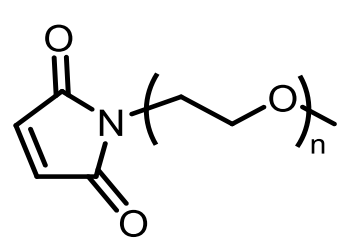

Colorless oil. ^{1}H NMR (400 MHz, $CDCl_3$) δ 6.63 (s, 2H), 3.68 – 3.42 (m, 37H), 3.28 (s, 3H); ^{13}C NMR (100 MHz, $CDCl_3$) δ 170.4, 133.9, 71.7, 70.34, 70.30, 70.27, 70.2, 69.8, 67.5, 58.7, 36.9.

6. Photoiniferter-mediated Homopolymerization of BCB1 and EtMI

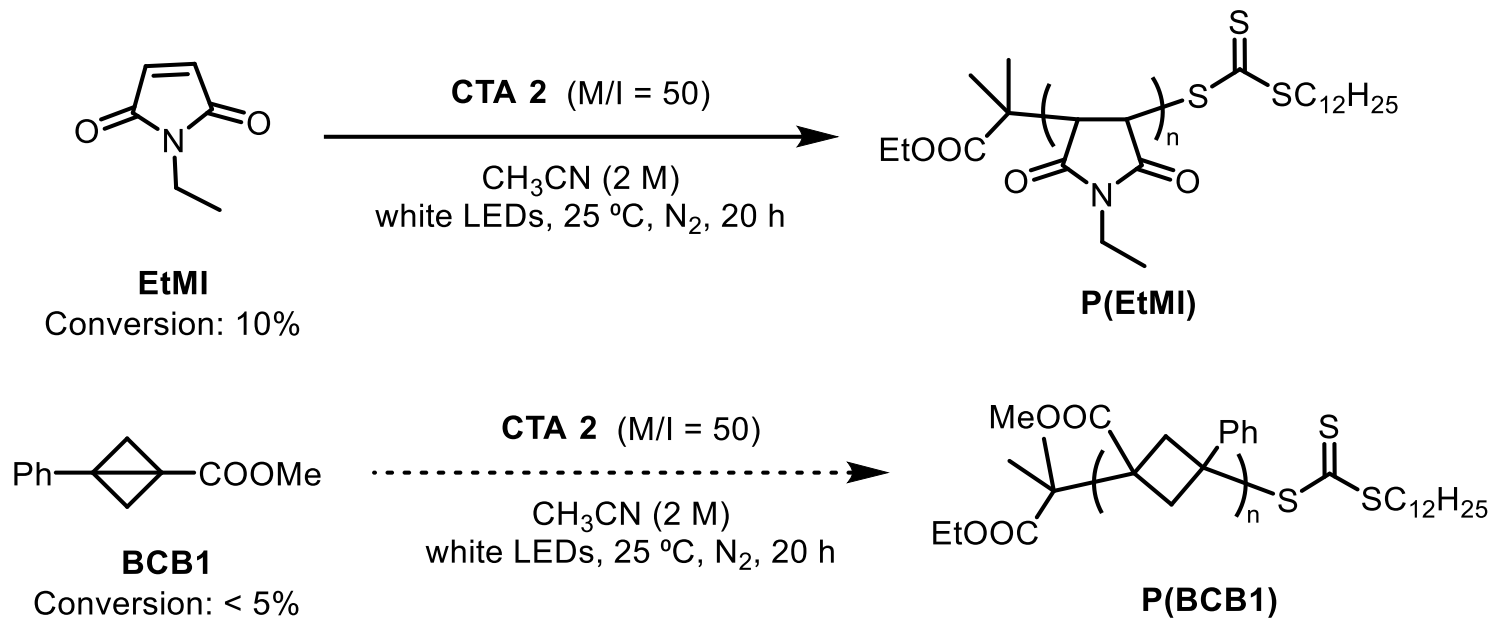

Scheme S14. Photoiniferter-mediated homopolymerization of **BCB1** and **EtMI.**

In a nitrogen-filled glovebox, a 5 mL reaction vial equipped with a magnetic stirring bar was charged with **BCB1** (0.2 mmol) or **EtMI** (0.2 mmol), followed by addition of **CTA2** (2 mol%, 0.004 mmol, 100 μL of a 0.04 M solution in acetonitrile). The vial was sealed, removed from the glovebox, and stirred under white light irradiation at 25 °C for 20 h. Upon completion, the resulting mixture was concentration under reduced pressure, the mixture was analyzed by ^{1}H NMR. The NMR sample was then recovered and combined with the remaining crude mixture, and naphthalene was added as an internal standard. Monomer conversion was determined by gas chromatography.

Note: Under otherwise standard reaction conditions, the conversion of **BCB1** remained below 5%, and the unreacted monomer could be recovered by flash column chromatography, demonstrating that **BCB1** is stable under the polymerization conditions.

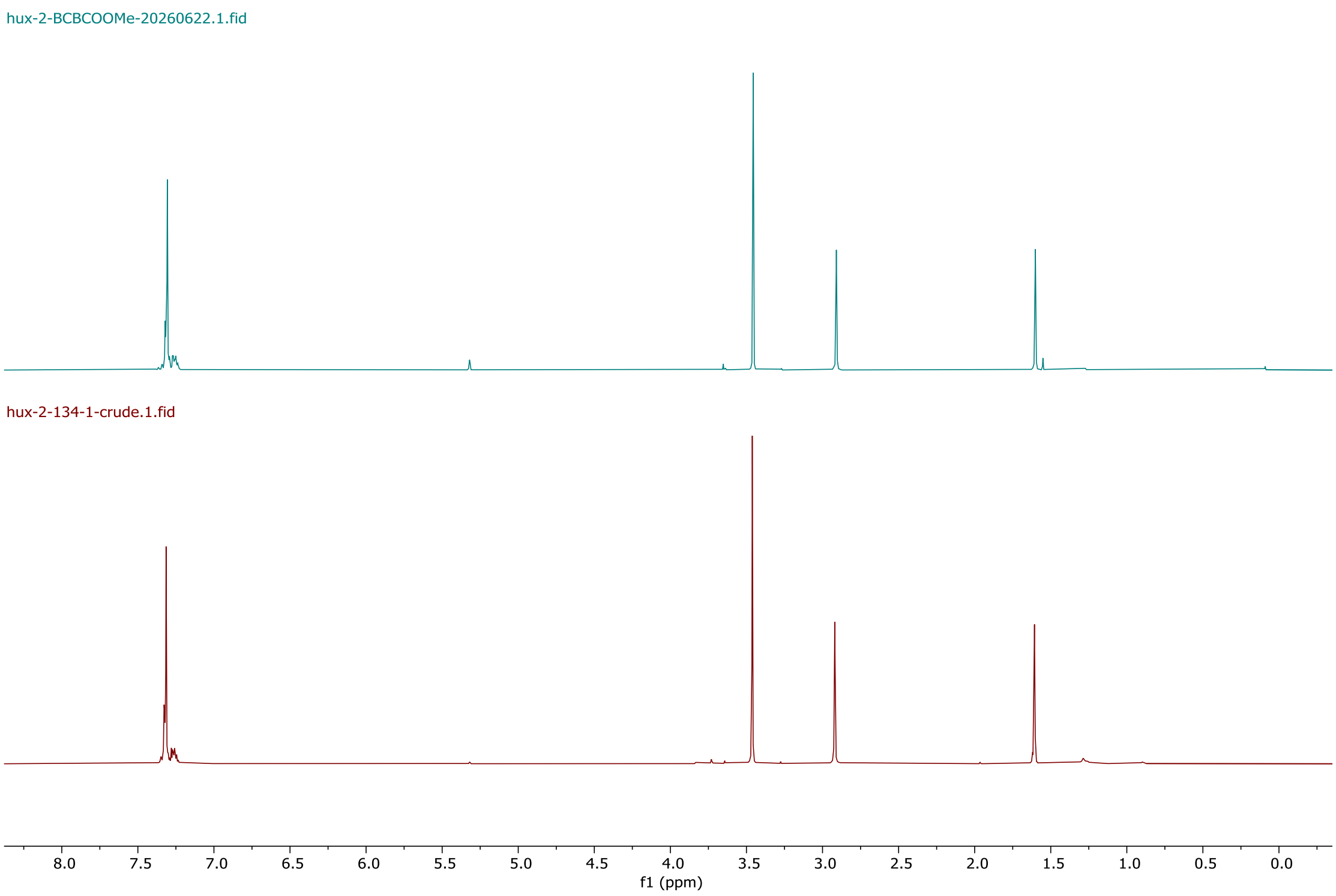

Figure S1. ^{1}H spectra of **BCB1** (top) and the crude mixture (bottom) from photoiniferter-mediated homopolymerization of **BCB1** in CD_2Cl_2.

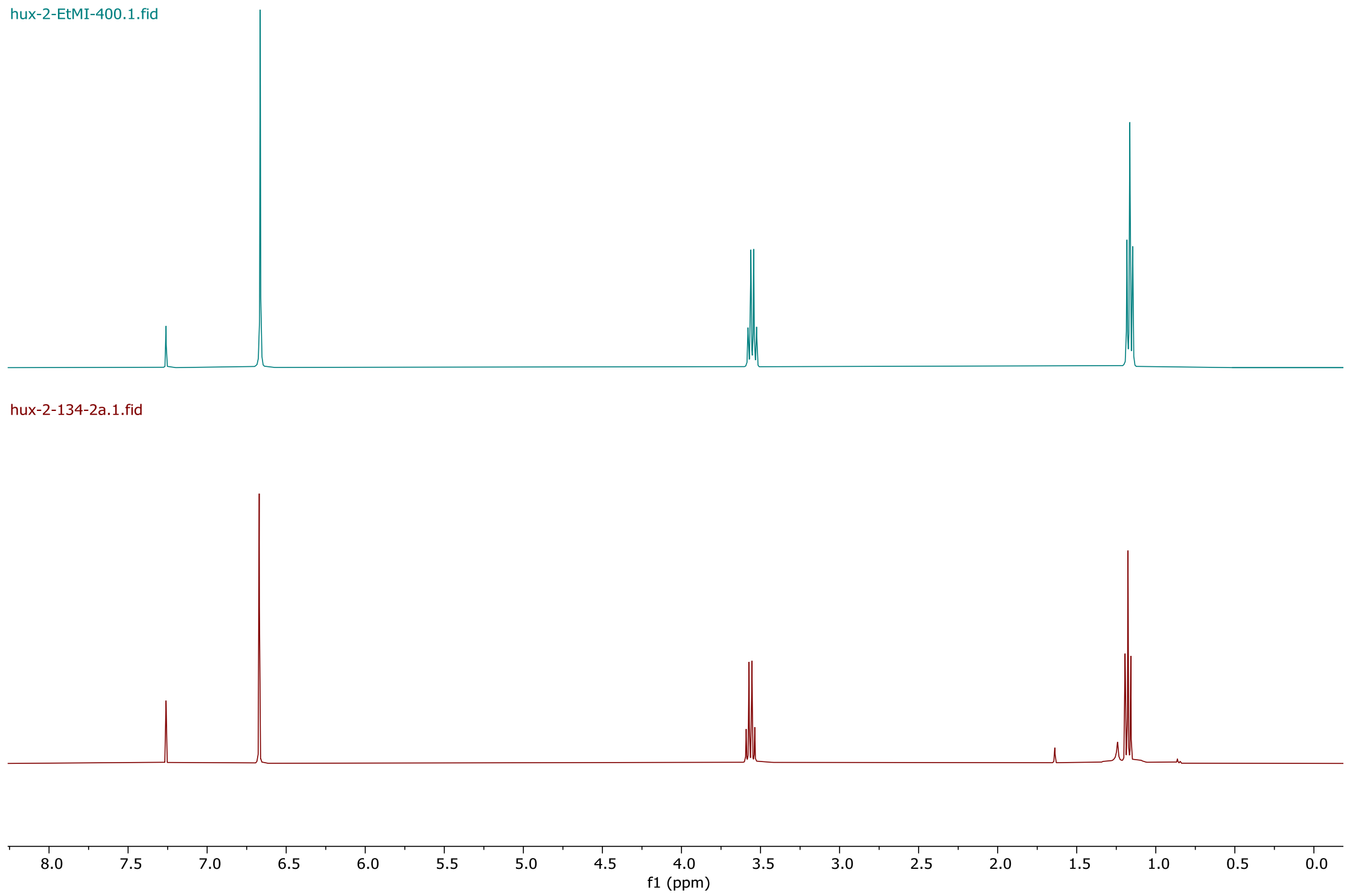

Figure S2. ^{1}H spectra of **EtMI** (top) and the crude mixture (bottom) from photoiniferter-mediated homopolymerization of **EtMI** in $CDCl_3$.

7. Radical Trapping Experiment

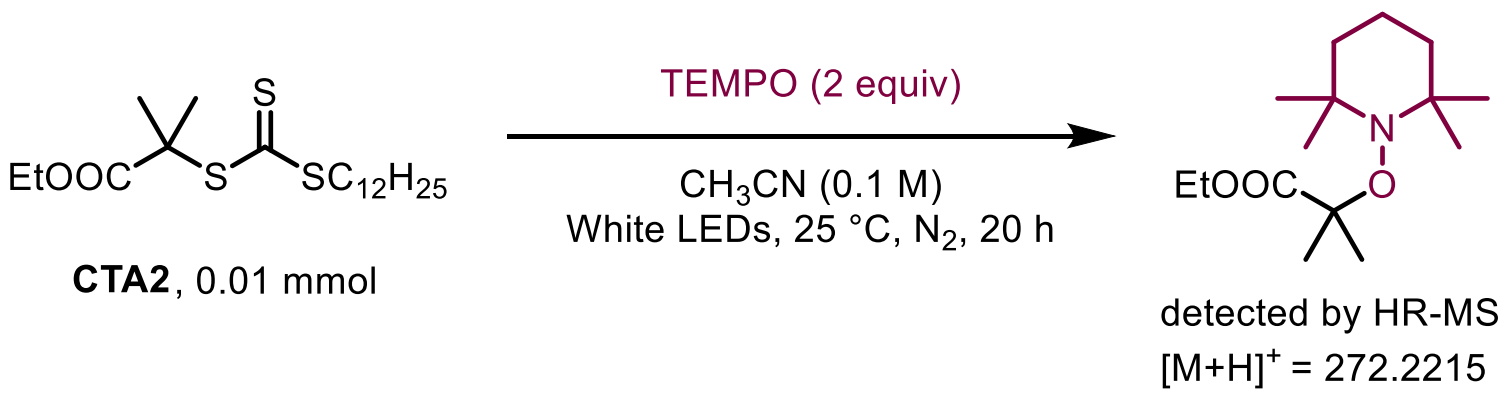

Scheme S15. General procedure for radical trapping experiment.

In a nitrogen-filled glovebox, a 5 mL reaction vial equipped with a magnetic stirring bar was charged with **CTA 2** (0.01 mmol, 1 equiv), 2,2,6,6-tetramethyl-1-piperidinyloxy (TEMPO, 0.02 mmol, 2 equiv), and MeCN (100 µL). The vial was sealed, removed from the glovebox, and stirred under white light irradiation at 25 °C for 20 h. Upon completion, the resulting mixture was concentration under reduced pressure, the mixture was analyzed by HR-MS. It was found that **CTA2**-derived adduct was detected by HR-MS (Figure S3), which suggests that a carbon radical derived from **CTA2** is generated under applied conditions.

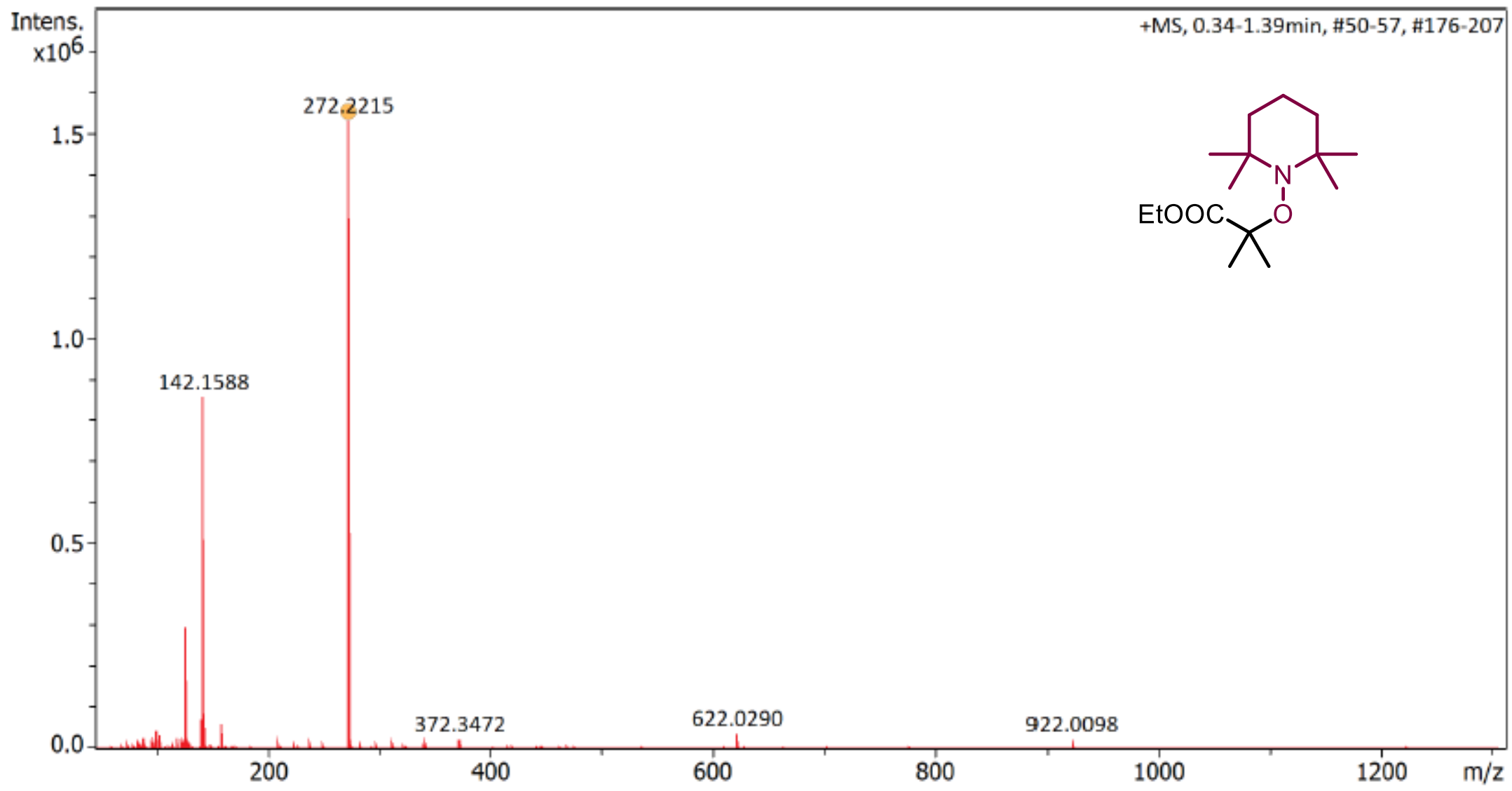

Figure S3. HR-MS for radical trapping experiment.

8. UV-vis Analysis

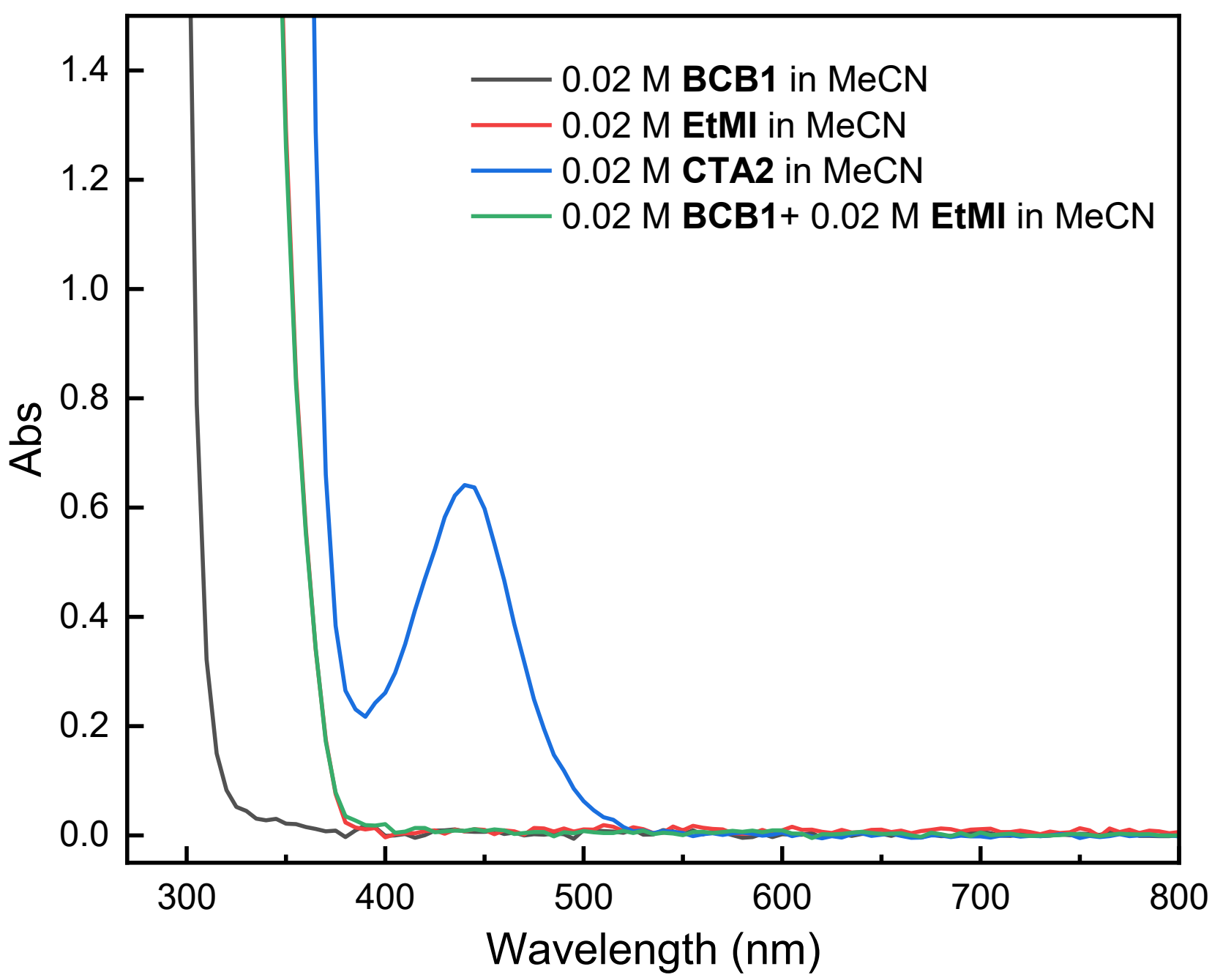

Figure S4. UV-vis absorption spectra of **BCB1**, **EtMI**, **CTA2** and a **BCB1**/**EtMI** mixture in MeCN at a concentration of 0.02 M.

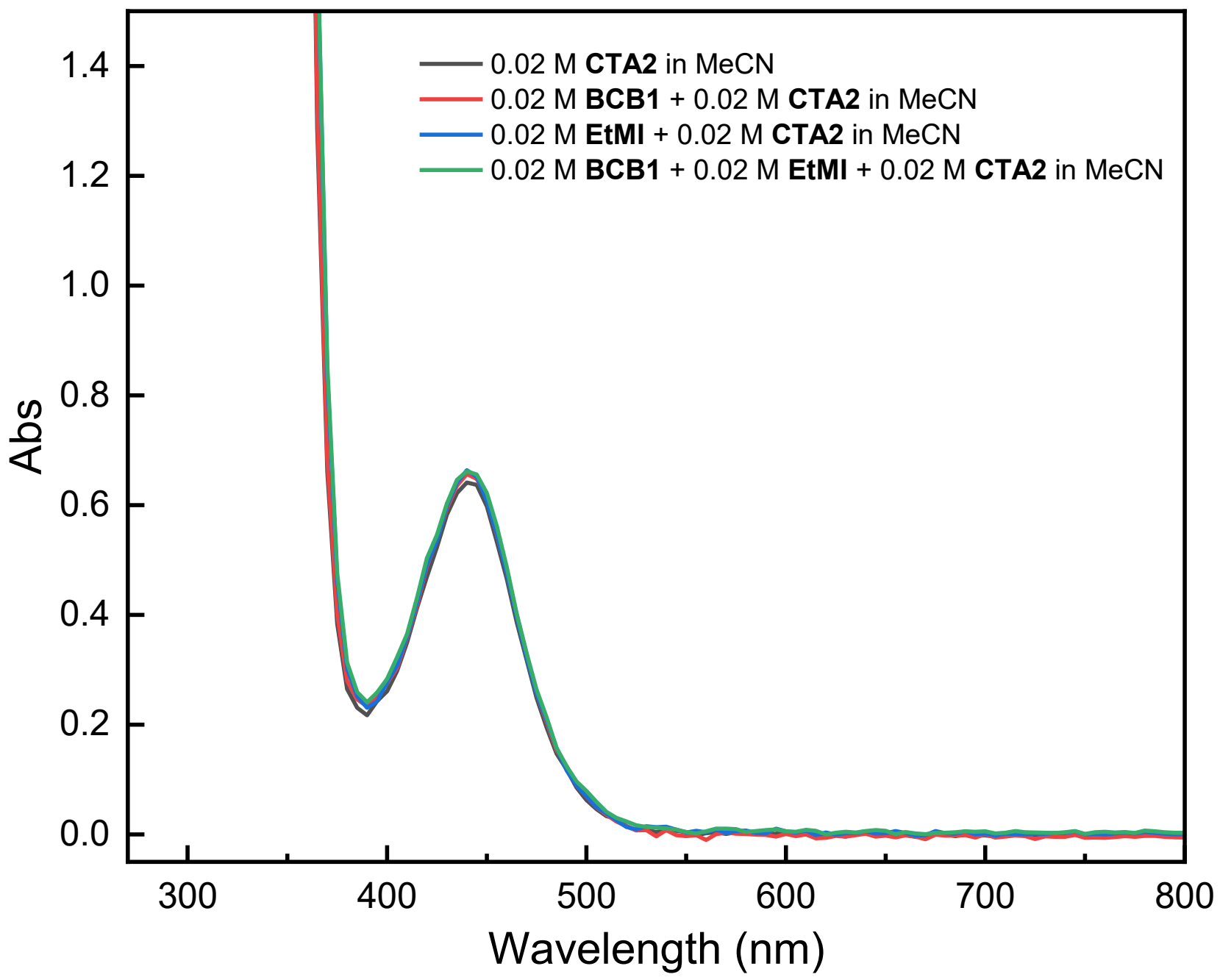

Figure S5. UV-vis absorption spectra of **CTA2** mixed with **BCB1**, **EtMI**, or both **BCB1** and **EtMI** in MeCN at a concentration of 0.02 M.

9. Solvent Effects on Photocontrolled RAFT Alternating Copolymerization

Table S3. Investigation of the solvent effects on photocontrolled RAFT alternating copolymerization of BCB1 with EtMI

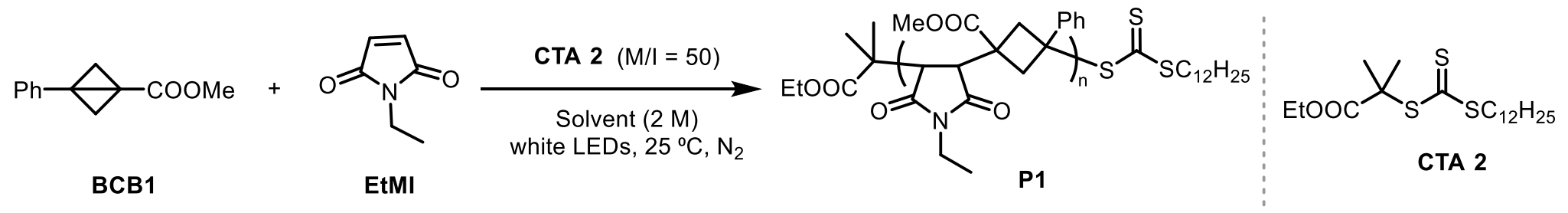

Entry[a]	Solvent	Conversion (%)[c]		$M_{n,SEC}$ (kDa)[d]	$Đ$[d]	F_{BCB1} (%)[e]
		BCB1	EtMI			
1	DMSO	95	96	13.2	1.29	50
2	DMA	87	88	11.7	1.28	50
3	DMF	89	90	11.9	1.28	50
4	Toluene	84	83	11.3	1.22	50
5	Benzene	71	72	9.9	1.22	50
6	1,4-dioxane	82	80	11.5	1.22	50
7[b]	CH_3CN	95	95	12.8	1.21	50
8[b]	DCM	96	97	11.8	1.21	50

[a] Reaction conditions: **BCB1** (0.2 mmol, 1.0 equiv), **EtMI** (0.2 mmol, 1.0 equiv), **CTA 2** (0.004 mmol, 2 mol%), solvent = 100 μL, white LEDs, N_2, 25 °C, 6 h. [b] Reaction time = 20 h. [c]Monomer conversions were determined by GC using naphthalene as an internal standard. [d] M_n and $Đ$ were determined by SEC in THF. [e] The **BCB1** incorporation ratio (F_{BCB1}) was determined by ^1H NMR of isolated polymer.

Supplementary discussion: A broader range of solvents with different polarities was systematically examined using **CTA2** as the chain-transfer agent to investigate the solvent effects. The possible solvent dependence of **CTA2** photoactivation was evaluated by UV-vis absorption spectroscopy. **CTA2** exhibited essentially the same absorption range in different solvents, suggesting that solvent polarity does not markedly affect its ground-state absorption (Figure S6). To determine whether the solvent effect was associated with the formation of charge-transfer complexes, the interaction between **BCB1** and **EtMI** was investigated by ^1H NMR spectroscopy. An equimolar mixture of **BCB1** and **EtMI** displayed

negligible chemical-shift changes in CD_2Cl_2, DMSO-d_6, and C_6D_6 relative to the individual monomers, with a maximum change of only 0.01 ppm for the vinyl protons of **EtMI** (Figures S7–S9). These results indicate weak interactions between the comonomers and suggest that strongly associated charge-transfer complexes are unlikely to play a dominant role [7]. Taken together, the observed solvent effect may primarily reflect differences in the solvation of the monomers and propagating radicals, which can alter local monomer concentrations and propagation kinetics [8].

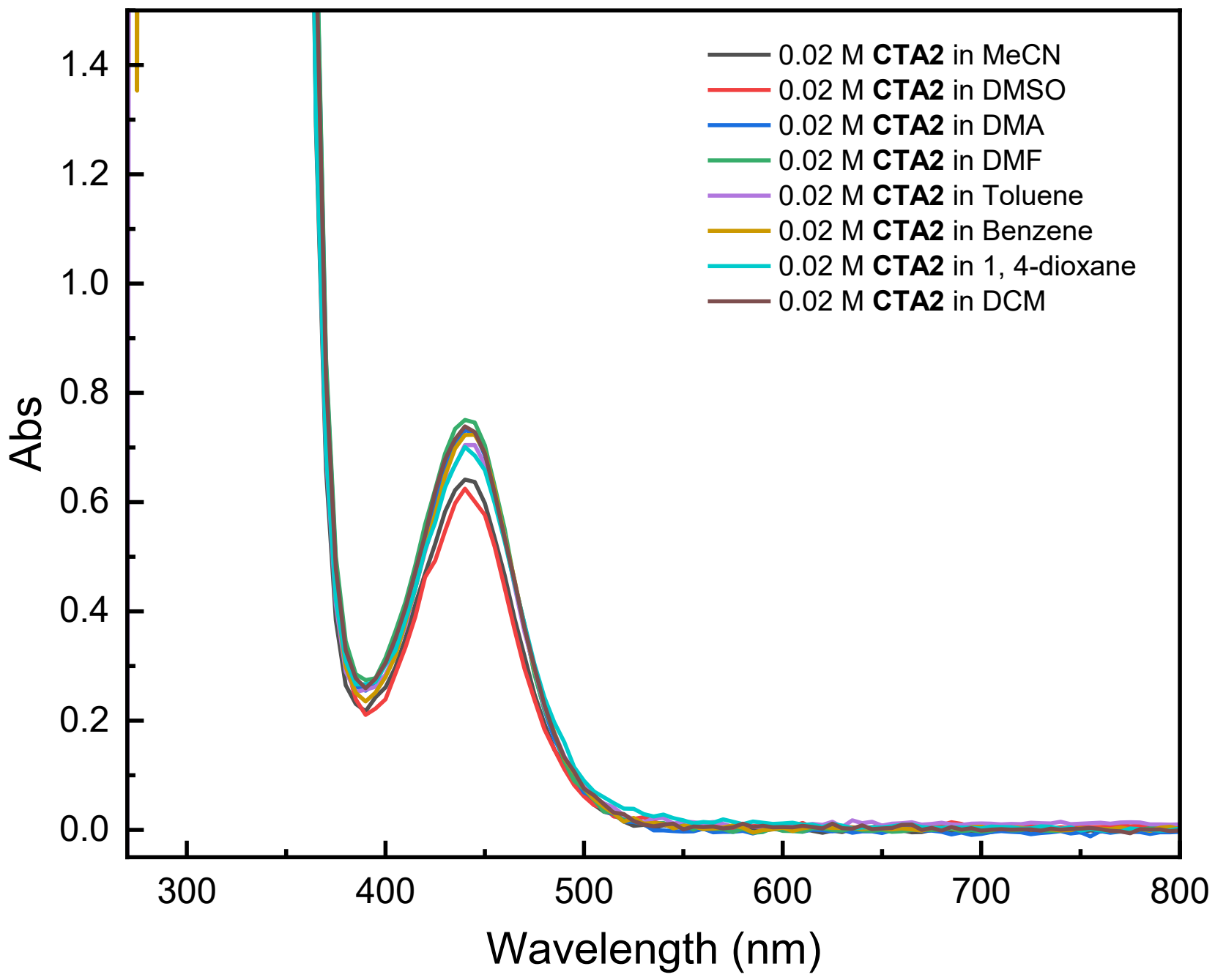

Figure S6. UV-vis absorption spectra of **CTA2** in different solvents at a concentration of 0.02 M.

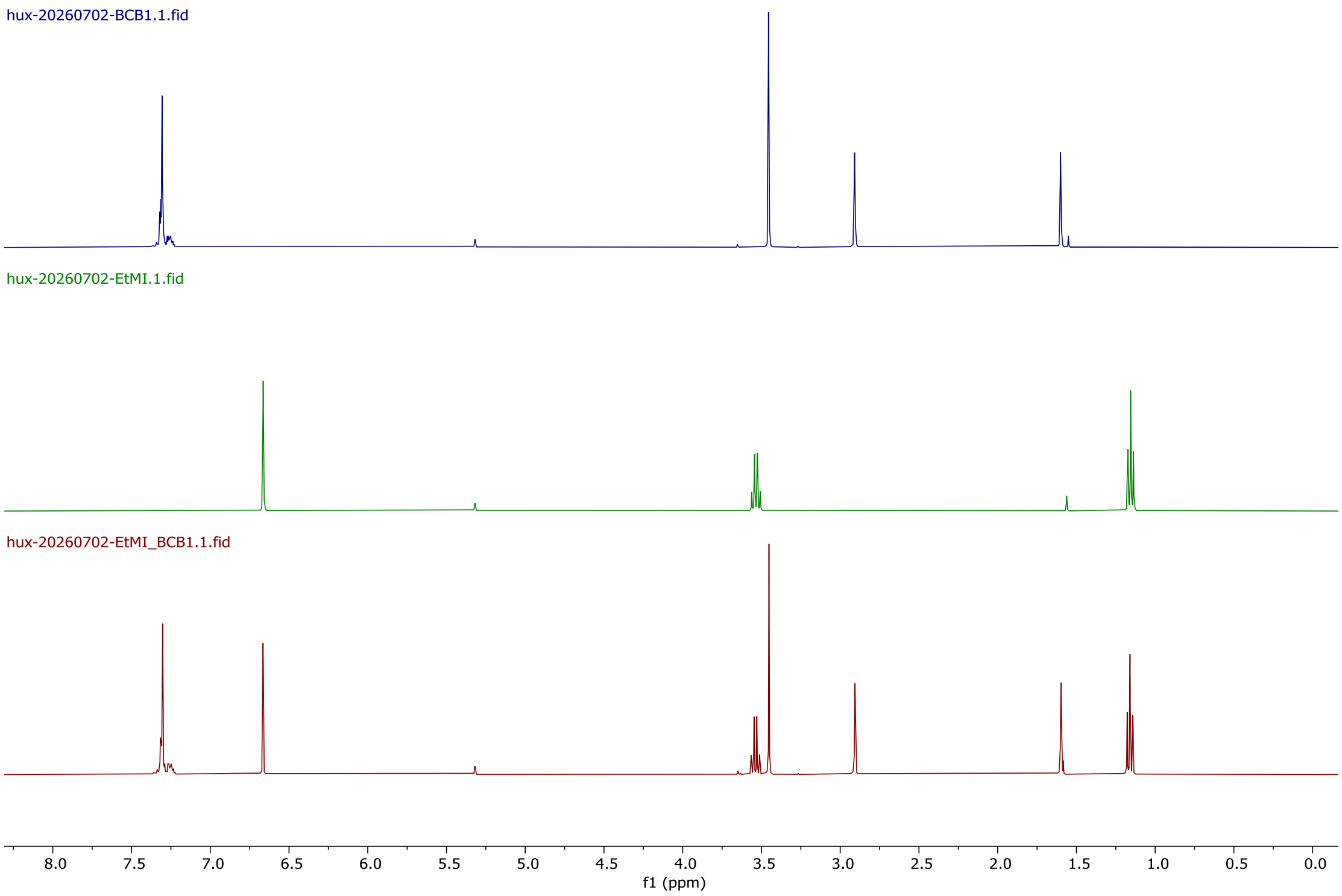

Figure S7. ^{1}H NMR analysis of **BCB1** (top), **EtMI** (middle), and an equimolar **BCB1/EtMI** mixture (bottom) in CD_2Cl_2 (chemical shift is 0.004 ppm for the vinyl protons of **EtMI**).

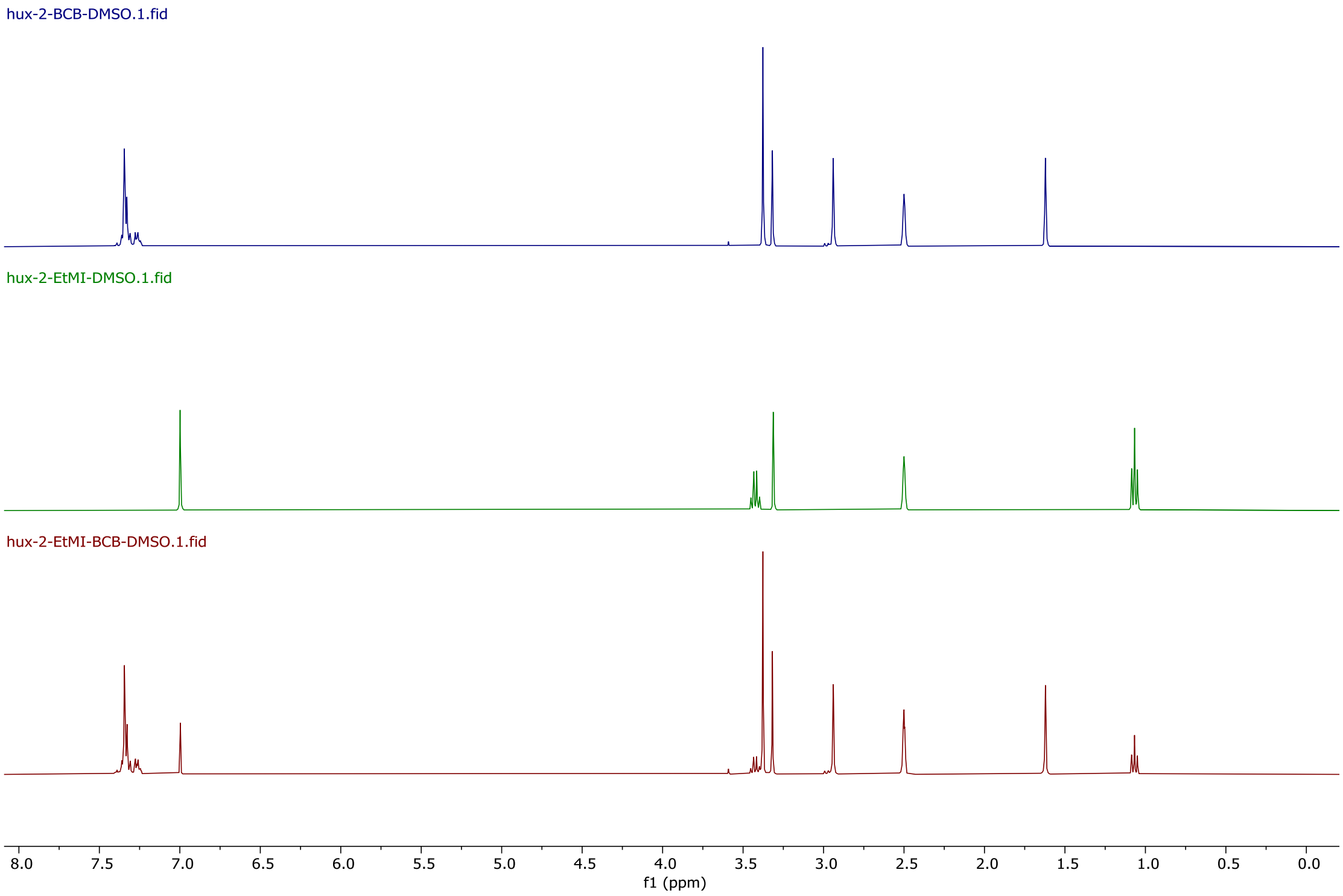

Figure S8. ^{1}H NMR analysis of **BCB1** (top), **EtMI** (middle), and an equimolar **BCB1/EtMI** mixture (bottom) in DMSO-d_6 (chemical shift is 0.002 ppm for the vinyl protons of **EtMI**).

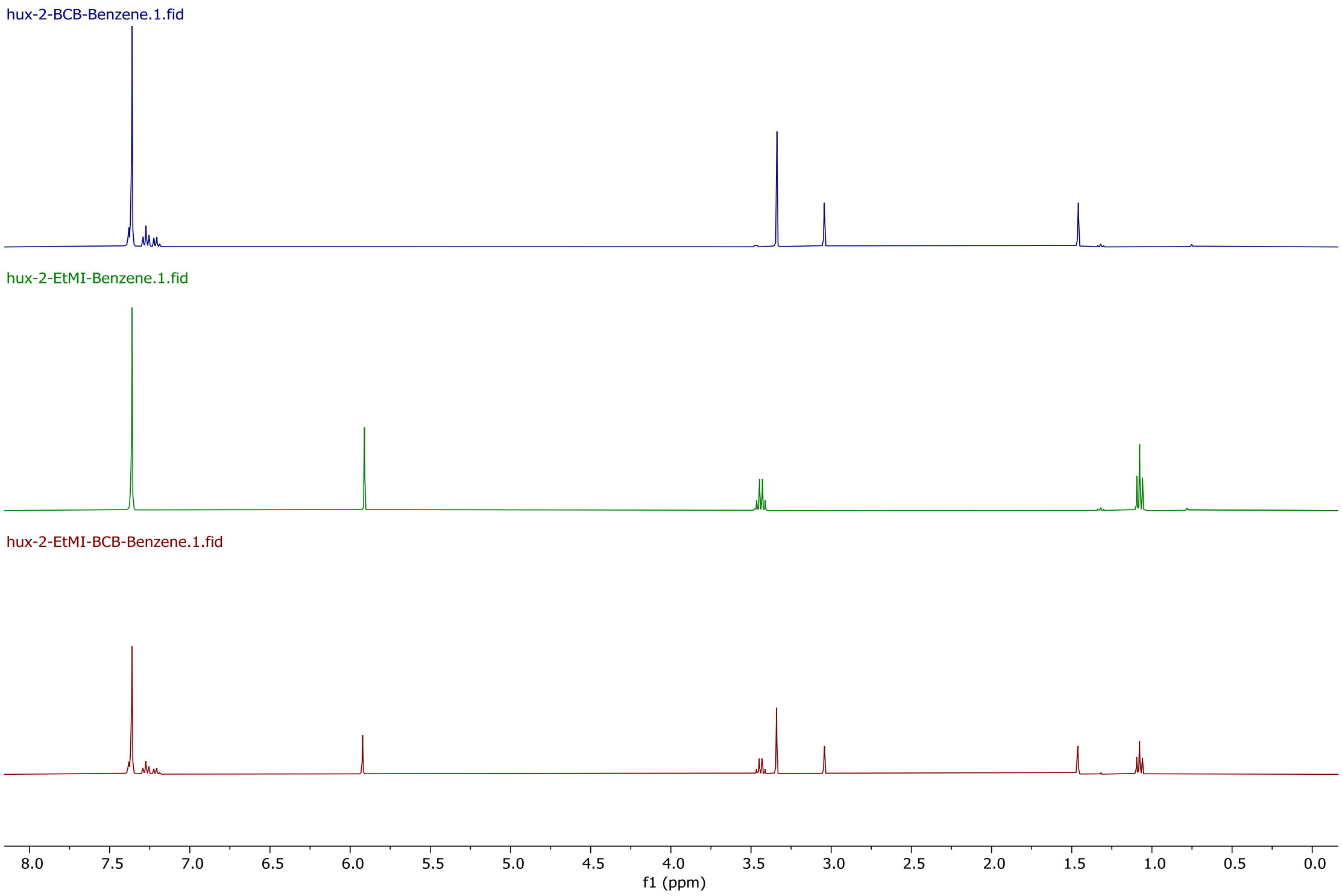

Figure S9. ^{1}H NMR analysis of **BCB1** (top), **EtMI** (middle), and an equimolar **BCB1/EtMI** mixture (bottom) in C_6D_6 (chemical shift is 0.01 ppm for the vinyl protons of **EtMI**).

10. Molecular Weight Control Experiments

Molecular weight control studies were carried out using the general procedure A (GP-A) with varying $[M]_0/[CTA]_0$ (10, 25, 50, 100, 200, 400). Monomer conversions were determined by GC using naphthalene as an internal standard. **BCB1** incorporation ratio $F_{\mathbf{BCB1}}$ was determined by ^{1}H NMR of isolated polymer. The molecular weight of the resulting copolymers was determined by SEC. Plots of M_n and $Đ$ as a function of $[M]_0/[CTA]_0 \times$ conversion and the corresponding SEC traces were obtained from molecular weight control experiments.

Table S4. Molecular weight control experiments

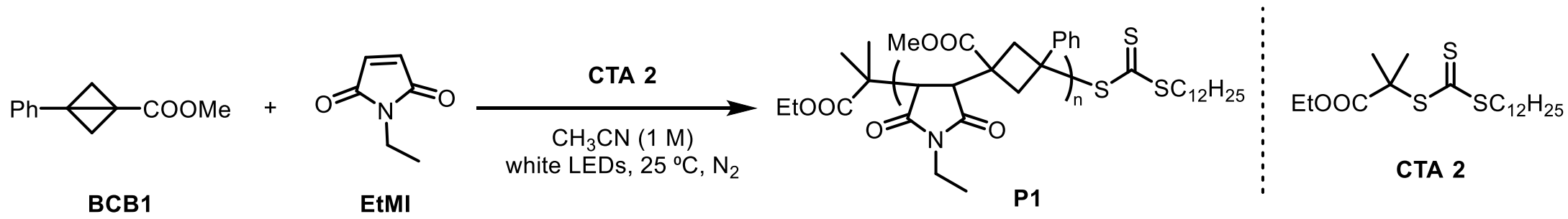

Entry[a]	BCB1:EtMI:CTA 2	Conversion (%)[b] BCB1	Conversion (%)[b] EtMI	$M_{n,SEC}$ (kDa)[c]	$Đ$[c]	F_{BCB1} (%)[d]
1	100:100:10	90	93	3.4	1.15	49
2	100:100:4	88	91	6.5	1.20	50
3	100:100:2	95	95	12.8	1.21	50
4	100:100:1	92	94	22.4	1.29	50
5	100:100:0.5	87	88	39.3	1.37	51
6	100:100:0.25	86	87	76.6	1.45	51

[a] Reaction conditions: **BCB1** (0.2 mmol, 1.0 equiv), **EtMI** (0.2 mmol, 1.0 equiv), **CTA 2**, CH_3CN = 100 μL, white LEDs, N_2, 25 °C, 20 h. [b] Monomer conversions were determined by GC using naphthalene as an internal standard. [c] M_n and $Đ$ were determined by SEC in THF. [d] The **BCB1** incorporation ratio (F_{BCB1}) was determined by ^{1}H NMR of isolated polymer.

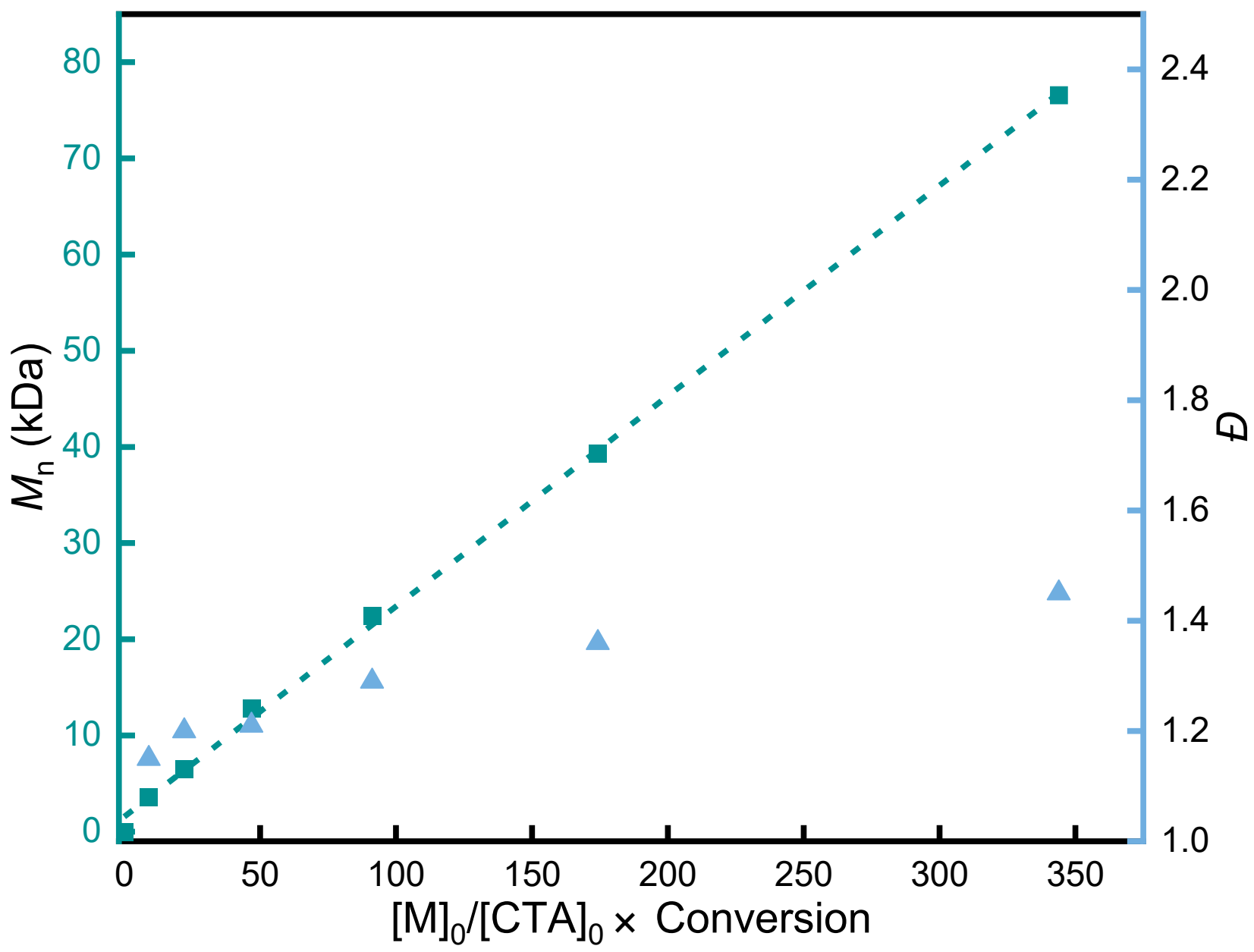

Figure S10. Plots of M_n and $Đ$ as a function of $[M]_0/[CTA]_0 \times$ conversion.

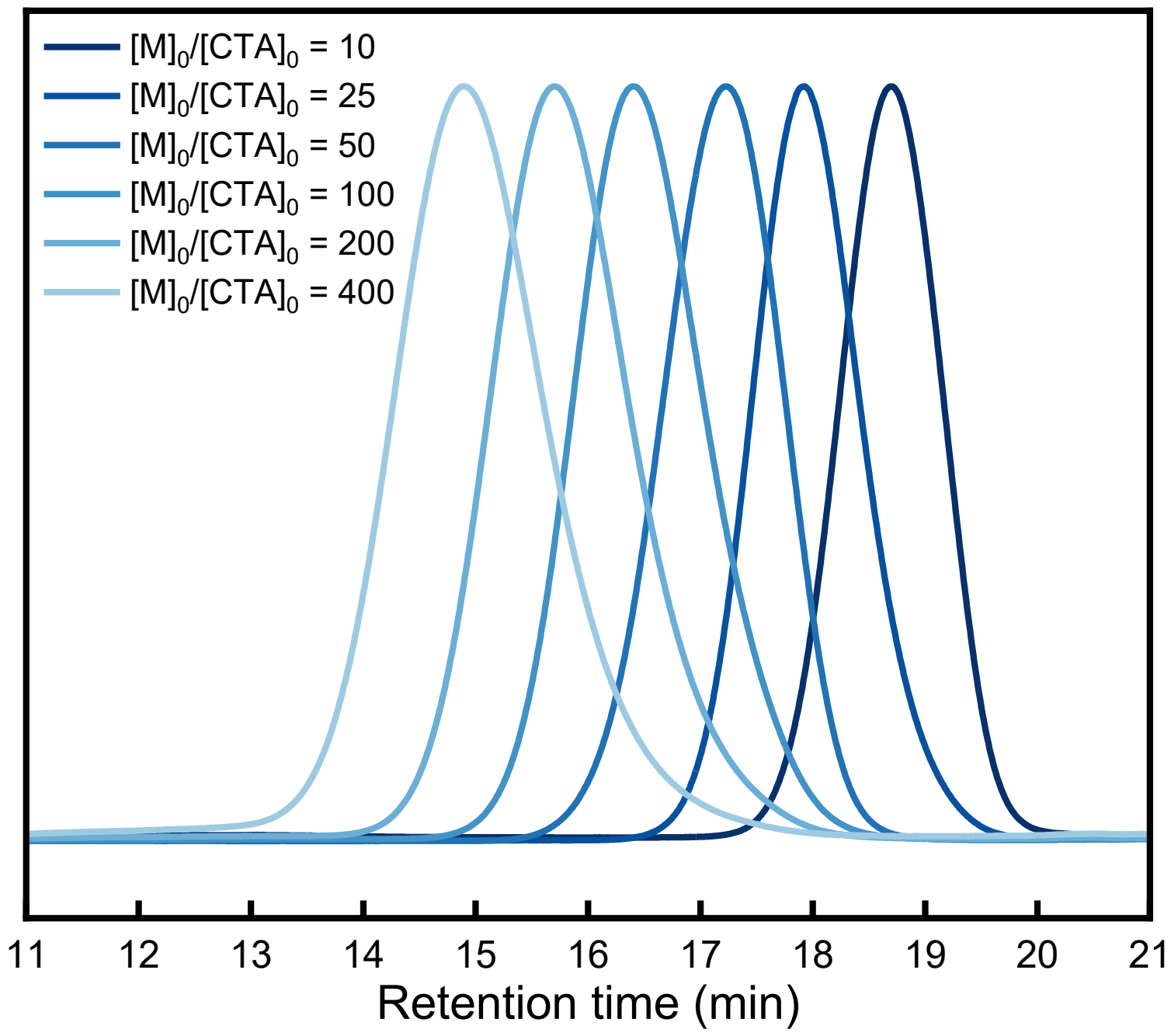

Figure S11. SEC traces from molecular weight control experiments.

11. Kinetic Studies

11-1. Kinetic studies of alternating copolymerization of BCB1 and EtMI

Kinetic studies of alternating copolymerization of **BCB1** and **EtMI** were carried out using the modified conditions of general procedure A (GP-A) using 200 uL CH_3CN as solvent with varying reaction time (1, 2, 4, 6, 8, 12, 20, 24 h). Monomer conversions were determined by GC using naphthalene as an internal standard. **BCB1** incorporation ratio $F_{\mathbf{BCB1}}$ was determined by ^{1}H NMR of isolated polymer. The molecular weight of the resulting copolymers was determined by SEC. The plots of M_n and $Đ$ as a function of monomer conversion and the corresponding SEC traces were obtained from kinetic experiments.

Table S5. Kinetic studies of alternating copolymerization of BCB1 and EtMI

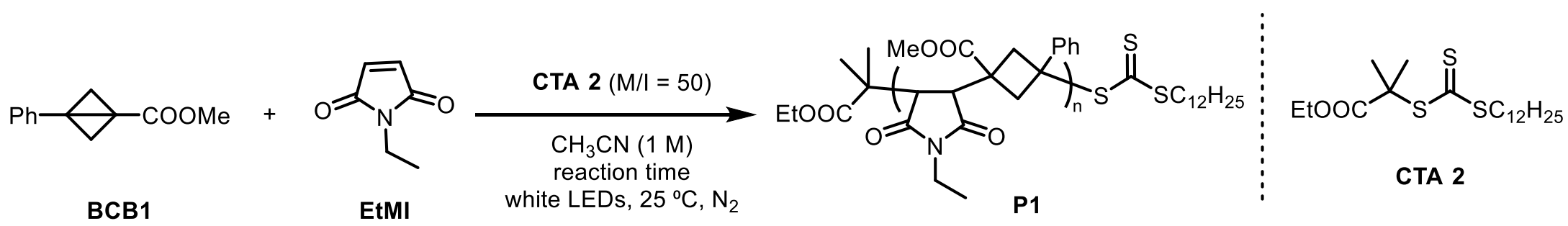

Entry[a]	Reaction time	Conversion (%)[b]		$M_{n,SEC}$ (kDa)[c]	$Đ$[c]	$F_{\mathbf{BCB1}}$ (%)[d]
		**BCB1**	**EtMI**			
1	1 h	14	16	3.28	1.42	50
2	2 h	30	27	4.45	1.44	50
3	4 h	43.8	44.7	6.45	1.37	50
4	6 h	58	61	8.50	1.25	51
5	8 h	70.5	72.6	10.25	1.23	50
6	12 h	80.9	82.2	11.58	1.20	51
7	20 h	92.5	94.3	12.71	1.21	50
8	24 h	94.7	95.2	12.83	1.21	50

[a] Reaction conditions: **BCB1** (0.2 mmol, 1.0 equiv), **EtMI** (0.2 mmol, 1.0 equiv), **CTA 2** (0.004 mmol, 2 mol%), CH_3CN = 200 μL, white LEDs, N_2, 25 °C. [b] Monomer conversions were determined by GC using naphthalene as an internal standard. [c] M_n and $Đ$ were determined by SEC in THF. [d] The **BCB1** incorporation ratio ($F_{\mathbf{BCB1}}$) was determined by ^{1}H NMR of isolated polymer.

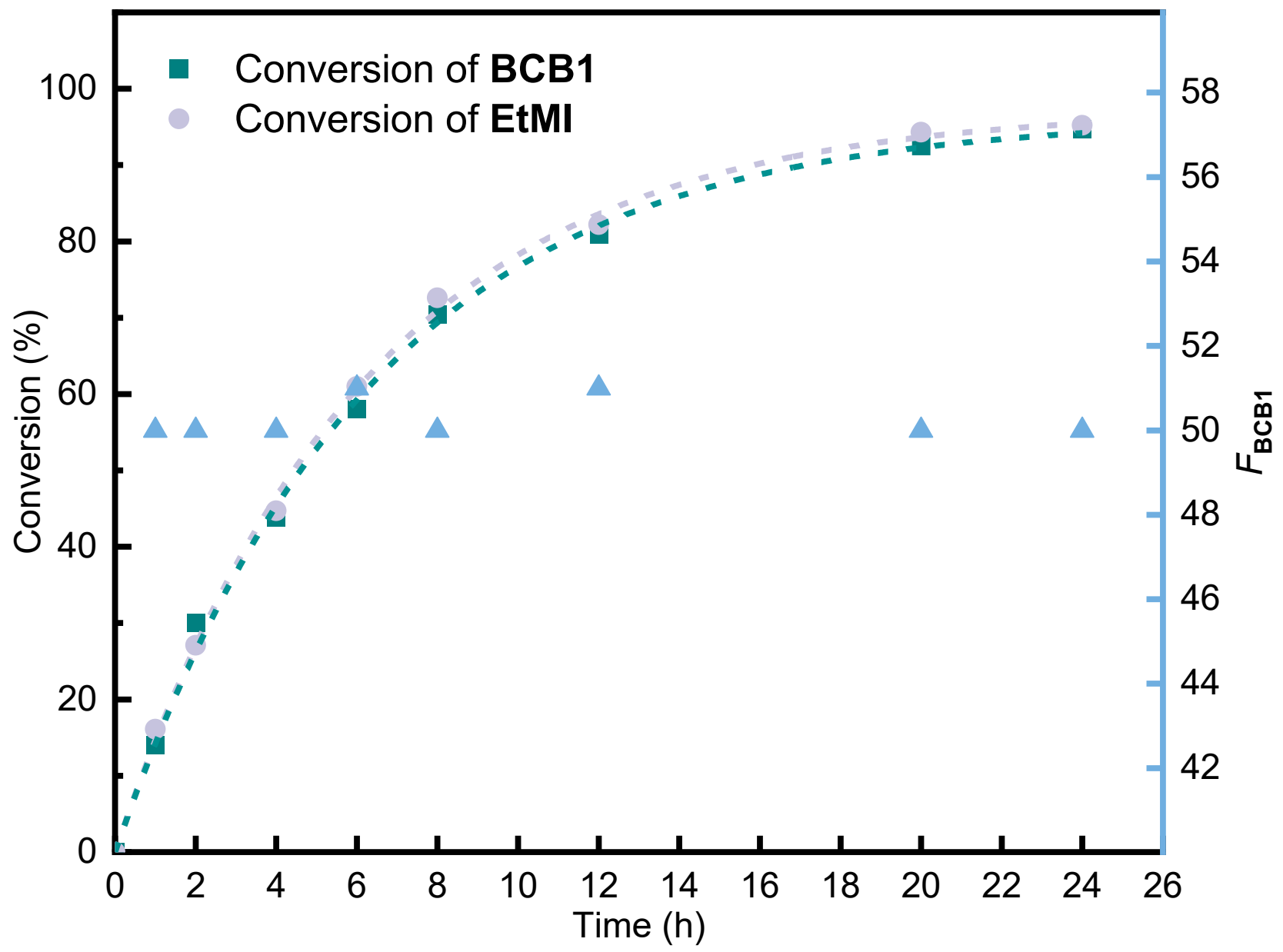

Figure S12. Plots of **BCB1** and **EtMI** conversions and F_{BCB1} versus time.

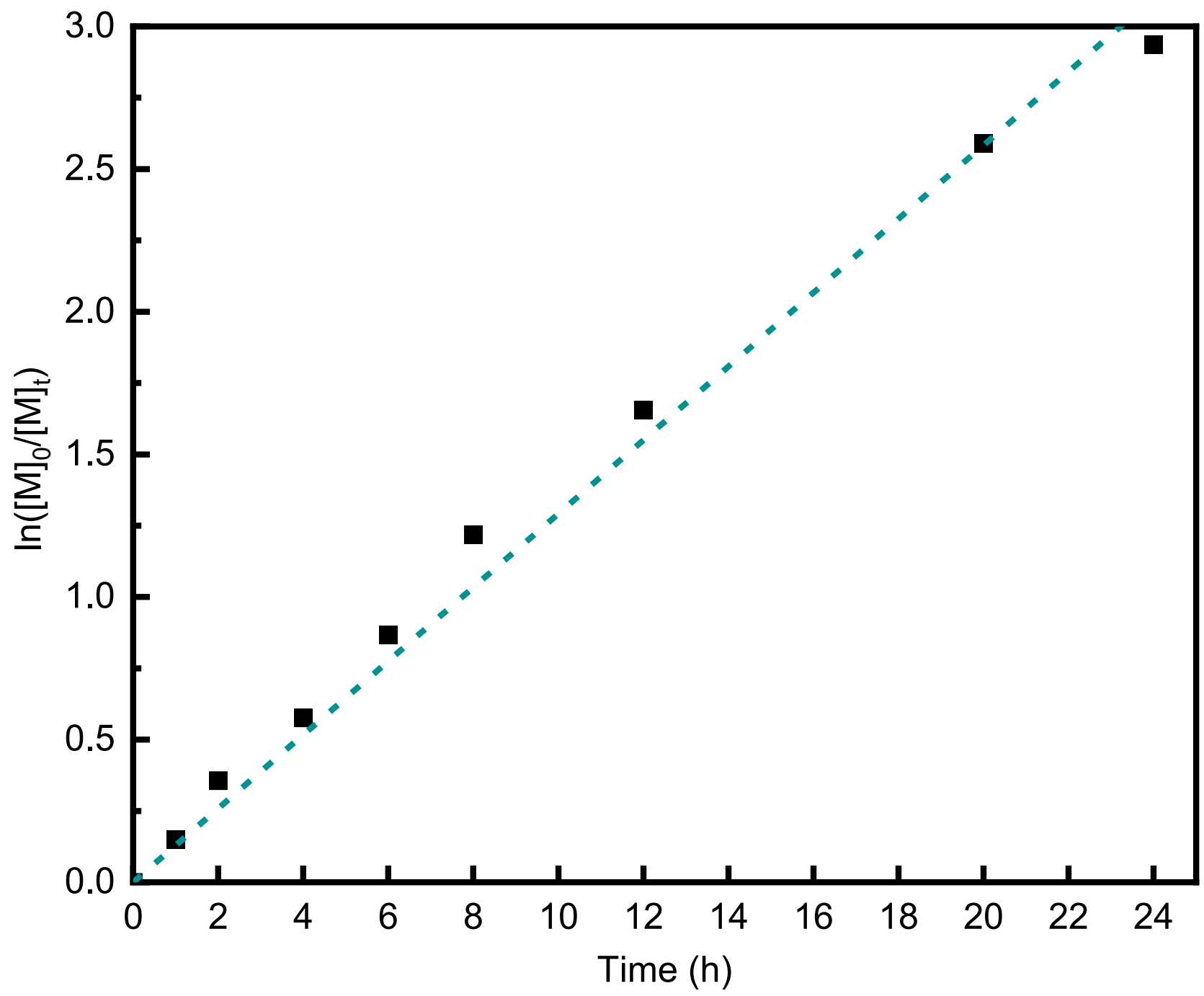

Figure S13. ln([M]$_0$/[M]$_t$) vs time plots from kinetic experiments.

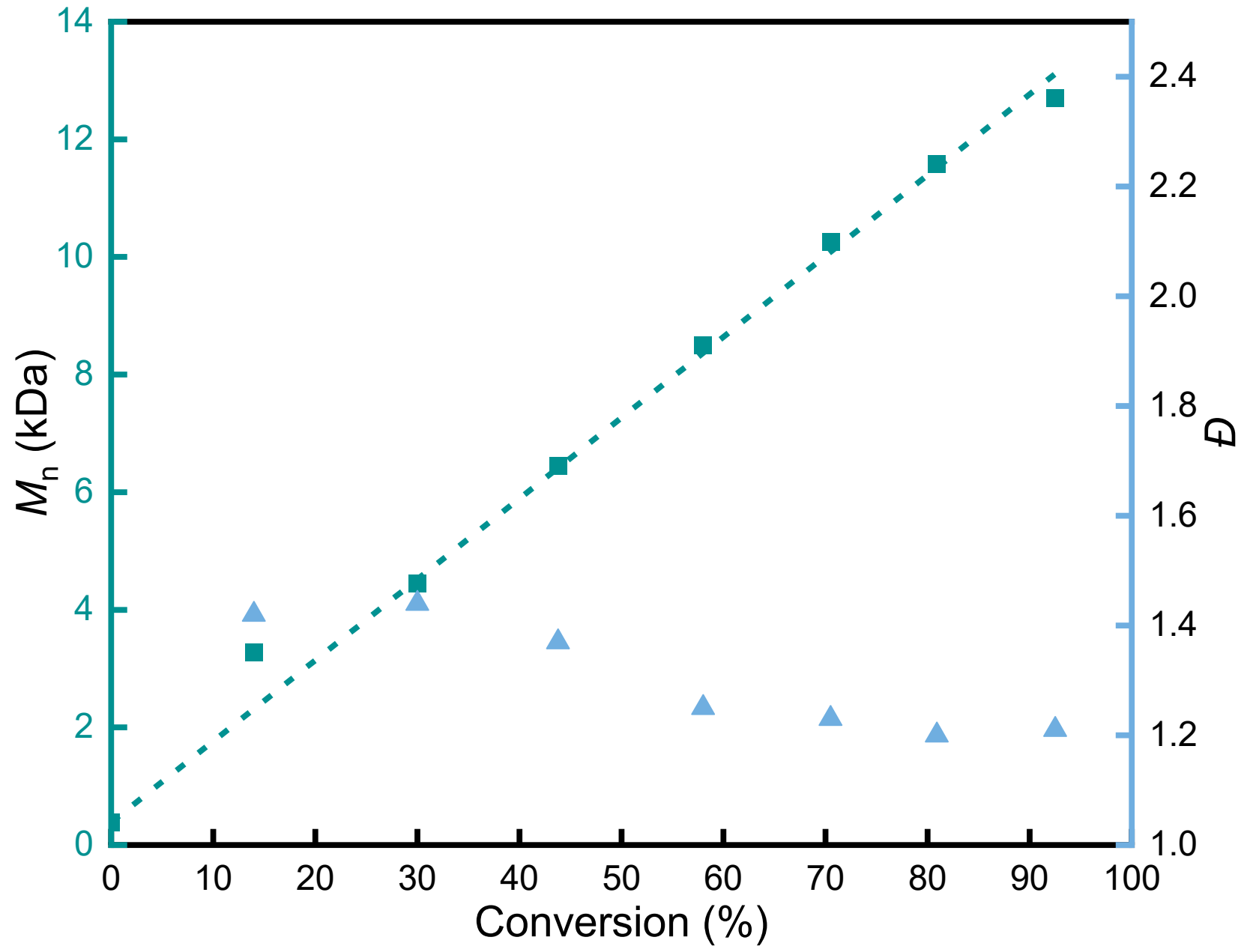

Figure S14. Plots of M_n and $Đ$ as a function of monomer conversion.

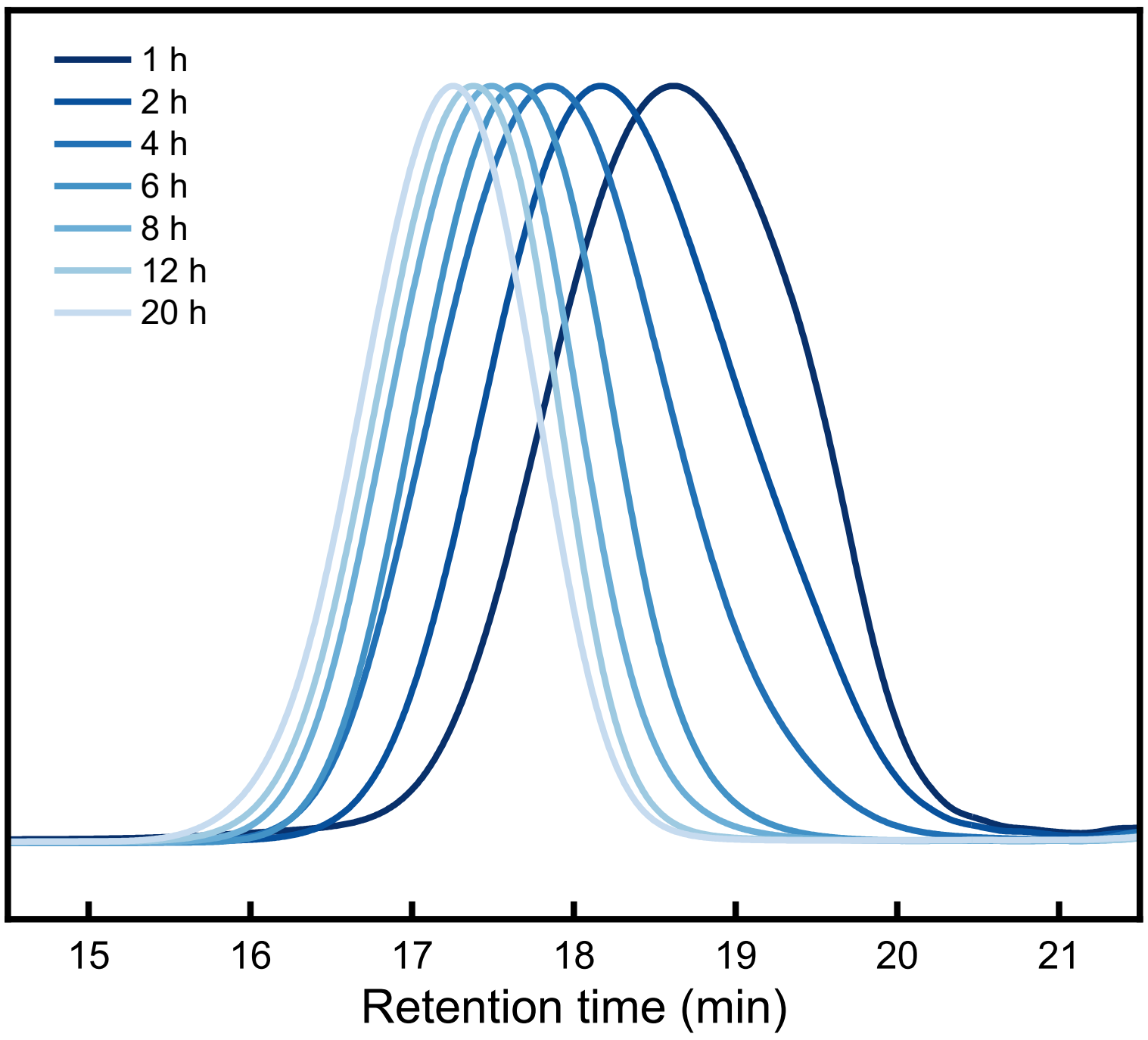

Figure S15. SEC traces (THF) from kinetic experiments.

11-2. Kinetic studies of copolymerization of BCB2 and EtMI

Kinetic studies of copolymerization of **BCB2** and **EtMI** were carried out using the modified conditions of general procedure A (GP-A) using **BCB2** and **EtMI** as comonomer with varying reaction time (2, 3, 4, 5, 6, 7, 10, 12, 16, 24 h). Monomer conversions were determined by GC using naphthalene as an internal standard. **BCB2** incorporation ($F_{\mathbf{BCB2}}$) at 12 h was determined by ^{1}H NMR of isolated polymer.

Note: The monomer incorporation ratio under photoinitiated conditions differed from that under

thermally initiated conditions. The incorporated **BCB2**/**EtMI** ratio was 1.1:1 at 12 h under photoiniferter-mediated conditions, whereas the incorporated **EtMI**/**BCB2** ratio was 1.1:1 at 0.5 h under thermally initiated conditions.

Table S6. Kinetic studies of copolymerization of BCB2 and EtMI

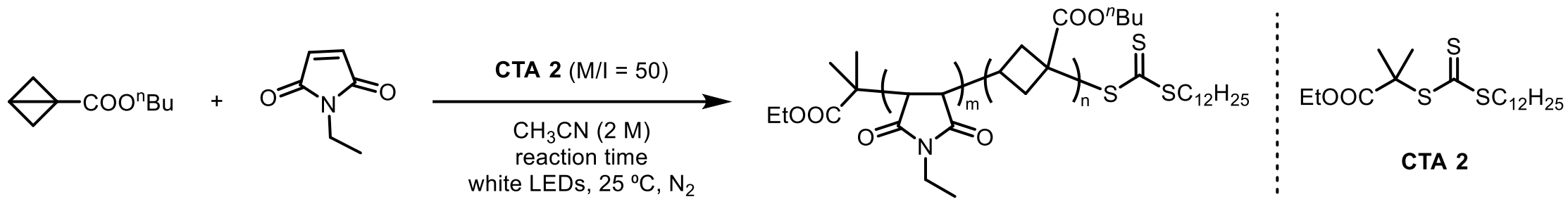

Entry	Reaction time	Conversion (%)[b]	
		BCB2	EtMI
1	2 h	9	5
2	3 h	22	17
3	4 h	37	33
4	5 h	45	42
5	6 h	63	59
6	7 h	72	67
7	10 h	85	81
8	12 h	90	86
9	16 h	93	90
10	24 h	98	95

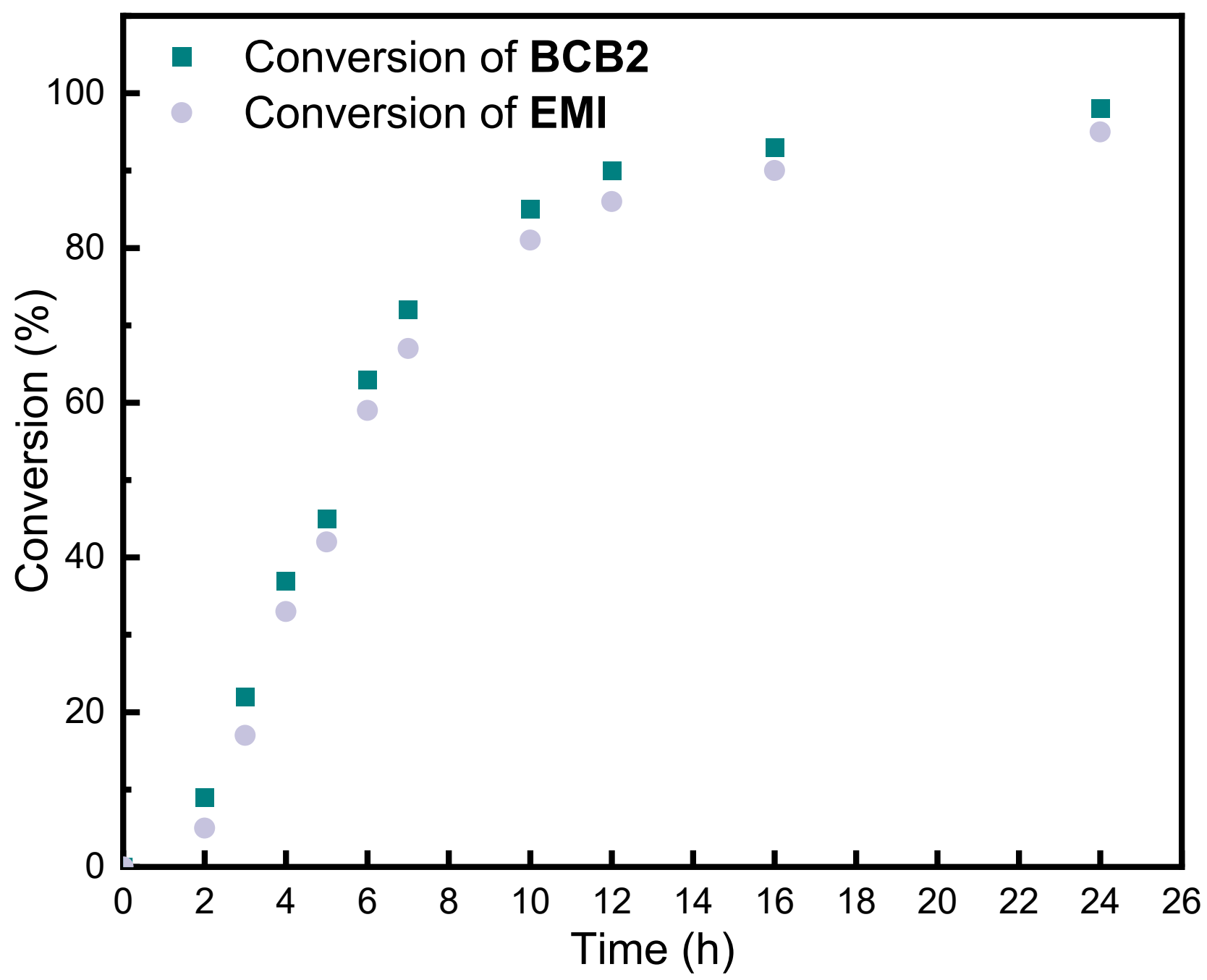

Figure S16. Plots of **BCB2** and **EtMI** conversions and F_{BCB2} versus time.

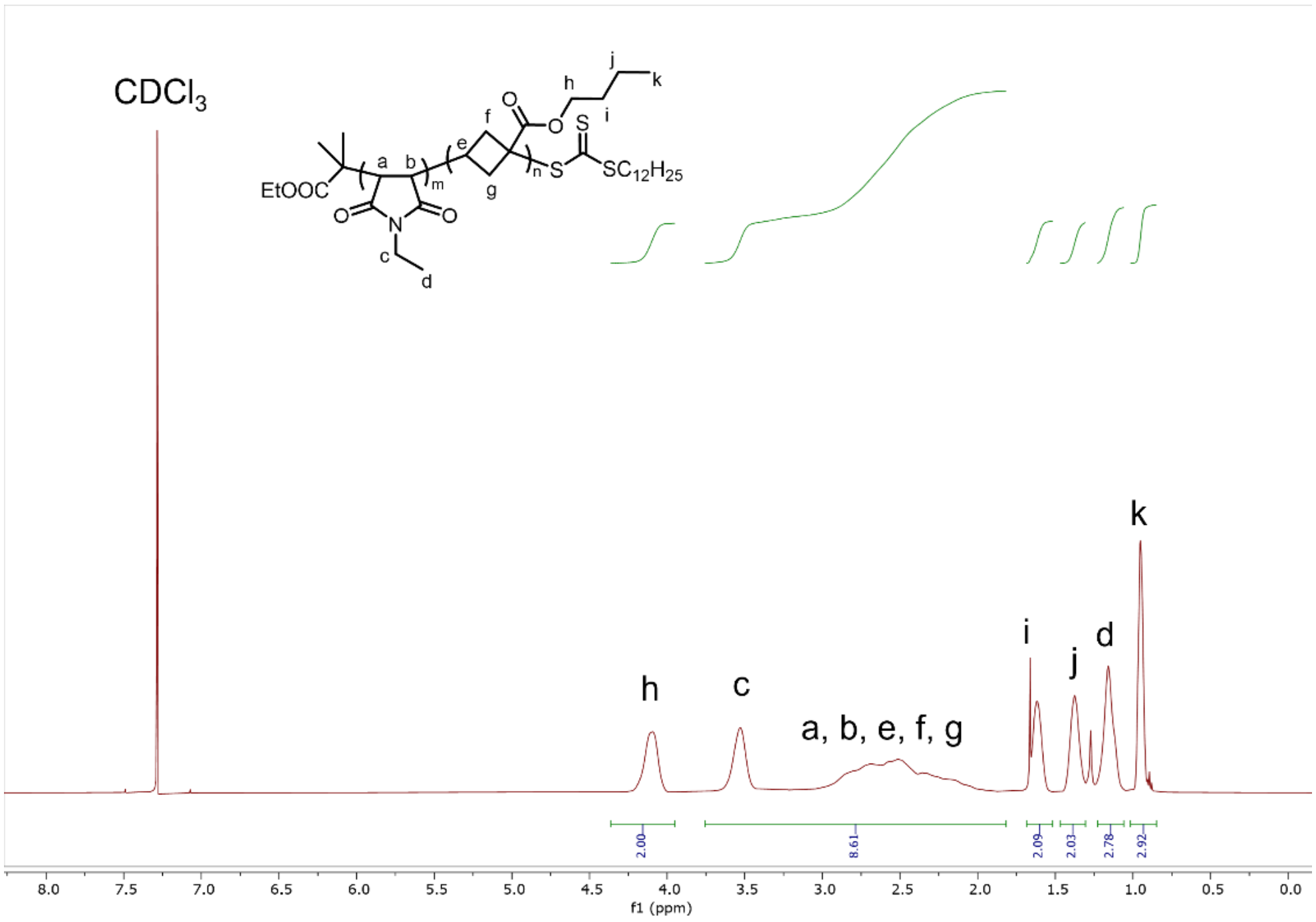

Figure S17. ^{1}H NMR spectrum of isolated **P18** obtained after 12 h in $CDCl_3$.

12. Determination of Reactivity Ratios

12-1. Reactivity ratios of BCB1/EtMI system

Copolymerization of **BCB1** and **EtMI** was carried out using the general procedure A (GP-A) with varying feed ratios ([**BCB1**] : [**EtMI**] = 20:80, 35:65, 50:50, 65:35, and 80:20). **BCB1** incorporation ratio $F_{\mathbf{BCB1}}$ was determined by ^{1}H NMR of isolated polymer. The monomer reactivity ratios r_{BCB1}= 0.014 and r_{EtMI}= 0.011 were then calculated via Mayo−Lewis plot with the $f_{\mathbf{BCB1}}$ and $F_{\mathbf{BCB1}}$ values. Note: conversion of the minor monomer was 5-10 %.

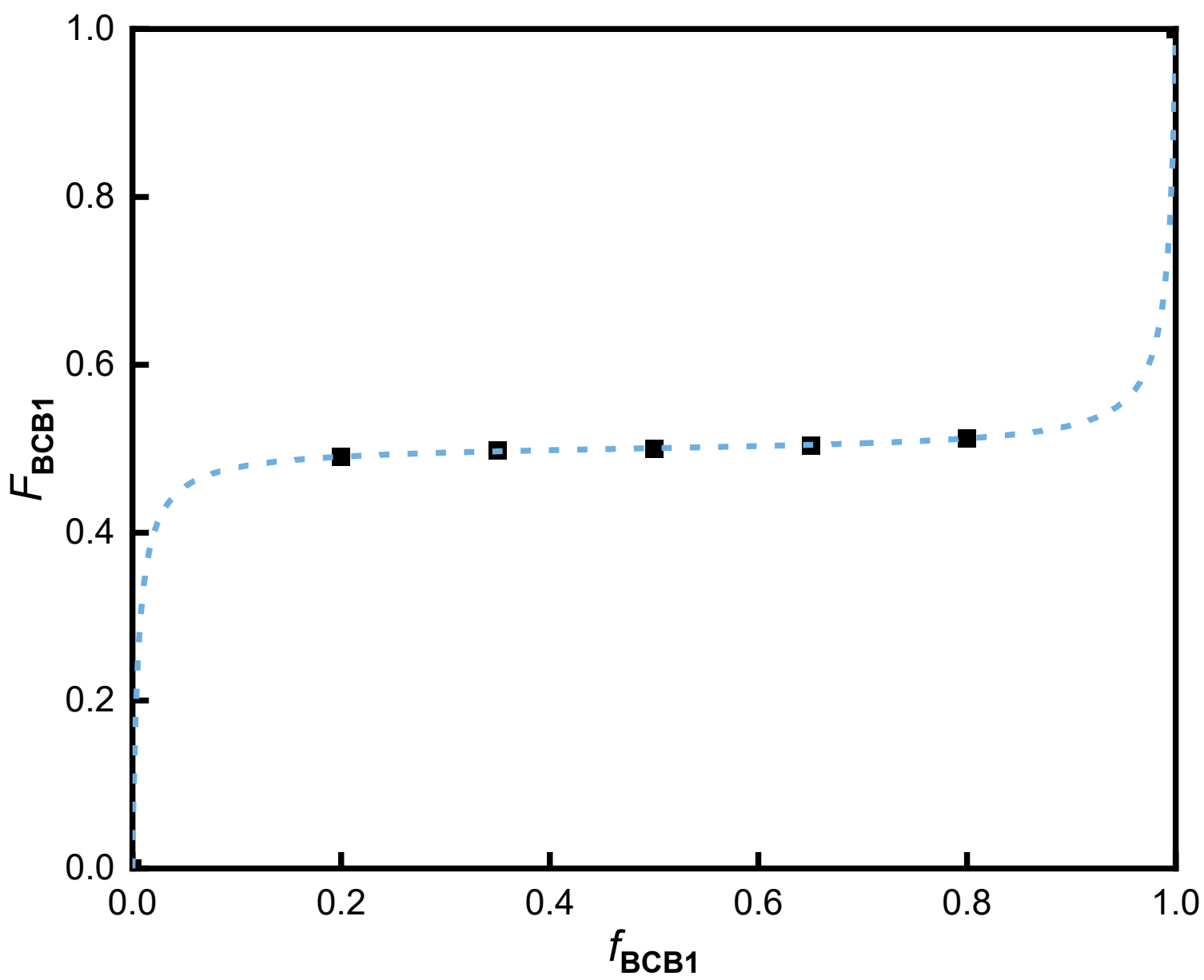

Figure S18. Mayo−Lewis plot of the **BCB1** incorporation ratio ($F_{\mathbf{BCB1}}$) versus **BCB1** feed composition ($f_{\mathbf{BCB1}}$).

12-2. Reactivity ratios of BCB2/EtMI system

Copolymerization of **BCB2** and **EtMI** was carried out using the general procedure C (GP-C) with varying feed ratios ([**BCB2**] : [**EtMI**] = 20:80, 35:65, 50:50, 65:35, and 80:20). **BCB2** incorporation ratio $F_{\mathbf{BCB2}}$ was determined by ^{1}H NMR of isolated polymer. The monomer reactivity ratios r_{BCB2}= 0.356 and r_{EtMI}= 0.436 were then calculated via Mayo–Lewis plot with the $f_{\mathbf{BCB2}}$ and $F_{\mathbf{BCB2}}$ values. Note: conversion of the minor monomer was 5-10 %.

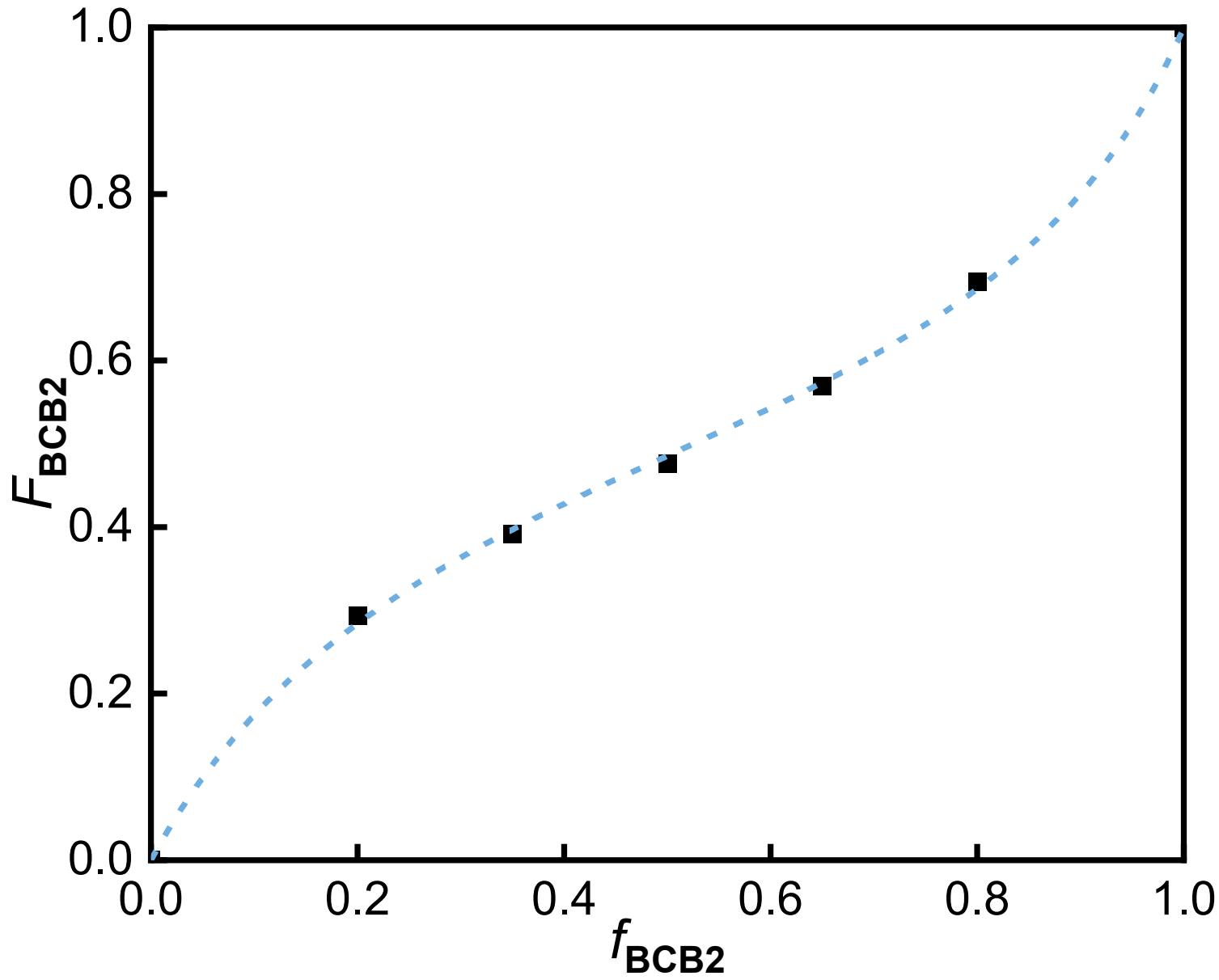

Figure S19. Mayo–Lewis plot of the **BCB2** incorporation ratio ($F_{\mathbf{BCB2}}$) versus **BCB2** feed composition ($f_{\mathbf{BCB2}}$).

13. Chain Extension

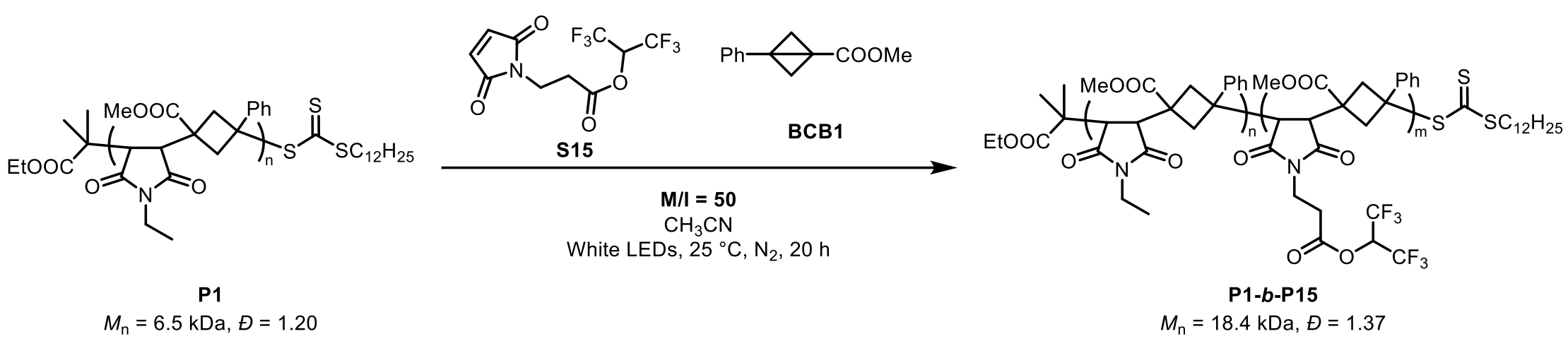

Scheme S16. Synthesis of diblock alternating copolymer **P1**-*b*-**P15**.

P1 (M_n = 6.5 kDa, $Đ$ = 1.20) was prepared according to the GP-A with $[M]_0$: $[CTA]_0$ = 25. In a nitrogen-filled glovebox, a 5 mL reaction vial equipped with a magnetic stirring bar was charged with **P1** (32, 0.1 mmol), maleimide derivatives **S15** (0.1 mmol) and **BCB1** (0.1 mmol), followed by acetonitrile (100 μL). The vial was sealed, removed from the glovebox, and stirred under white light irradiation at 25 °C for 20 h. Upon completion, the polymer solution was precipitated into pentane/Et_2O (v/v = 3:1) to give **P1**-*b*-**P15** (M_n = 18.4 kDa, $Đ$ = 1.37) as a white powder.

Note: The polymers exhibited good long-term stability, as macroinitiator **P1** (M_n = 6.5 kDa, $Đ$ = 1.20) remained unchanged after storage for more than six months at room temperature under ambient conditions without rigorous exclusion of moisture or oxygen. Furthermore, successful chain extension to afford a diblock copolymer demonstrates that the polymer retains its structural integrity and chain-end functionality during storage and light irradiation.

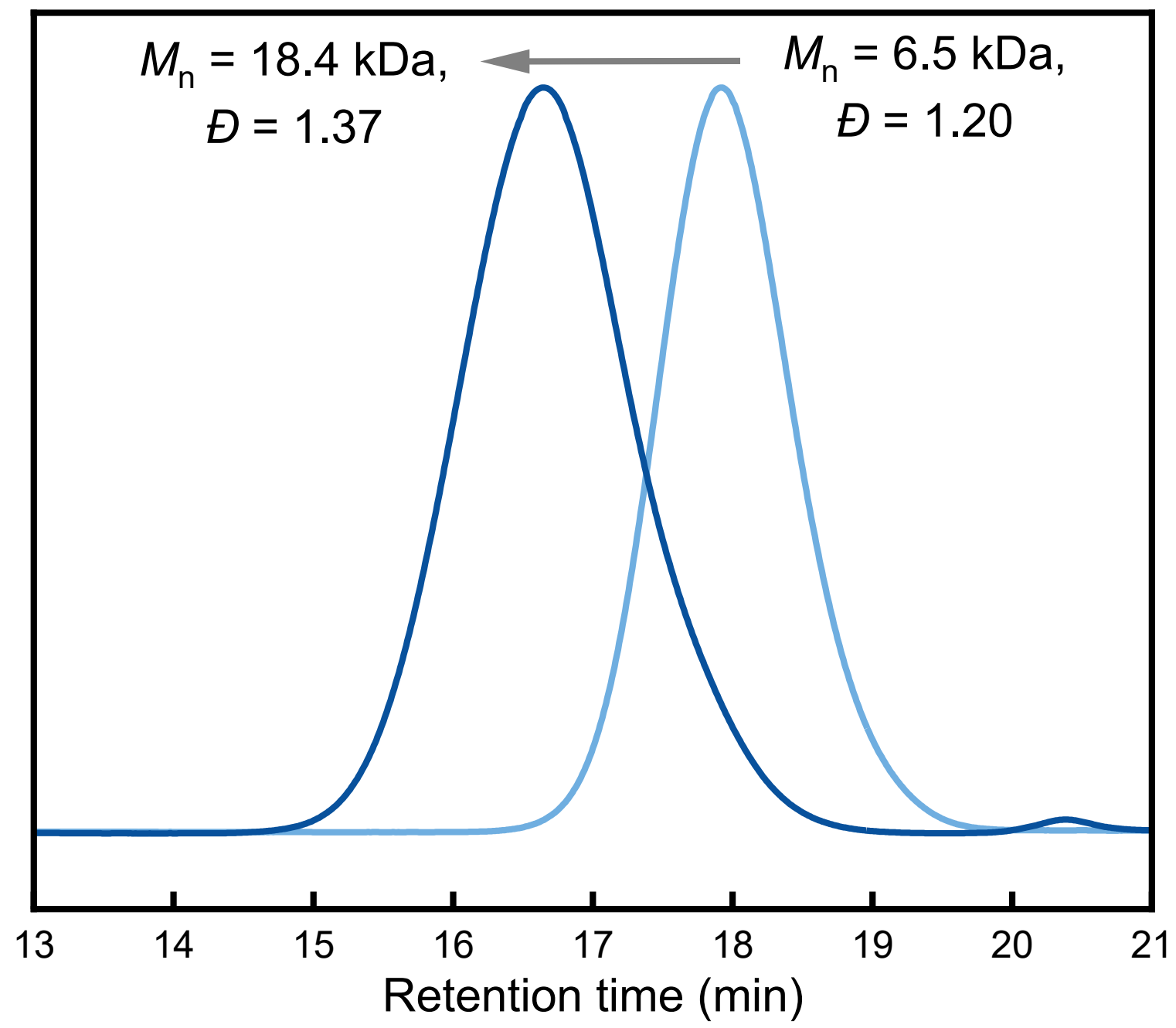

Figure S20. SEC trace (THF) of macroinitiator **P1** (M_n = 6.5 kDa, $Đ$ = 1.20) and diblock copolymer **P1-*b*-P15** (M_n = 18.4 kDa, $Đ$ = 1.37).

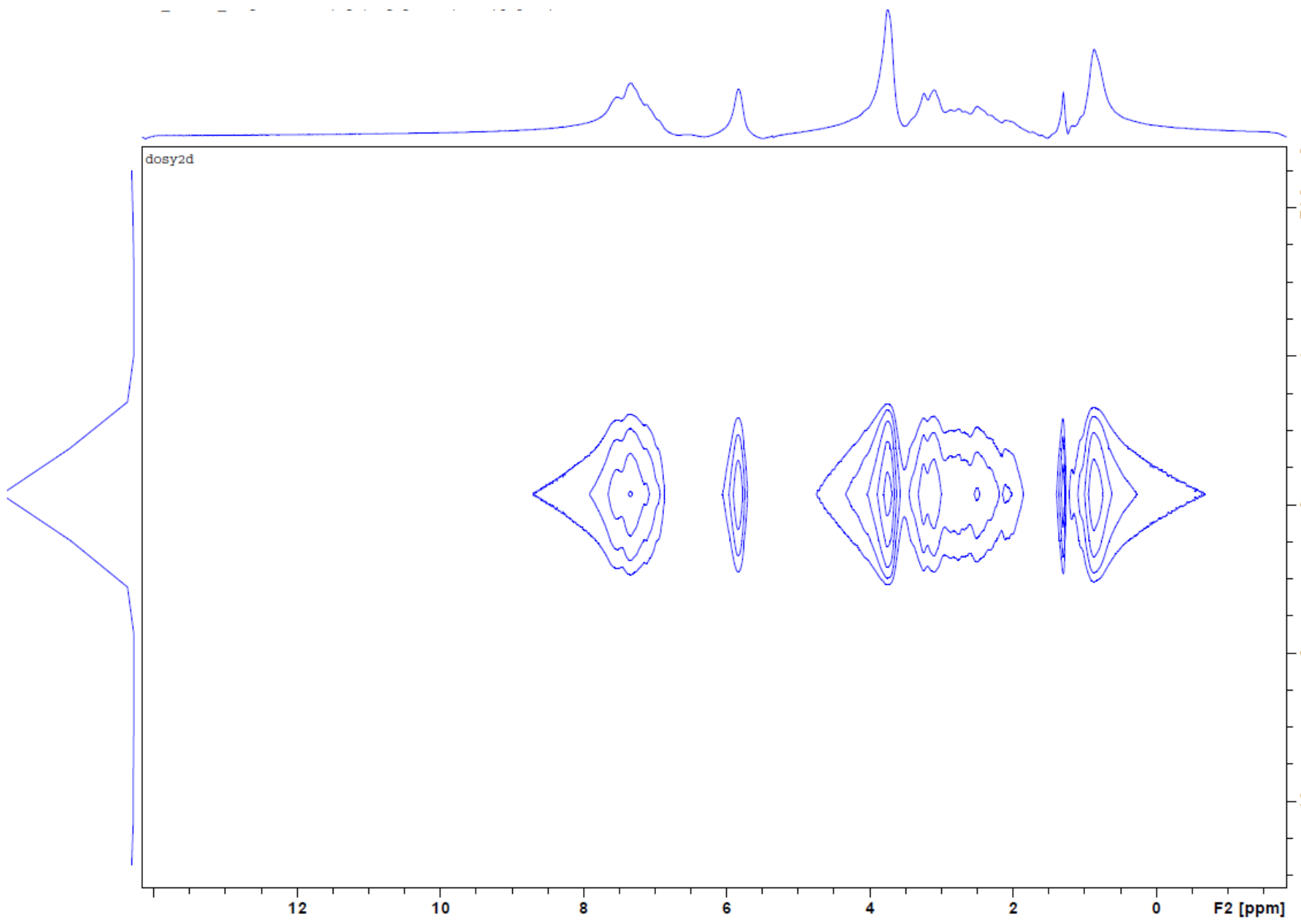

Figure S21. DOSY NMR spectrum of **P1-*b*-P15**.

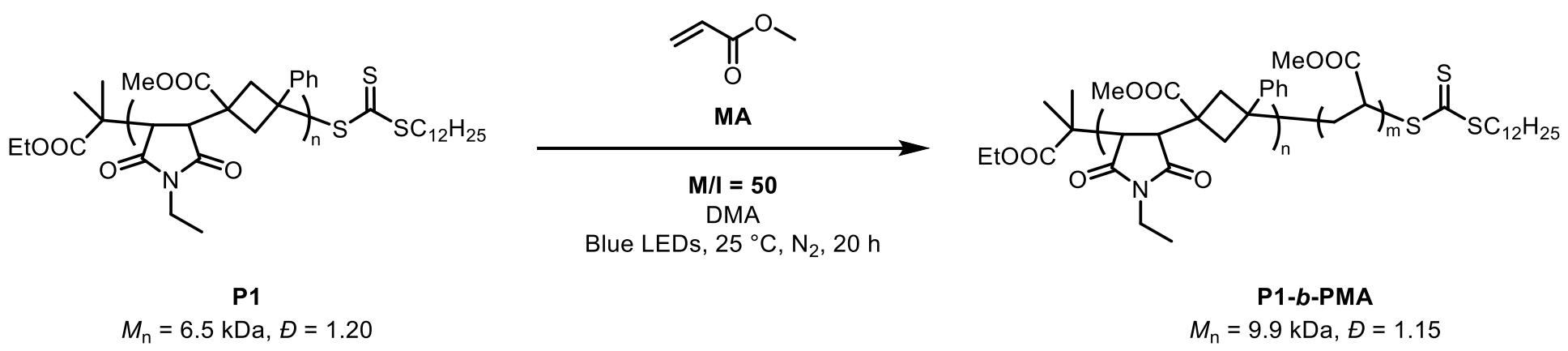

Scheme S17. Synthesis of diblock alternating copolymer **P1-*b*-PMA**.

P1 (M_n = 6.5 kDa, $Đ$ = 1.20) was prepared according to the GP-A with $[M]_0 : [CTA]_0$ = 25. In a nitrogen-filled glovebox, a 5 mL reaction vial equipped with a magnetic stirring bar was charged with **P1** (32, 0.1 mmol), methyl acrylate (MA, 0.1 mmol), followed by DMA (100 μL). The vial was sealed, removed from the glovebox, and stirred under 450 nm blue light irradiation at 25 °C for 20 h. Upon completion, the polymer solution was precipitated into pentane/Et_2O (v/v = 3:1) to give **P1-*b*-PMA** (M_n = 9.9 kDa, $Đ$ = 1.15) as a white powder.

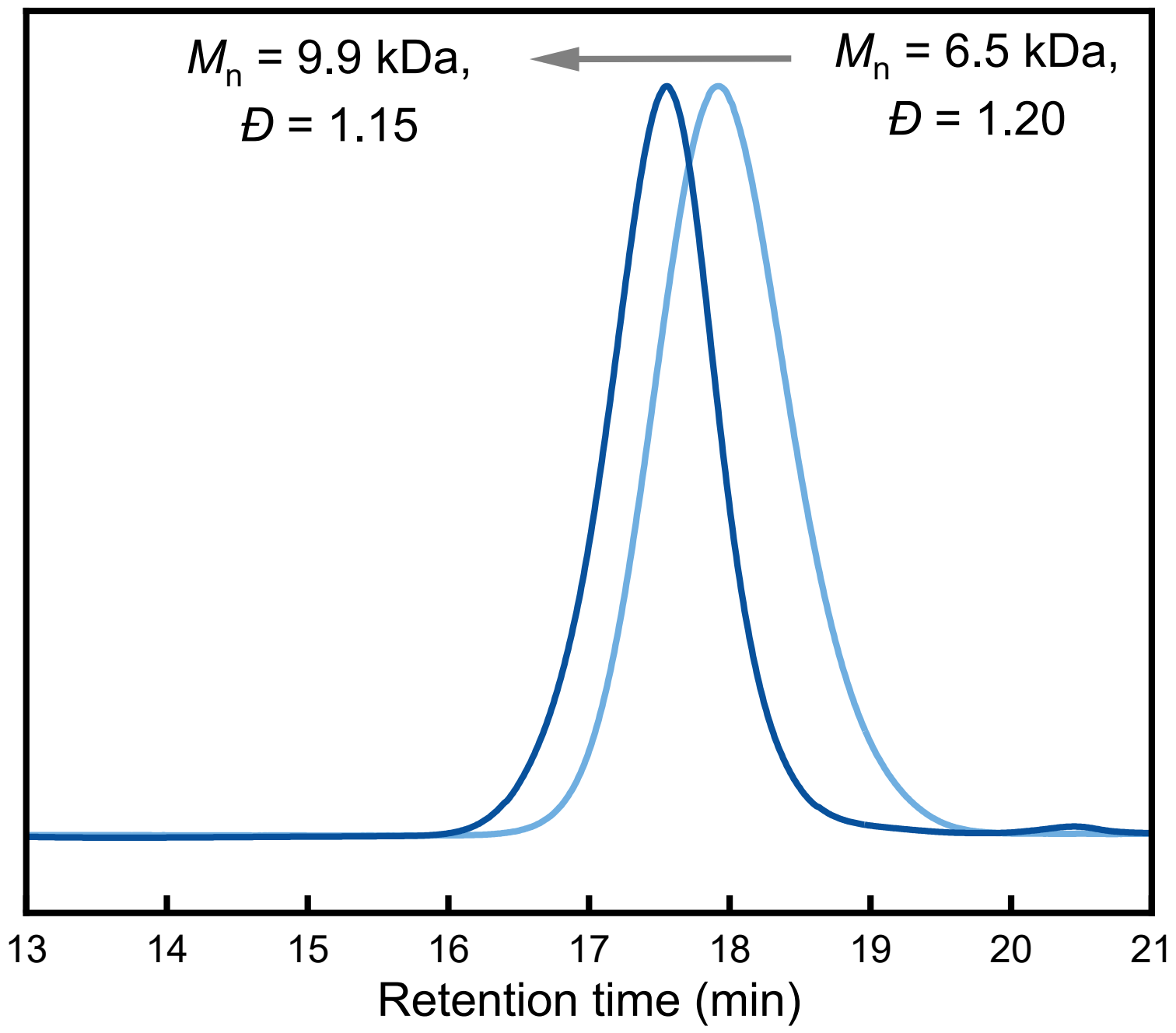

Figure S22. SEC trace (THF) of macroinitiator **P1** (M_n = 6.5 kDa, $Đ$ = 1.20) and diblock copolymer **P1-*b*-PMA** (M_n = 9.9 kDa, $Đ$ = 1.15).

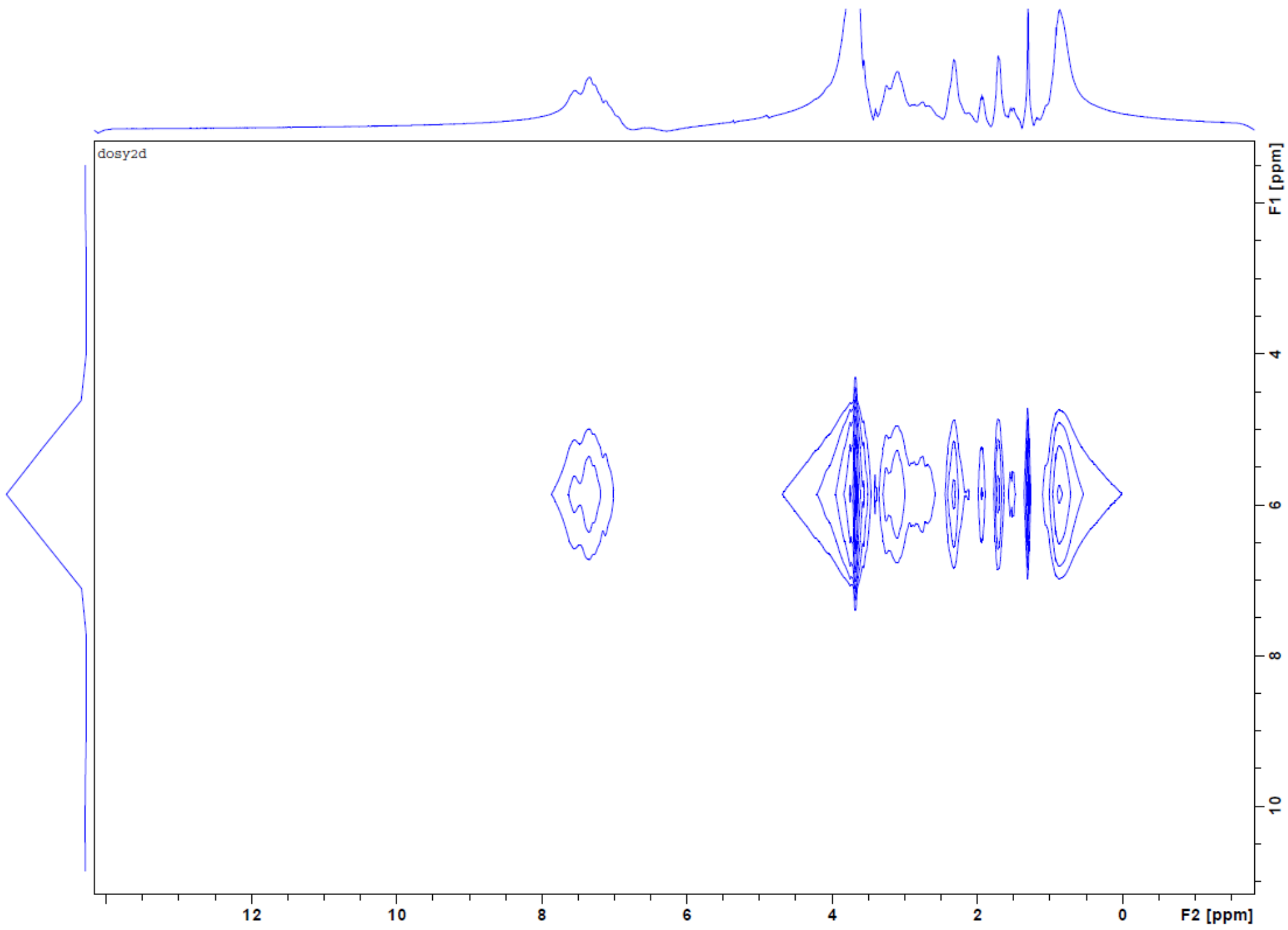

Figure S23. DOSY NMR spectrum of **P1-*b*-PMA**.

14. Mechanochemical Degradation via Ball-mill Grinding

P1 (5 mg, M_n = 115.0 kDa, $Đ$ = 1.81) or **P2** (5 mg, M_n = 118.1 kDa, $Đ$ = 1.75) or **P3** (5 mg, M_n = 63.5 kDa, $Đ$ = 1.68) or **P4** (5 mg, M_n = 80.2 kDa, $Đ$ = 1.55) was added to a 5 mL stainless steel screw cap jar containing stainless steel balls. The jar was then locked into the ball-mill machine, and milling was performed at 30 Hz for varying durations. At the designated time point, the entire contents of the jar (including the jar and balls) were collected and dissolved in THF. The molecular weight of the resulting fragments was determined by SEC. Note: for the sample obtained after 30 min of ball-milling, the entire contents of the jar (including the jar and balls) were dissolved in CD_2Cl_2 for NMR analysis. After removal of the solvent under reduced pressure, a portion of sample was redissolved in THF for SEC analysis, and another portion was used for MALDI-TOF MS characterization.

Table S7. Effect of ball-milling time on fragment molecular weight of P1

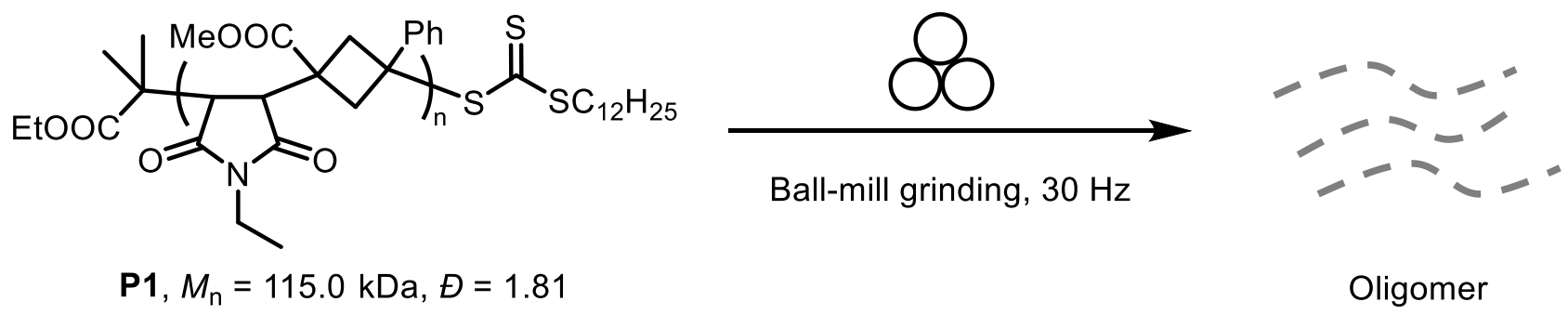

Entry	Time	$M_{n,SEC}$ (kDa)	$Đ$
1	0 min	115.0	1.18
2	2 min	2.87	2.16
3	5 min	1.98	1.71
4	10 min	1.61	1.59
5	15 min	1.36	1.58
6	20 min	1.14	1.69
7	25 min	0.99	1.60
8	30 min	0.99	1.60

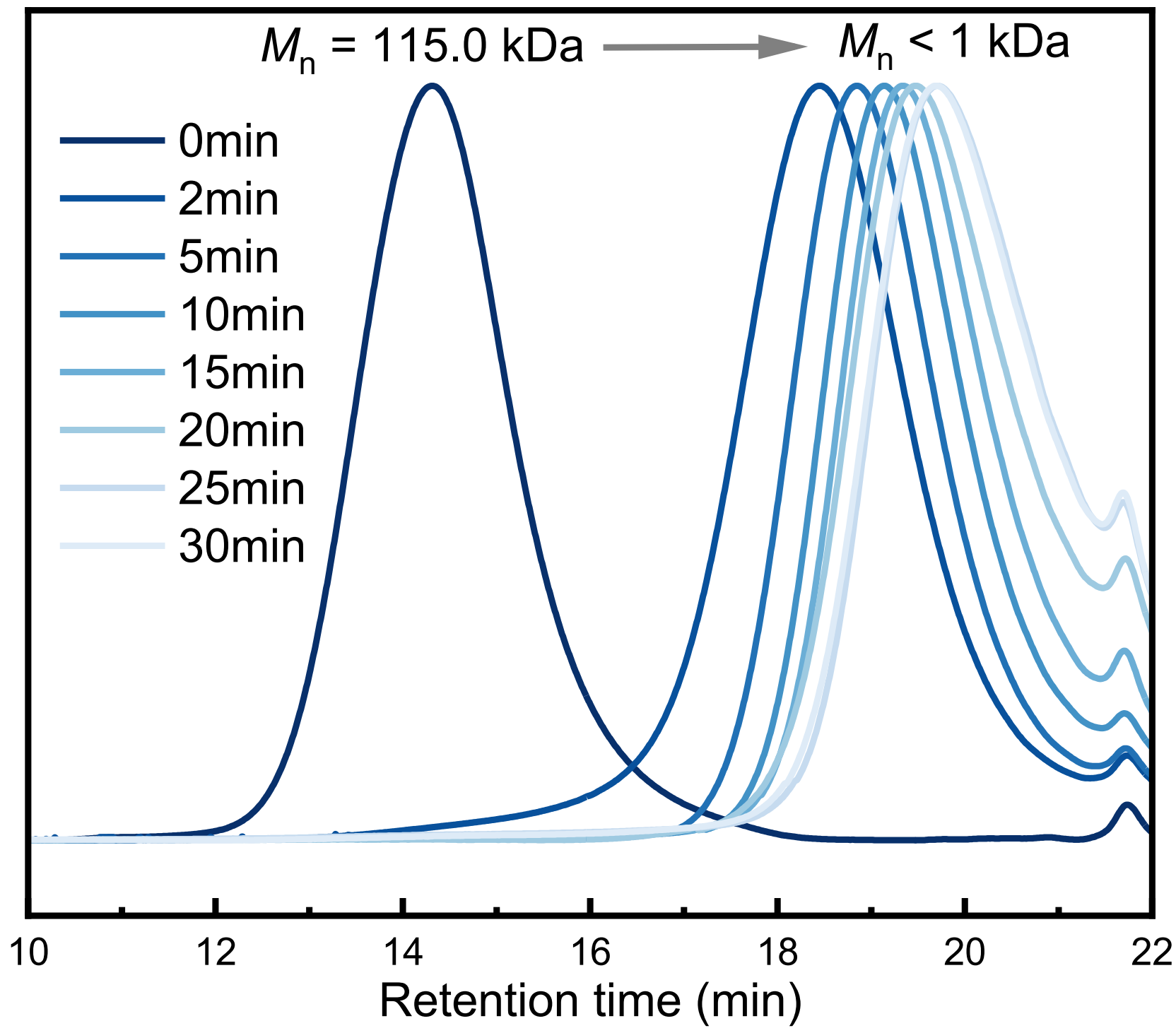

Figure S24. SEC trace (THF) of **P1** (M_n = 115.0 kDa, $Đ$ = 1.81) and oligomers during ball-mill grinding degradation.

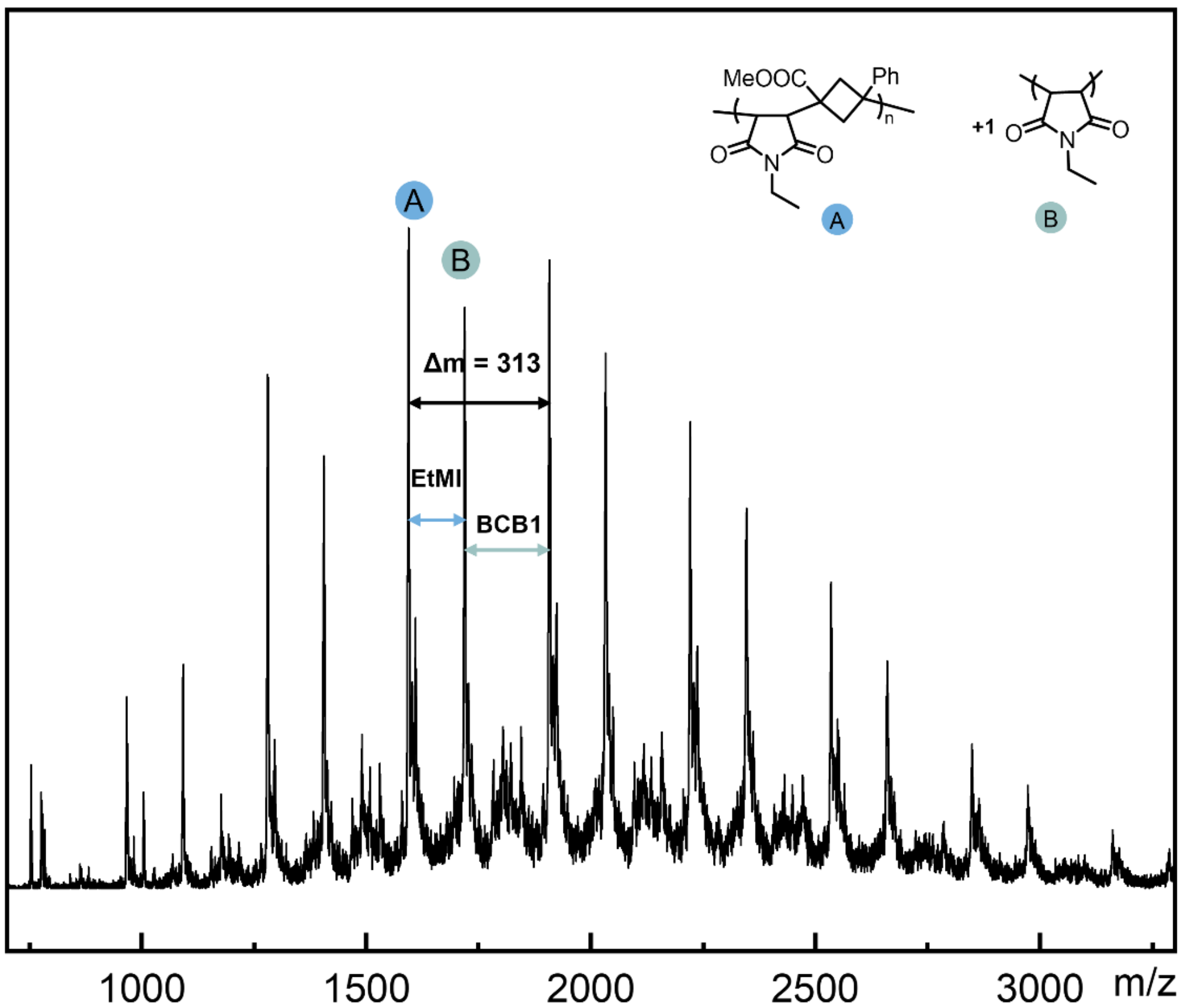

Figure S25. MALDI-TOF spectrum of oligomers after ball-mill grinding degradation.

Table S8. Effect of ball-milling time on fragment molecular weight of P2

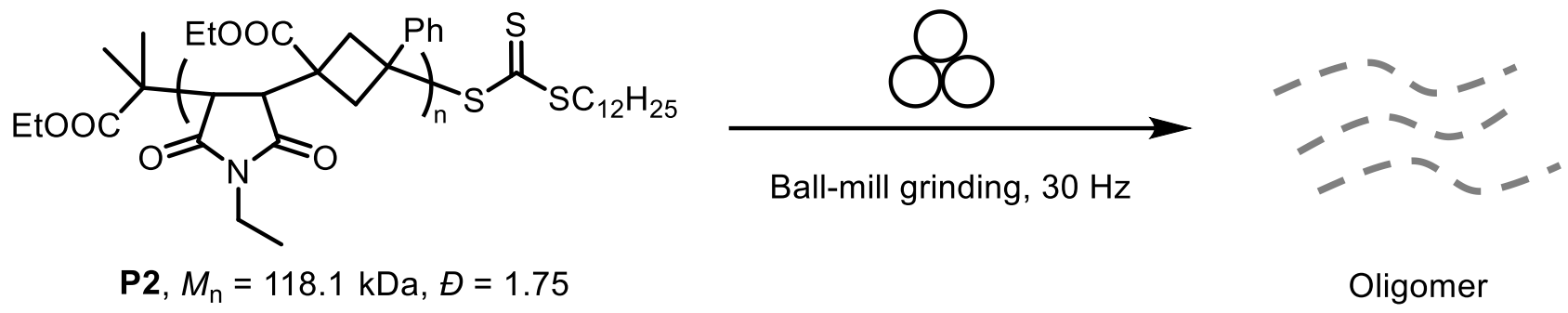

Entry	Time	$M_{n,SEC}$ (kDa)	*Đ*
1	0 min	118.1	1.75
2	2 min	3.04	2.38
3	5 min	1.95	1.74
4	10 min	1.54	1.65
5	20 min	1.32	1.65
6	30 min	1.13	1.66

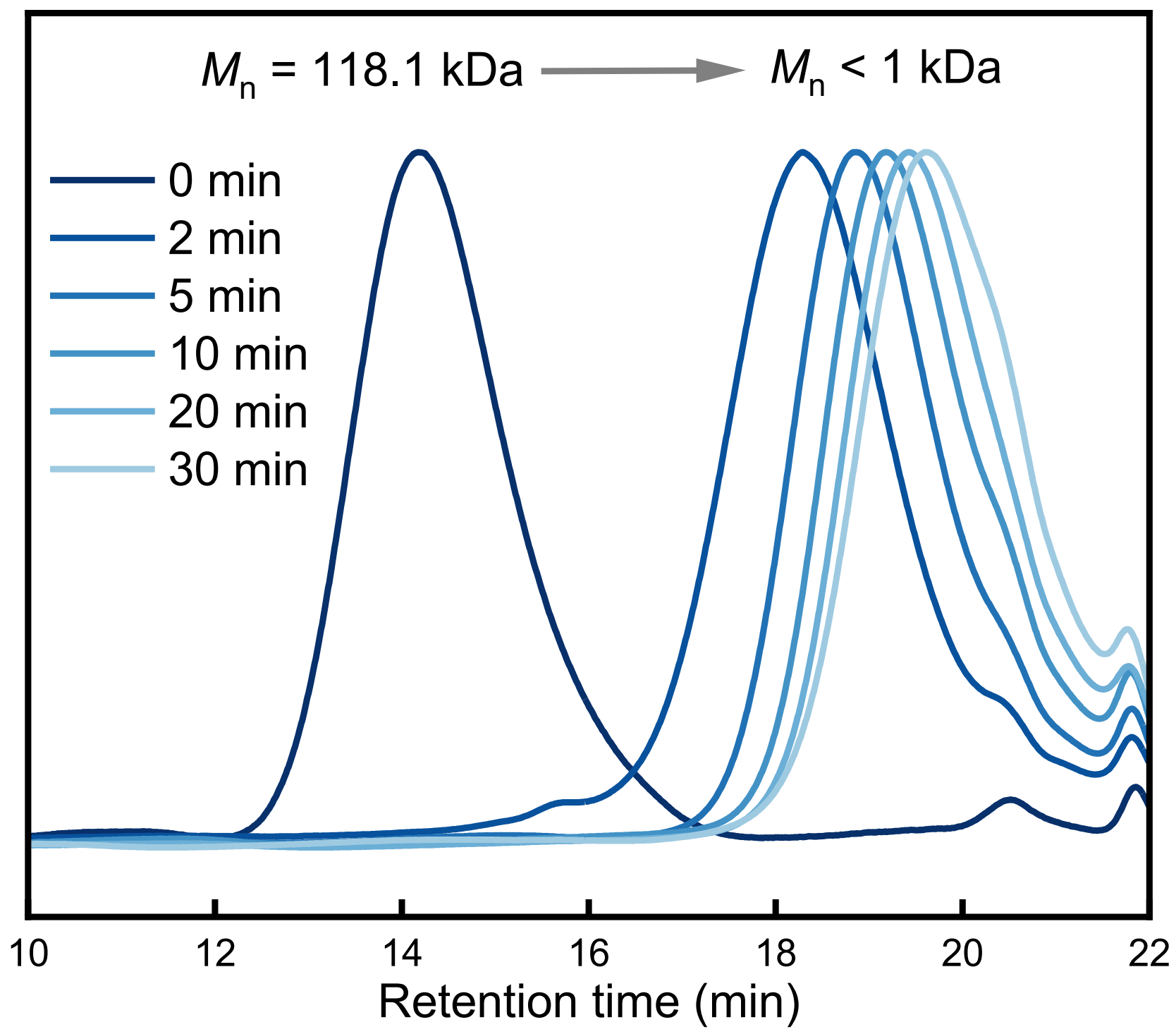

Figure S26. SEC trace (THF) of **P2** (5 mg, M_n = 118.1 kDa, *Đ* = 1.75) and oligomers during ball-mill grinding degradation.

Table S9. Effect of ball-milling time on fragment molecular weight of P3

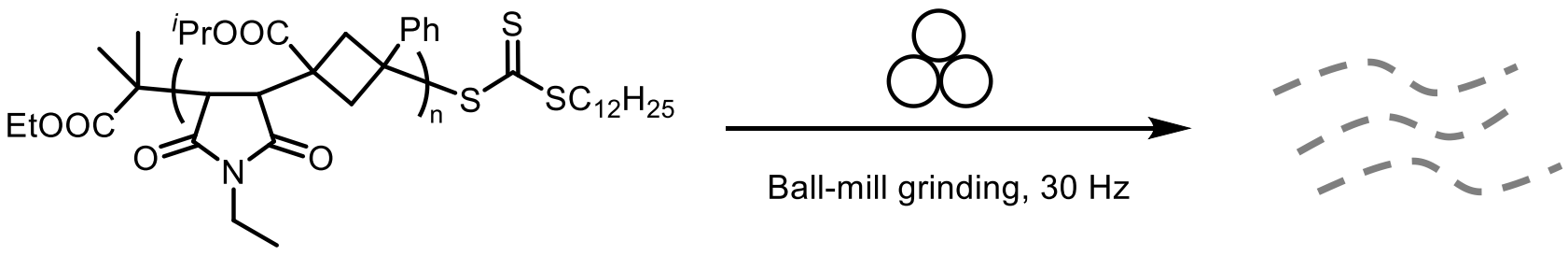

P3, M_n = 63.5 kDa, $Đ$ = 1.68 Oligomer

Entry	Time	$M_{n,SEC}$ (kDa)	$Đ$
1	0 min	63.5	1.68
2	2 min	3.31	2.12
3	5 min	1.77	1.73
4	10 min	1.30	1.64
5	20 min	1.02	1.62
6	30 min	0.84	1.66

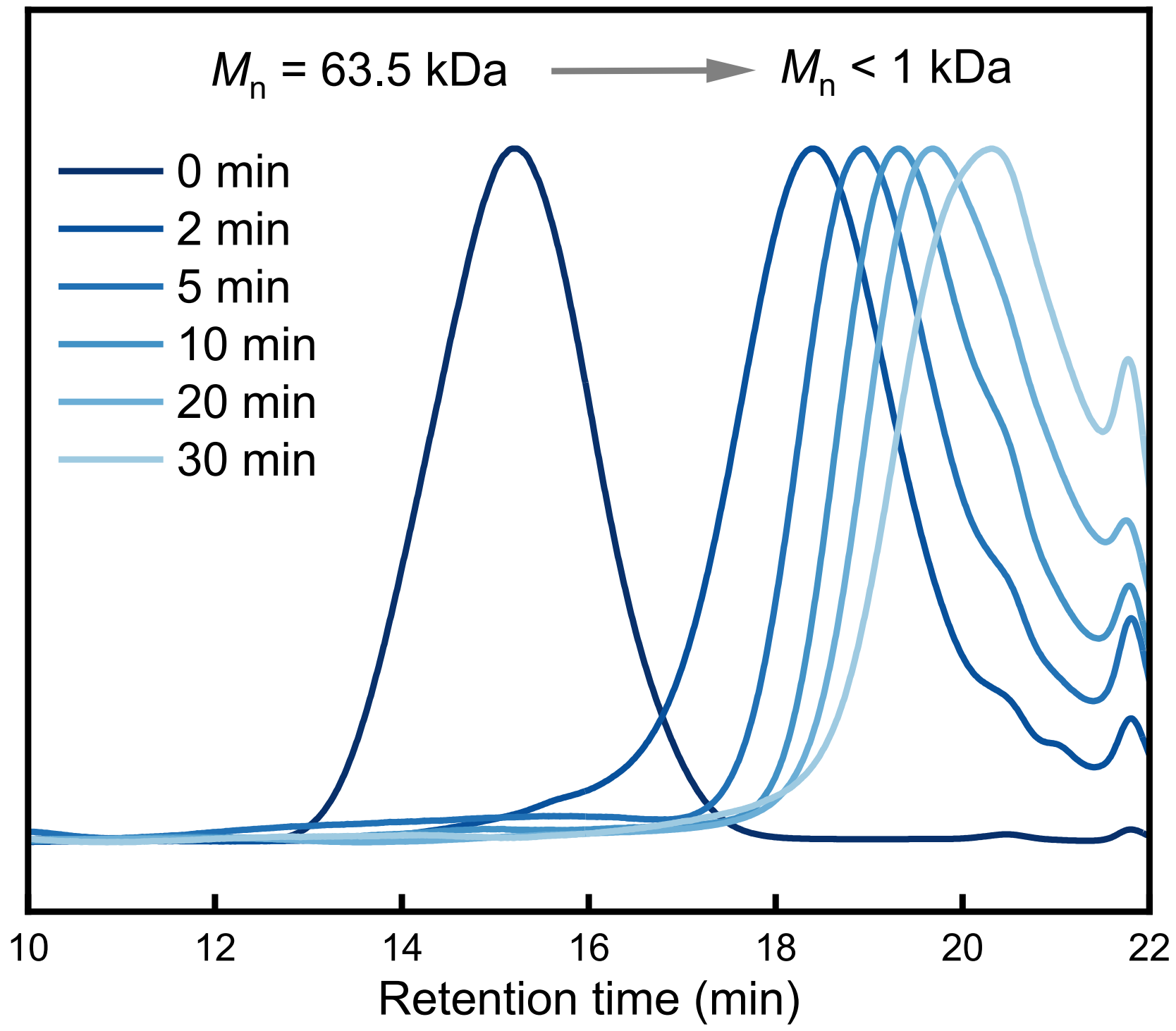

Figure S27. SEC trace (THF) of **P3** (5 mg, M_n = 63.5 kDa, $Đ$ = 1.68) and oligomers during ball-mill grinding degradation.

Table S10. Effect of ball-milling time on fragment molecular weight of P4

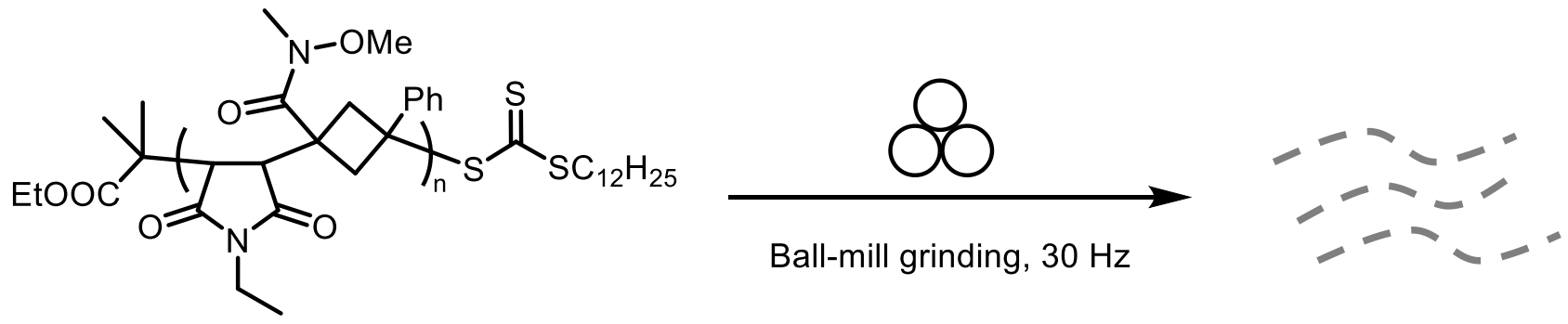

P4, M_n = 80.2 kDa, $Đ$ = 1.55

Oligomer

Entry	Time	$M_{n,SEC}$ (kDa)	$Đ$
1	0 min	80.2	1.55
2	2 min	2.50	1.81
3	5 min	1.67	1.67
4	10 min	1.35	1.61
5	20 min	1.09	1.53
6	30 min	0.98	1.51

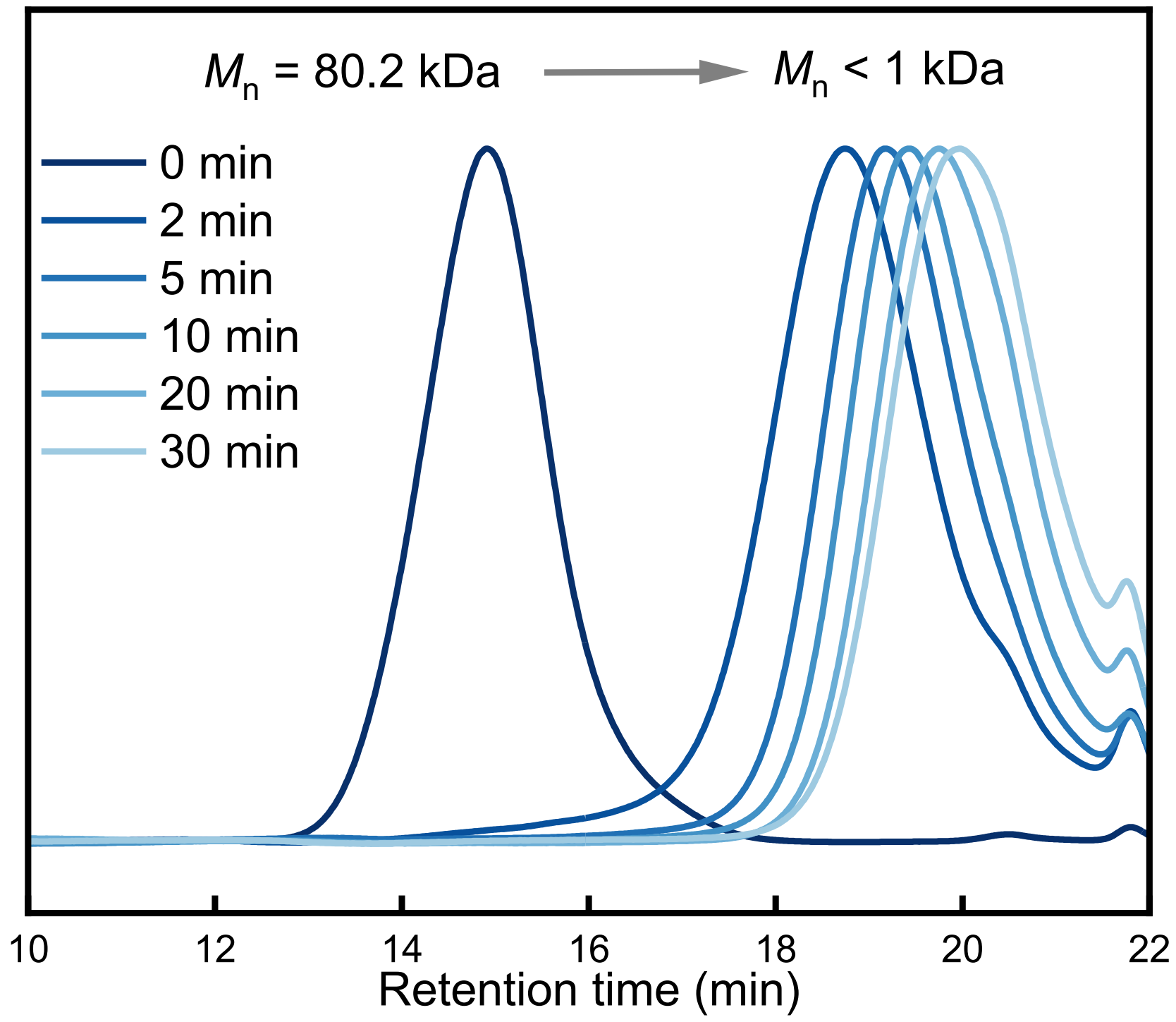

Figure S28. SEC trace (THF) of **P4** (5 mg, M_n = 80.2 kDa, $Đ$ = 1.55) and oligomers during ball-mill grinding degradation.

15. Computational Details

All density functional theory (DFT) calculations were performed using the Gaussian 09 suite of programs.[9] Geometry optimizations were carried out using the B3LYP functional[10–12] augmented with Grimme's D3 dispersion correction (B3LYP-D3)[13,14] and the Pople's 6-31G(d,p) basis set.[15,16] Vibrational frequency calculations were conducted at the same level of theory to confirm that all optimized structures are true local minima on the potential energy surface, characterized by the absence of imaginary frequencies.

To evaluate the mechanical strength of the polymer backbone, relaxed potential energy surface (PES) scans were performed at the identical level of theory. The central bond of the tetramer model was systematically elongated in increments of 0.02 Å over 75 steps. The restoring force at each elongation step was then mathematically derived by calculating the first derivative of the potential energy with respect to the bond distance.

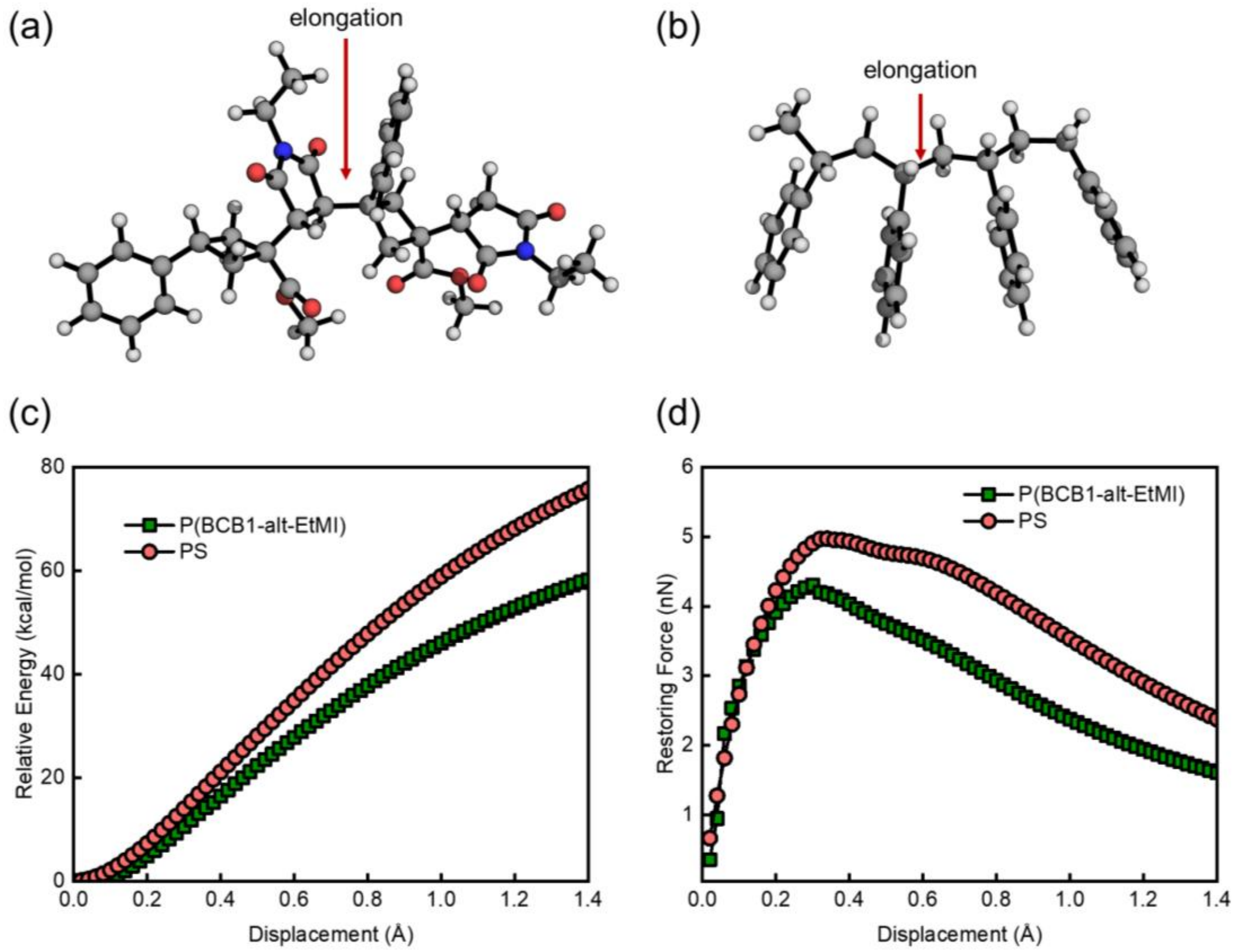

Figure S29. DFT-based evaluation of the intrinsic mechanical strength of **P(BCB1-*alt*-EtMI)** and polystyrene backbones. (a, b) Optimized tetramer models of (a) **P(BCB1-*alt*-EtMI)** and (b) polystyrene, with the target central bonds highlighted in red. (c) Relative potential energy profiles obtained from relaxed PES scans as a function of bond length. (d) Restoring force curves derived from the energy gradients. The maximum restoring forces (F_{max}) reveal that **P(BCB1-*alt*-EtMI)** and polystyrene cleave at critical forces of 4.31 nN and 4.97 nN, respectively.

Appendix

Cartesian Coordinate

P(BCB1-*alt*-EtMI) Model; E= -2105.16891174 Hartree

Element	X (Å)	Y (Å)	Z (Å)	Element	X (Å)	Y (Å)	Z (Å)
N	-2.009214	2.482274	-0.936232	H	6.663764	-0.211433	3.226957
C	-2.318183	1.984445	0.321534	H	7.947309	0.280538	2.081451
C	-1.121878	1.684377	-1.638722	C	4.893478	0.274194	-1.134448
O	-3.009519	2.559850	1.124104	H	4.717288	-0.229810	-2.095002
O	-0.722355	1.918196	-2.752473	H	5.019683	1.345341	-1.344144
C	-2.533720	3.739446	-1.438044	C	1.111819	1.988260	0.229914
H	-2.638848	3.629640	-2.526583	C	1.001098	2.196967	1.610946
H	-3.532931	3.863840	-0.997506	C	1.485004	3.075442	-0.571370

C	-1.644801	4.926042	-1.096507	C	1.249337	3.448005	2.172586
H	-0.663762	4.828886	-1.580852	H	0.726563	1.369509	2.268263
H	-2.112462	5.858797	-1.443560	C	1.746532	4.324249	-0.011430
H	-1.490733	4.996630	-0.010023	H	1.554949	2.951307	-1.653664
C	-1.688314	0.604127	0.449896	C	1.627457	4.517156	1.363198
H	-1.178093	0.538623	1.420624	H	1.152106	3.583120	3.251718
C	-0.717805	0.525145	-0.740941	H	2.038338	5.154732	-0.658125
H	-0.855450	-0.406129	-1.311356	H	1.830292	5.496030	1.802532
C	0.786802	0.631419	-0.387112	C	-6.192540	-0.844595	0.385983
C	1.350595	-0.572843	0.414475	C	-7.434073	-0.203837	0.440710
C	1.727192	0.219122	-1.552670	C	-6.171081	-2.245445	0.336271
C	2.530402	-0.695035	-0.586760	C	-8.621297	-0.936290	0.445383
H	1.641200	-0.418441	1.459983	H	-7.470849	0.888096	0.481484
H	0.695451	-1.449075	0.368046	C	-7.352964	-2.981086	0.341114
H	2.300949	1.007237	-2.056737	H	-5.213075	-2.772247	0.291760
H	1.192992	-0.362327	-2.314184	C	-8.584798	-2.327856	0.395521
C	-2.761683	-0.511978	0.432026	H	-9.580138	-0.414651	0.488441
C	-3.876056	-0.366947	1.505409	H	-7.313314	-4.072072	0.301938
C	-3.840752	-0.403691	-0.674908	H	-9.512823	-2.903663	0.399000
C	-4.911689	-0.052772	0.392781	C	2.711151	-2.108477	-1.106822
H	-4.074339	-1.308217	2.036770	O	1.801803	-2.813832	-1.464525
H	-3.718211	0.432626	2.239412	O	3.985067	-2.503775	-1.138552
H	-4.022806	-1.360441	-1.177993	C	-2.008071	-1.823421	0.550373
H	-3.672037	0.356523	-1.451155	O	-1.362678	-2.124606	1.522770
H	-5.146311	1.020488	0.383776	O	-2.108173	-2.589076	-0.534822
C	4.534161	-0.833071	0.985378	C	3.815182	-0.024034	-0.087946
C	6.182047	-0.271062	-0.548106	H	3.511727	0.913555	0.405048
N	5.881375	-0.864983	0.671201	C	-1.355497	-3.800024	-0.548388
O	4.044296	-1.349524	1.958463	H	-1.756522	-4.395623	-1.375764
O	7.284806	-0.223313	-1.027621	H	-0.294092	-3.577747	-0.728615
C	6.874184	-1.515681	1.505090	H	-1.467348	-4.334715	0.404030
H	6.382004	-2.376243	1.980138	C	4.241022	-3.831784	-1.572676
H	7.663034	-1.882981	0.833769	H	3.752311	-4.554270	-0.904447
C	7.453890	-0.579795	2.556597	H	5.328482	-3.957315	-1.540455
H	8.200406	-1.109149	3.166207	H	3.867273	-3.983378	-2.594722

Poly(styrene) model; E = -1240.02731559 Hartree.

Element	X (Å)	Y (Å)	Z (Å)	Element	X (Å)	Y (Å)	Z (Å)
C	-4.675797	2.873269	1.095479	C	-1.414623	-2.421094	0.992011
H	-4.207643	3.645398	1.715814	H	-0.978081	-0.708467	2.216628
H	-5.591408	2.539292	1.592771	C	-1.834676	-1.966046	-1.337083
H	-4.964076	3.337115	0.145840	H	-1.747119	0.094303	-1.926533
C	-3.711302	1.693059	0.864053	C	-1.719827	-2.881000	-0.288241
H	-3.414518	1.311012	1.850061	H	-1.312645	-3.122648	1.815001

C	-2.426548	2.177754	0.146156	H	-2.086349	-2.310820	-2.335771
H	-2.224533	3.212165	0.452728	H	-1.868214	-3.941642	-0.469142
H	-2.602031	2.215563	-0.936301	C	1.764028	0.330448	0.616496
C	-1.145392	1.357519	0.434862	C	1.956957	-0.063245	1.943899
H	-0.927832	1.478904	1.505388	C	1.872660	-0.640179	-0.388109
C	0.044782	1.954751	-0.359166	C	2.251523	-1.389765	2.266783
H	-0.119866	3.032620	-0.487746	H	1.875690	0.678372	2.735276
H	0.053109	1.530010	-1.370510	C	2.165586	-1.963114	-0.073916
C	1.439906	1.776356	0.284271	H	1.724392	-0.357588	-1.425609
H	1.425957	2.328297	1.235402	C	2.356711	-2.344095	1.256984
C	2.506675	2.448800	-0.610427	H	2.397802	-1.674178	3.305226
H	2.213418	3.497967	-0.743158	H	2.244433	-2.700023	-0.867215
H	2.478963	1.997781	-1.610302	H	2.585079	-3.377408	1.501214
C	-4.424682	0.563256	0.131528	C	3.956887	2.403715	-0.074366
C	-4.627271	-0.678078	0.743162	H	4.511698	3.231720	-0.533854
C	-4.911033	0.745845	-1.170980	H	3.951437	2.600823	1.004376
C	-5.271637	-1.716131	0.070171	C	4.701069	1.112984	-0.357620
H	-4.242109	-0.845269	1.744525	C	5.105132	0.256188	0.671198
C	-5.560525	-0.285727	-1.847362	C	4.990529	0.745739	-1.679357
H	-4.773366	1.703042	-1.667423	C	5.754142	-0.946342	0.390976
C	-5.740234	-1.524993	-1.229660	H	4.876015	0.516123	1.699979
H	-5.396973	-2.677633	0.559634	C	5.642260	-0.452800	-1.966040
H	-5.924441	-0.123004	-2.858223	H	4.695795	1.404810	-2.492975
H	-6.238666	-2.332929	-1.757473	C	6.022927	-1.307589	-0.929388
C	-1.335345	-0.130881	0.178337	H	6.039614	-1.605250	1.205853
C	-1.232436	-1.056945	1.220215	H	5.853811	-0.719651	-2.997876
C	-1.639148	-0.607248	-1.104182	H	6.524851	-2.245291	-1.149706

Vibration Modes (top 20)

P(BCB1-*alt*-EtMI)			PS		
Mode	Frequency (cm^{-1})	IR Intensity (KM/Mole)	Mode	Frequency (cm^{-1})	IR Intensity (KM/Mole)
1	10.8887	0.0285	1	16.5220	0.0443
2	12.1538	0.0391	2	22.0580	0.0112
3	14.8875	0.0709	3	28.9870	0.0085
4	19.8520	0.0452	4	34.8333	0.0006
5	22.2526	0.0544	5	49.1173	0.1002
6	27.3946	0.0807	6	54.0395	0.0267
7	31.8144	0.2044	7	61.6228	0.0047
8	37.0769	1.3931	8	68.3666	0.0585
9	38.4685	0.4757	9	74.1358	0.1093
10	40.3389	0.1754	10	79.0818	0.3983
11	52.9894	0.3236	11	92.5681	0.0016
12	55.8240	0.2911	12	94.8437	0.3020

13	61.4838	0.1764	13	104.0032	0.2217
14	67.1829	4.3038	14	129.8067	0.0315
15	79.1939	0.1019	15	159.7909	0.0475
16	79.7570	0.3599	16	182.2876	0.0574
17	94.8491	1.3540	17	195.3400	0.0709
18	103.3919	0.8210	18	218.7664	0.0239
19	104.1898	0.8616	19	220.7375	0.0736
20	108.3413	5.1574	20	240.1441	0.2686

16. References

[1] S. L. Lin, Y. H. Chen, H. H. Liu, S. H. Xiang, B. Tan, Enantioselective synthesis of chiral cyclobutenes enabled by brønsted acid-catalyzed isomerization of BCBs. *J. Am. Chem. Soc.* **2023**, *145*, 21152–21158.

[2] S. Dutta, C. G. Daniliuc, C. Muck-Lichtenfeld, A. Studer, Enantioselective synthesis of tetrahydro-1H-1,3-methanocarbazoles by formal (3+3)-cycloaddition using bicyclo[1.1.0]butanes. *J. Am. Chem. Soc.* **2025**, *147*, 4249–4257.

[3] J. L. Tyler, F. Schafer, H. Shao, C. Stein, A. Wong, C. G. Daniliuc, K. N. Houk, F. Glorius, Bicyclo[1.1.0]butyl radical cations: synthesis and application to [2π + 2σ] cycloaddition reactions. *J. Am. Chem. Soc.* **2024**, *146*, 16237–16247.

[4] K. Li, Y. Shen, D.-F. Chen, Visible light-driven organocatalyzed radical ring-opening polymerization of bicyclo[1.1.0]butanes. *Polymer* **2024**, *308*, 127331–127337.

[5] Y. C. Chang, M. Martin, K. Bortey, Q. Lefebvre, T. Fessard, C. Salome, R. J. Vazquez, M. K. Brown, Catalyst-controlled regiodivergent synthesis of bicyclo[2.1.1]hexanes via photochemical strain-release cycloadditions. *J. Am. Chem. Soc.* **2025**, *147*, 14936–14944.

[6] Y. Zhang, D. Riemer, W. Schilling, J. Kollmann, S. Das, Visible-Light-Mediated Efficient Metal-Free Catalyst for α-Oxygenation of Tertiary Amines to Amides. *ACS Catalysis* **2018**, *8*, 6659–6664.

[7] Y. Gu, Z. Zhang, T. Gao, R. Gomez-Bombarelli, M. Chen, Low-Dispersity Polymers via Free Radical Alternating Copolymerization: Effects of Charge-Transfer-Complexes, *Angew. Chem. In. Ed.* **2024**, *63*, e202409744.

[8] S. Beuermann, N. García, A Novel Approach to the Understanding of the Solvent Effects in Radical Polymerization Propagation Kinetics, *Macromolecules* **2004**, *37*, 3018–3025.

[9] M. J. Frisch, G. W. Trucks, H. B. Schlegel, G. E. Scuseria, M. A. Robb, J. R. Cheeseman, G. Scalmani, V. Barone, B. Mennucci, G. A. Petersson, H. Nakatsuji, M. Caricato, X. Li, H. P. Hratchian, A. F. Izmaylov, J. Bloino, G. Zheng, J. L. Sonnenberg, M. Hada, M. Ehara, K. Toyota, R. Fukuda, J. Hasegawa, M. Ishida, T. Nakajima, Y. Honda, O. Kitao, H. Nakai, T. Vreven, J. A. Montgomery Jr., J. E. Peralta, F. Ogliaro, M. Bearpark, J. J. Heyd, E. Brothers, K. N. Kudin, V. N. Staroverov, R. Kobayashi, J. Normand, K. Raghavachari, A. Rendell, J. C. Burant, S. S. Iyengar, J. Tomasi, M. Cossi, N. Rega, J. M. Millam, M. Klene, J. E. Knox, J. B. Cross, V. Bakken, C. Adamo, J. Jaramillo, R. Gomperts, R. E. Stratmann, O. Yazyev, A. J. Austin, R. Cammi, C. Pomelli, J. W. Ochterski, R. L. Martin, K. Morokuma, V. G. Zakrzewski, G. A. Voth, P. Salvador, J. J. Dannenberg, S. Dapprich, A. D. Daniels, Ö. Farkas, J. B. Foresman, J. V. Ortiz, J. Cioslowski, D. J. Fox, Gaussian 09, Revision D.01, Gaussian, Inc., Wallingford CT, **2009**.

[10] A. D. Becke, Density-functional thermochemistry. III. The role of exact exchange. *J. Chem. Phys.* **1993**, *98*, 5648–5652.

[11] C. Lee, W. Yang, R. G. Parr, *Phys. Rev. B* Development of the Colle-Salvetti correlation-energy formula into a functional of the electron density. **1988**, *37*, 785–789.

[12] P. J. Stephens, F. J. Devlin, C. F. Chabalowski, M. J. Frisch, Ab Initio Calculation of Vibrational Absorption and Circular Dichroism Spectra Using Density Functional Force Fields. *J. Phys. Chem.* **1994**, *98*, 11623–11627.

[13] S. Grimme, J. Antony, S. Ehrlich, H. Krieg, A consistent and accurate ab initio parametrization of density functional dispersion correction (DFT-D) for the 94 elements H-Pu. *J. Chem. Phys.* **2010**, *132*, 154104.

[14] S. Grimme, S. Ehrlich, L. Goerigk, Effect of the damping function in dispersion corrected density functional theory. *J. Comput. Chem.* **2011**, *32*, 1456–1465.
[15] W. J. Hehre, R. Ditchfield, J. A. Pople, Self—Consistent Molecular Orbital Methods. XII. Further Extensions of Gaussian—Type Basis Sets for Use in Molecular Orbital Studies of Organic Molecules. *J. Chem. Phys.* **1972**, *56*, 2257–2261.
[16] P. C. Hariharan, J. A. Pople, Influence of Polarization Functions on Molecular-Orbital Hydrogenation Energies. *Theor. Chim. Acta* **1973**, *28*, 213–222.

17. NMR spectra

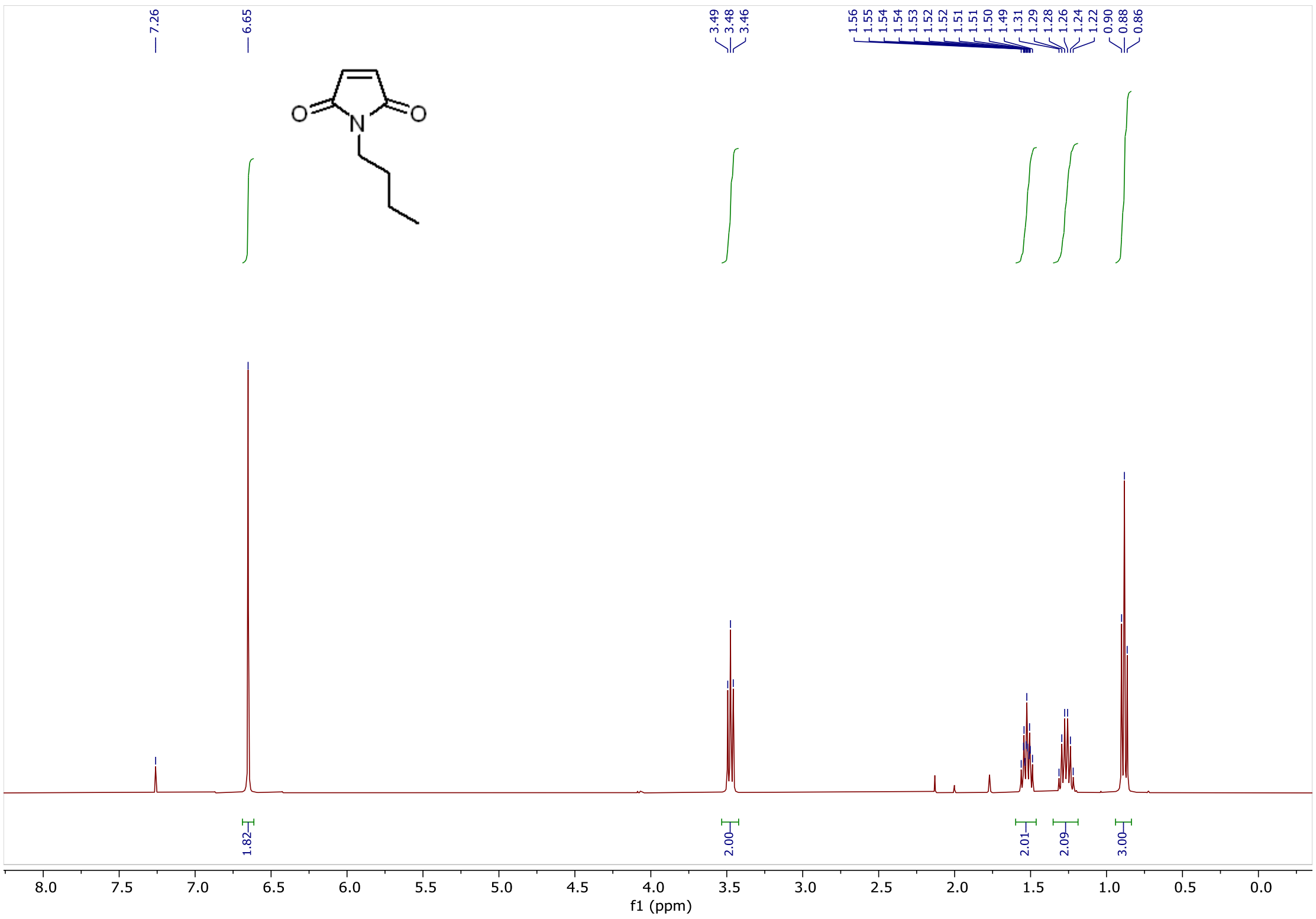

Figure S30. ^{1}H NMR spectrum of **S9**.

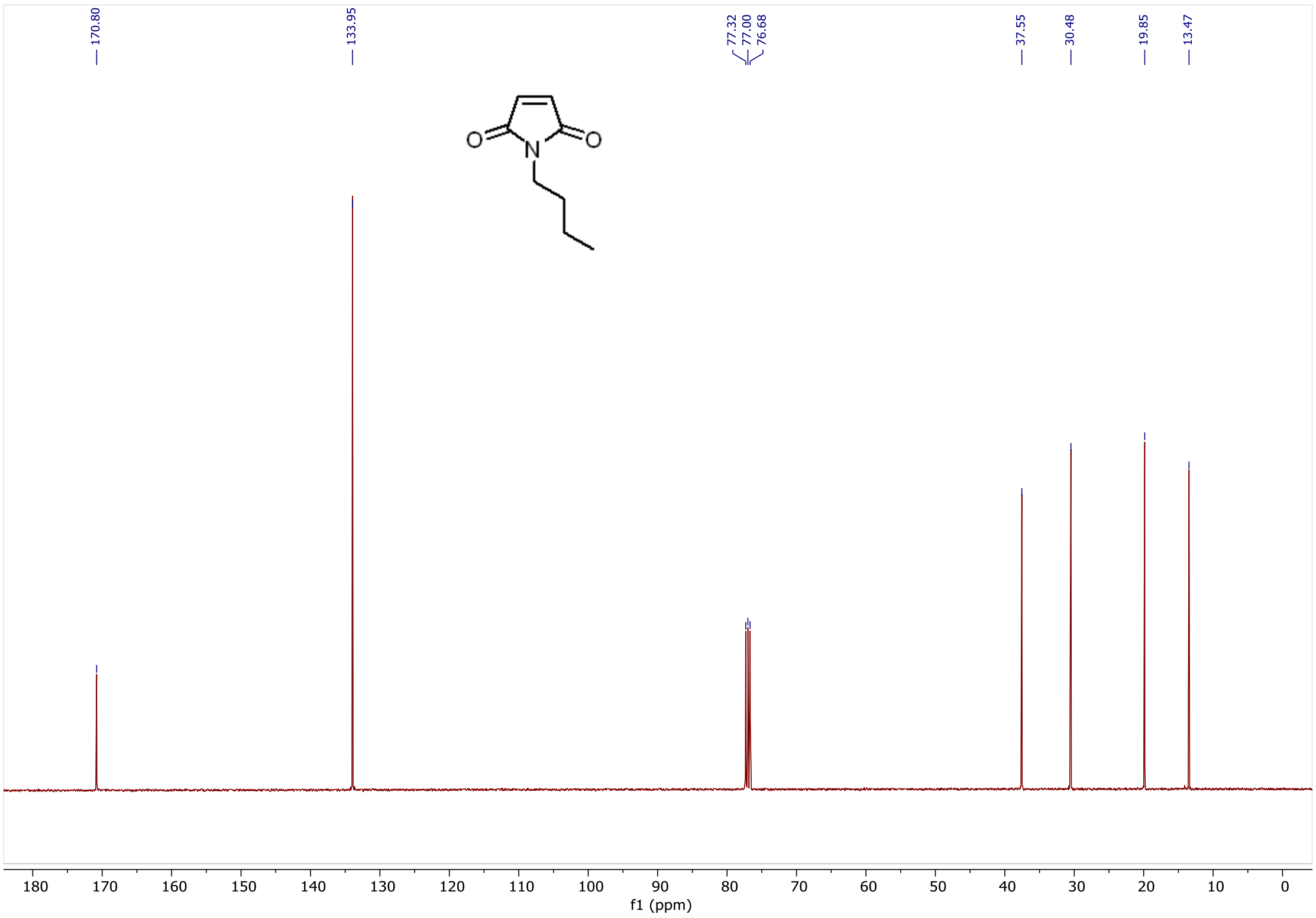

Figure S31. ^{13}C NMR spectrum of **S9**.

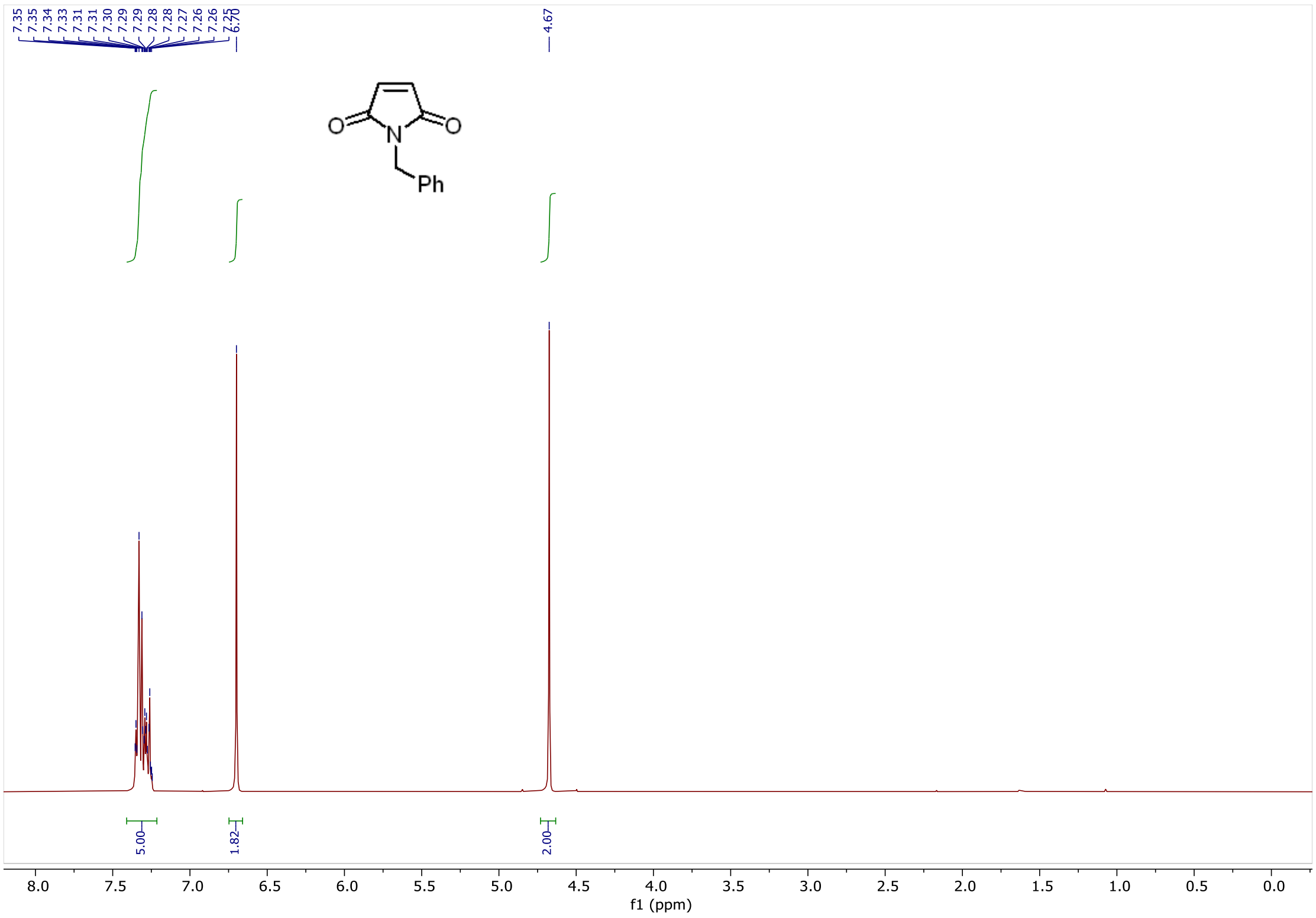

Figure S32. ^{1}H NMR spectrum of **S10**.

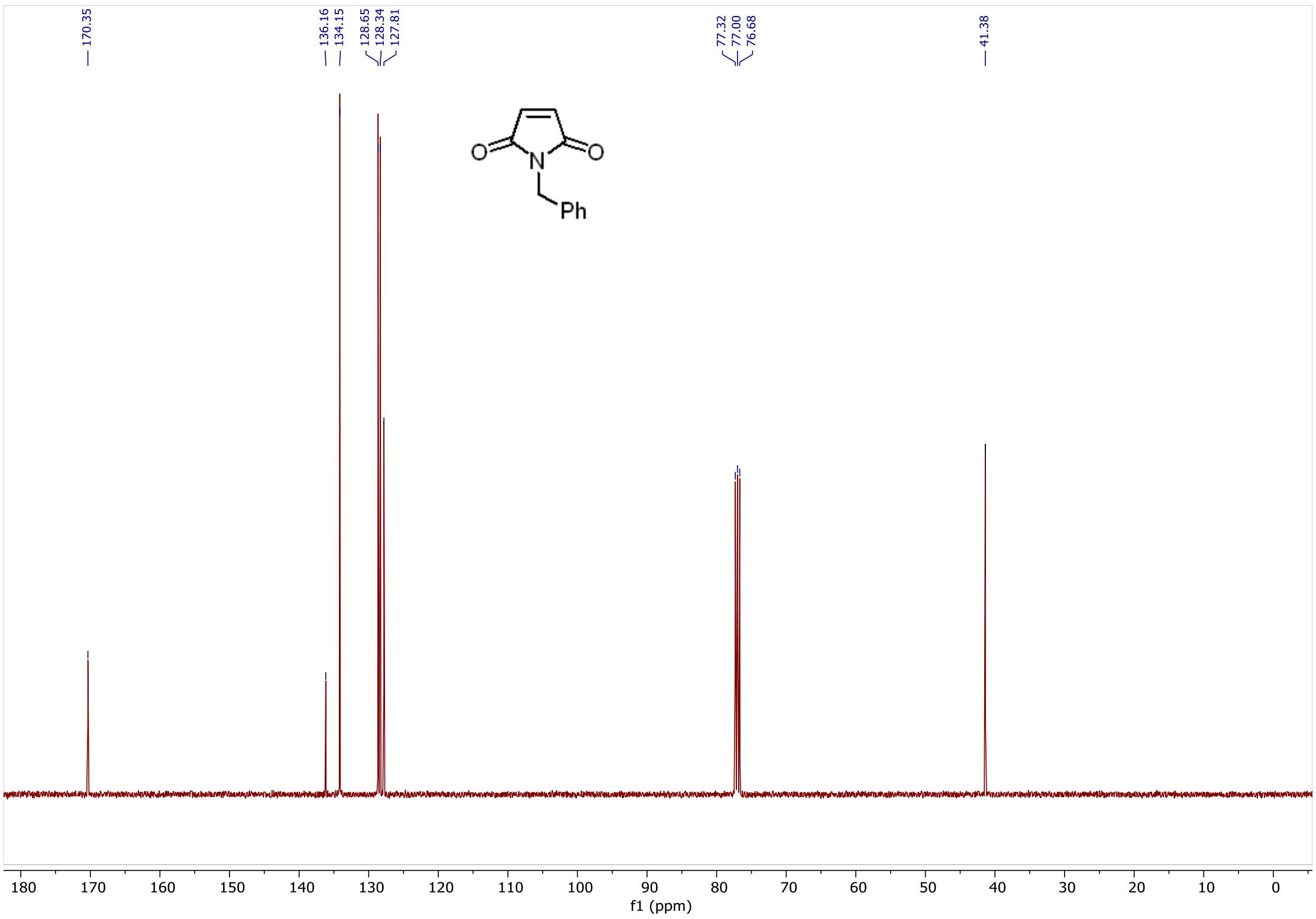

Figure S33. ^{13}C NMR spectrum of **S10**.

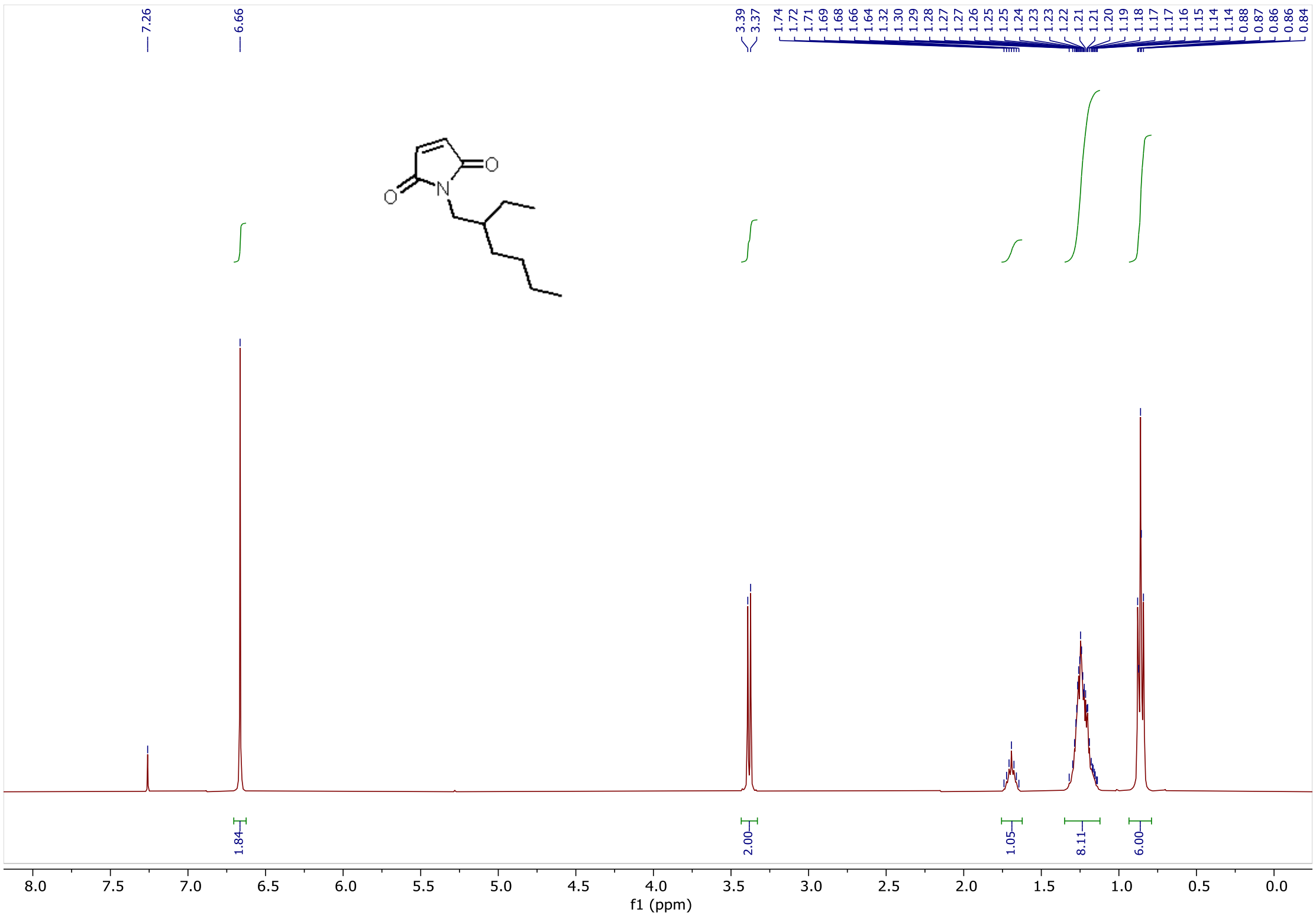

Figure S34. ^{1}H NMR spectrum of **S11**.

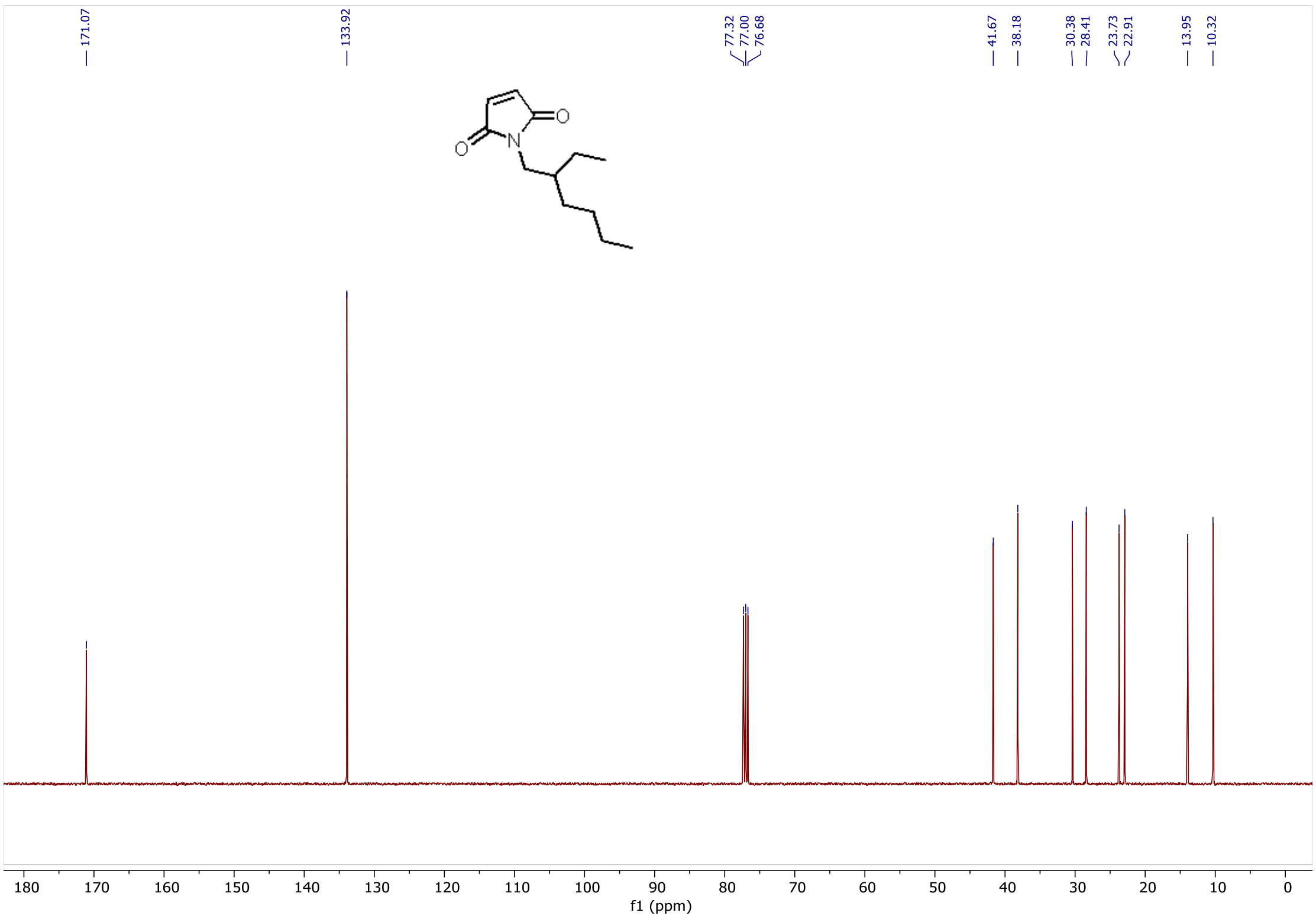

Figure S35. ^{13}C NMR spectrum of **S11**.

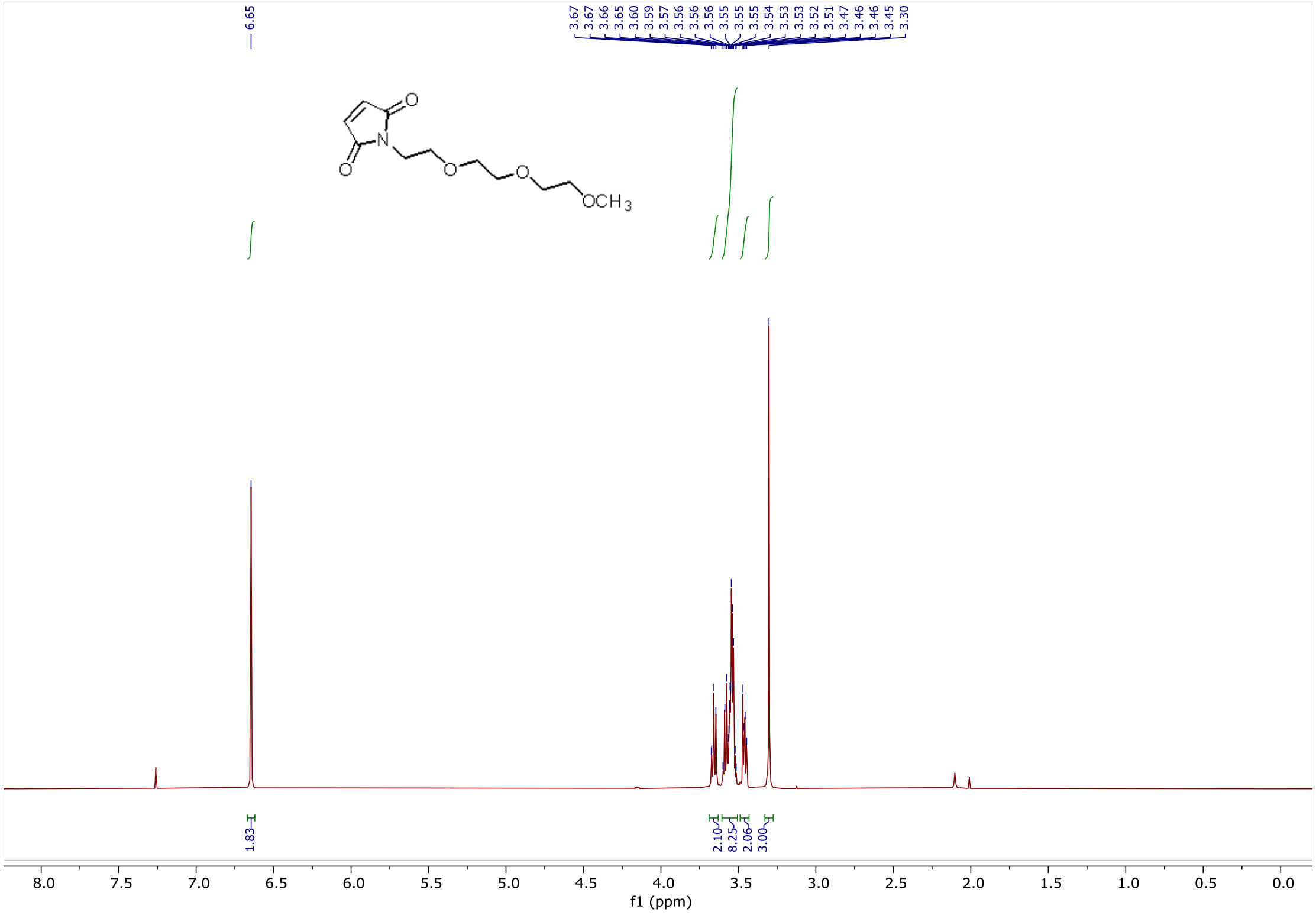

Figure S36. ^{1}H NMR spectrum of **S12**.

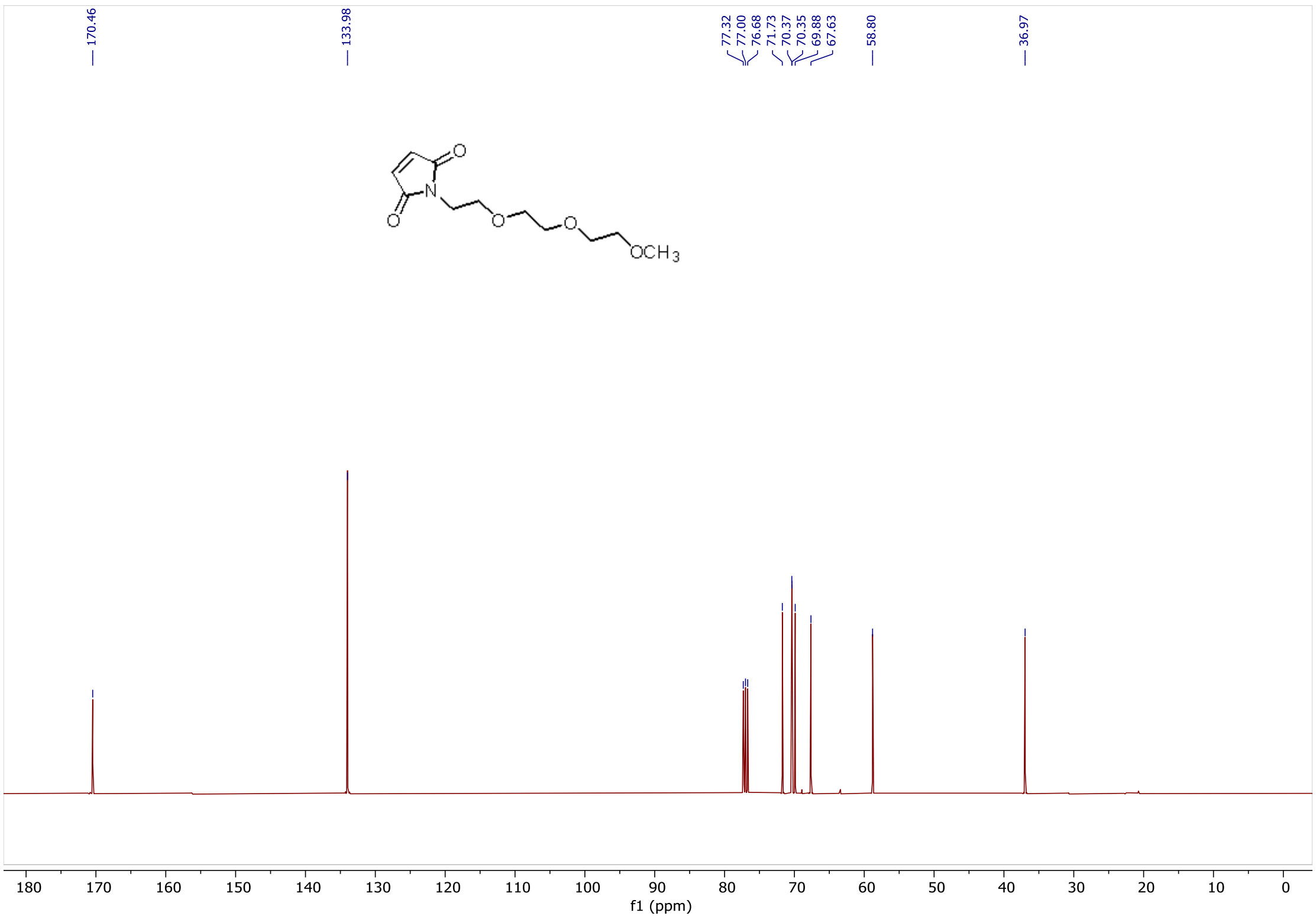

Figure S37. ^{13}C NMR spectrum of **S12**.

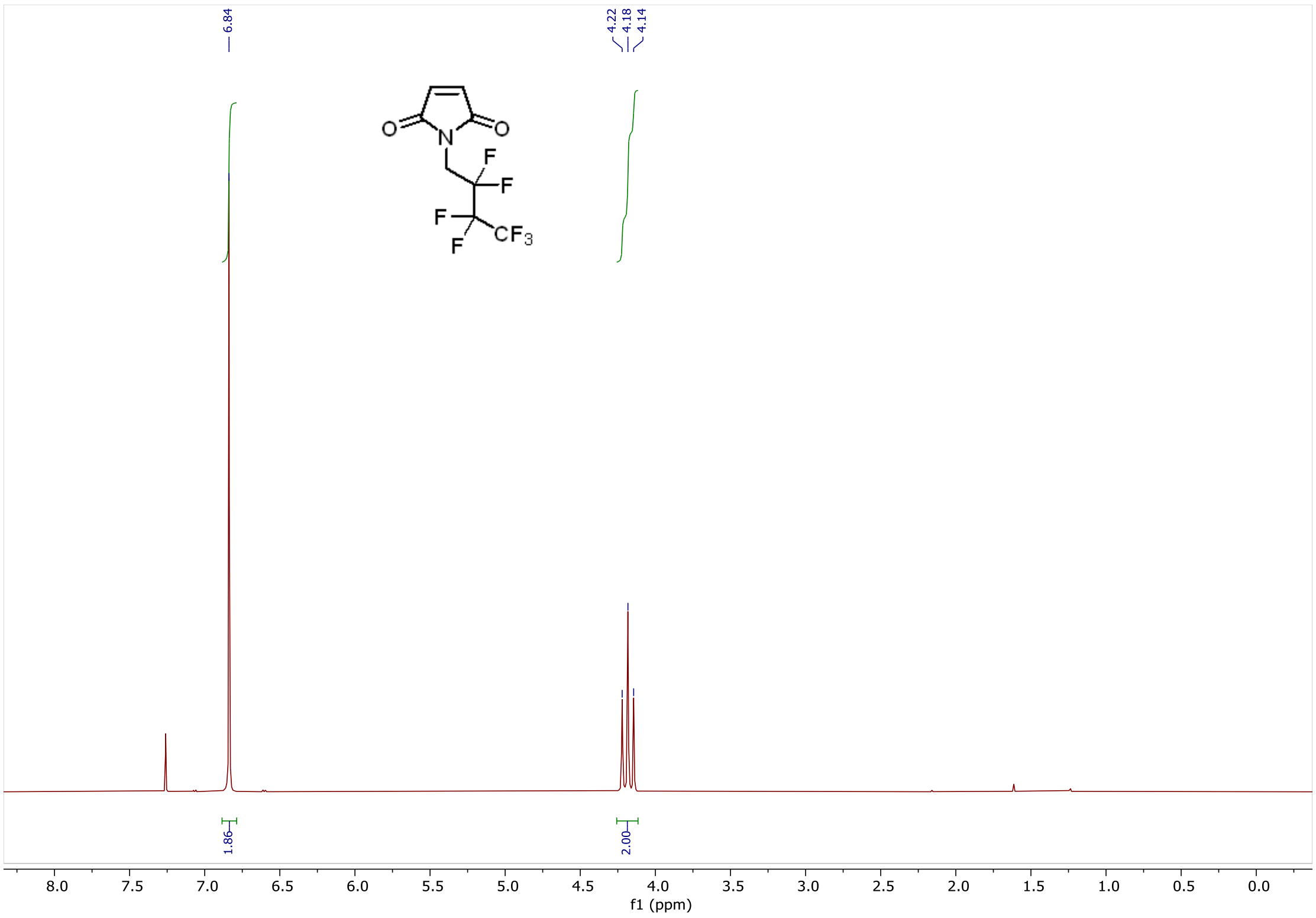

Figure S38. ^{1}H NMR spectrum of **S13**.

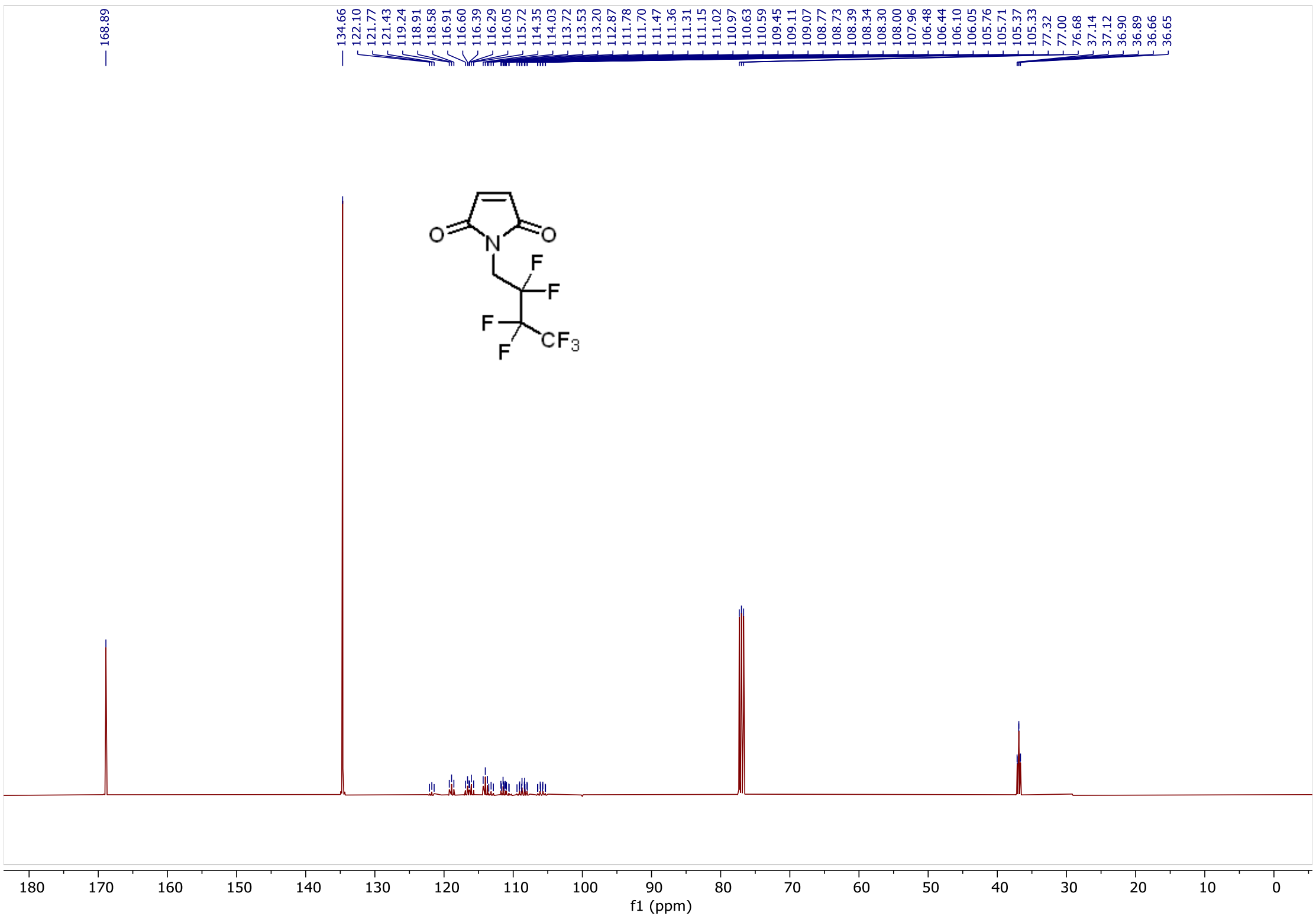

Figure S39. ^{13}C NMR spectrum of **S13**.

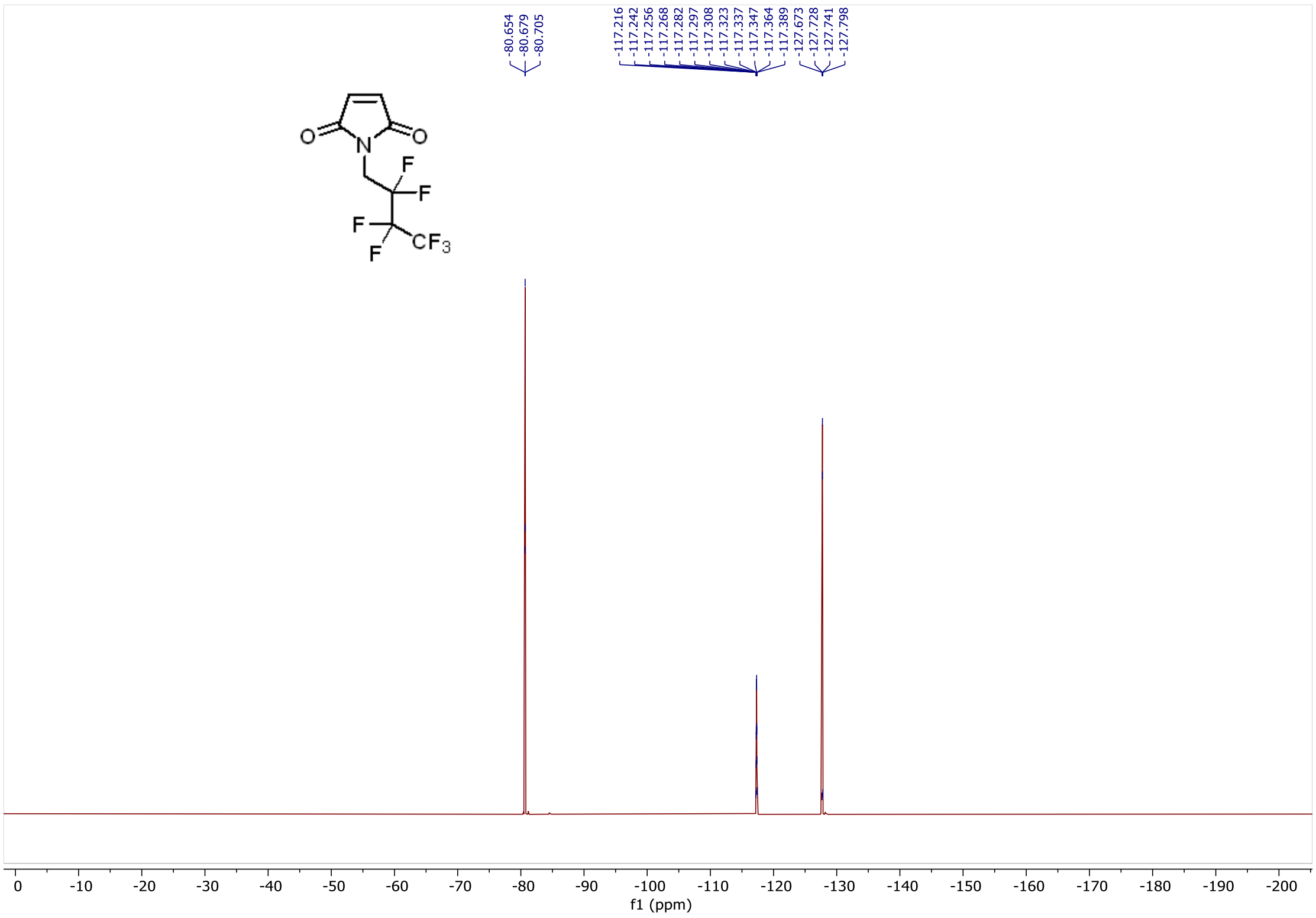

Figure S40. ^{19}F NMR spectrum of **S13**.

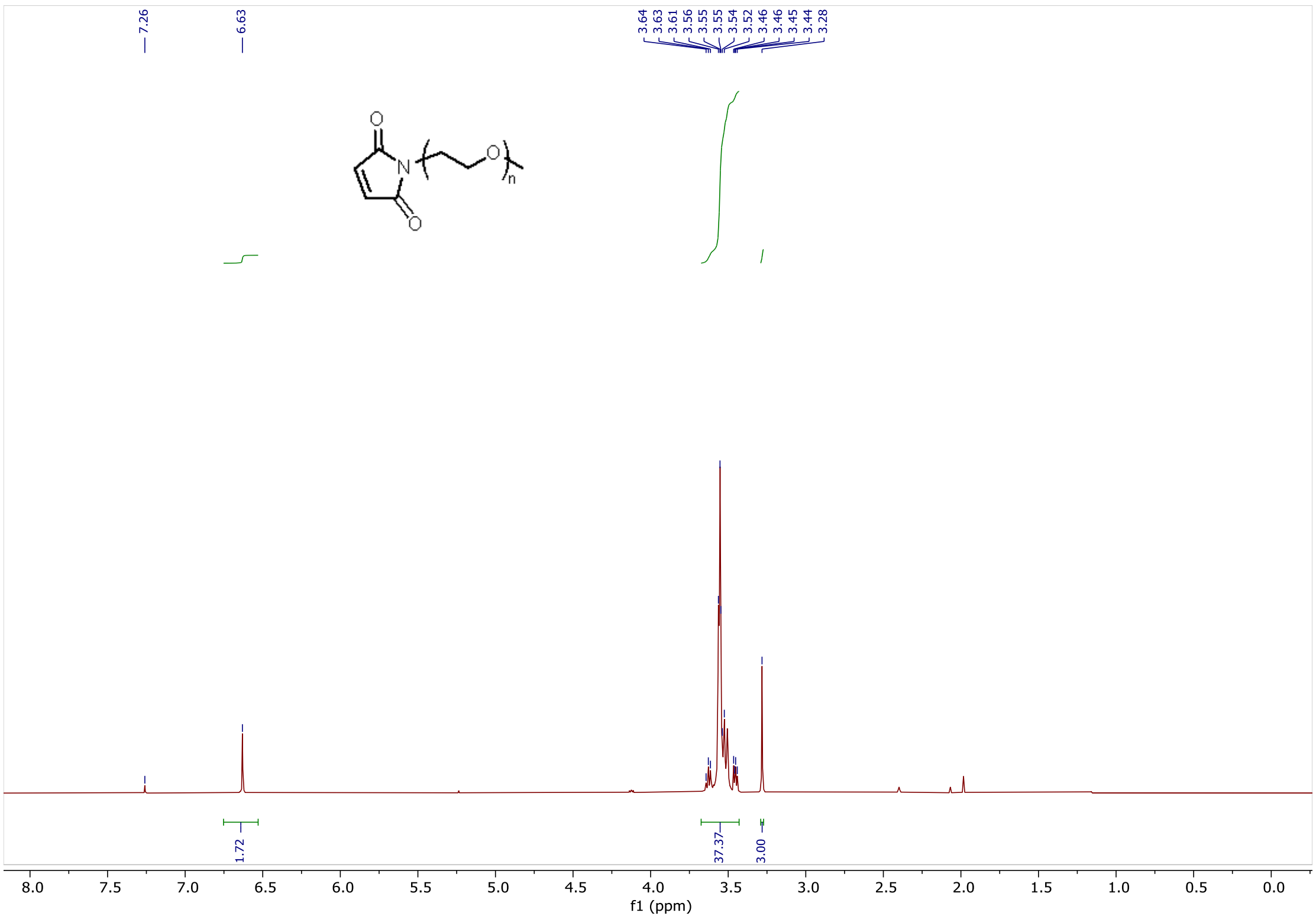

Figure S41. ^{1}H NMR spectrum of **S14**.

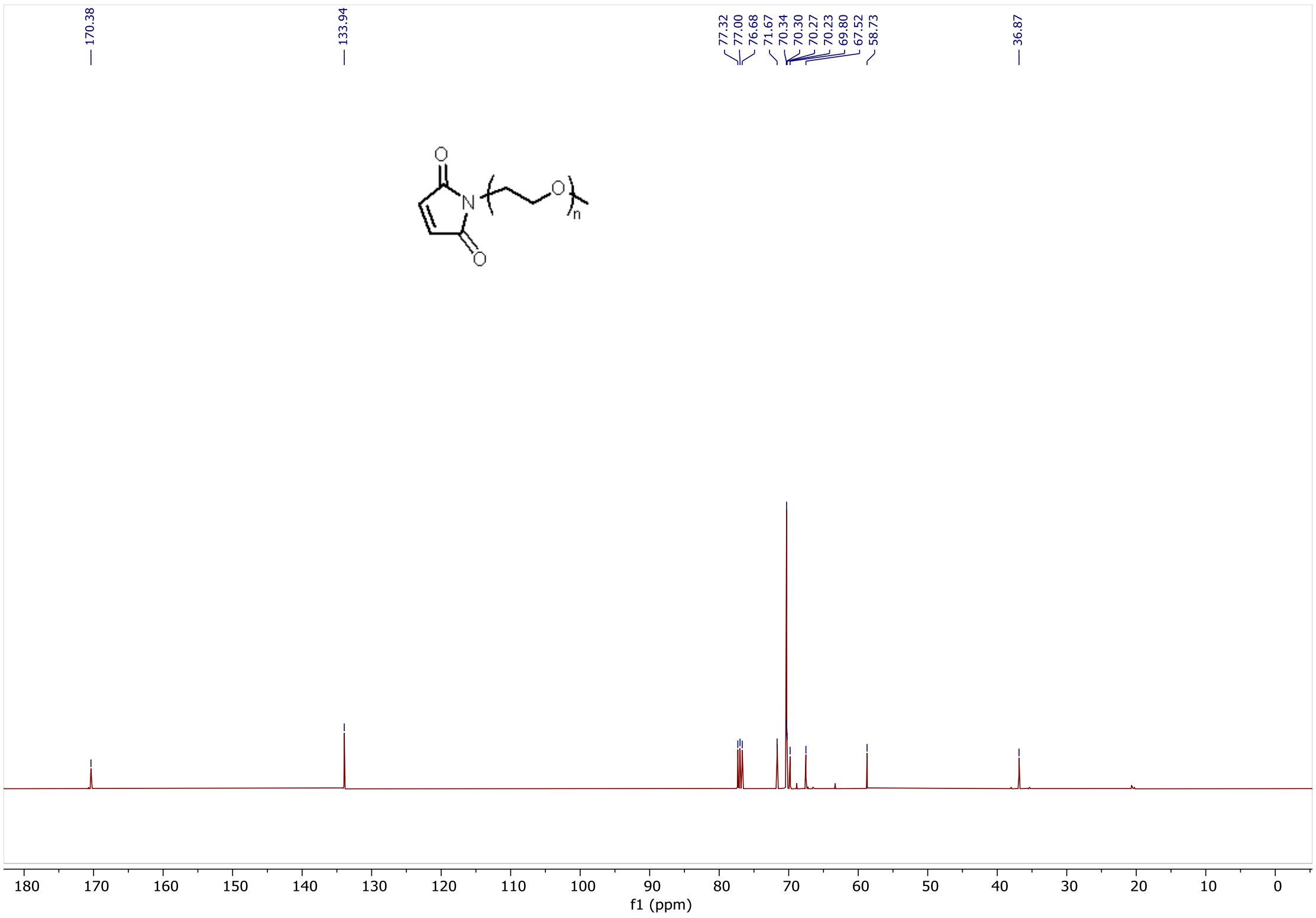

Figure S42. ^{13}C NMR spectrum of **S14**.

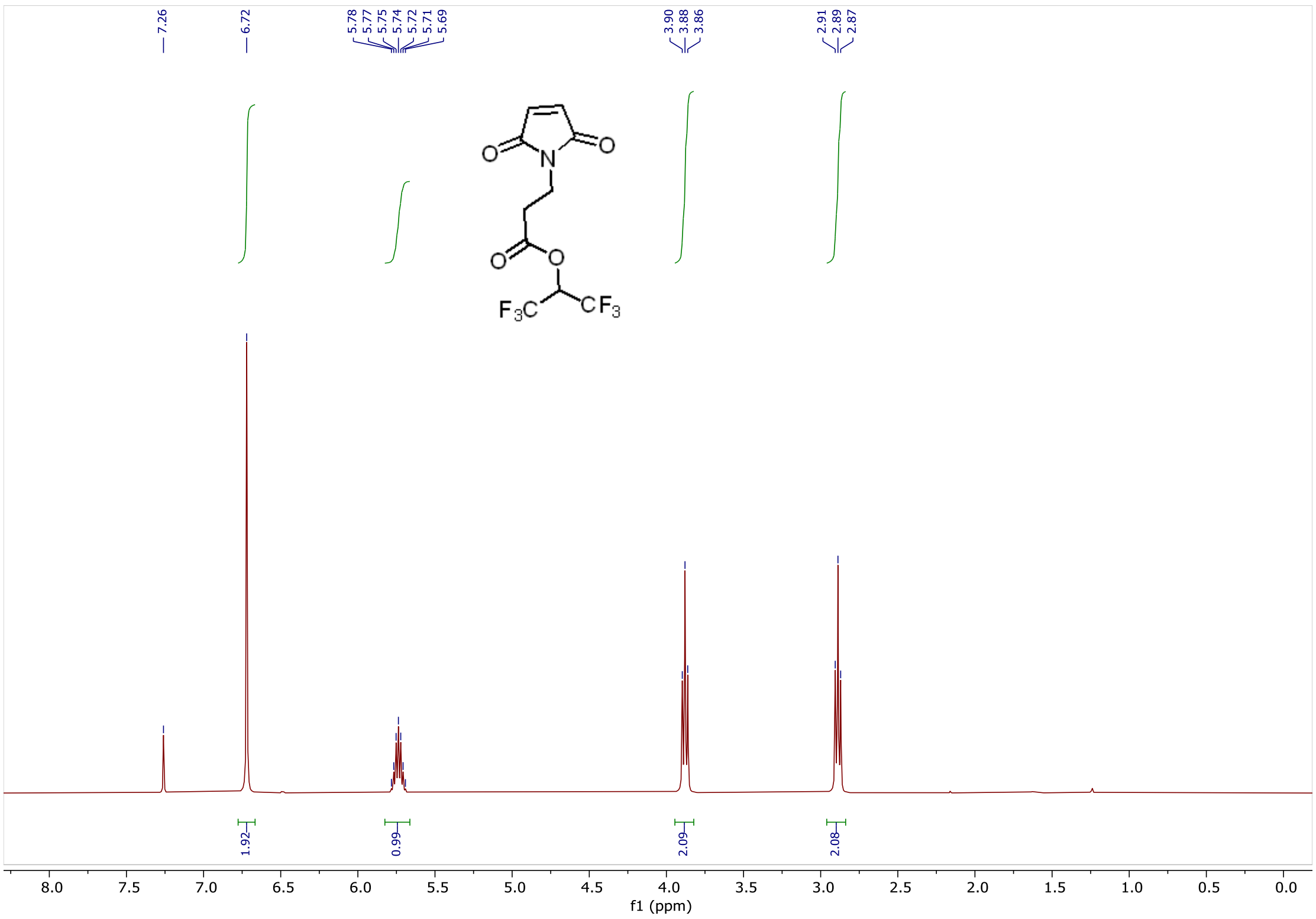

Figure S43. ^{1}H NMR spectrum of **S15**.

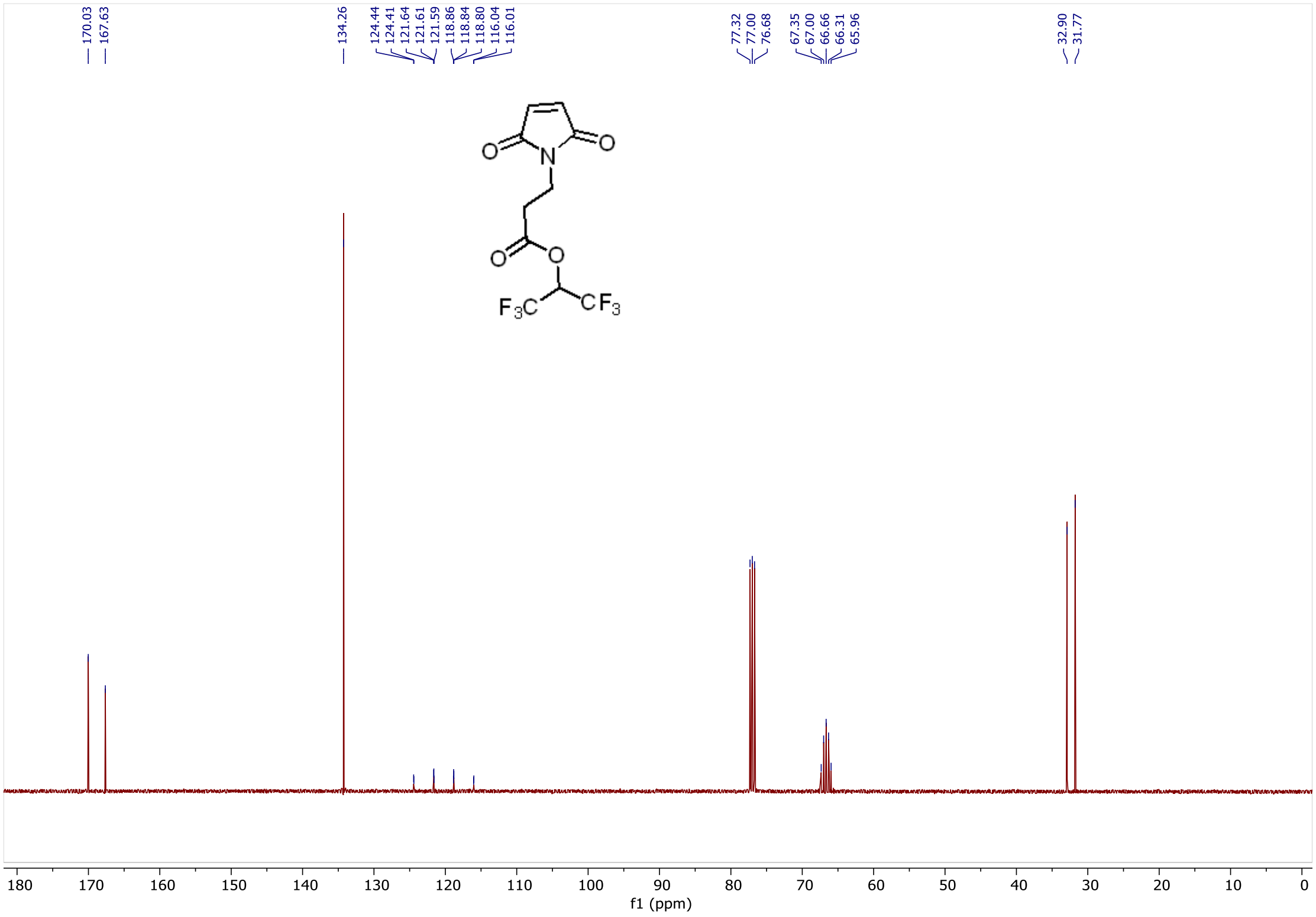

Figure S44. ^{13}C NMR spectrum of **S15**.

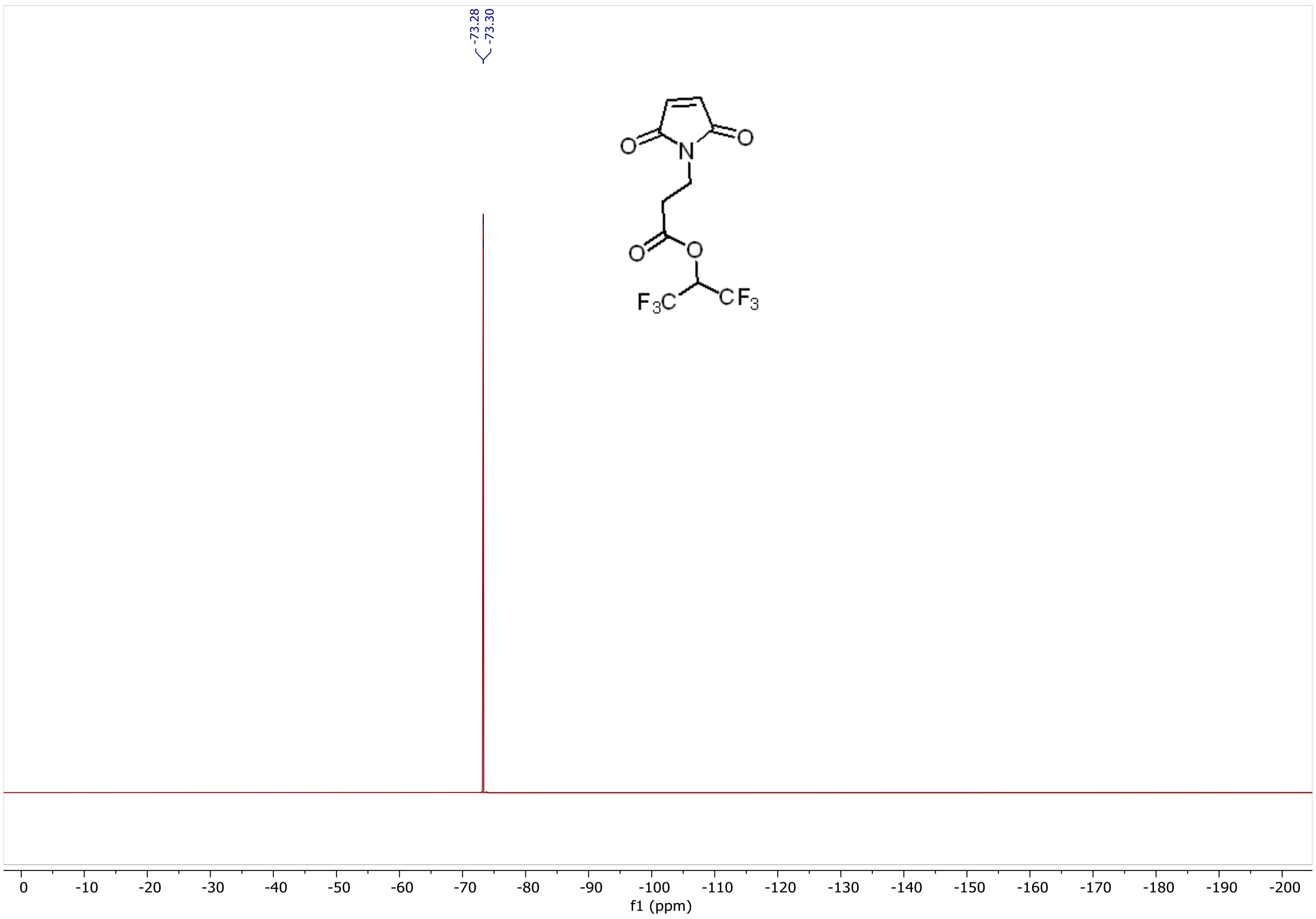

Figure S45. ^{19}F NMR spectrum of **S15**.

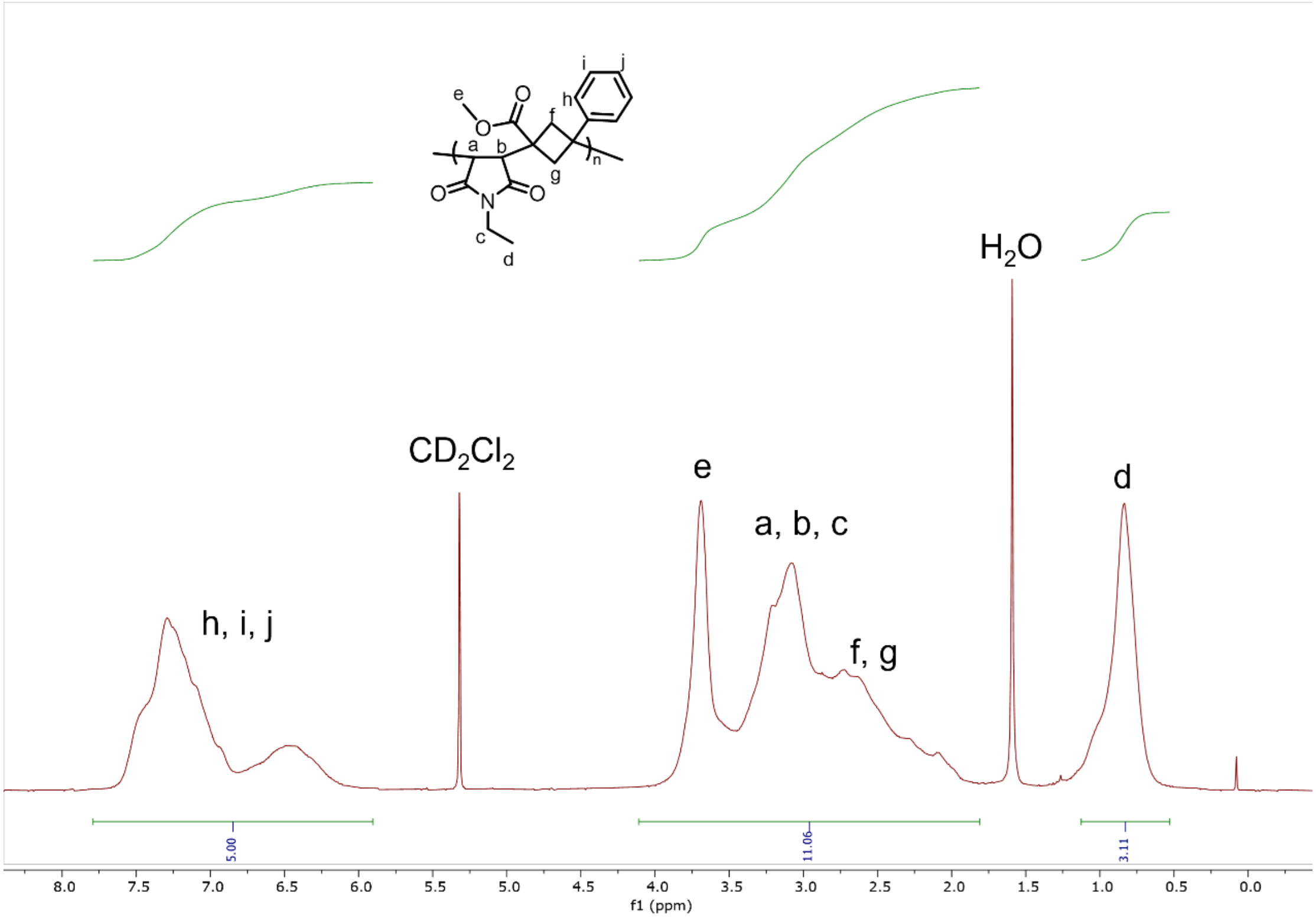

Figure S46. ^{1}H NMR spectrum of **P(BCB1-*alt*-EtMI)**.

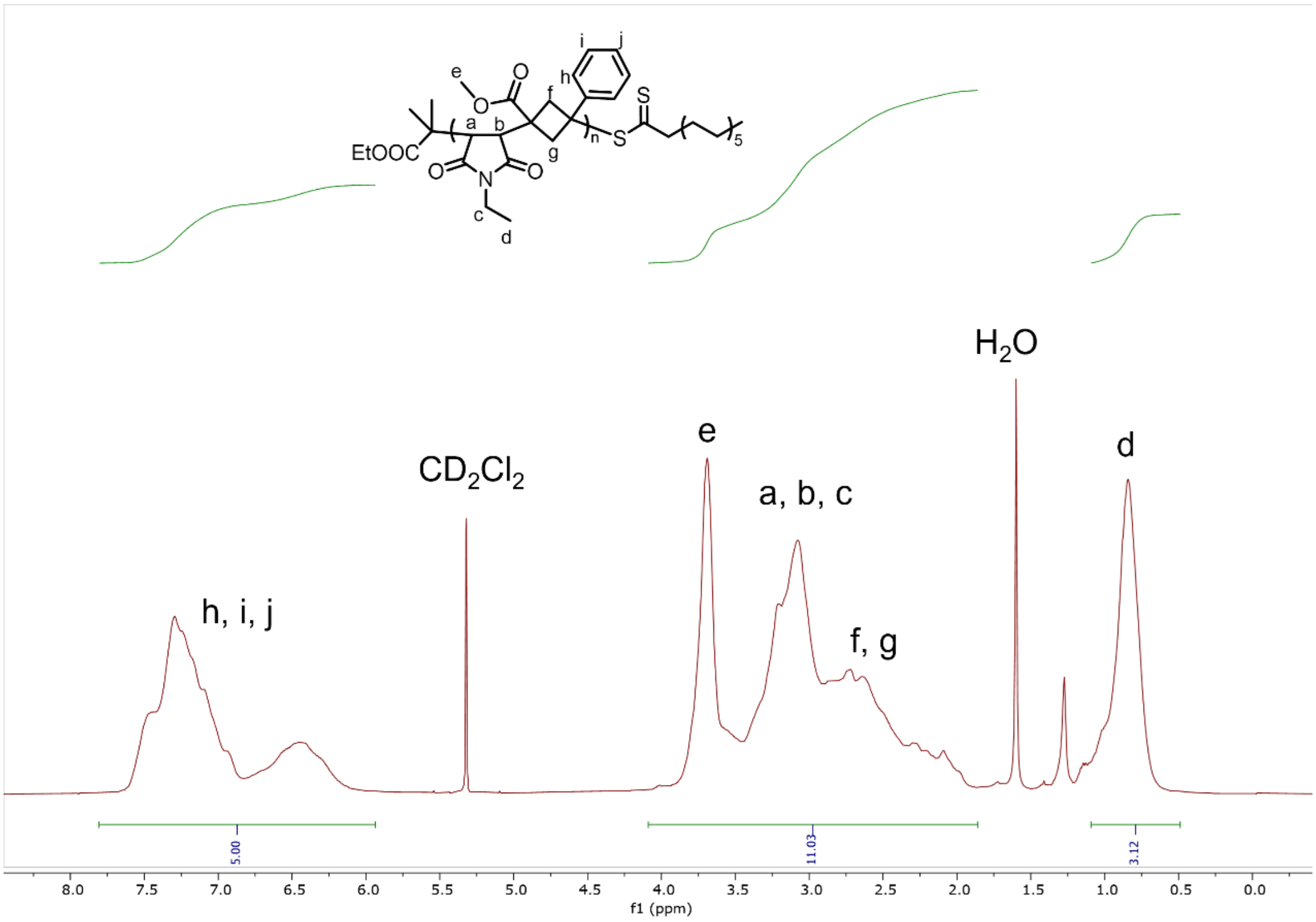

Figure S47. ^{1}H NMR spectrum of **P1.**

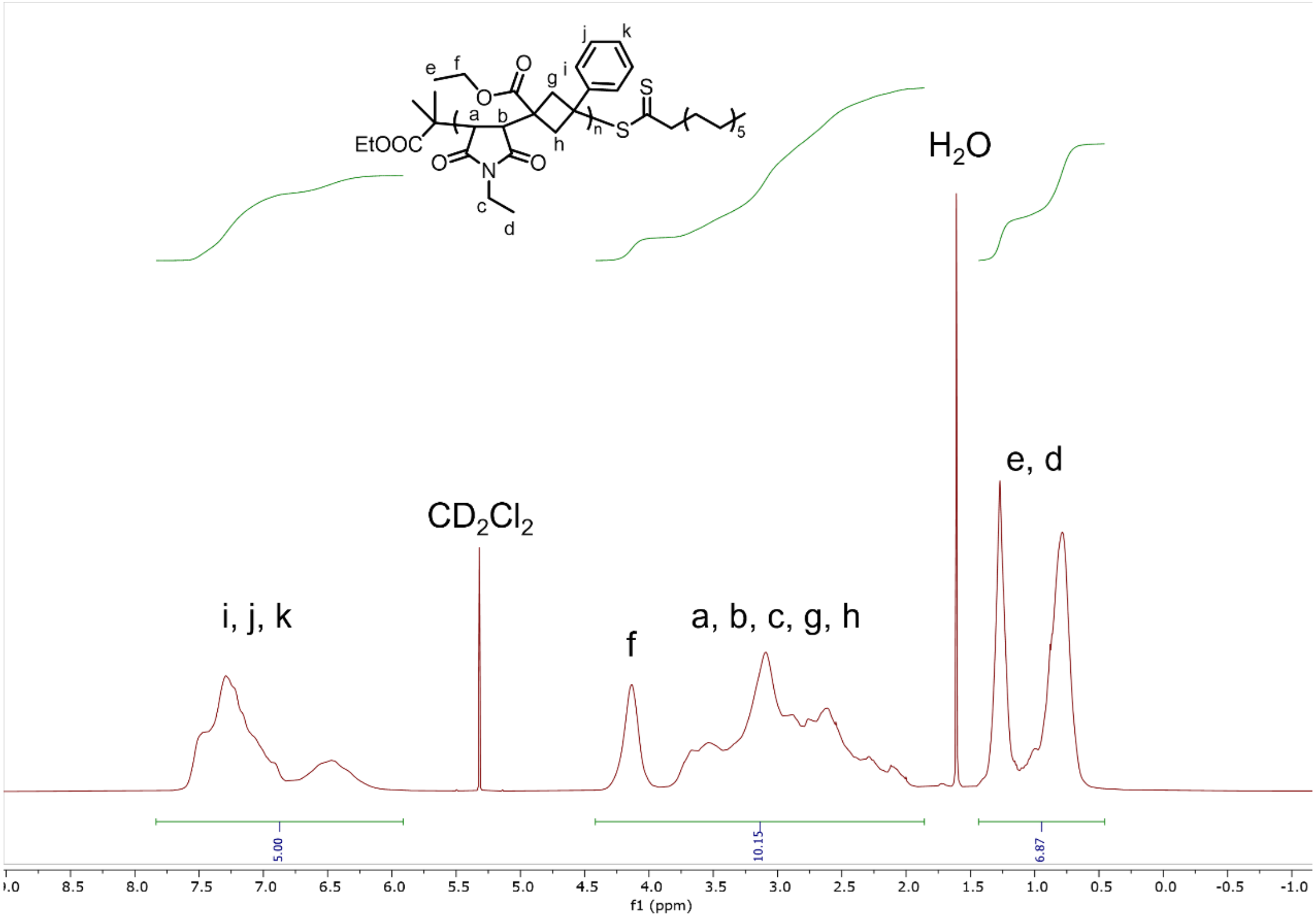

Figure S48. ^{1}H NMR spectrum of **P2.**

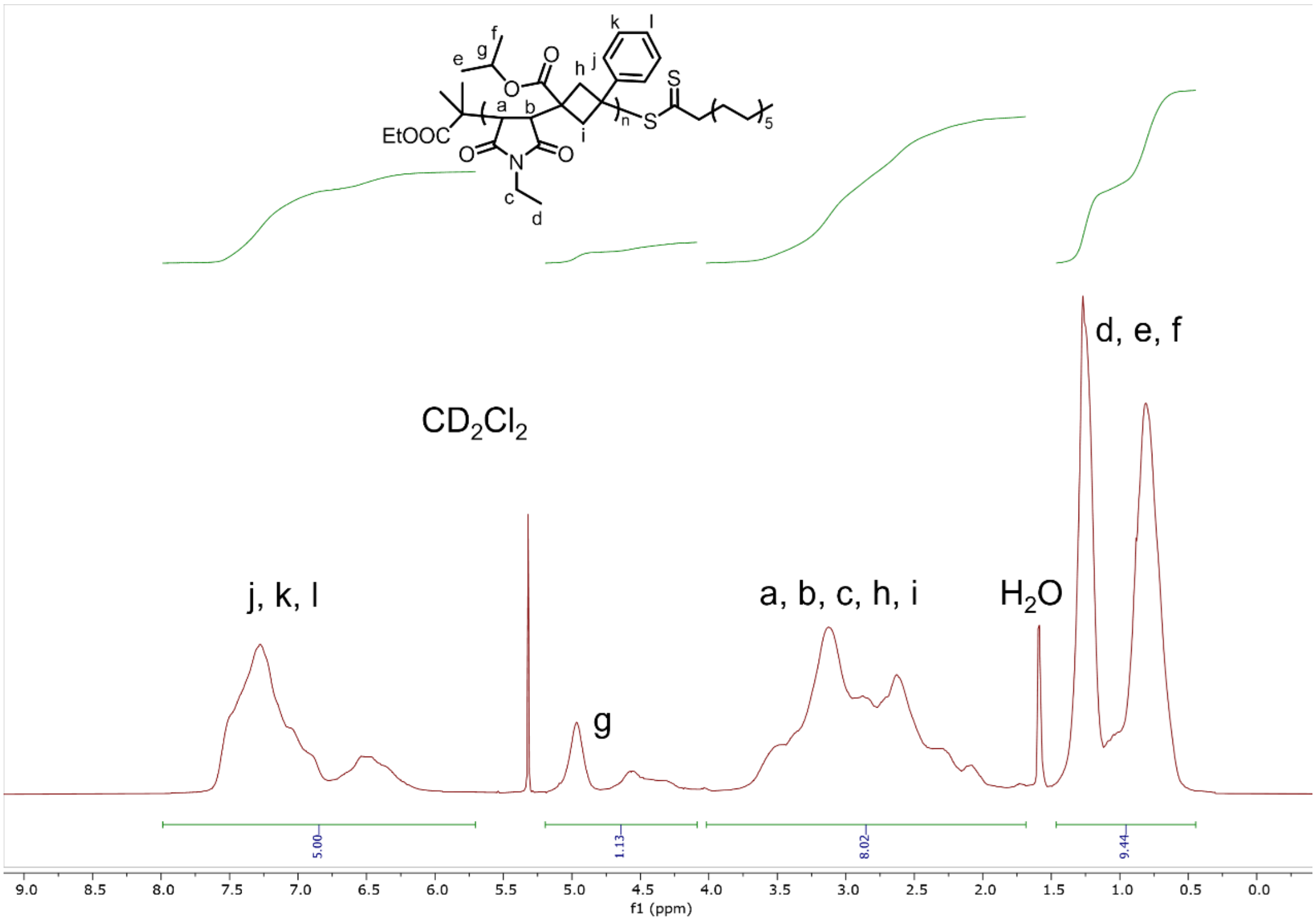

Figure S49. ^{1}H NMR spectrum of **P3.**

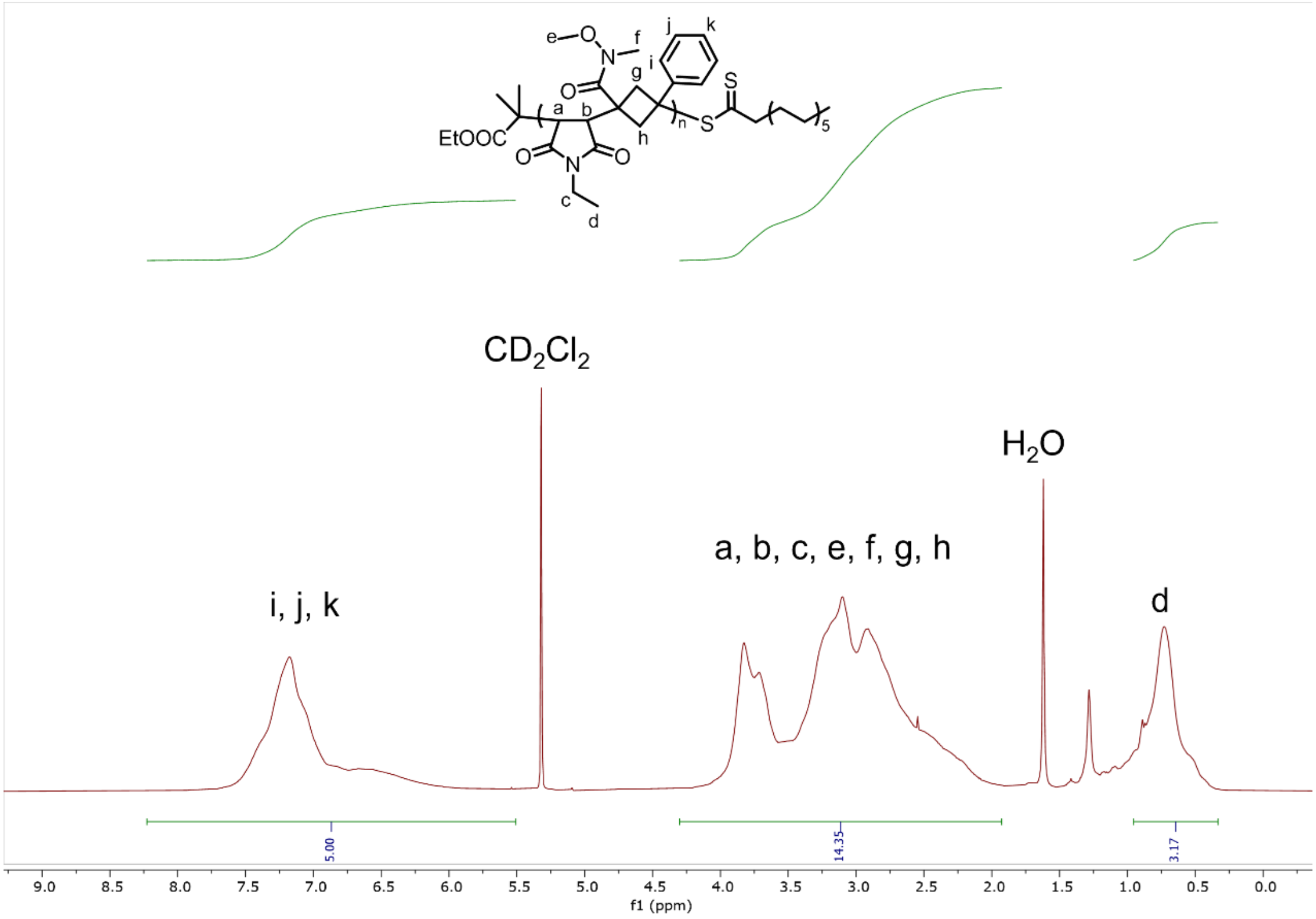

Figure S50. ^{1}H NMR spectrum of **P4.**

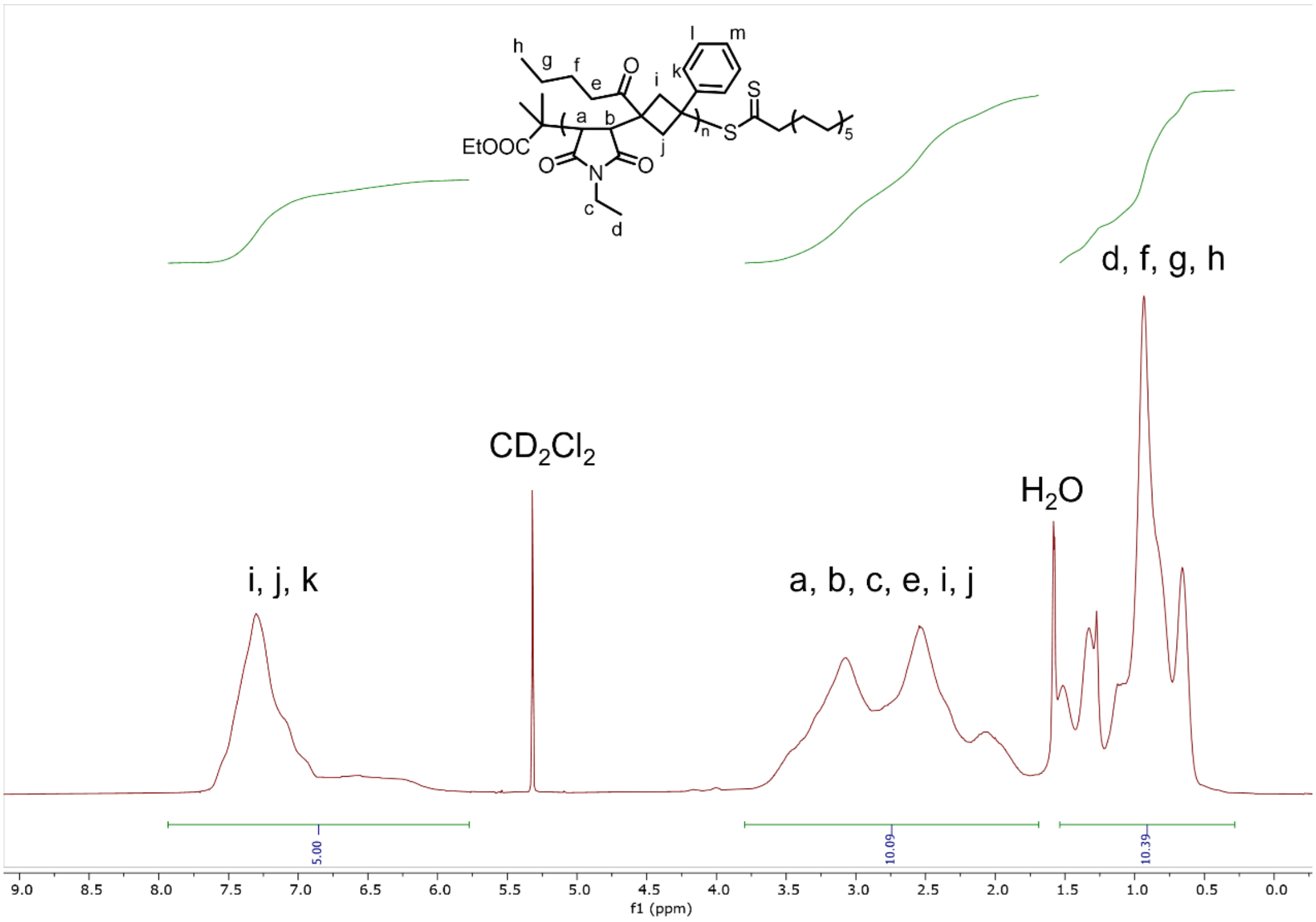

Figure S51. ^{1}H NMR spectrum of **P5.**

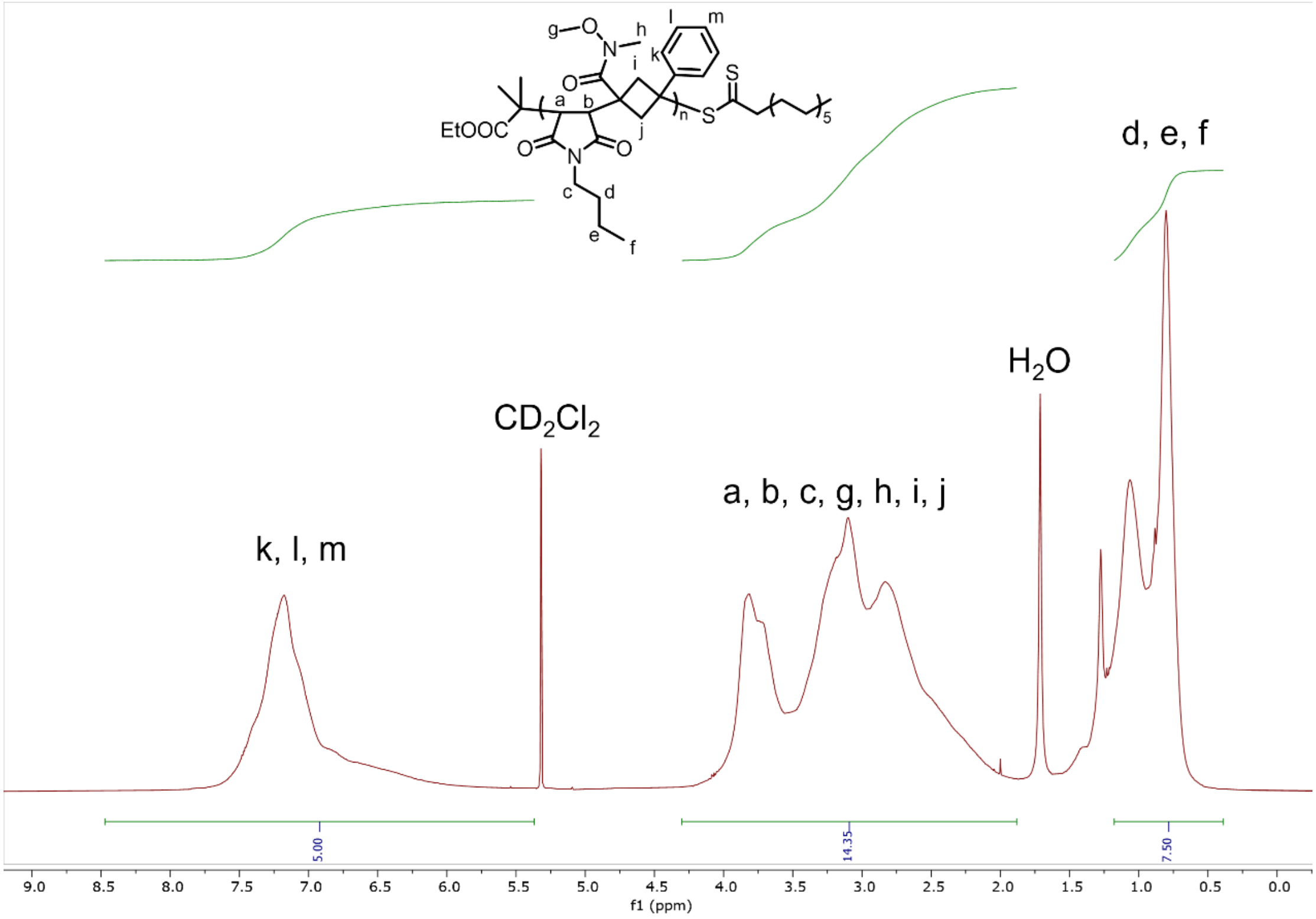

Figure S52. ^{1}H NMR spectrum of **P6.**

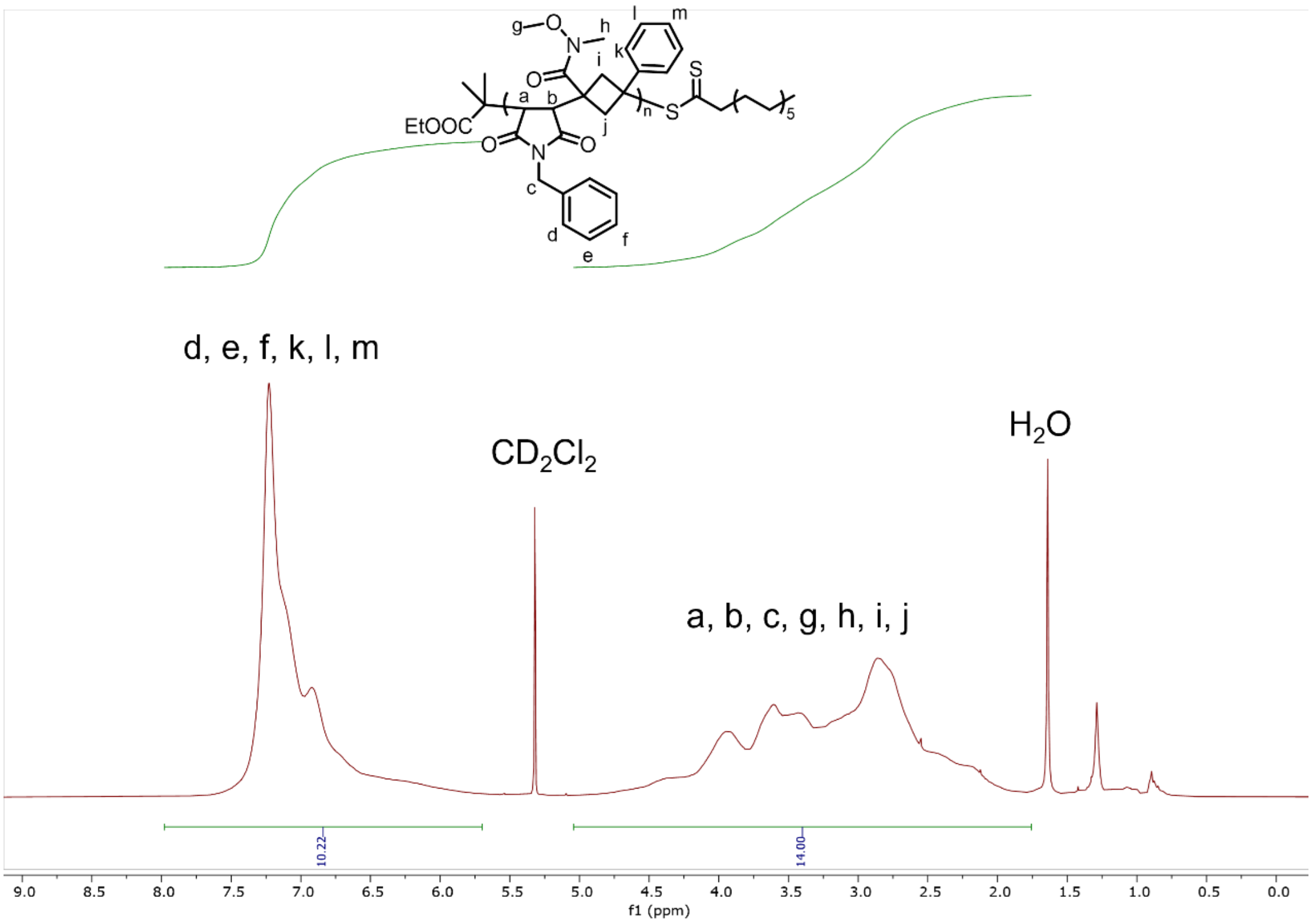

Figure S53. ^{1}H NMR spectrum of **P7.**

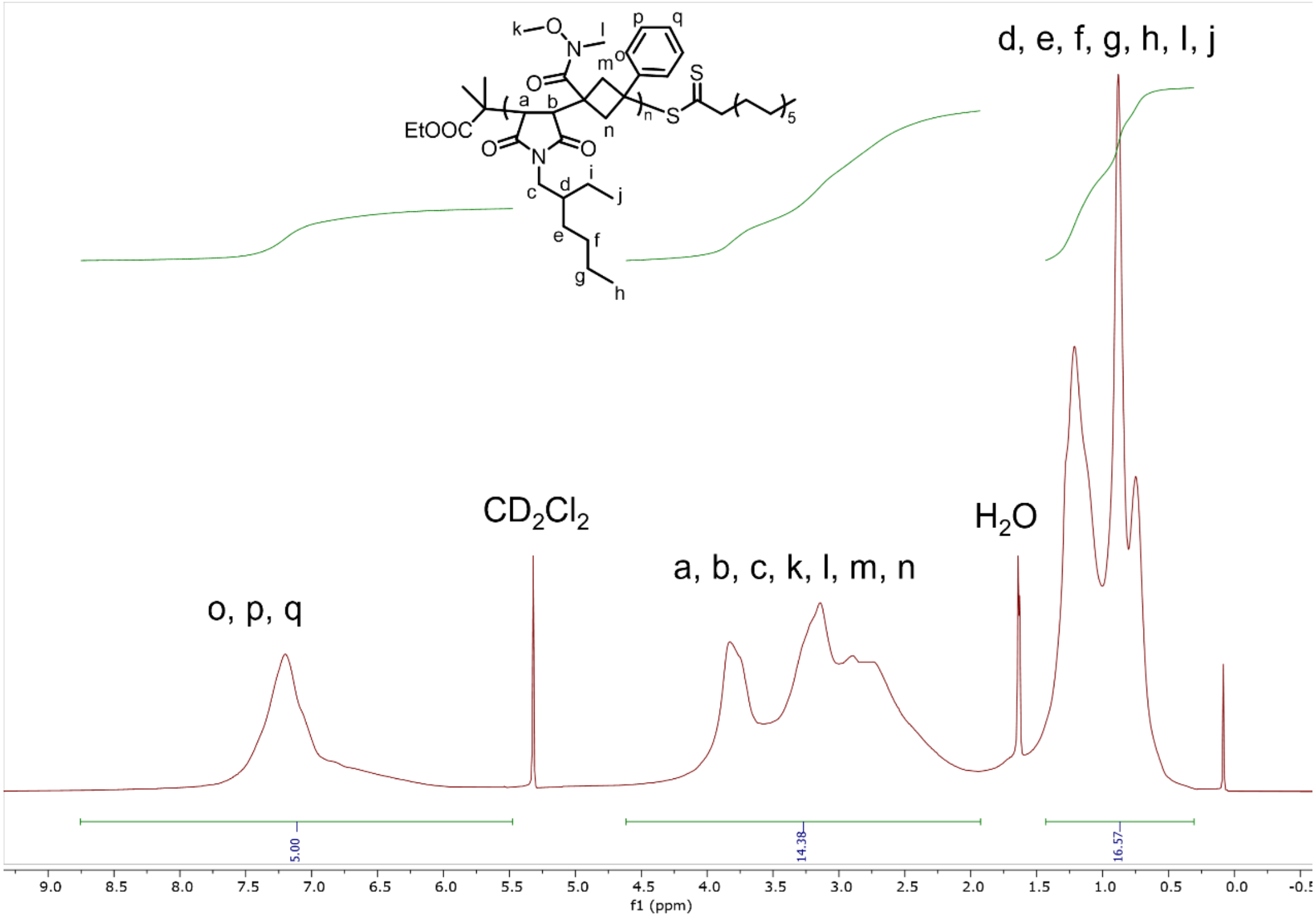

Figure S54. ^{1}H NMR spectrum of **P8.**

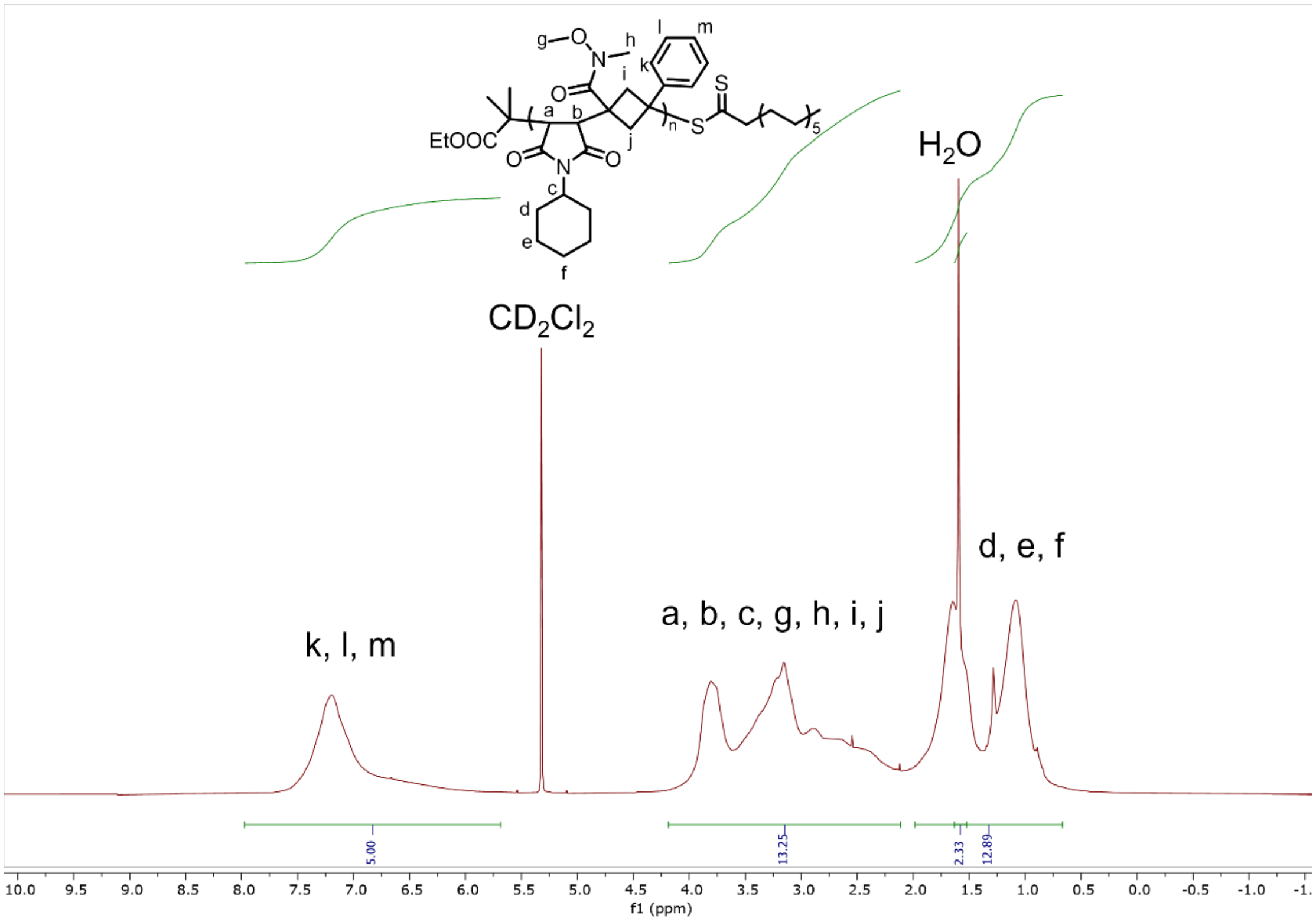

Figure S55. ^{1}H NMR spectrum of **P9.**

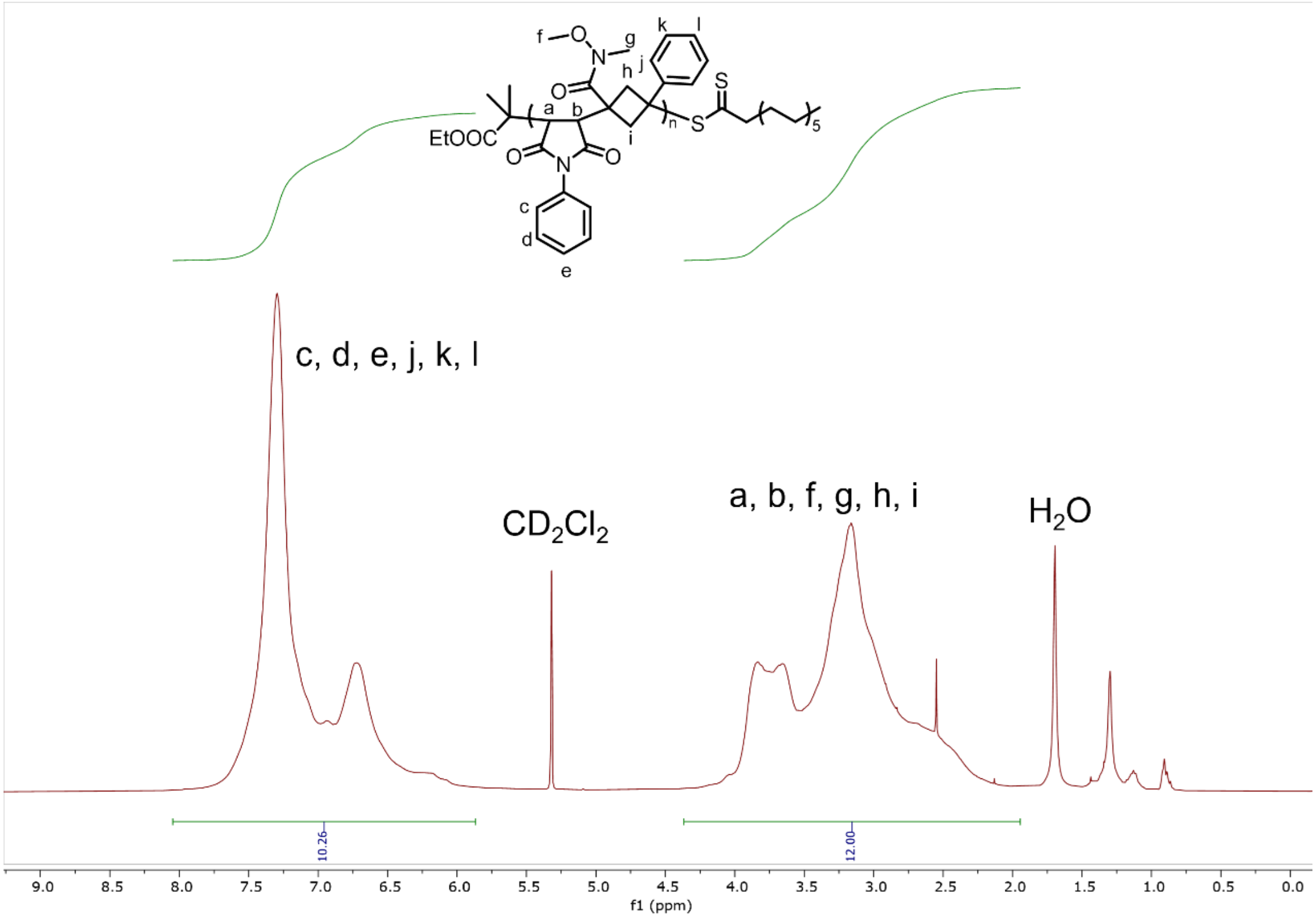

Figure S56. ^{1}H NMR spectrum of **P10.**

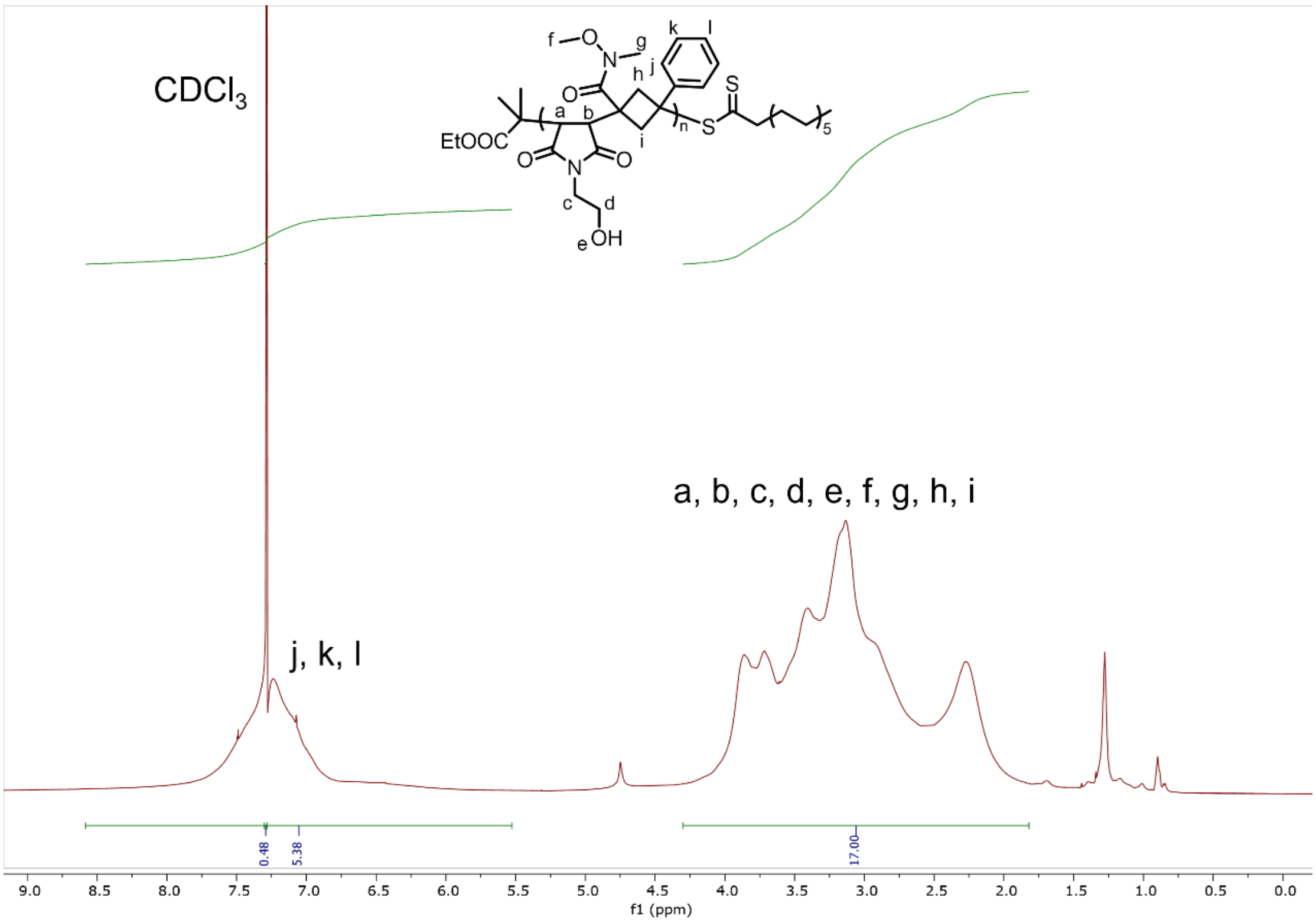

Figure S57. ^{1}H NMR spectrum of **P11.**

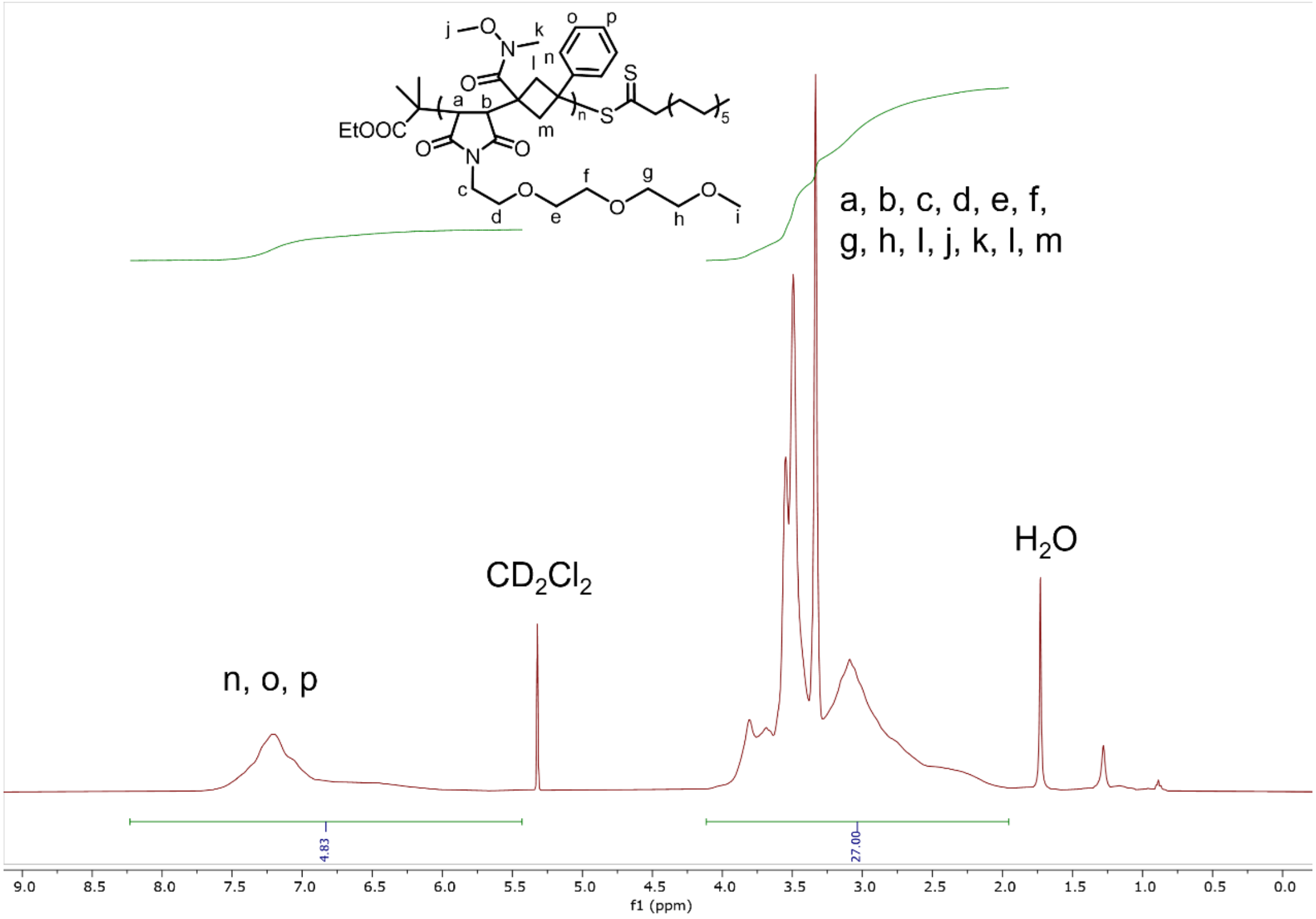

Figure S58. ^{1}H NMR spectrum of **P12.**

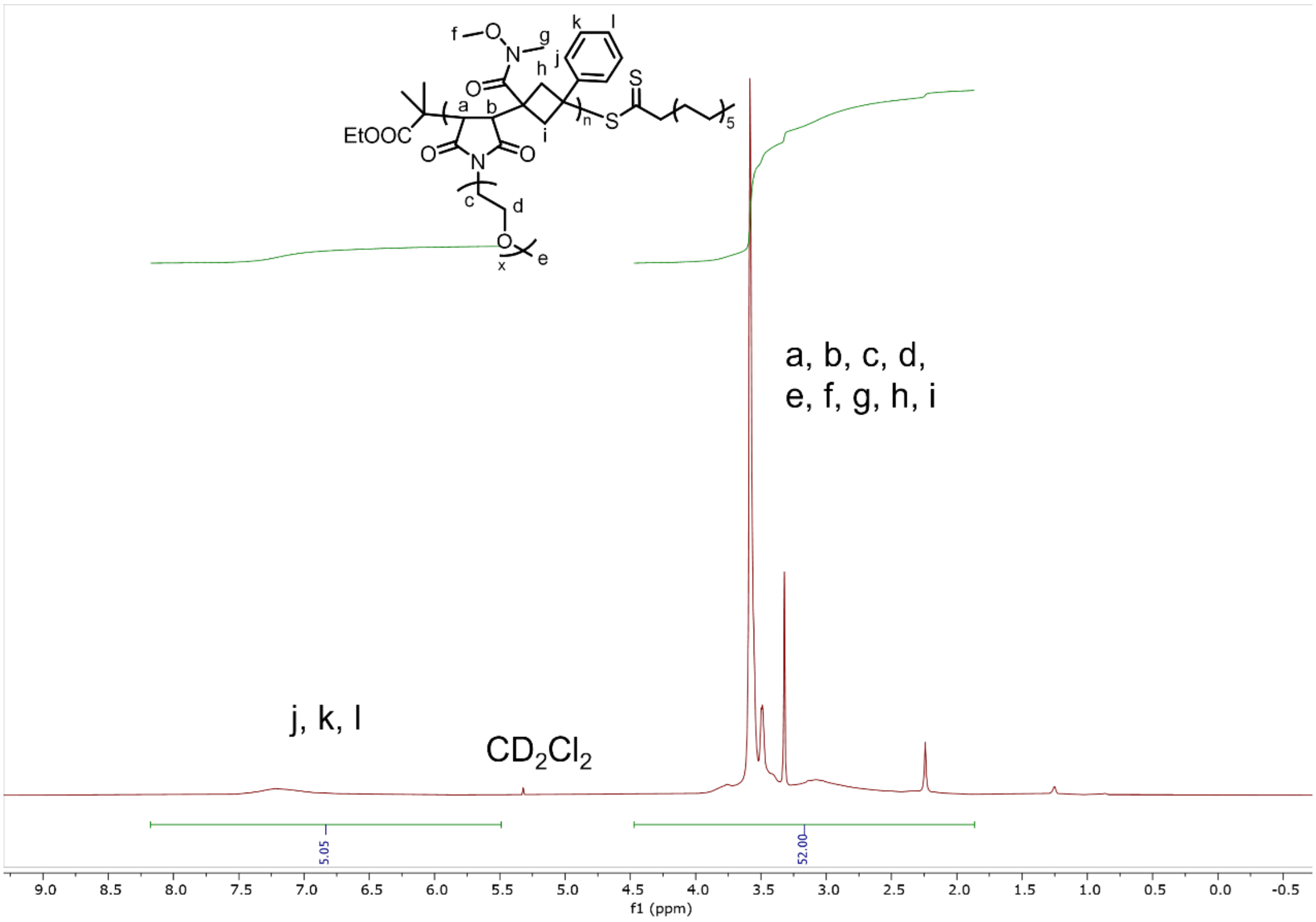

Figure S59. ^{1}H NMR spectrum of **P13.**

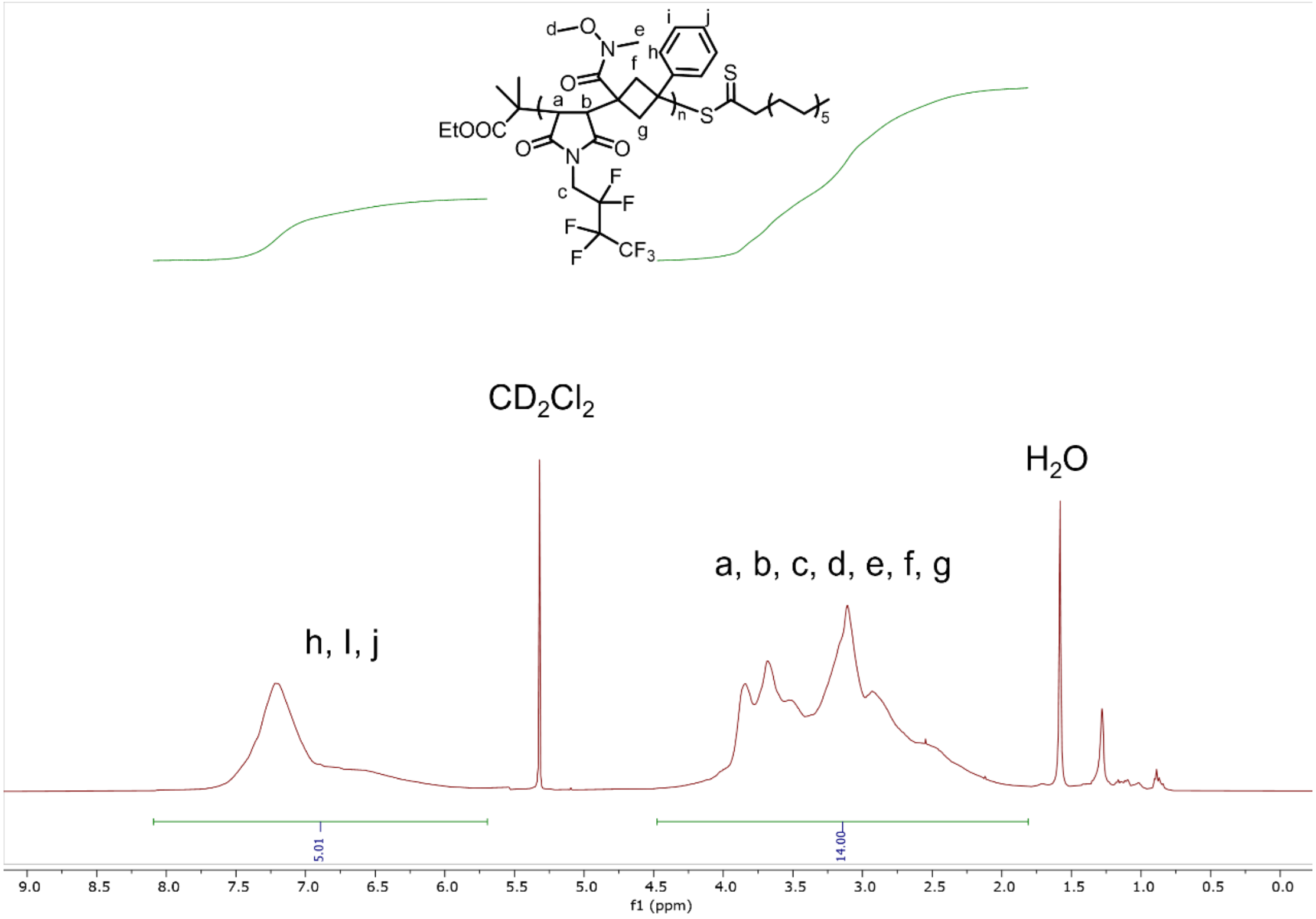

Figure S60. ^{1}H NMR spectrum of **P14.**

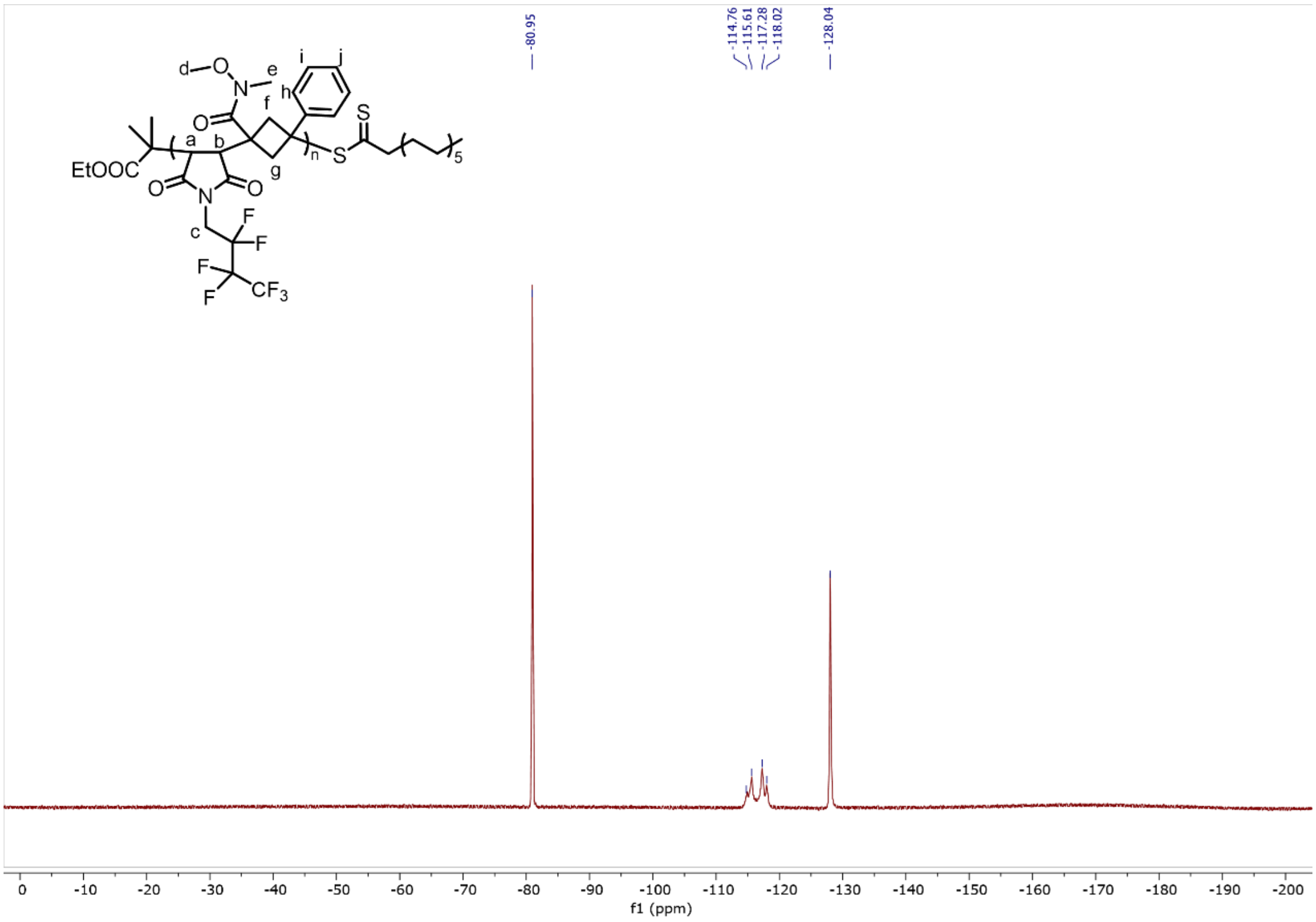

Figure S61. ^{19}F NMR spectrum of **P14.**

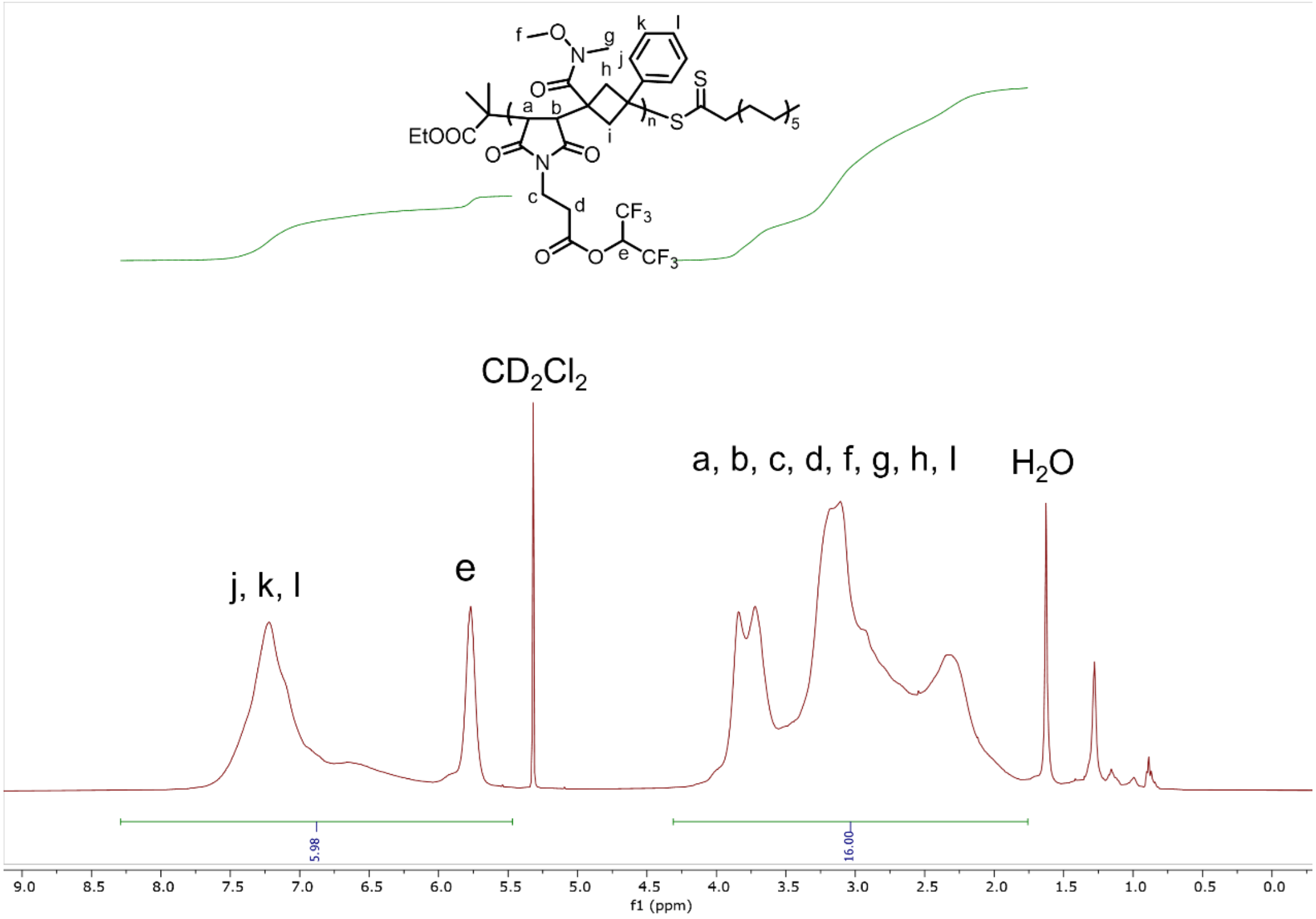

Figure S62. ^{1}H NMR spectrum of **P15.**

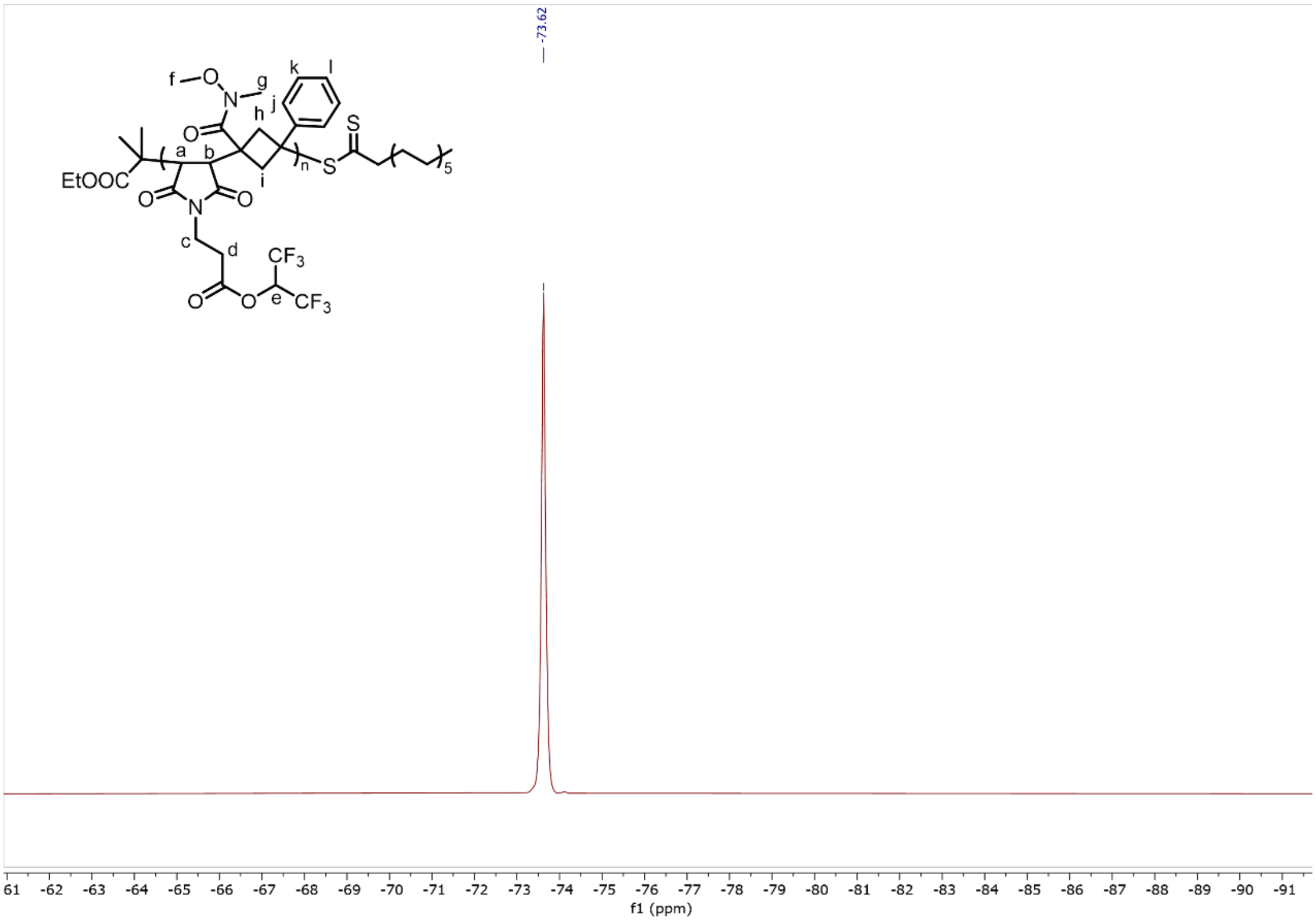

Figure S63. ^{19}F NMR spectrum of **P15.**

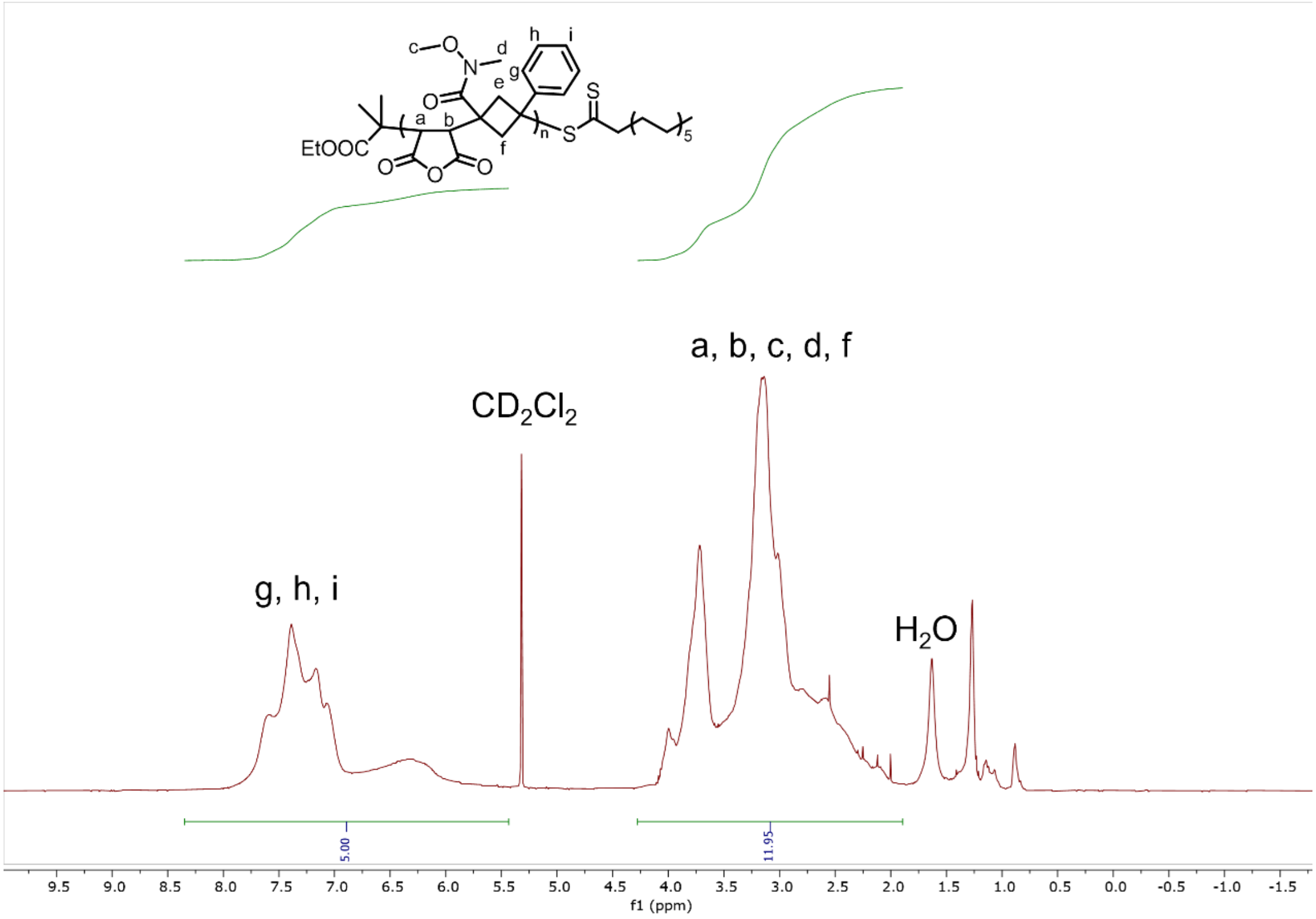

Figure S64. ^{1}H NMR spectrum of **P16.**

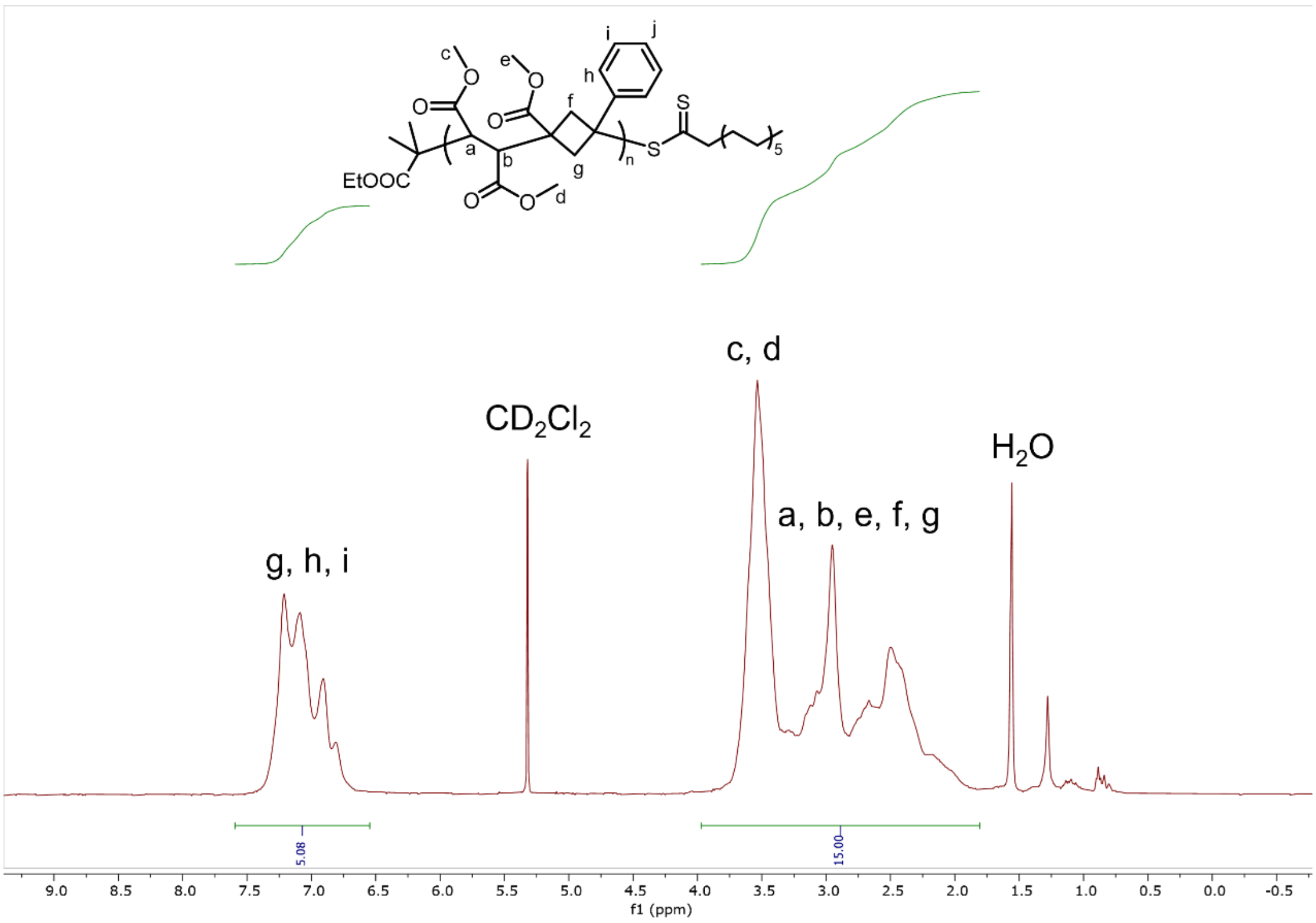

Figure S65. ^{1}H NMR spectrum of **P17.**

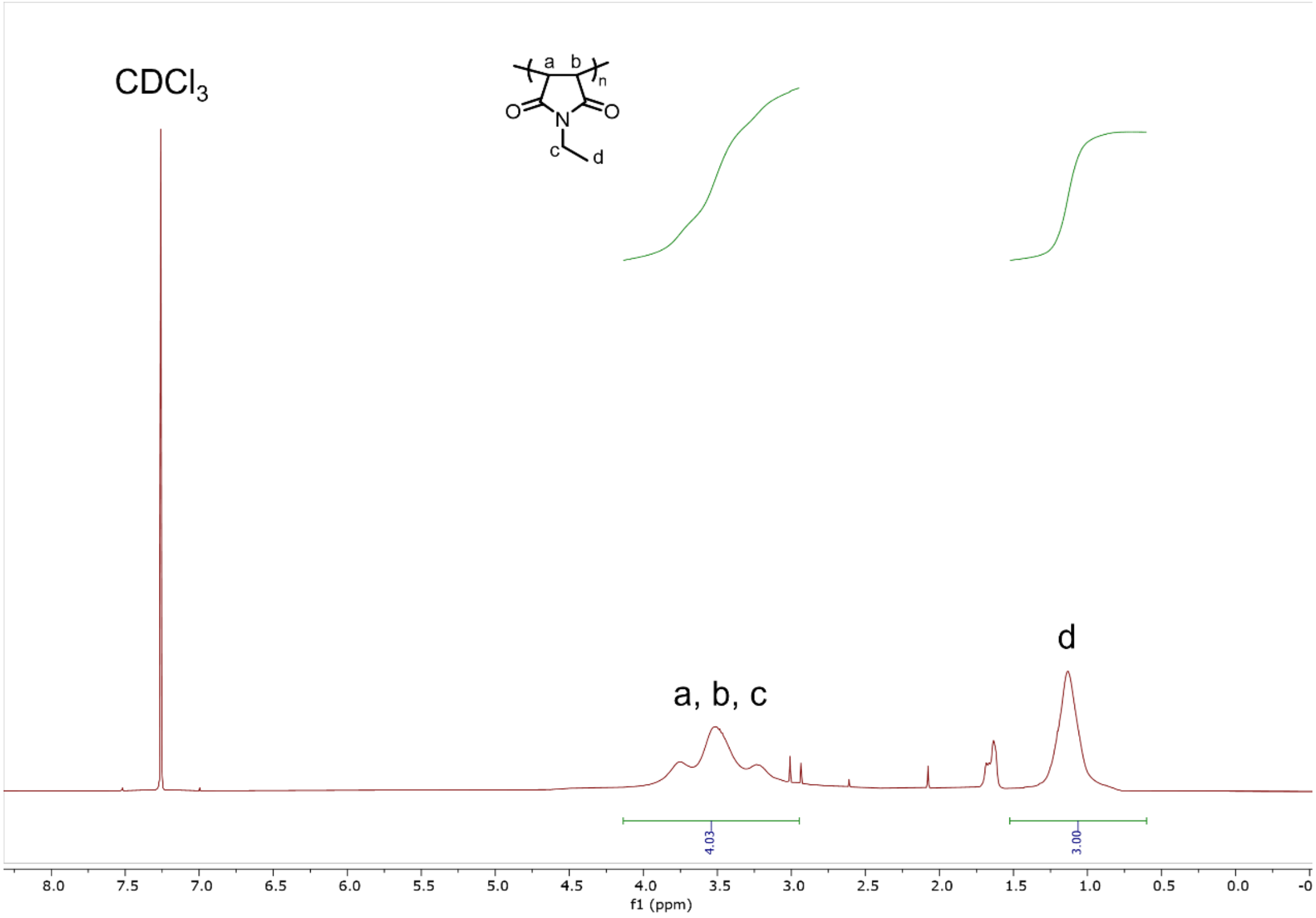

Figure S66. ^{1}H NMR spectrum of **P(EtMI).**

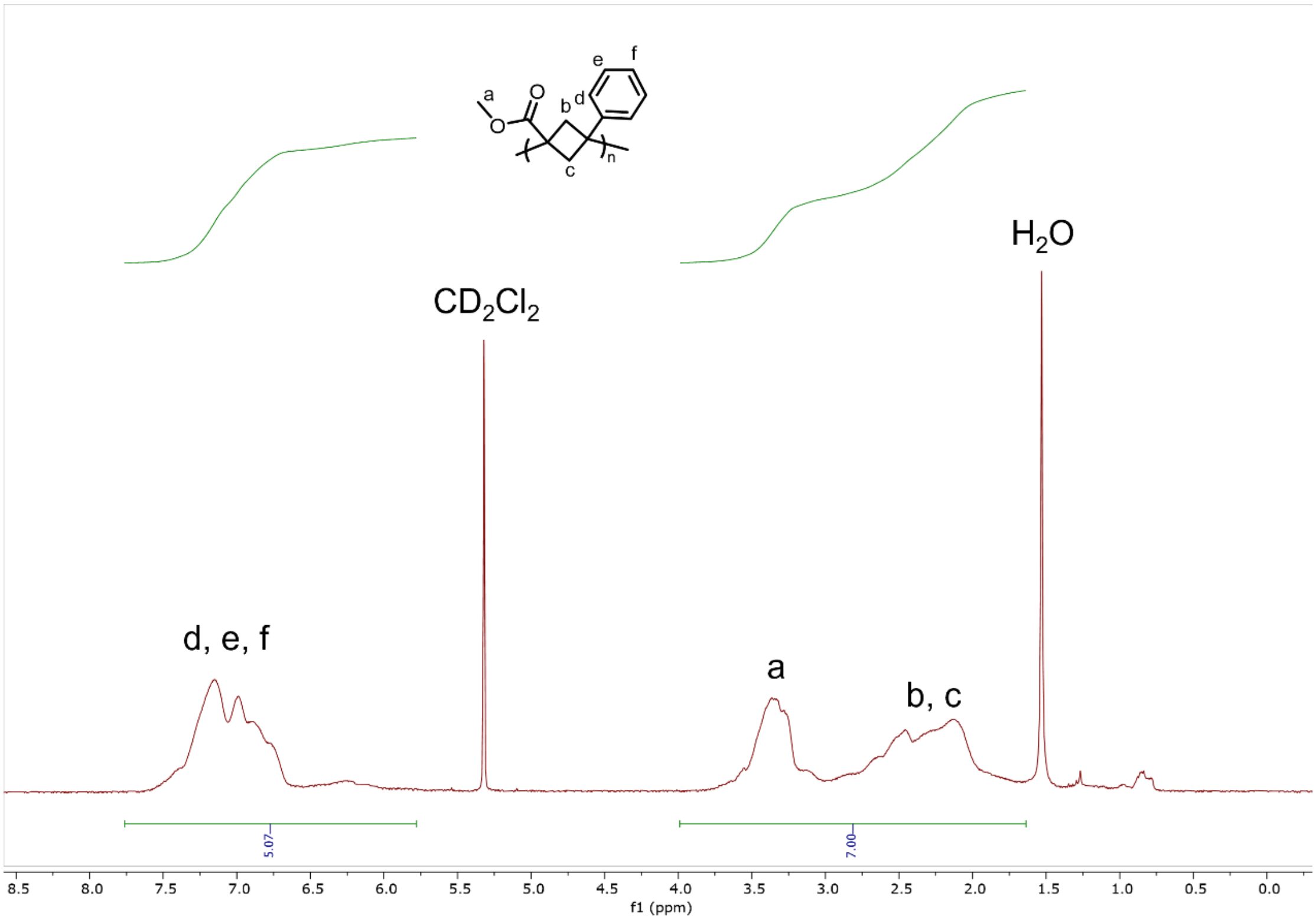

Figure S67. ^{1}H NMR spectrum of **P(BCB1).**

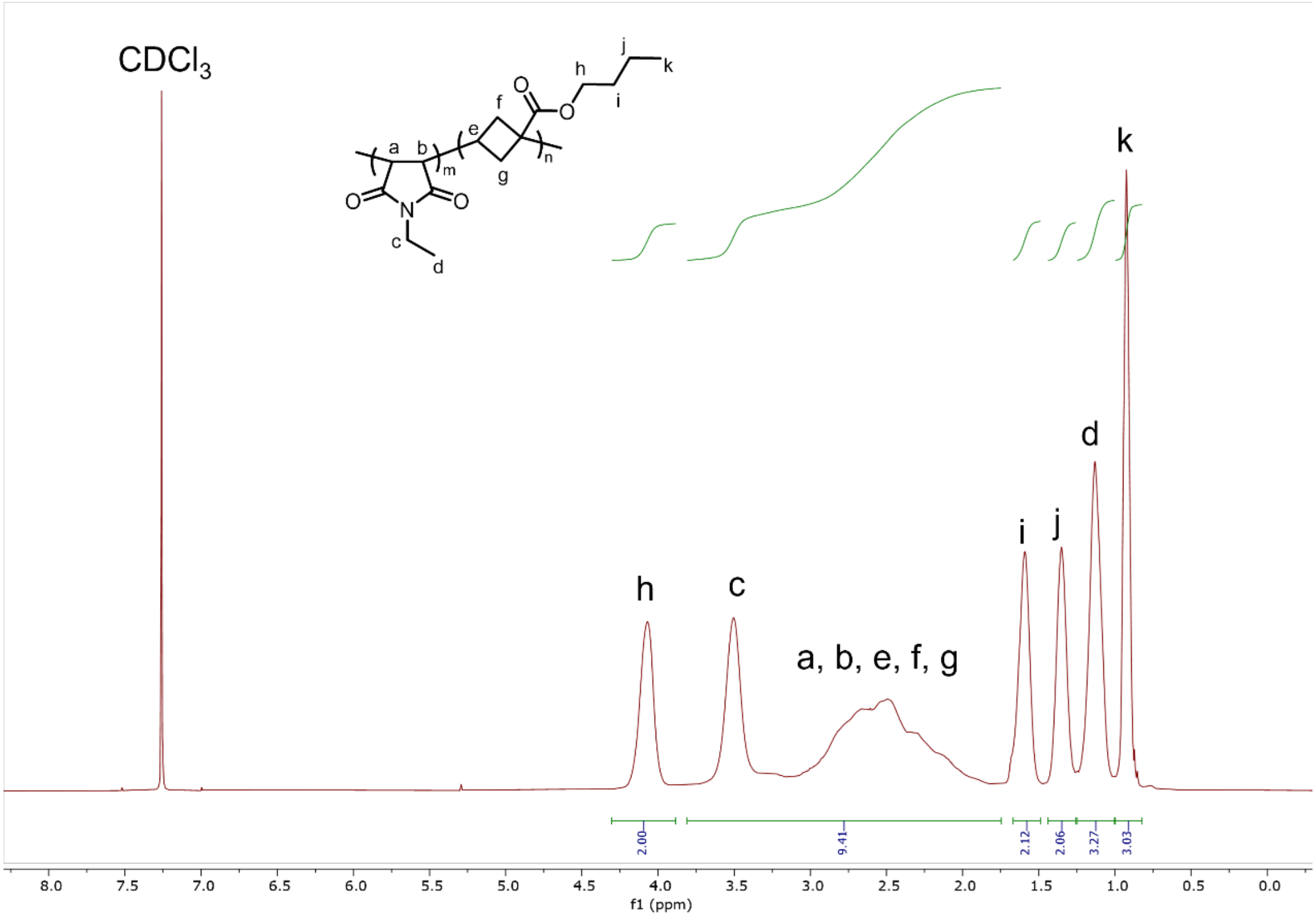

Figure S68. ^{1}H NMR spectrum of **P(BCB2-*r*-EtMI).**

18. MALDI-TOF MS spectra

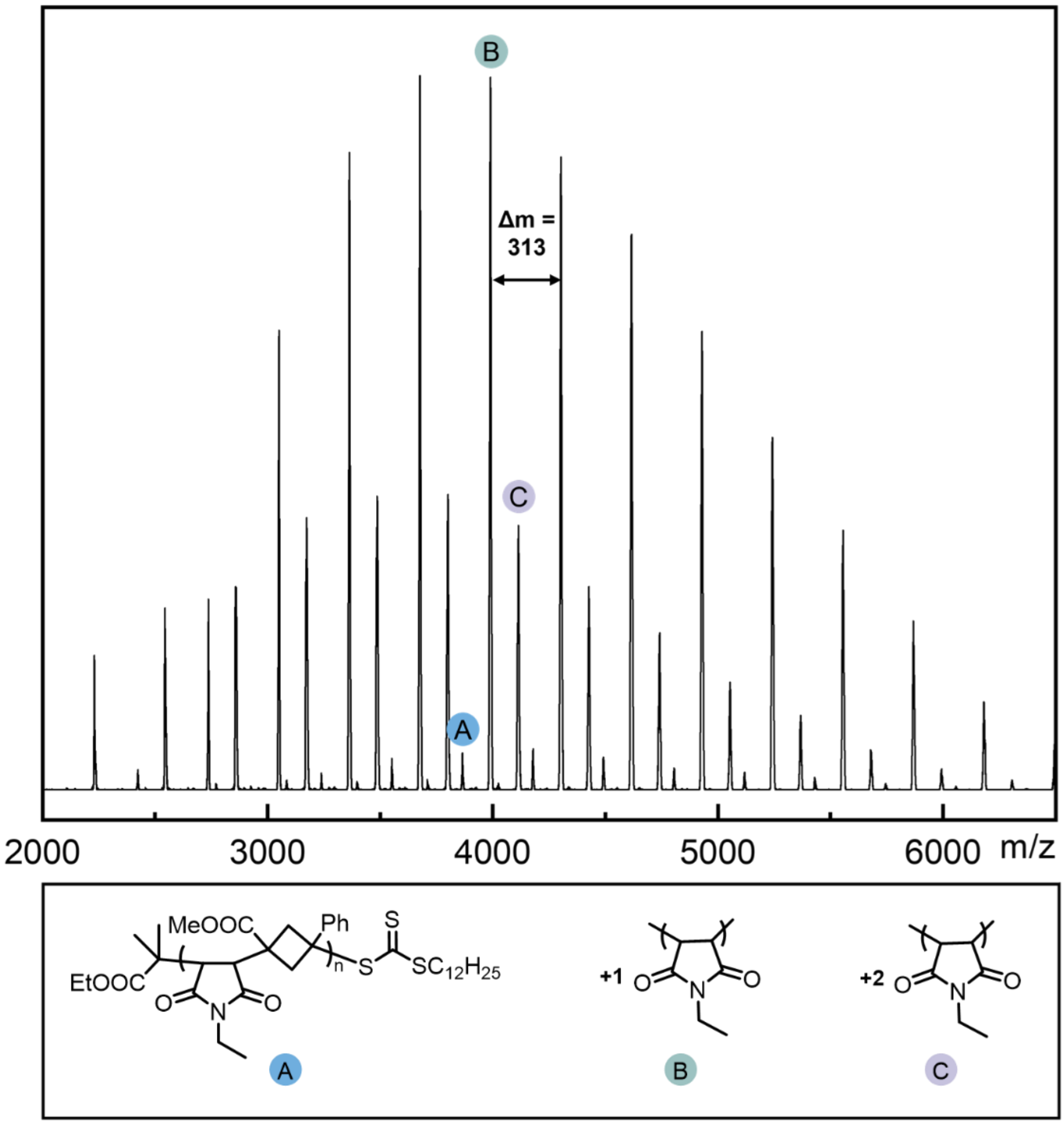

Figure S69. MALDI-TOF MS spectrum of **P1.**

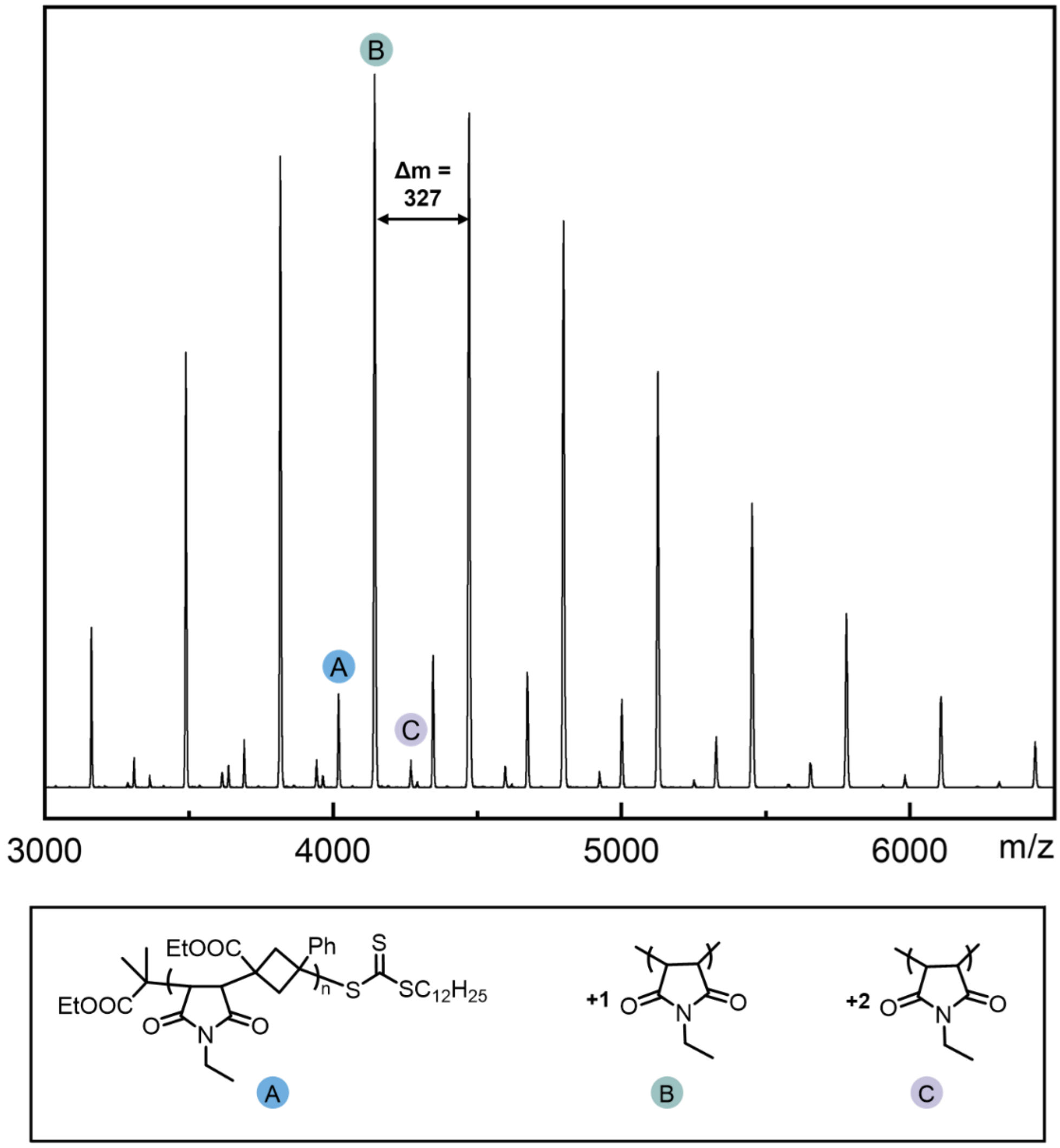

Figure S70. MALDI-TOF MS spectrum of **P2.**

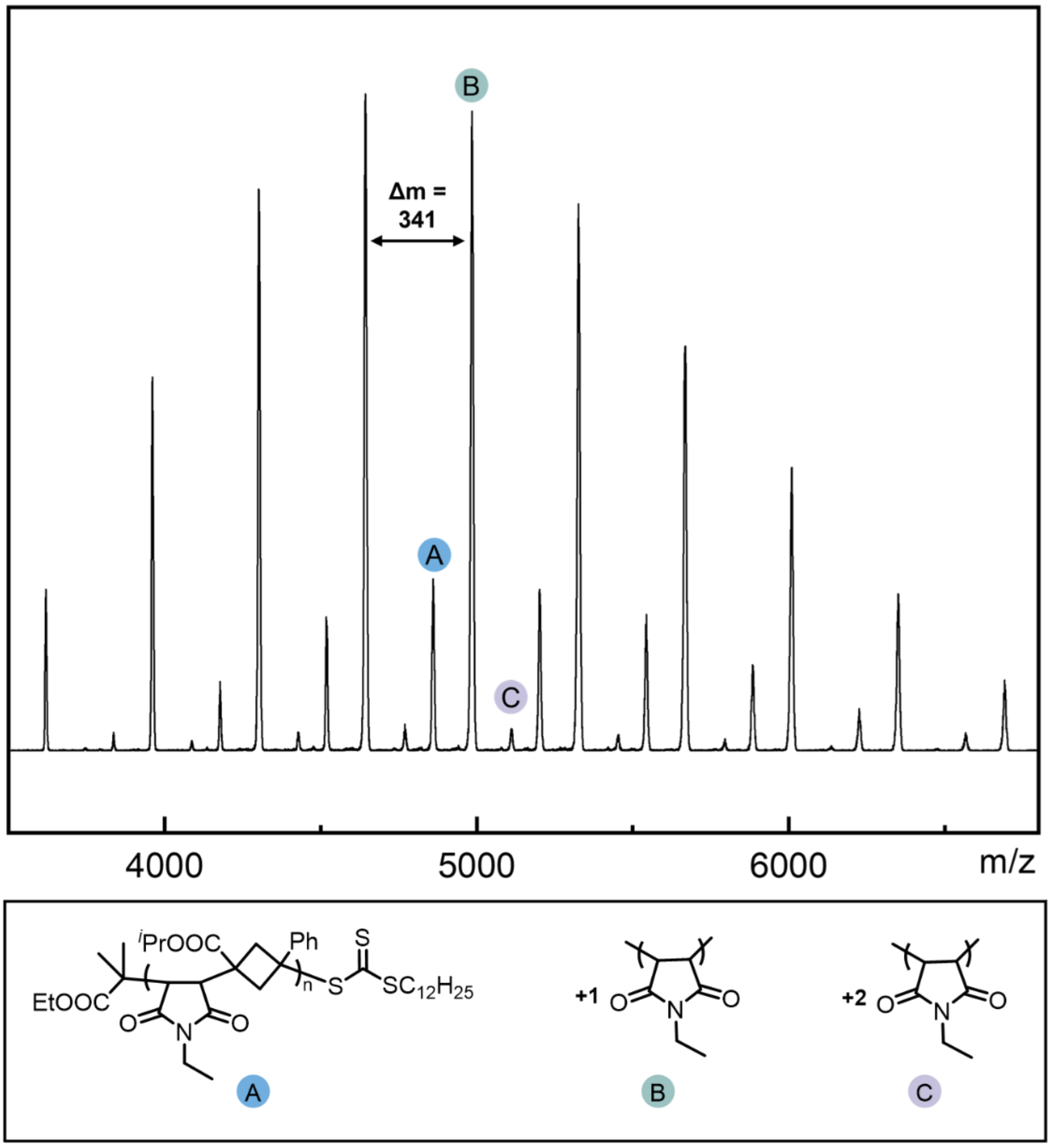

Figure S71. MALDI-TOF MS spectrum of **P3.**

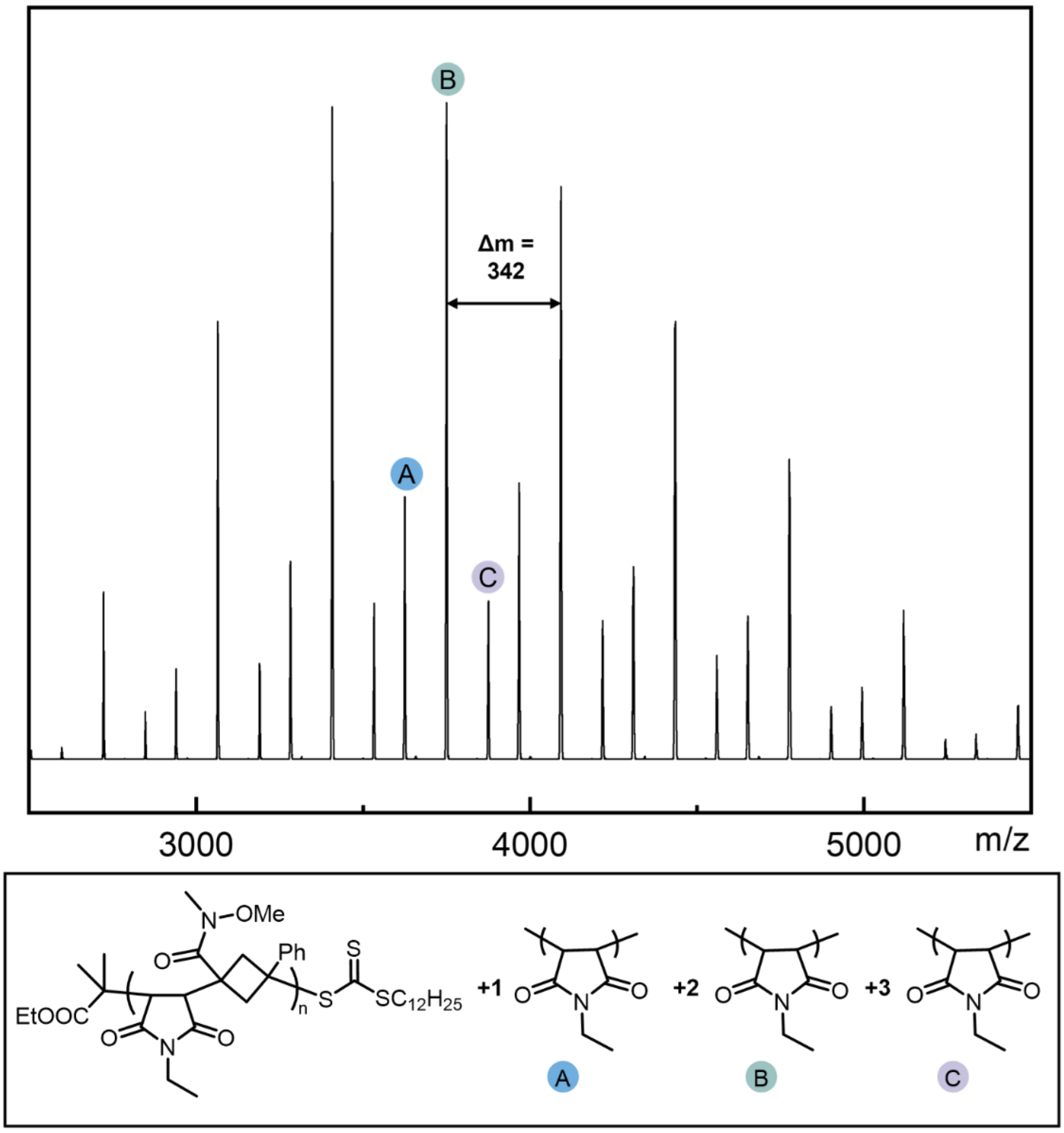

Figure S72. MALDI-TOF MS spectrum of **P4.**

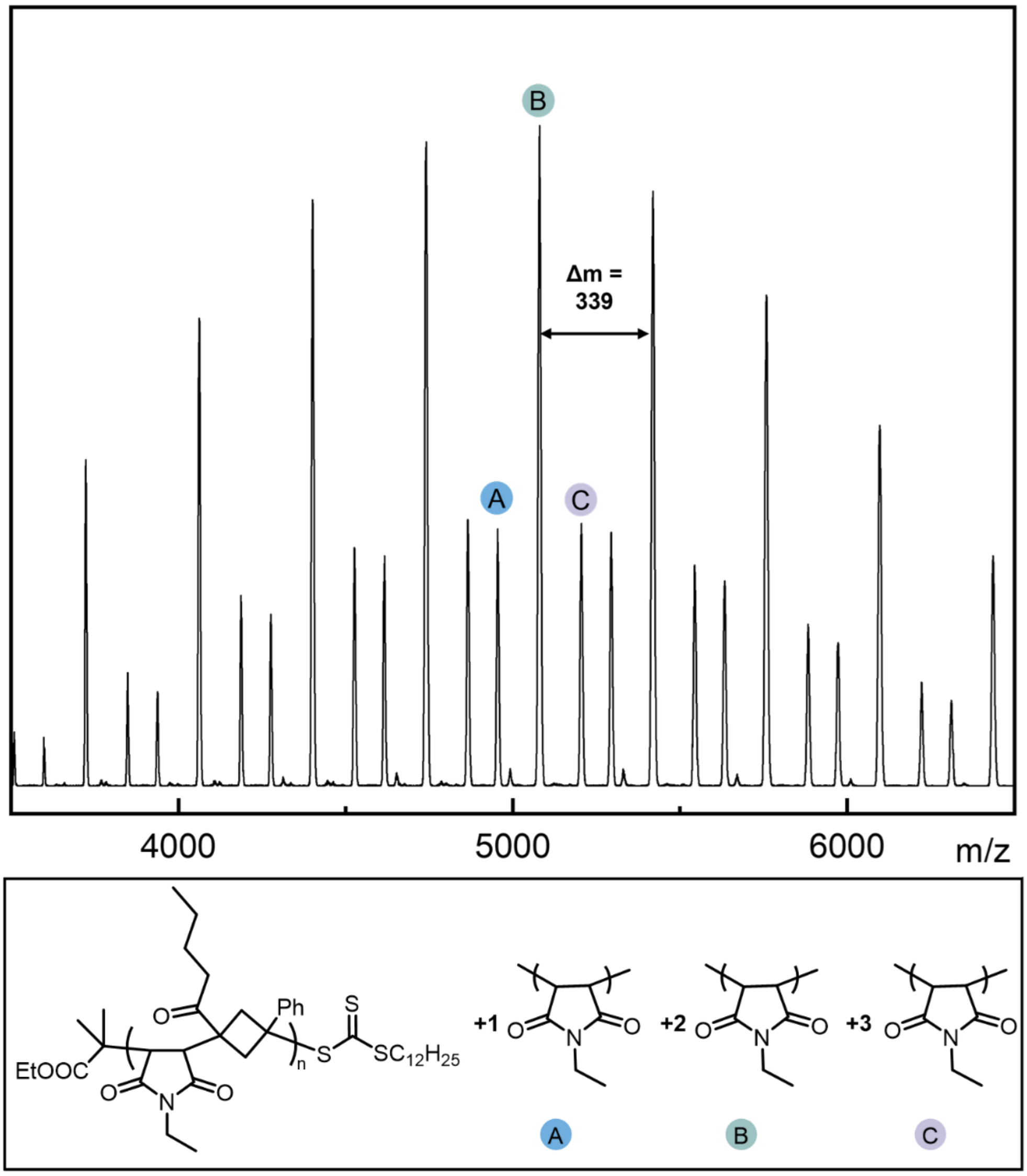

Figure S73. MALDI-TOF MS spectrum of **P5.**

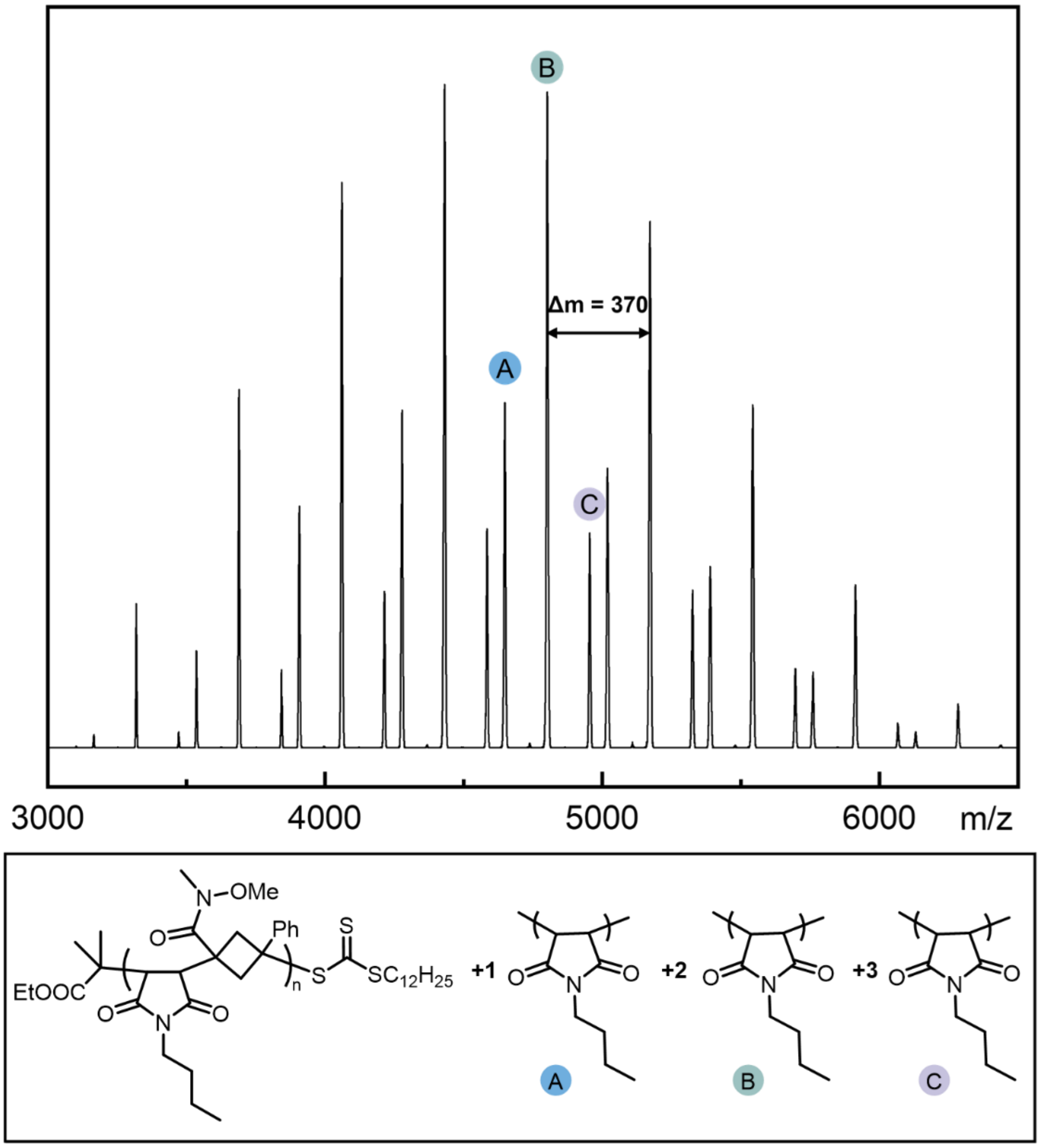

Figure S74. MALDI-TOF MS spectrum of **P6.**

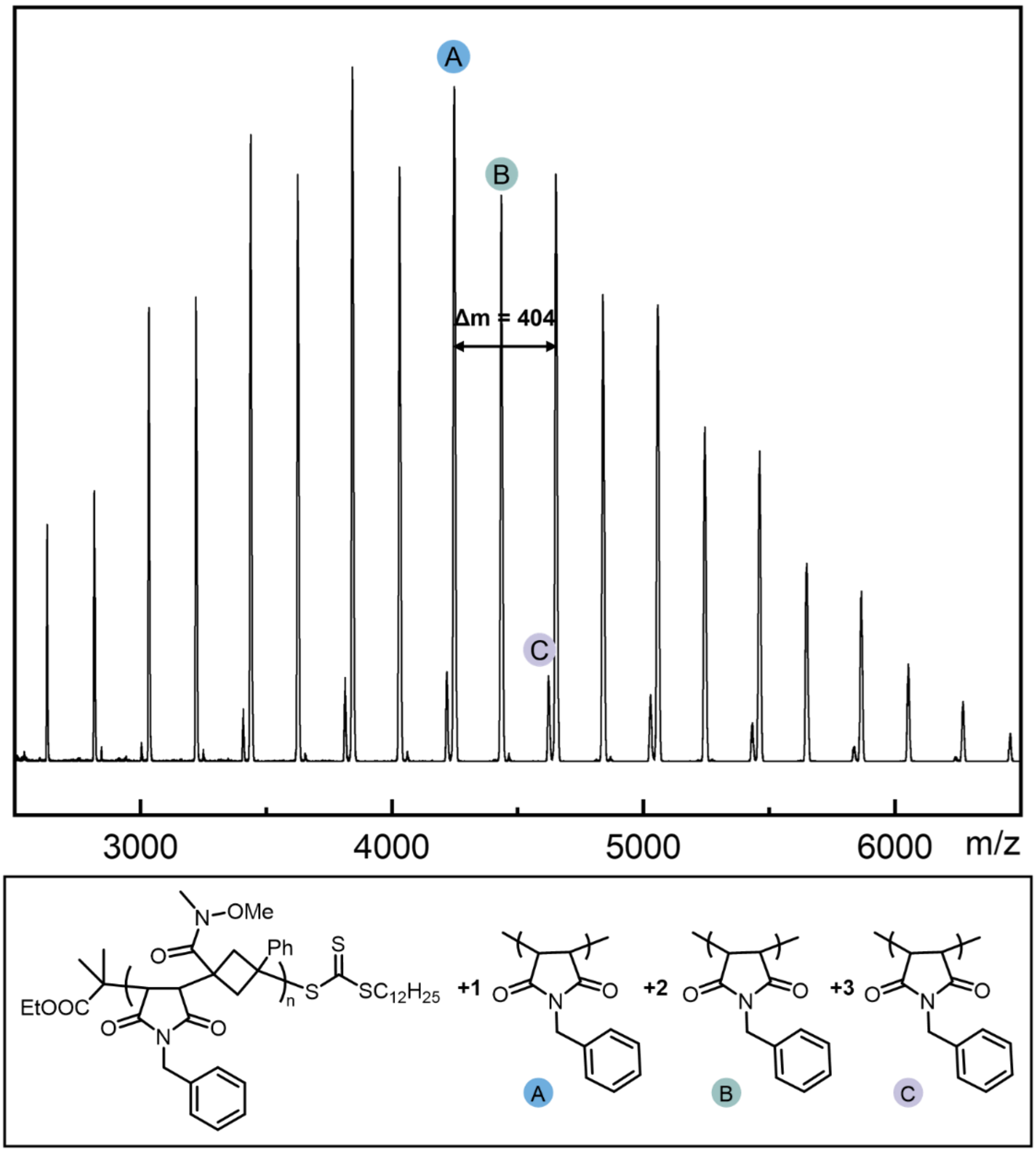

Figure S75. MALDI-TOF MS spectrum of **P7.**

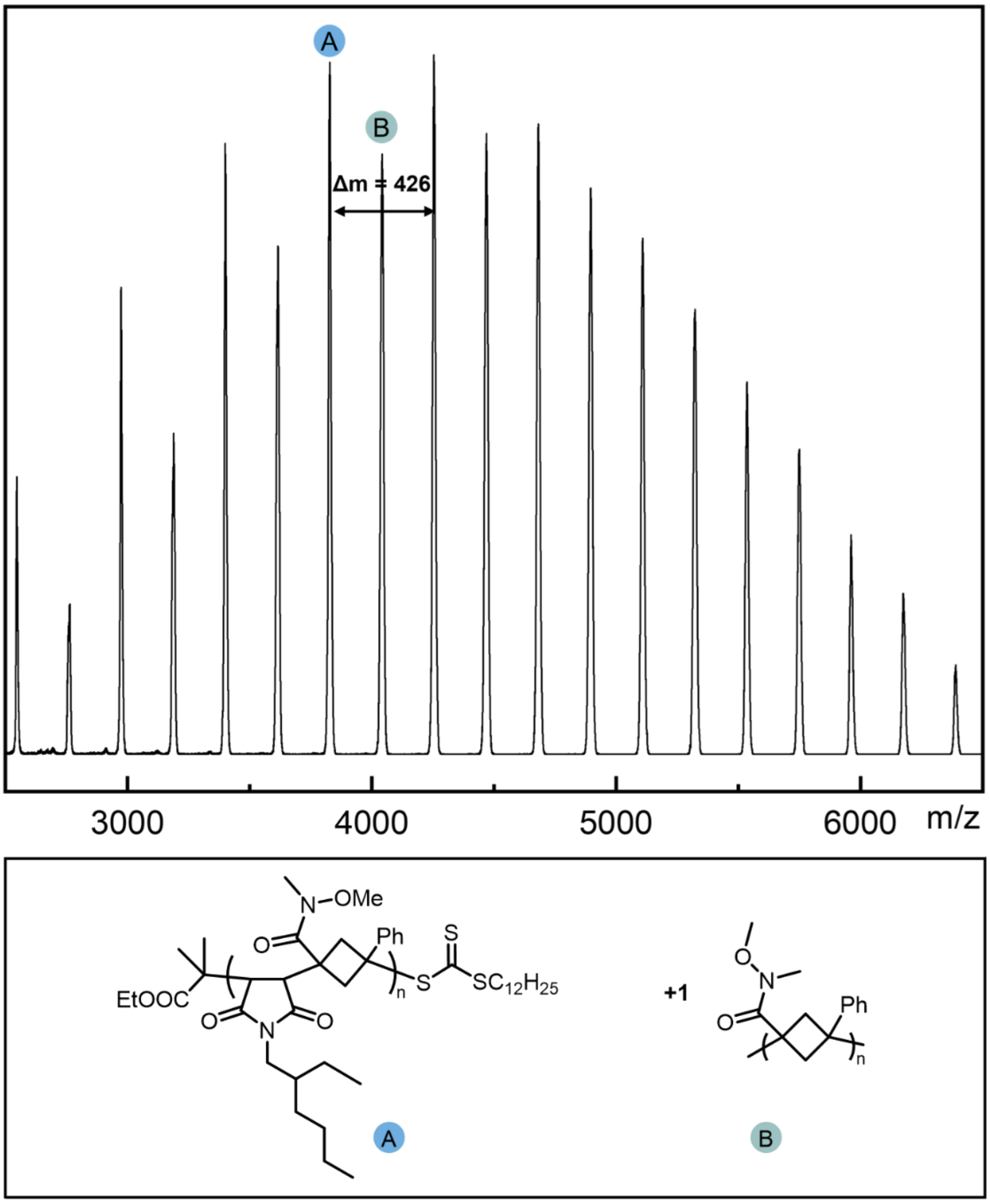

Figure S76. MALDI-TOF MS spectrum of **P8.**

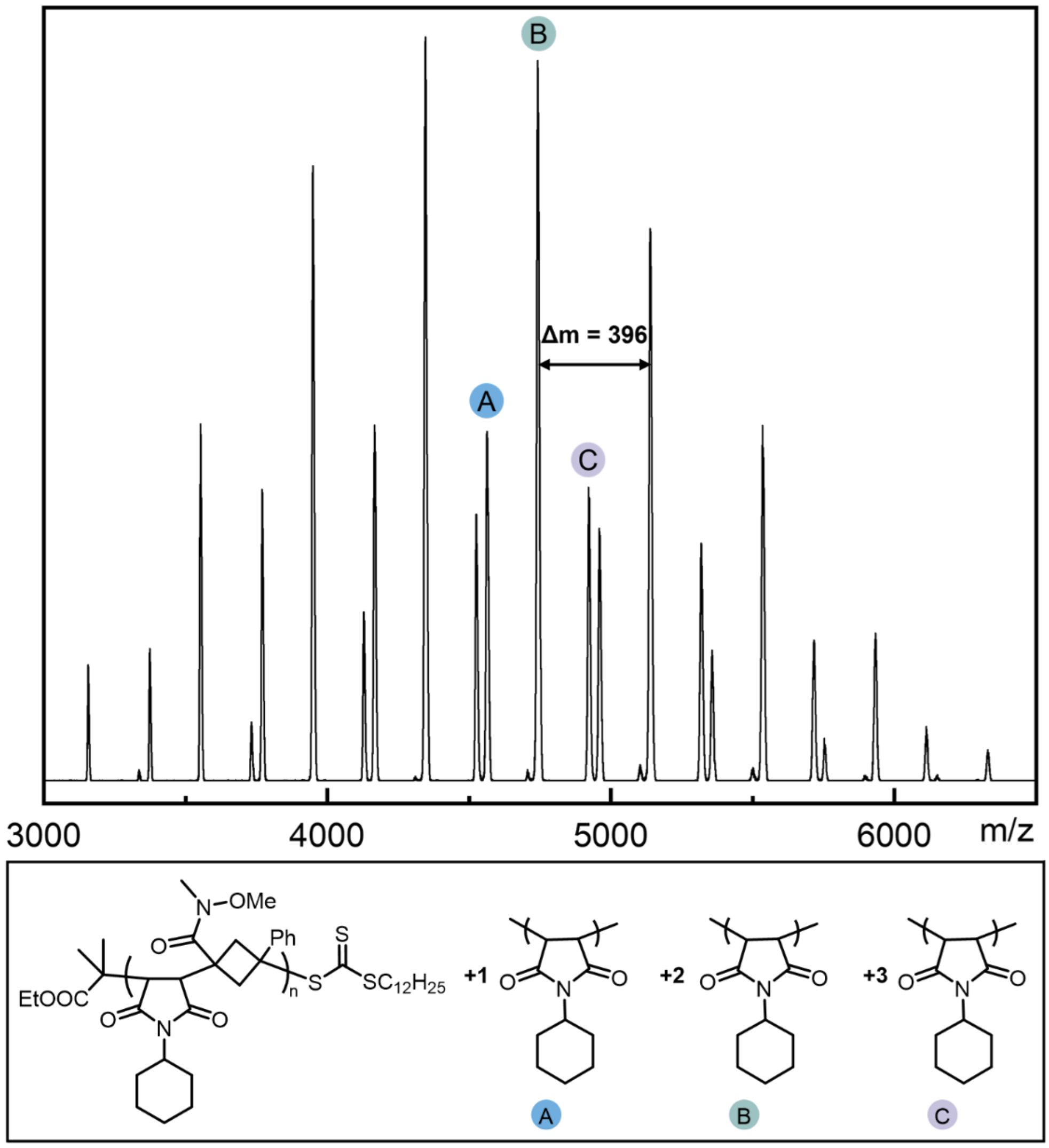

Figure S77. MALDI-TOF MS spectrum of **P9.**

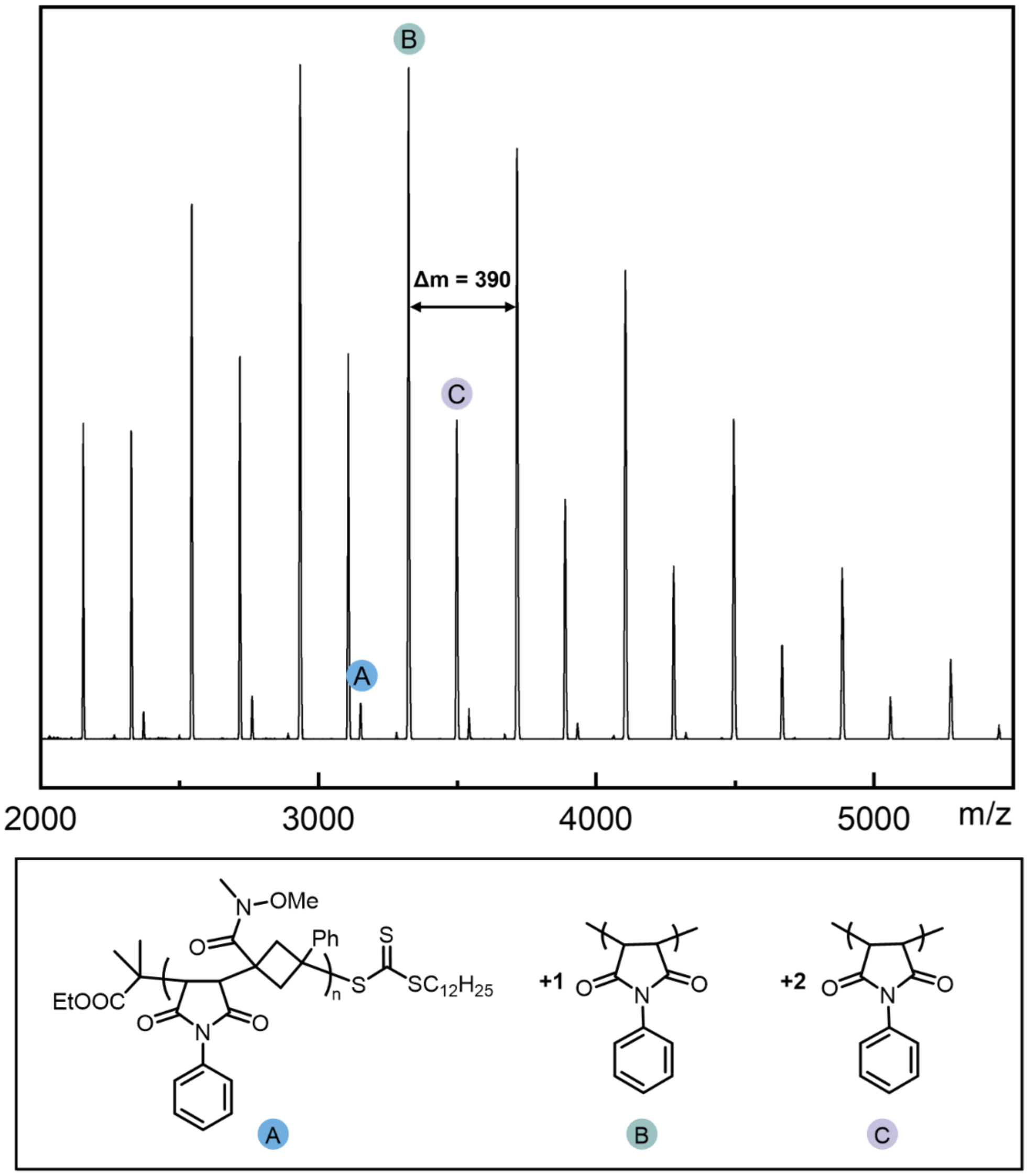

Figure S78. MALDI-TOF MS spectrum of **P10.**

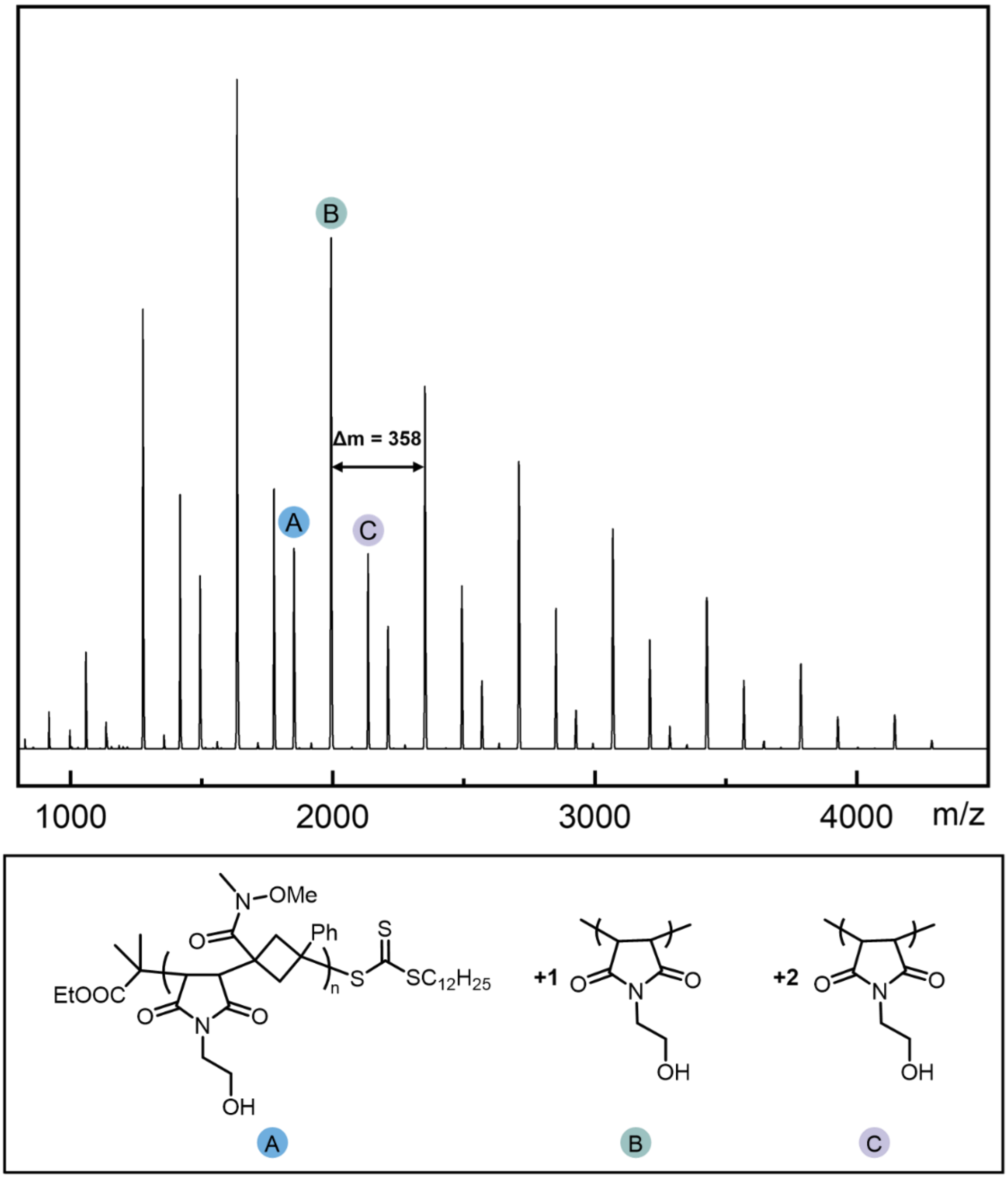

Figure S79. MALDI-TOF MS spectrum of **P11.**

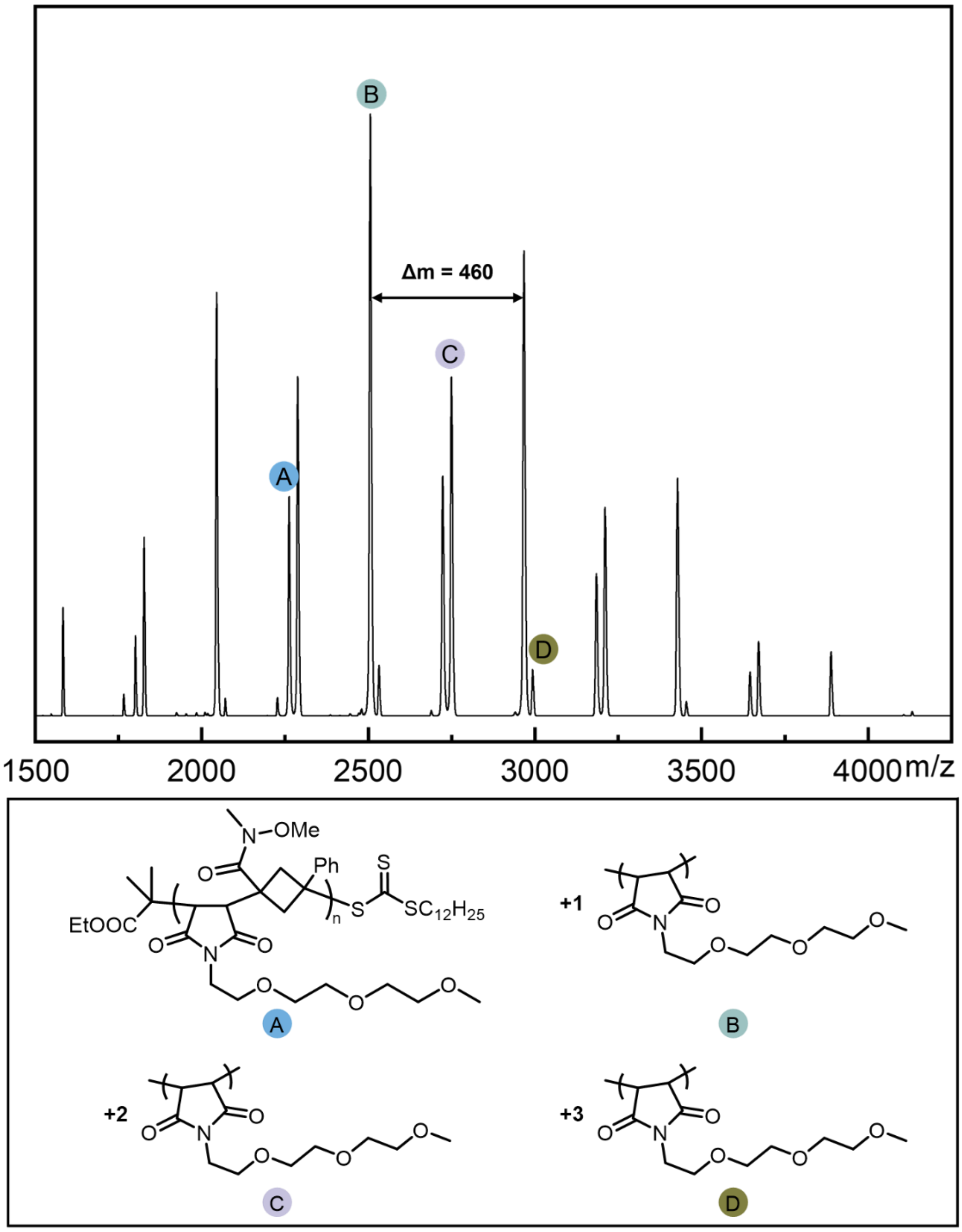

Figure S80. MALDI-TOF MS spectrum of **P12.**

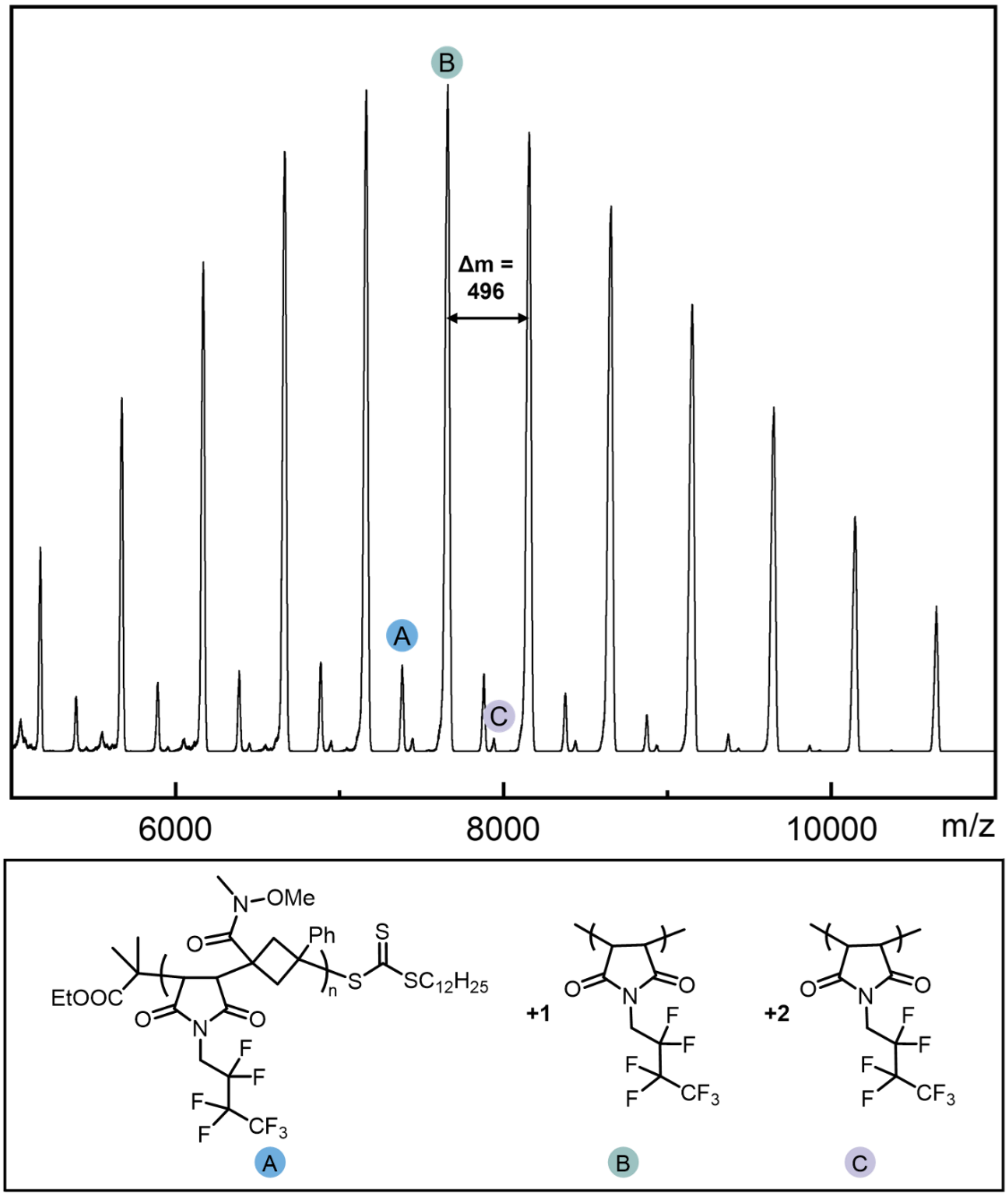

Figure S81. MALDI-TOF MS spectrum of **P14.**

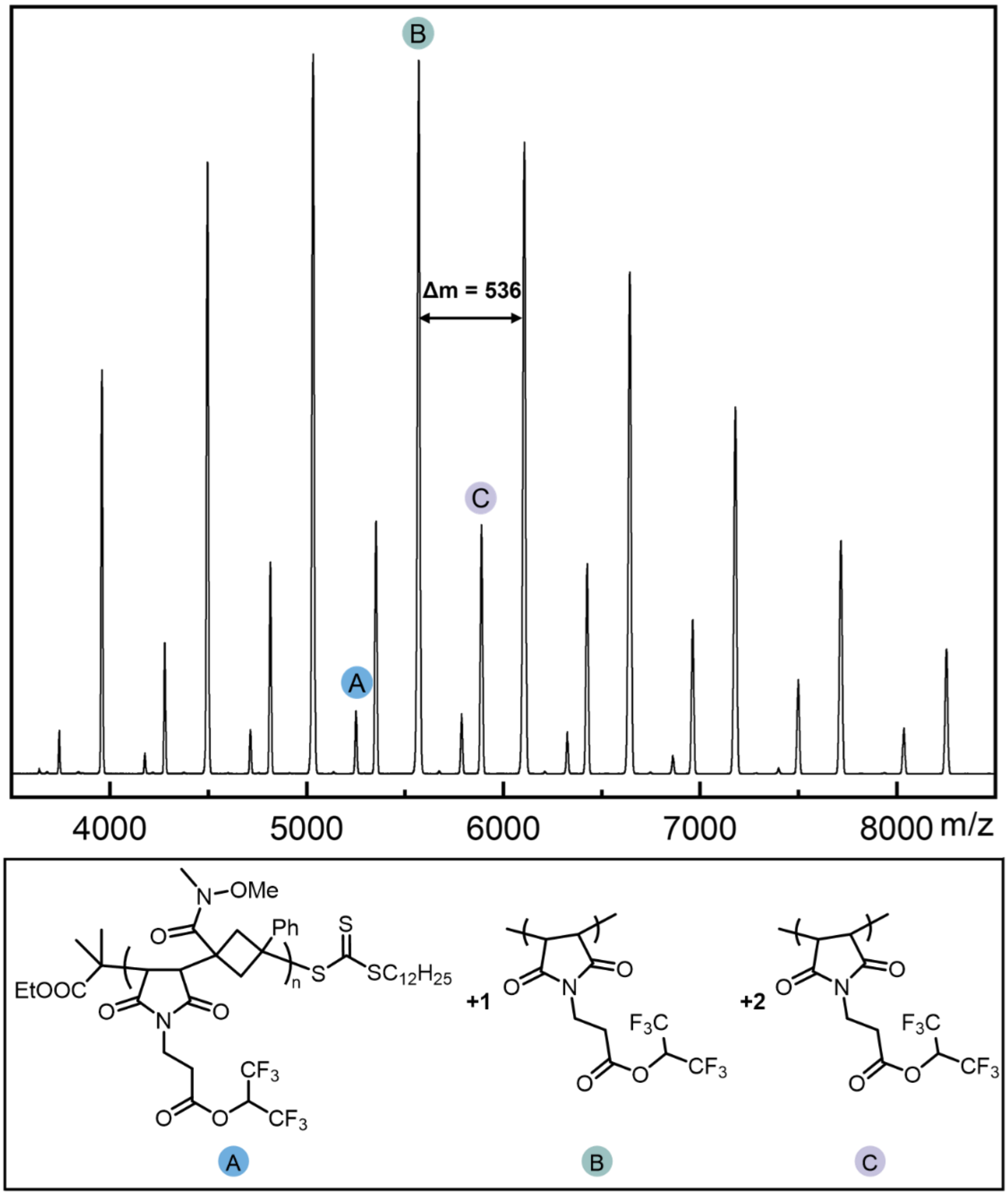

Figure S82. MALDI-TOF MS spectrum of **P15.**

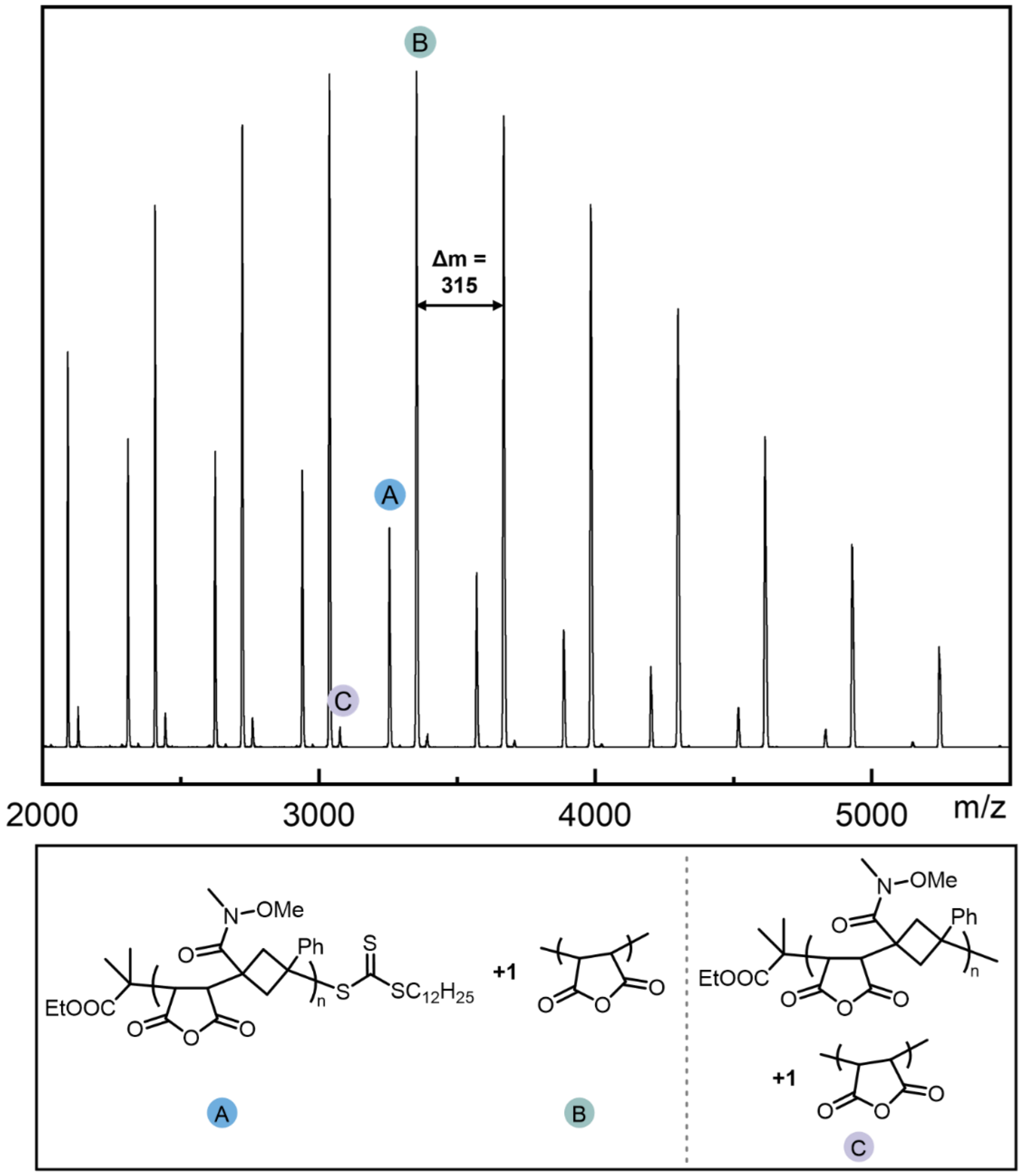

Figure S83. MALDI-TOF MS spectrum of **P16.**

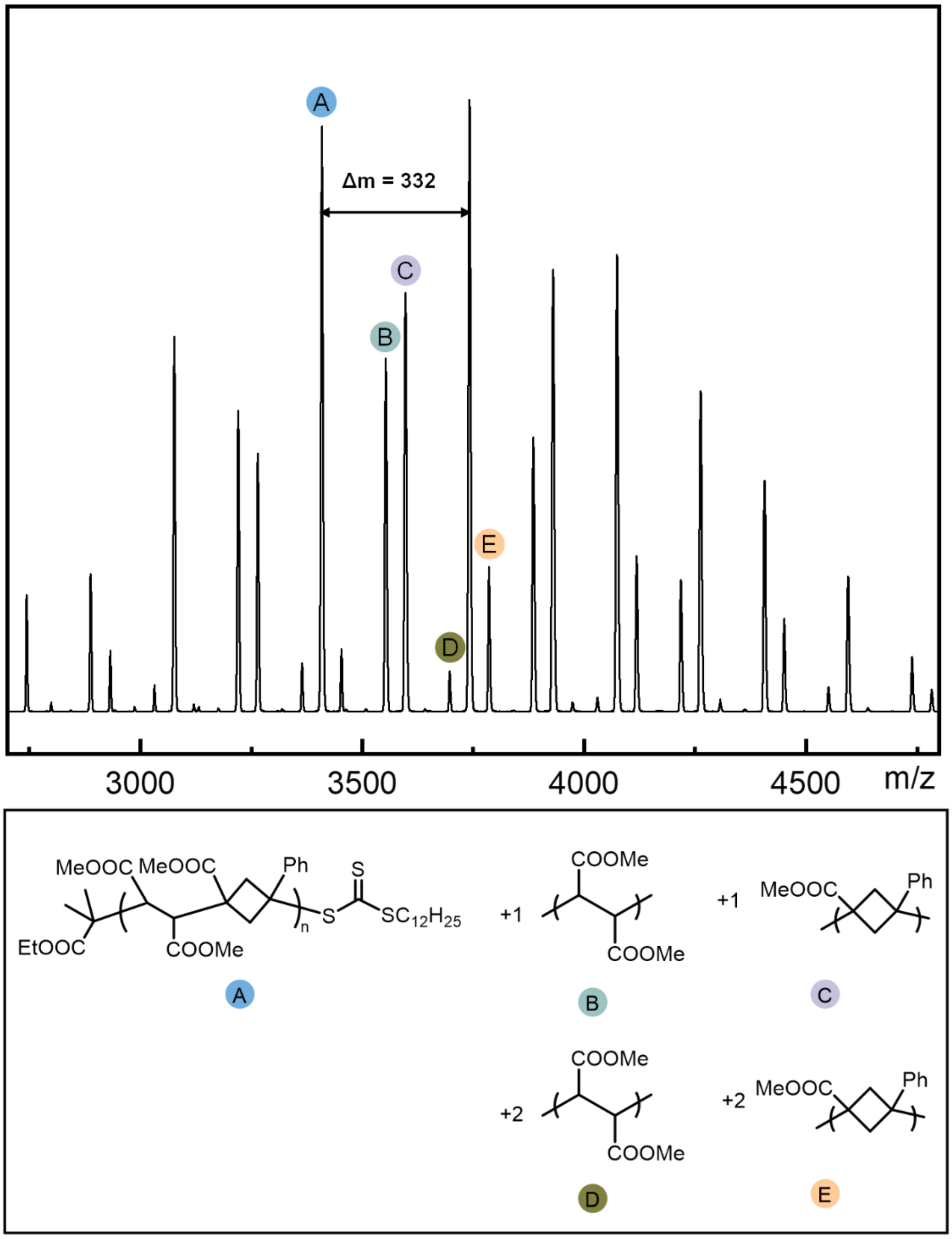

Figure S84. MALDI-TOF MS spectrum of **P17.**

19. SEC traces

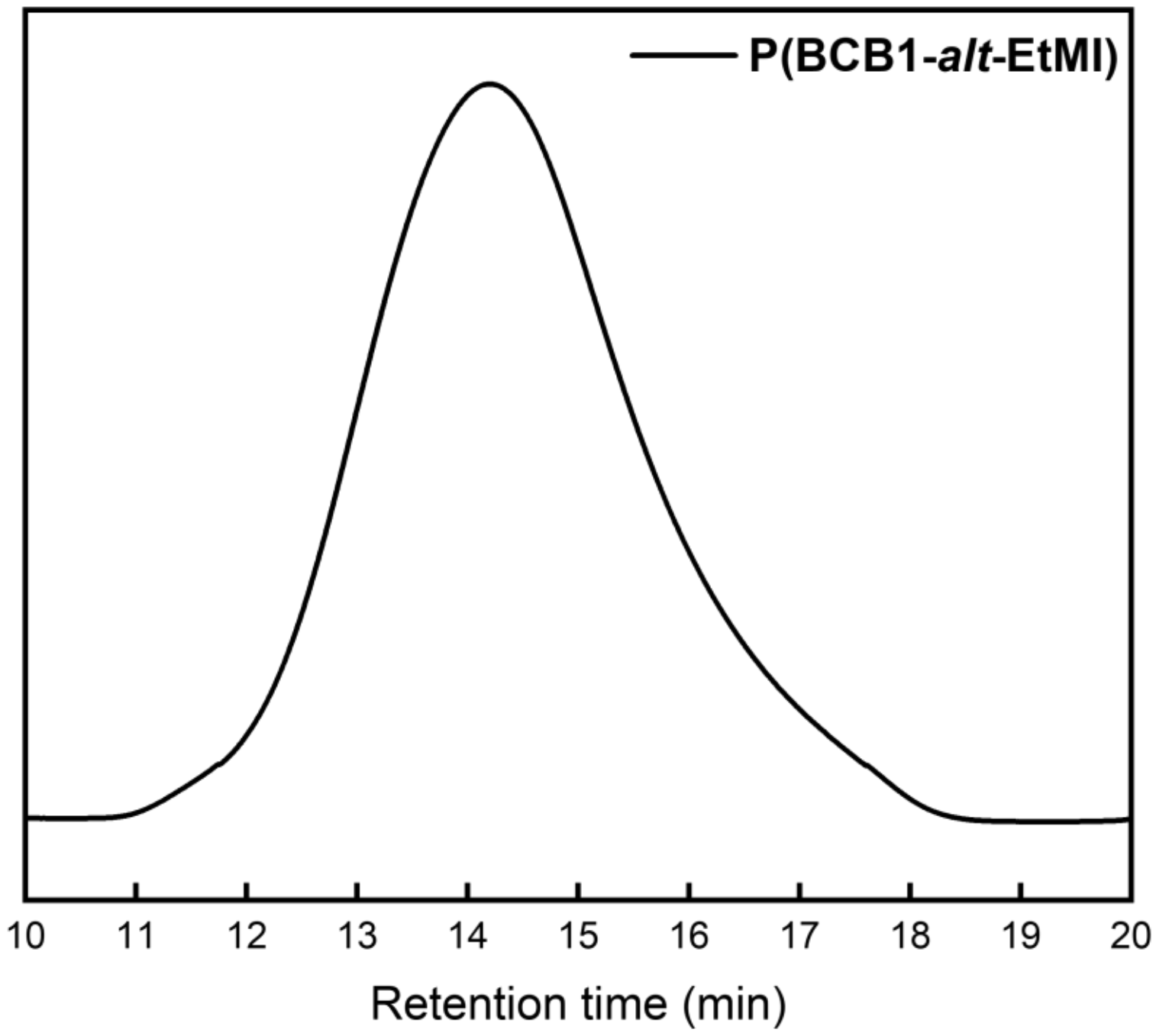

Figure S85. SEC trace (THF) of **P(BCB1-alt-EtMI)**.

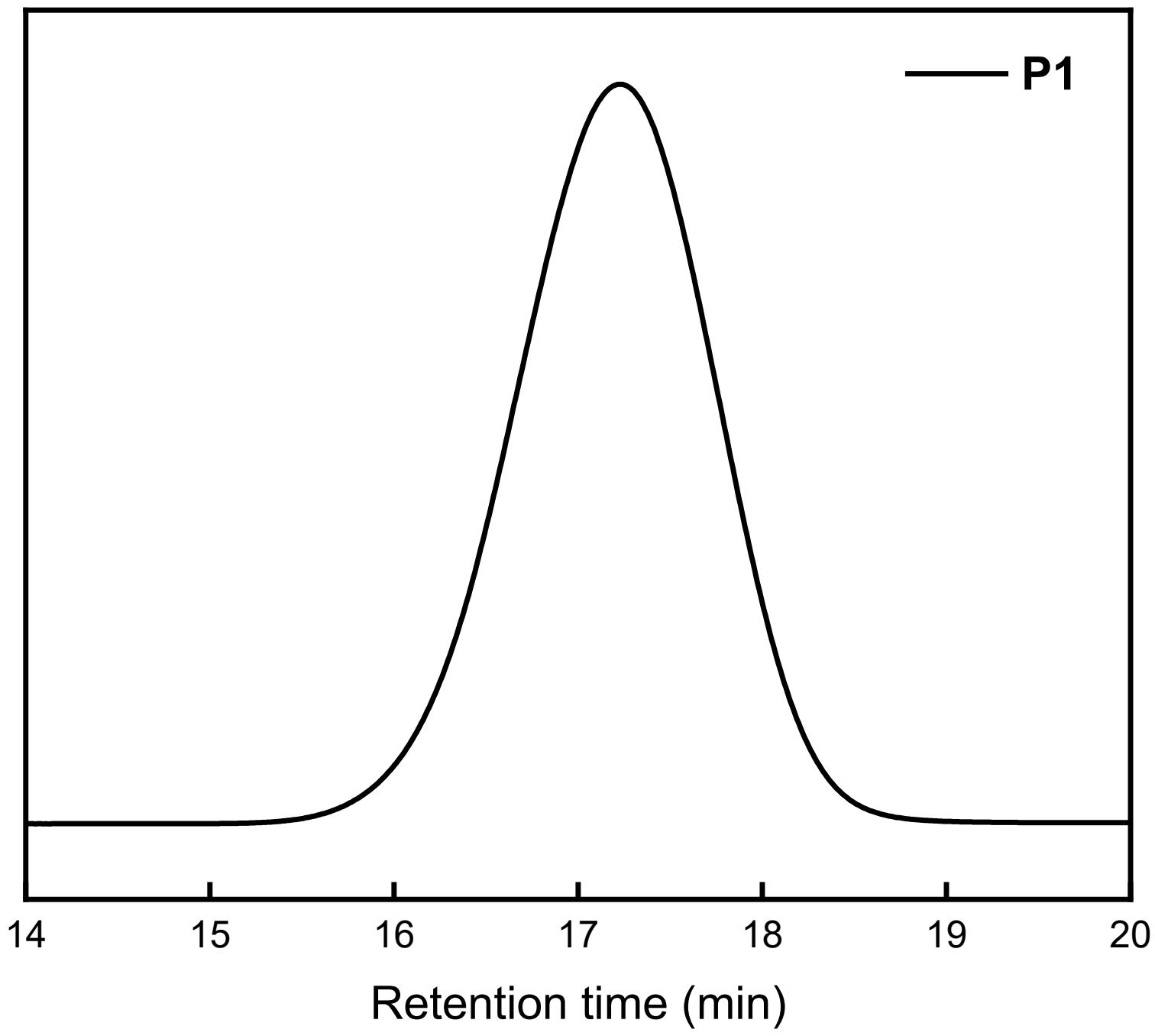

Figure S86. SEC trace (THF) of **P1**.

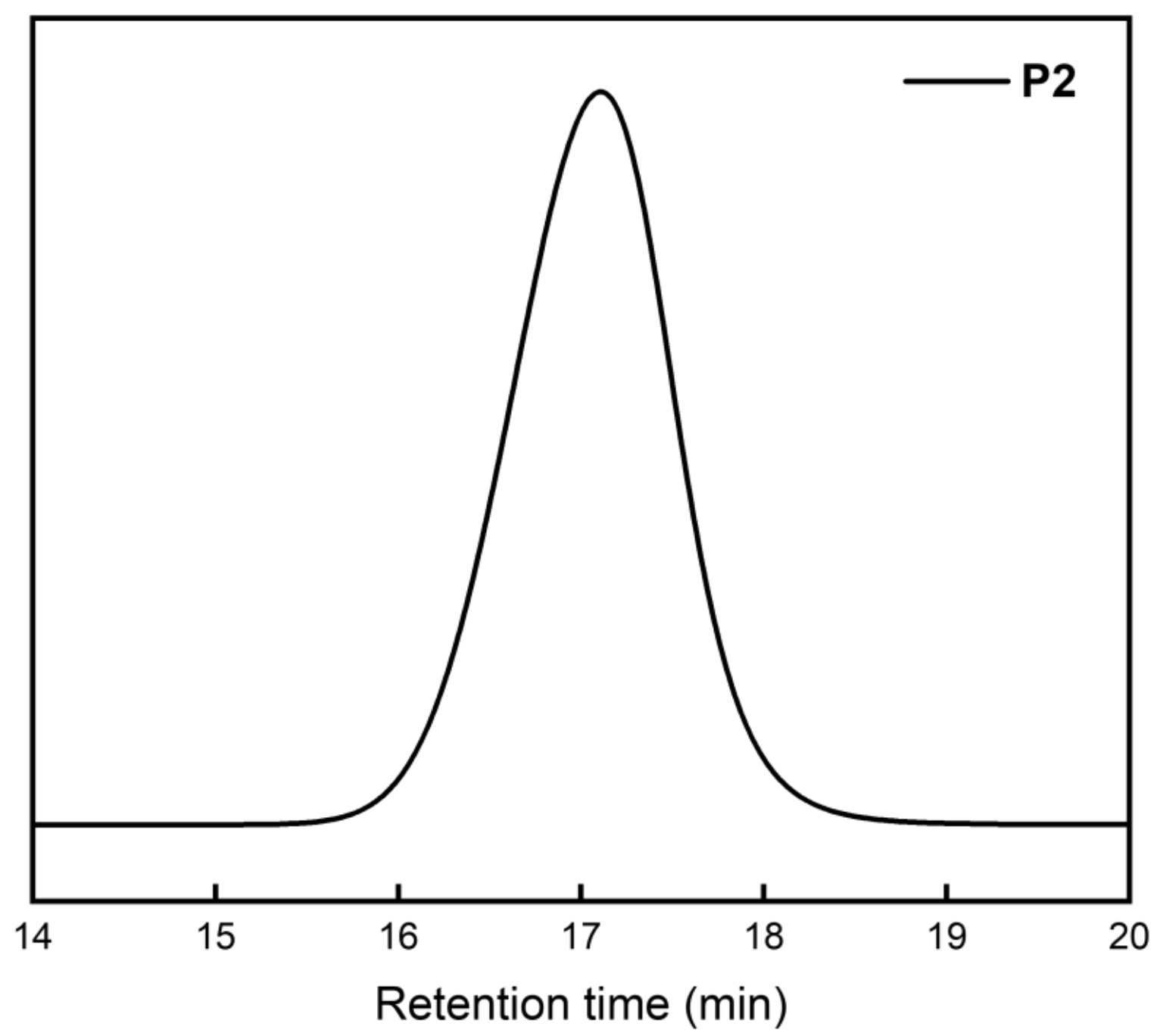

Figure S87. SEC trace (THF) of **P2**.

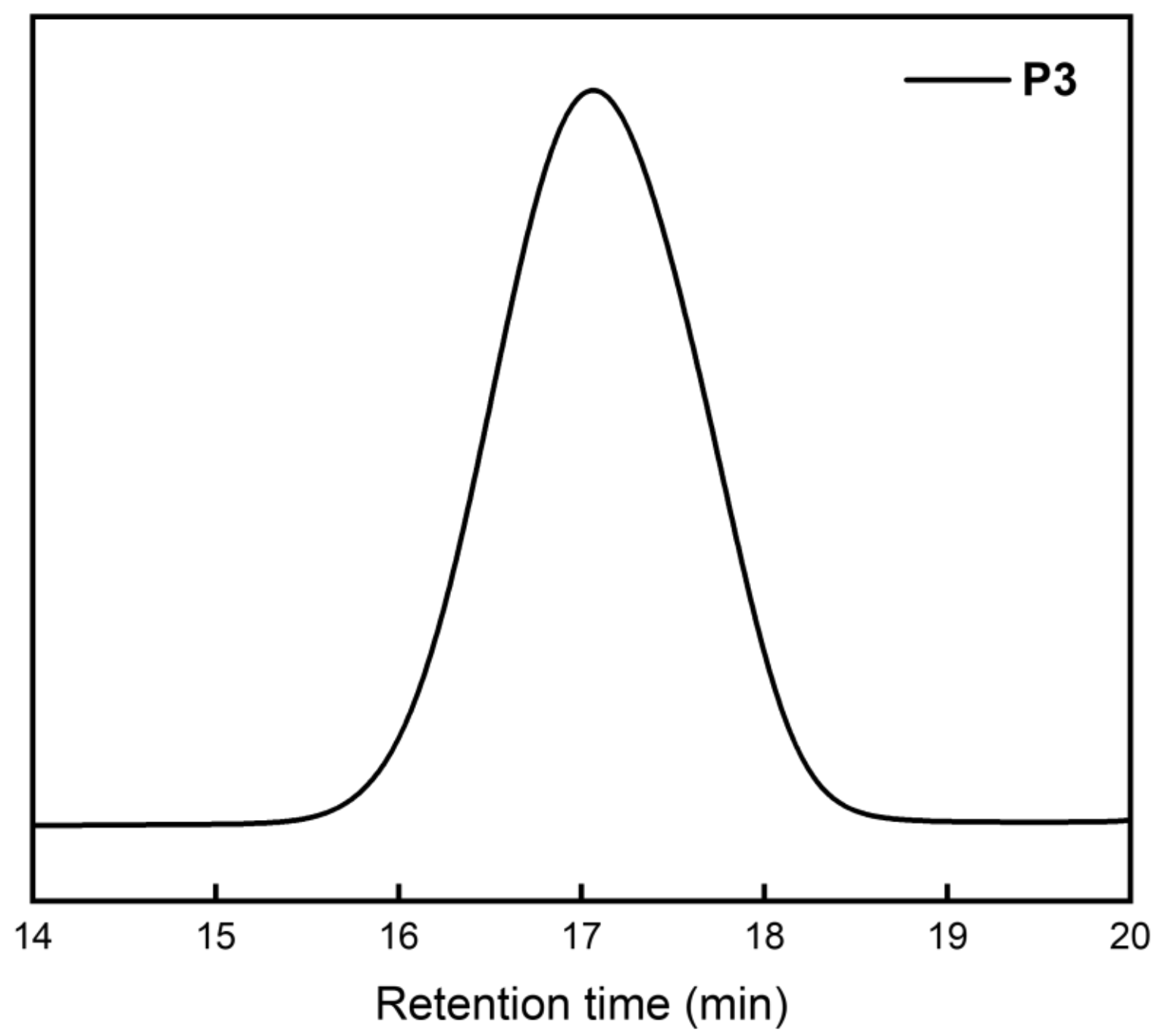

Figure S88. SEC trace (THF) of **P3**.

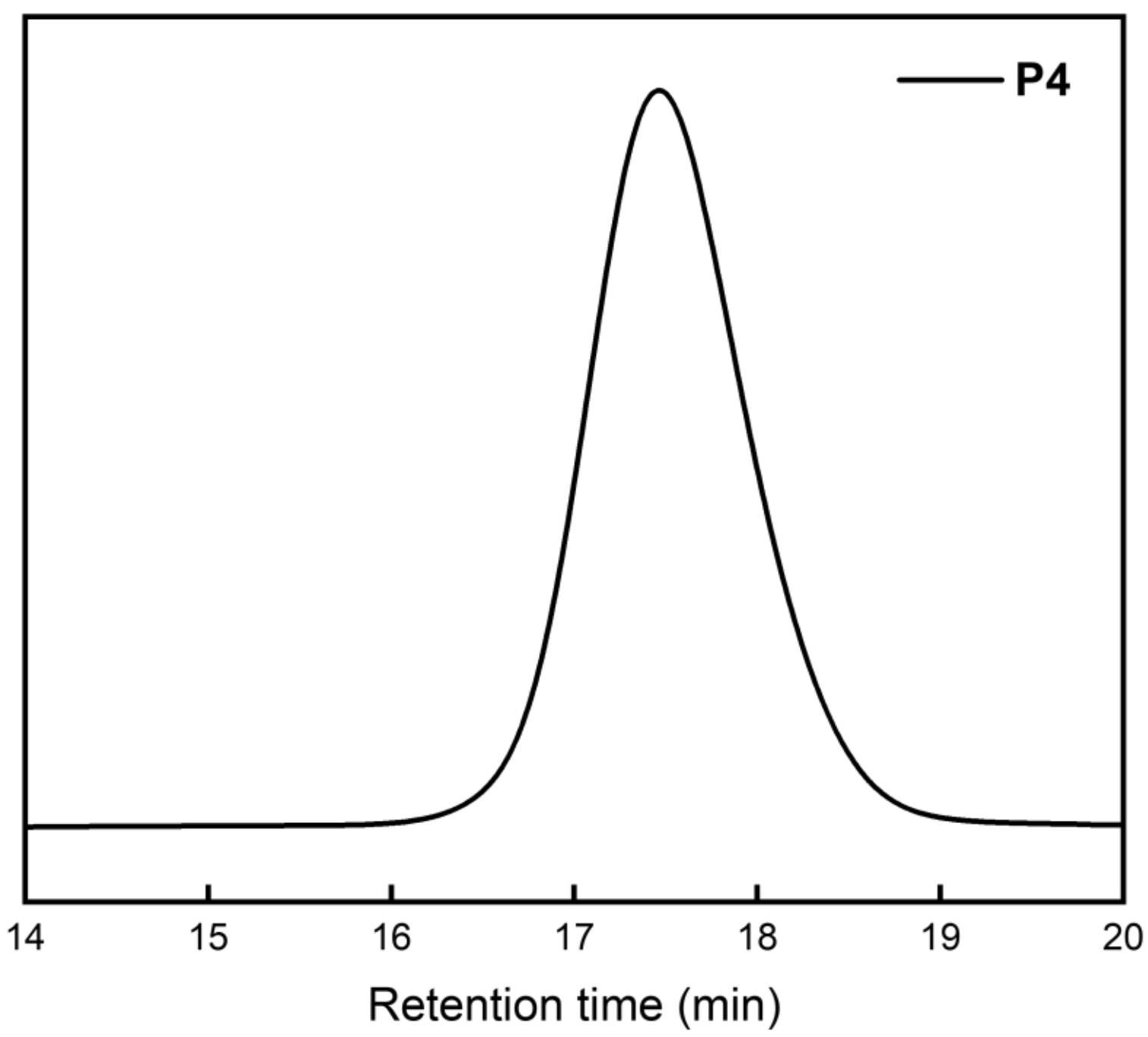

Figure S89. SEC trace (THF) of **P4**.

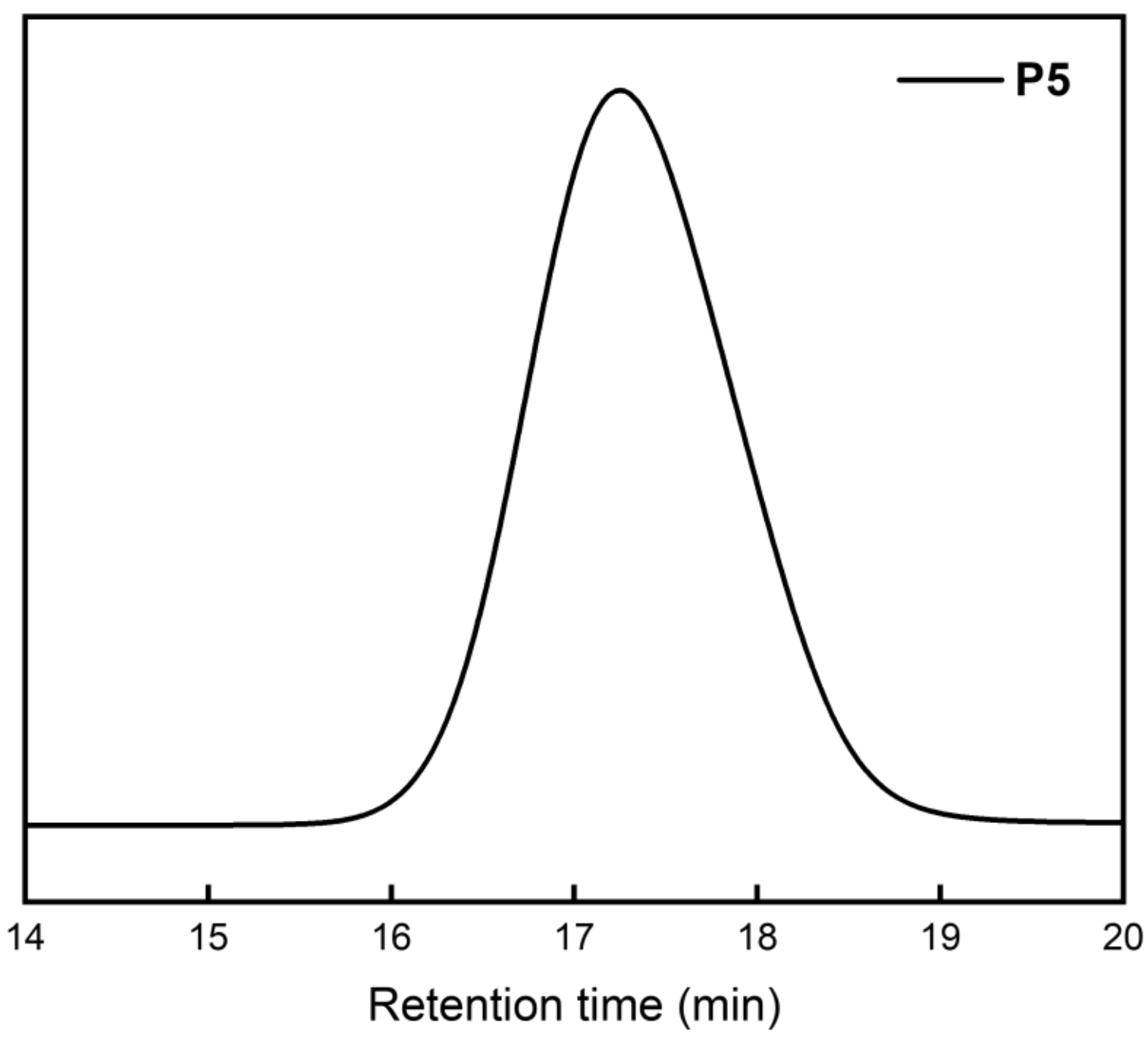

Figure S90. SEC trace (THF) of **P5**.

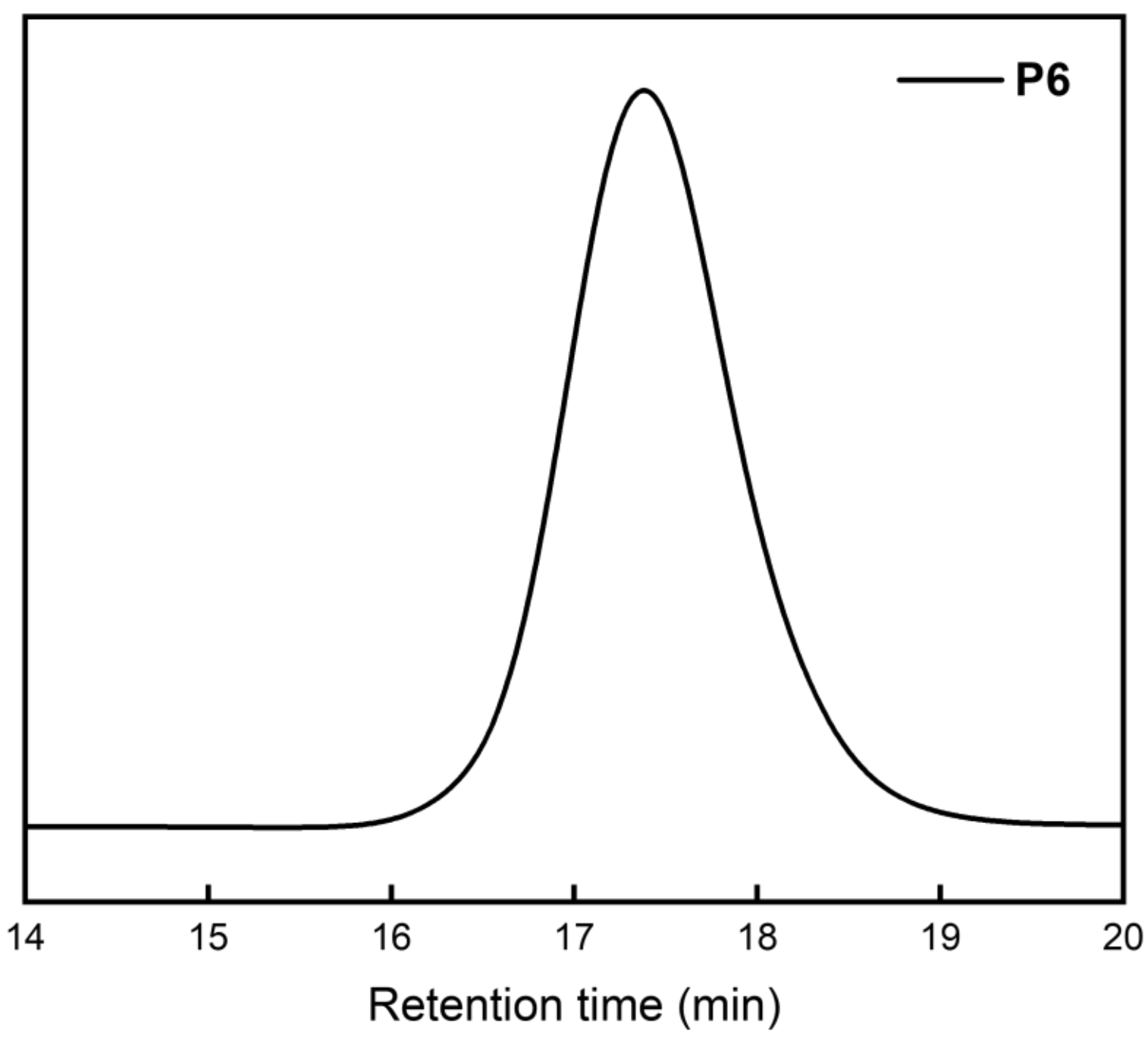

Figure S91. SEC trace (THF) of **P6**.

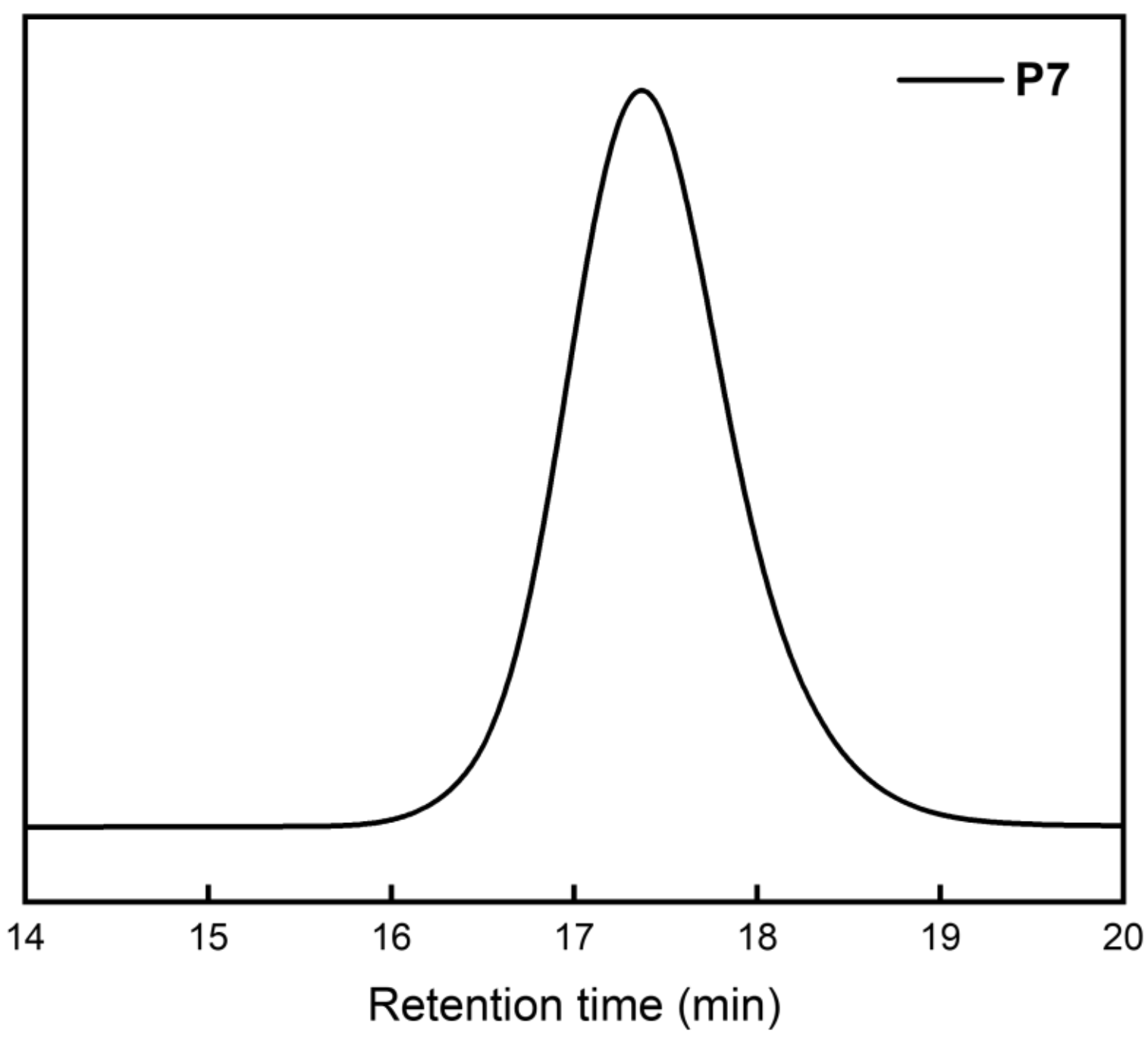

Figure S92. SEC trace (THF) of **P7**.

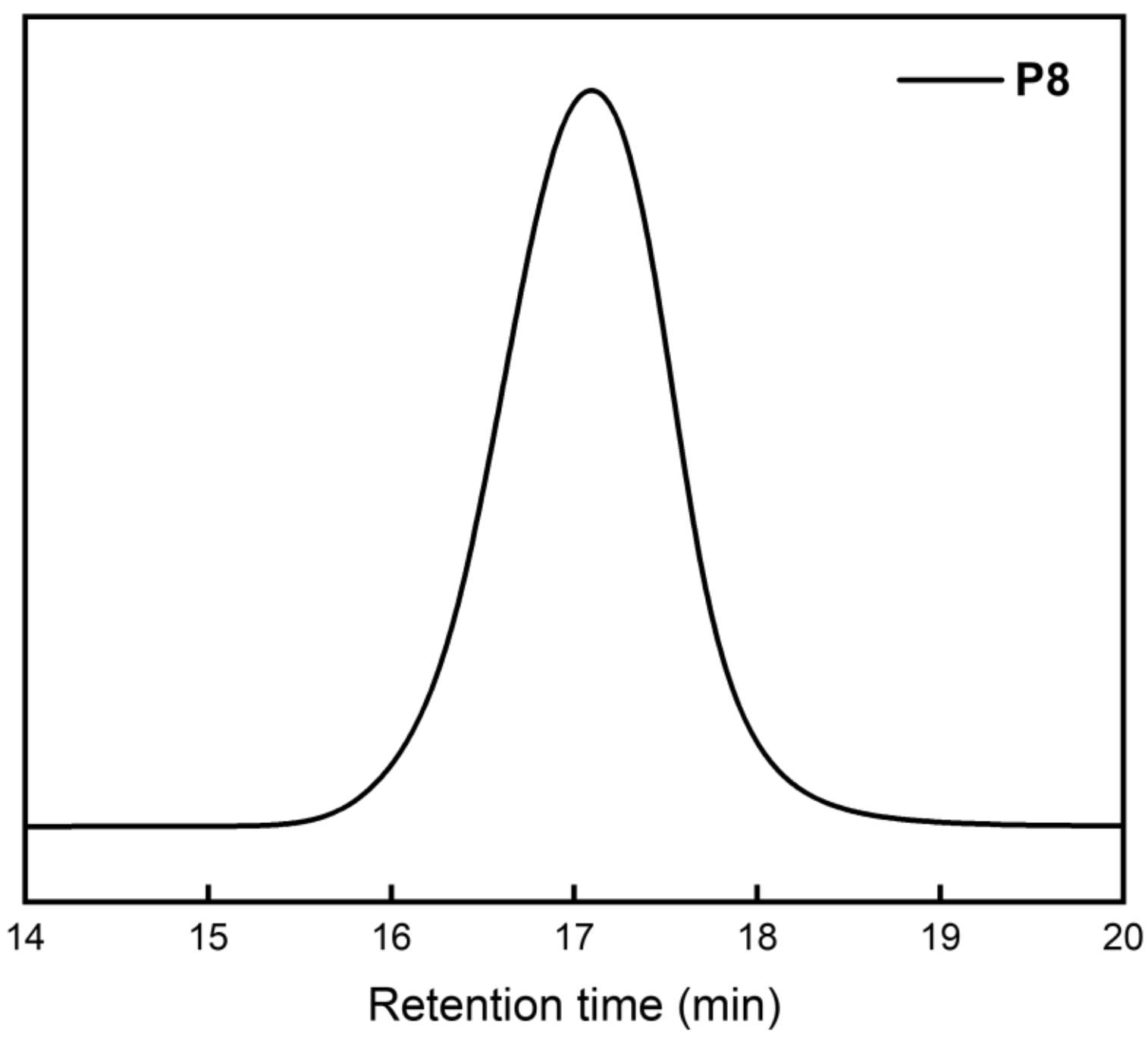

Figure S93. SEC trace (THF) of **P8**.

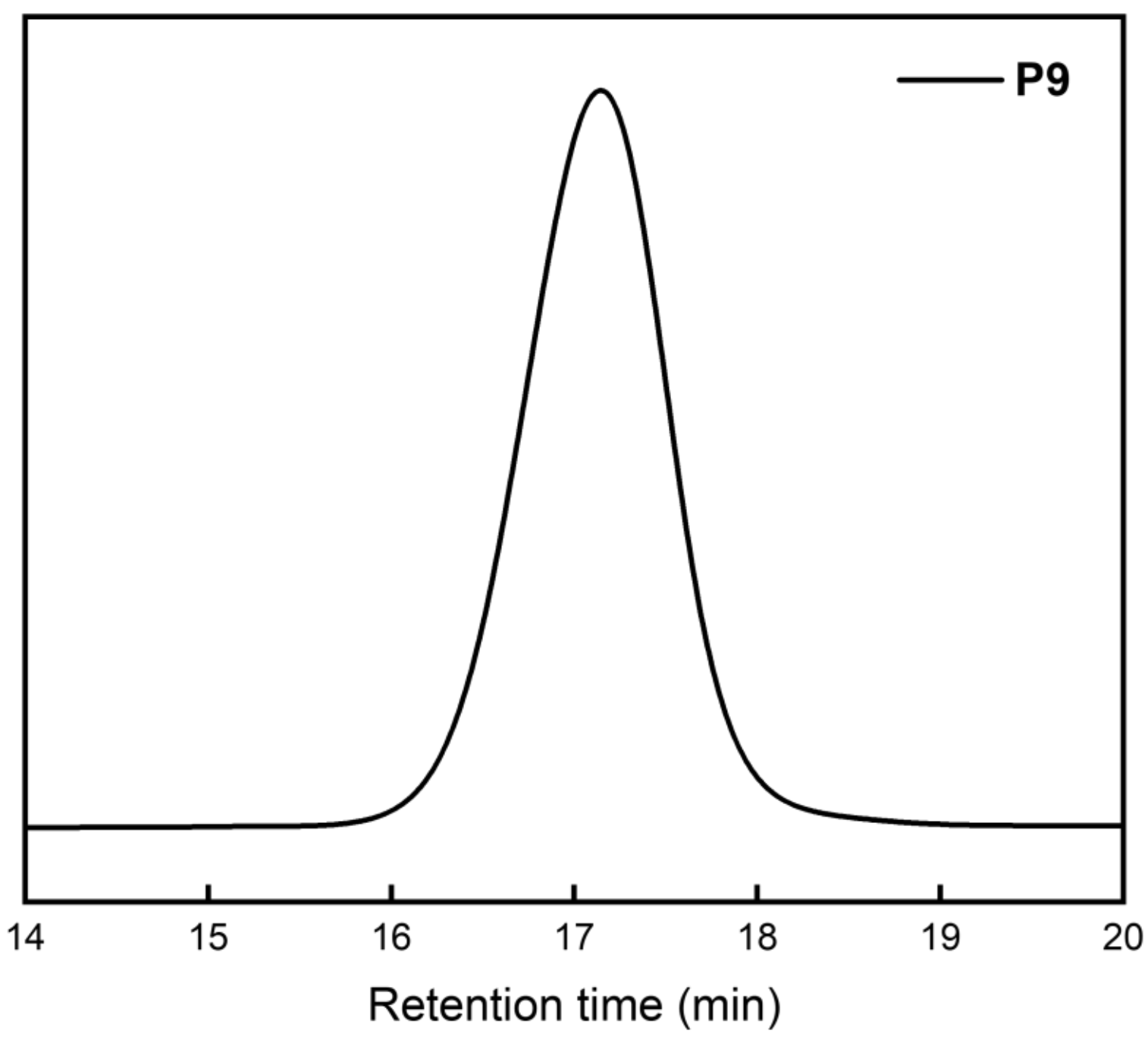

Figure S94. SEC trace (THF) of **P9**.

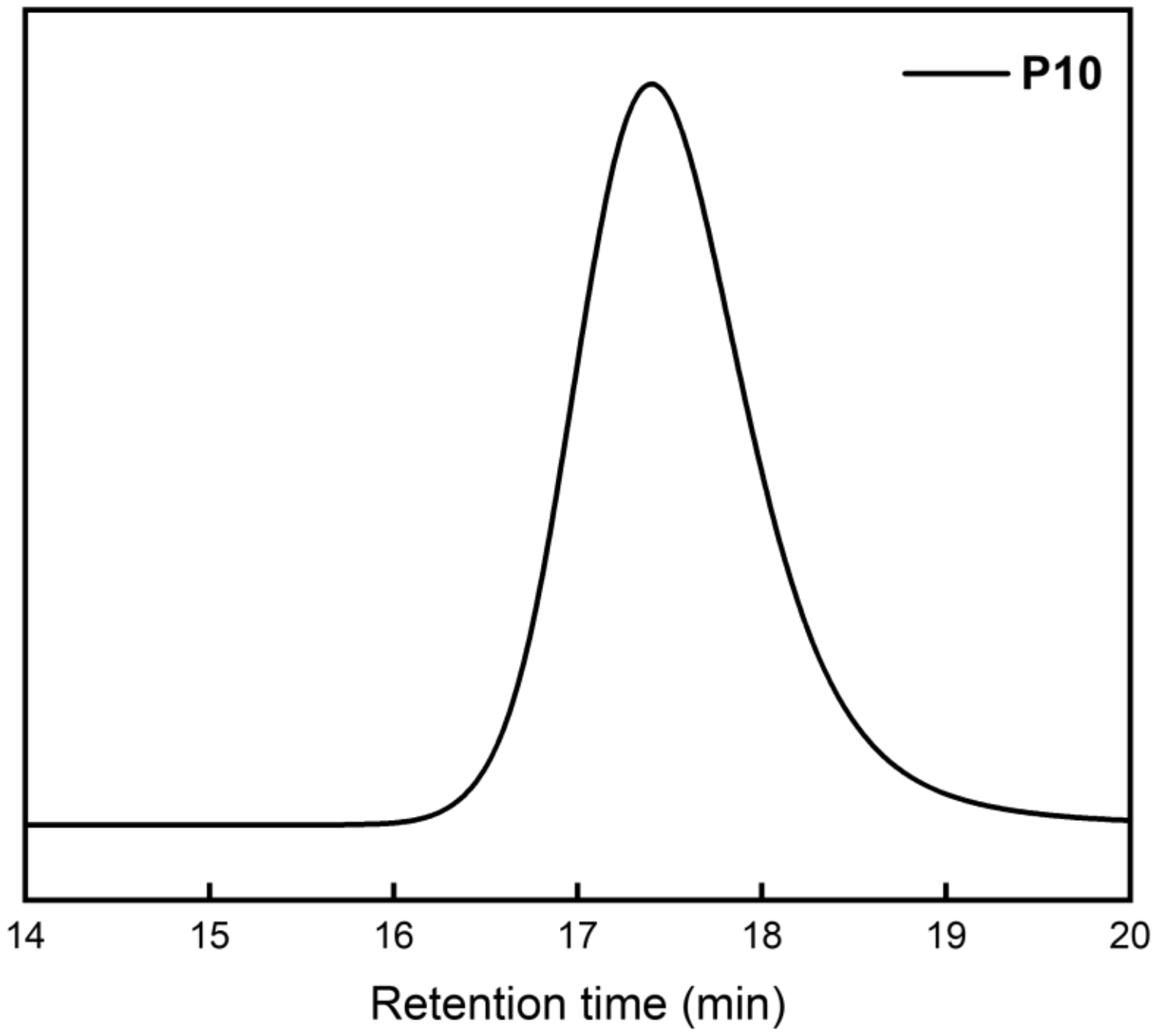

Figure S95. SEC trace (THF) of **P10**.

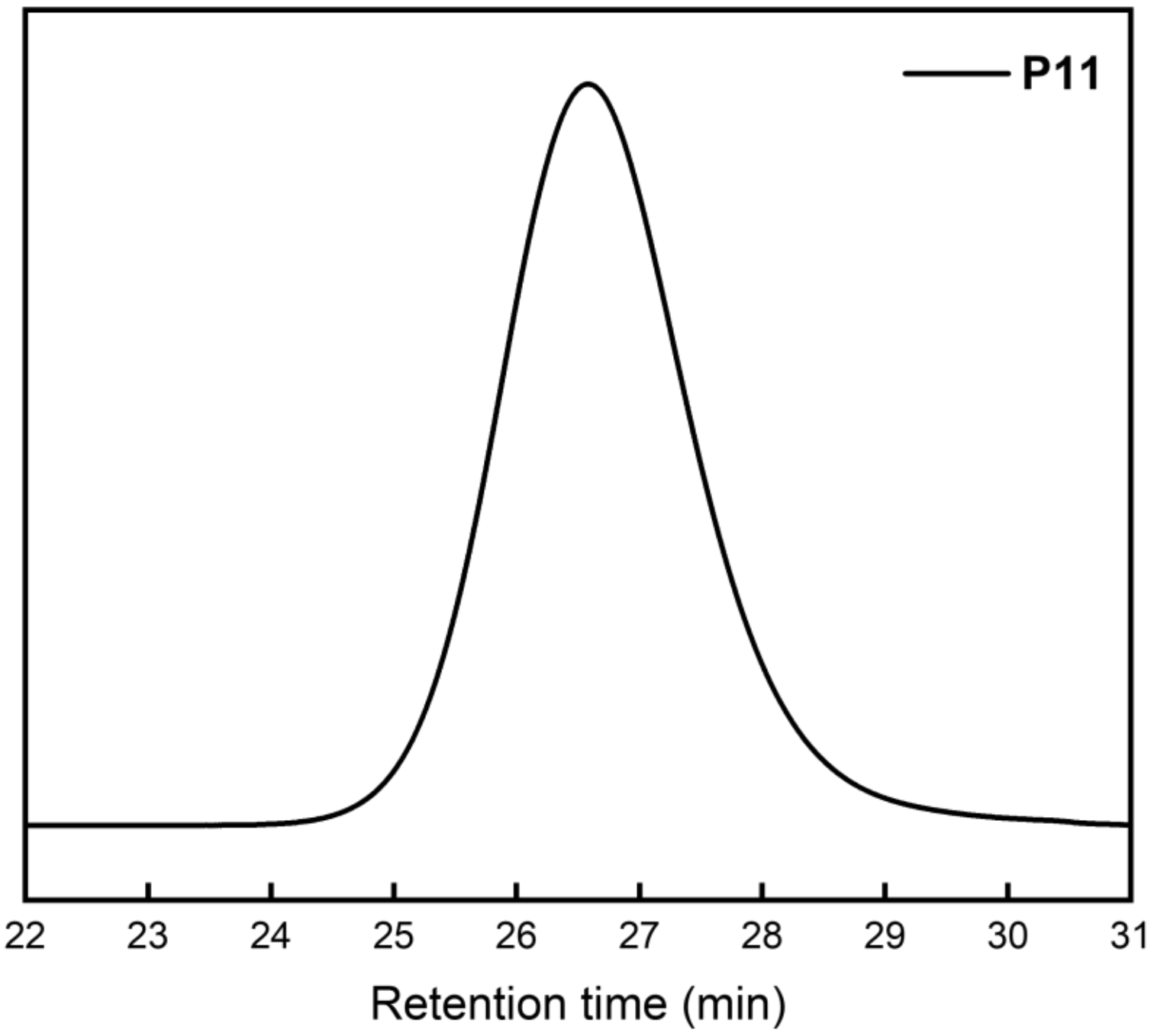

Figure S96. SEC trace (DMA) of **P11**.

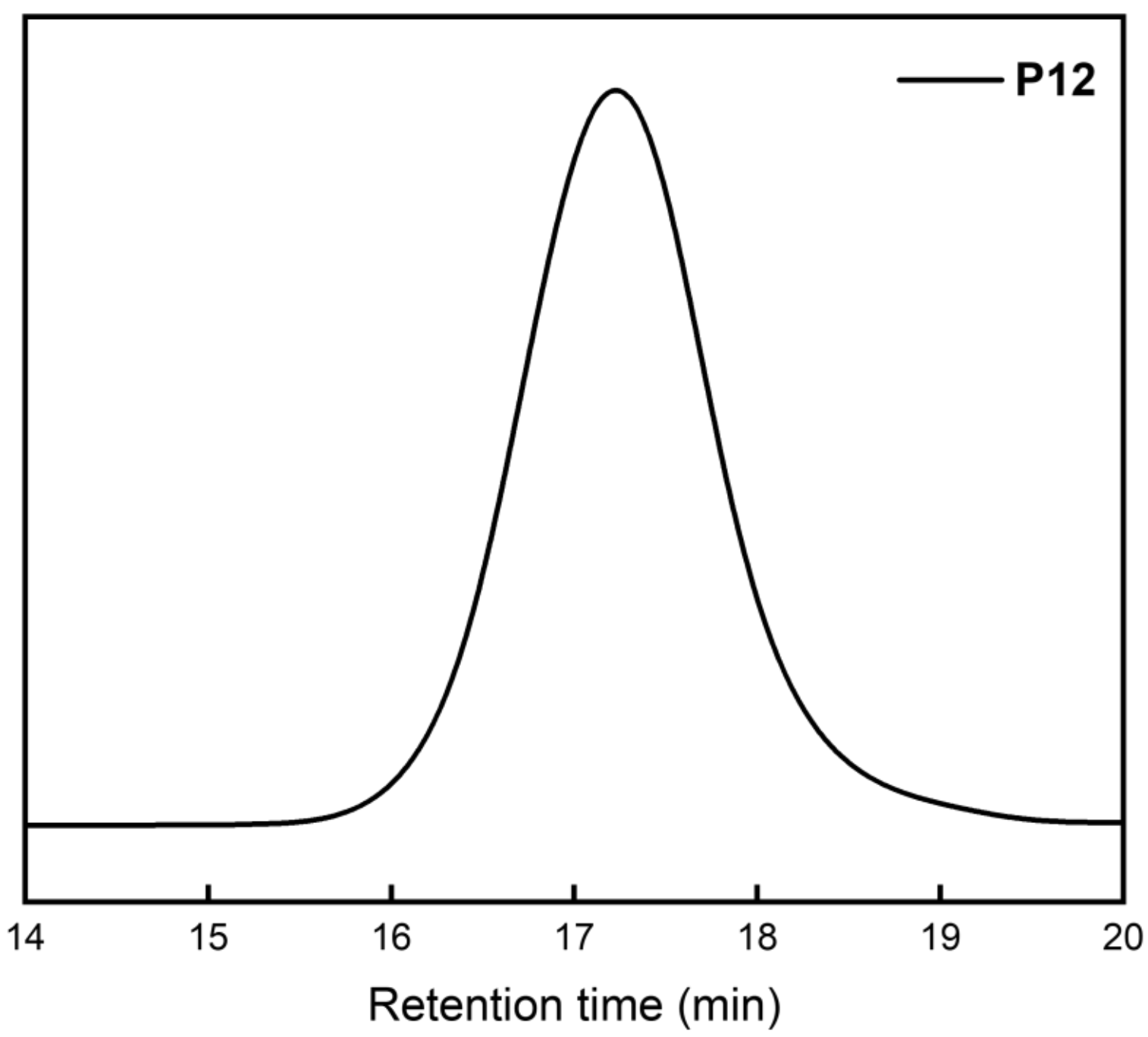

Figure S97. SEC trace (THF) of **P12**.

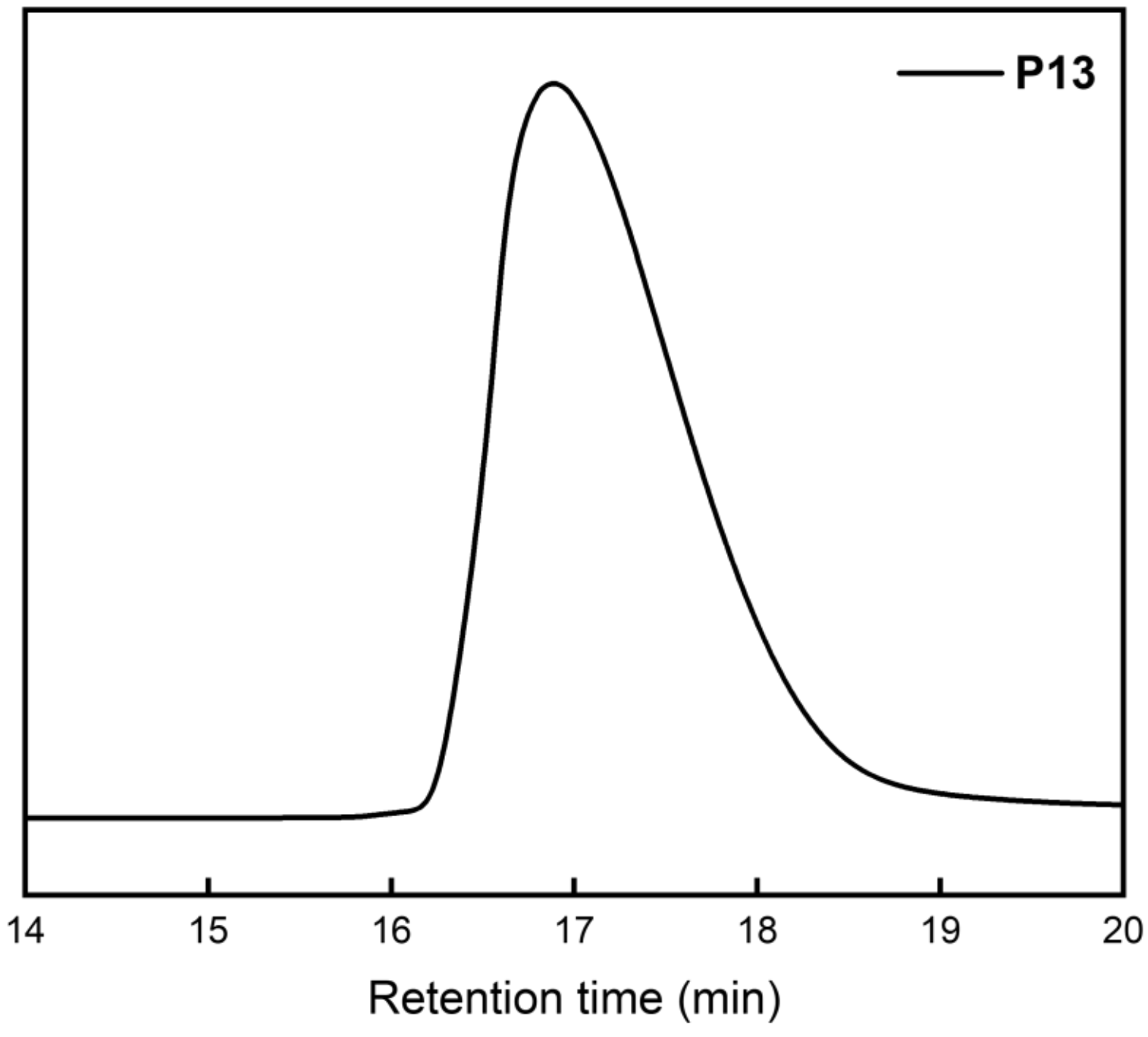

Figure S98. SEC trace (THF) of **P13**.

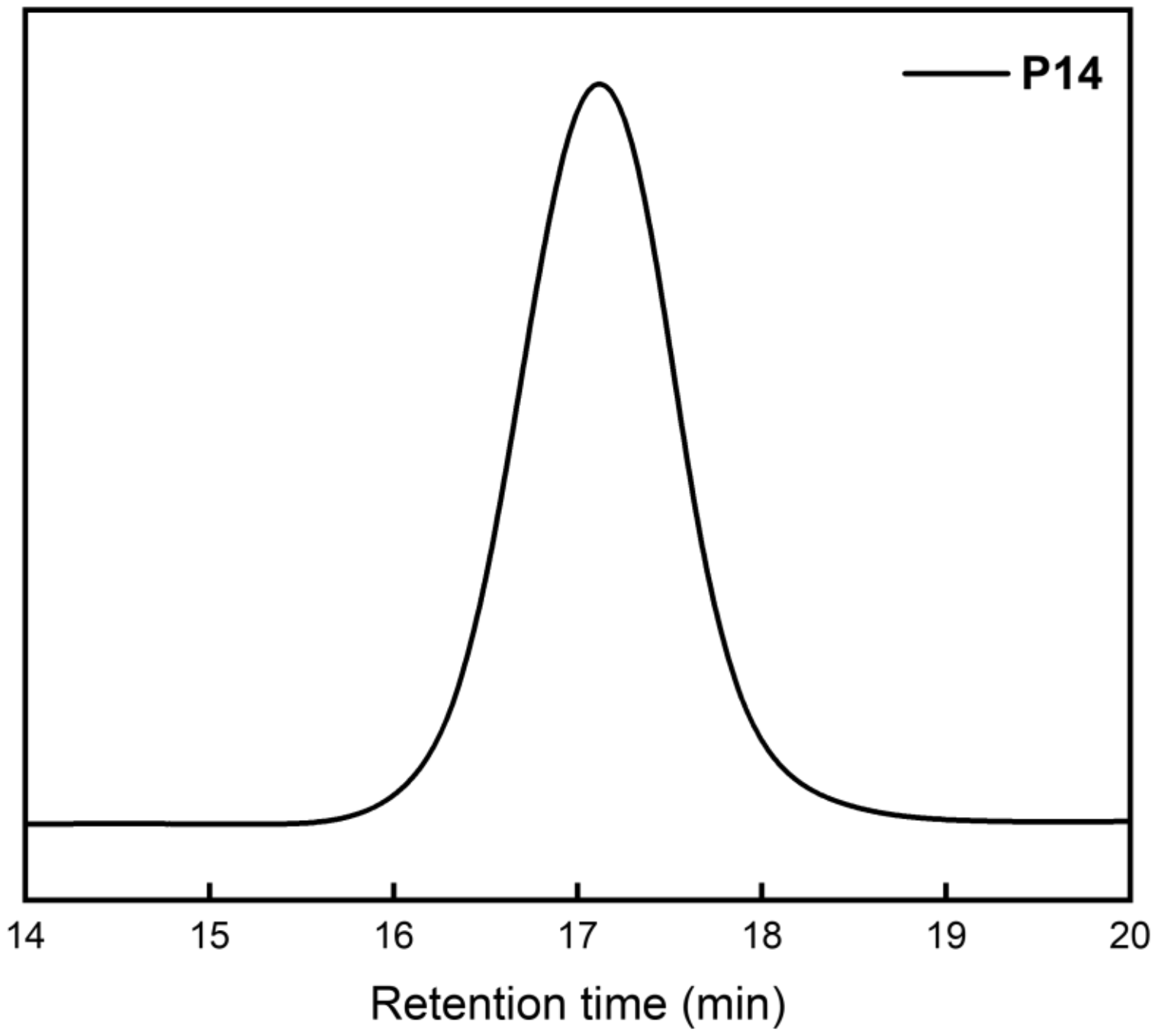

Figure S99. SEC trace (THF) of **P14**.

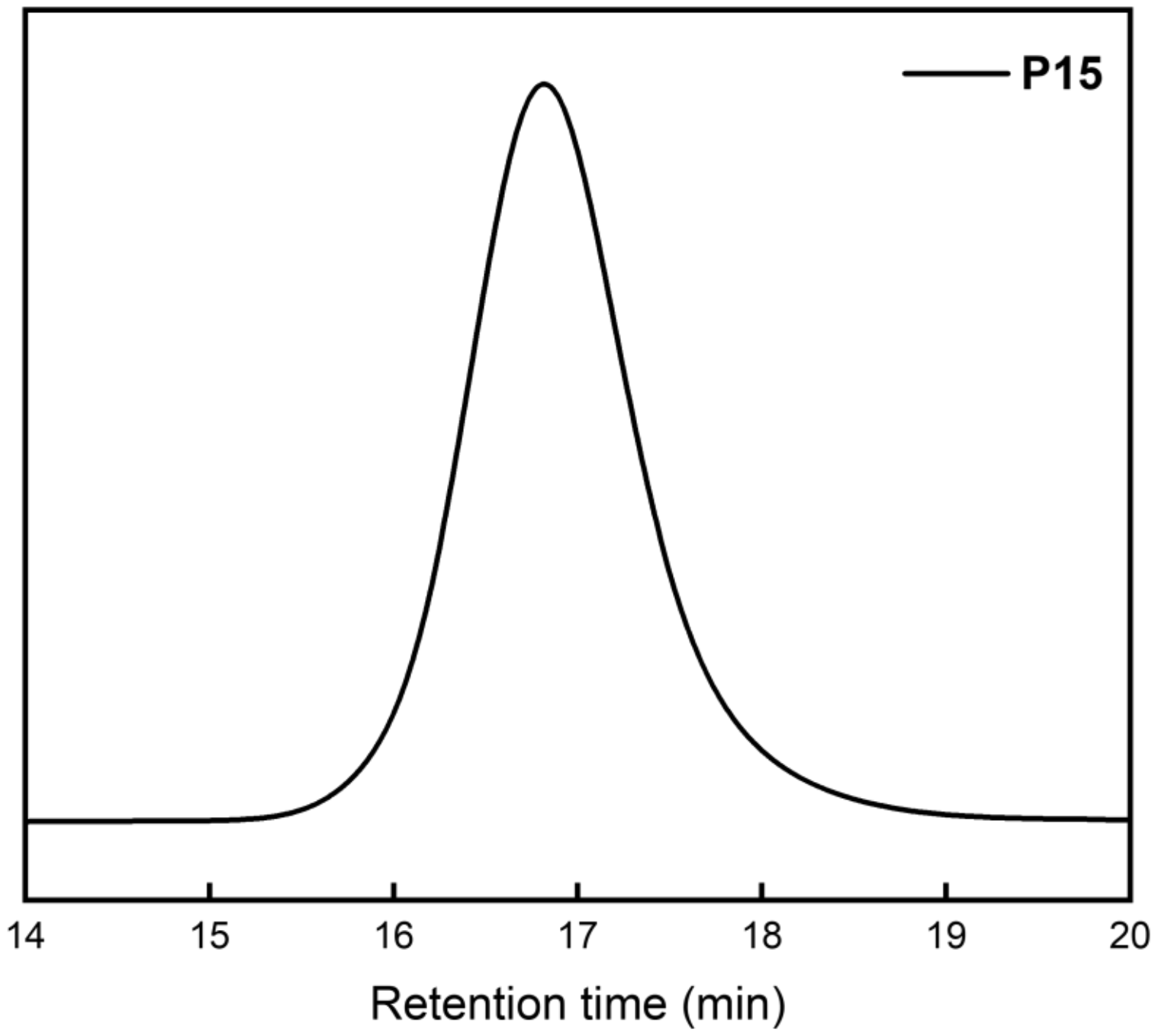

Figure S100. SEC trace (THF) of **P15**.

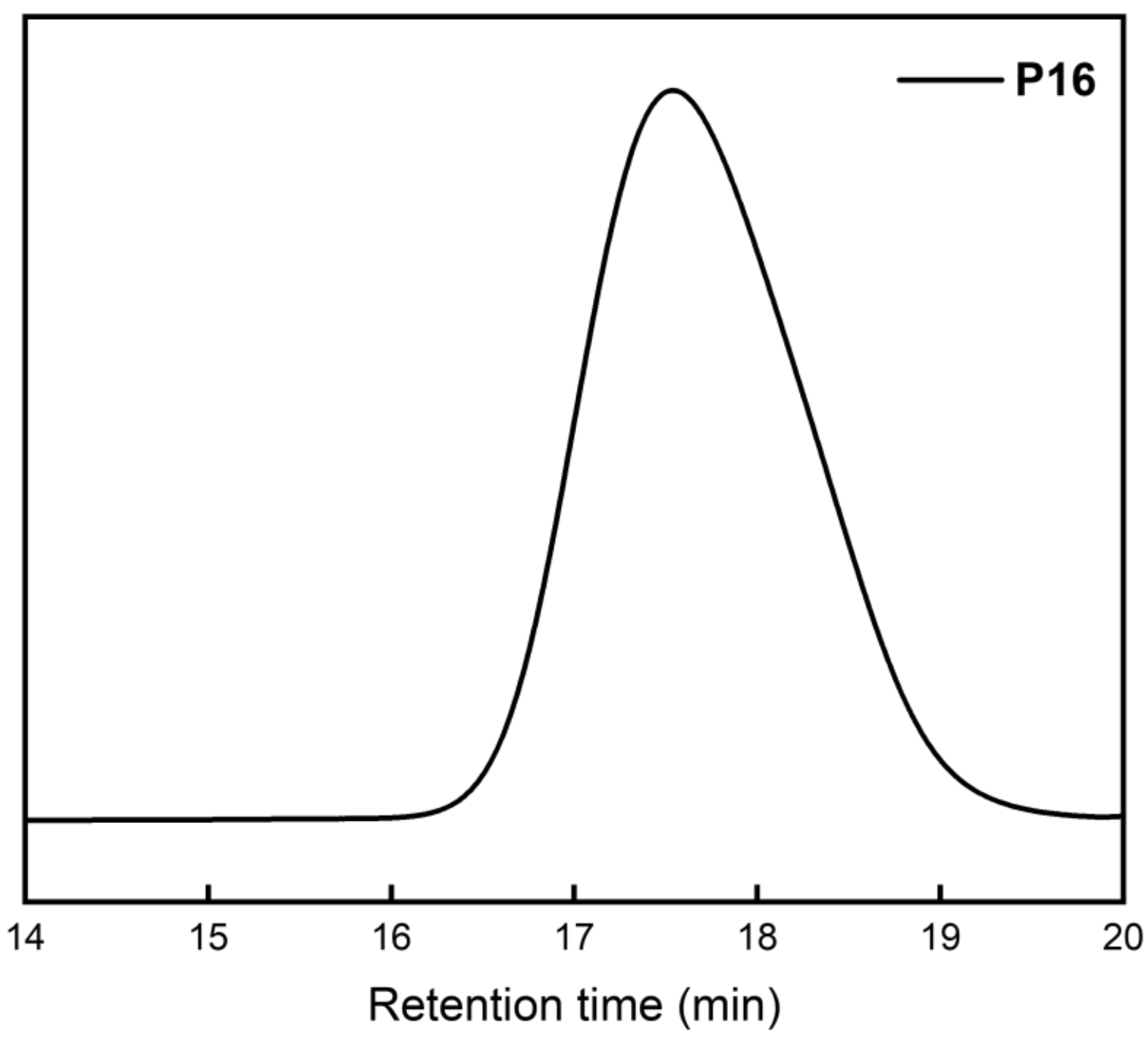

Figure S101. SEC trace (THF) of **P16**.

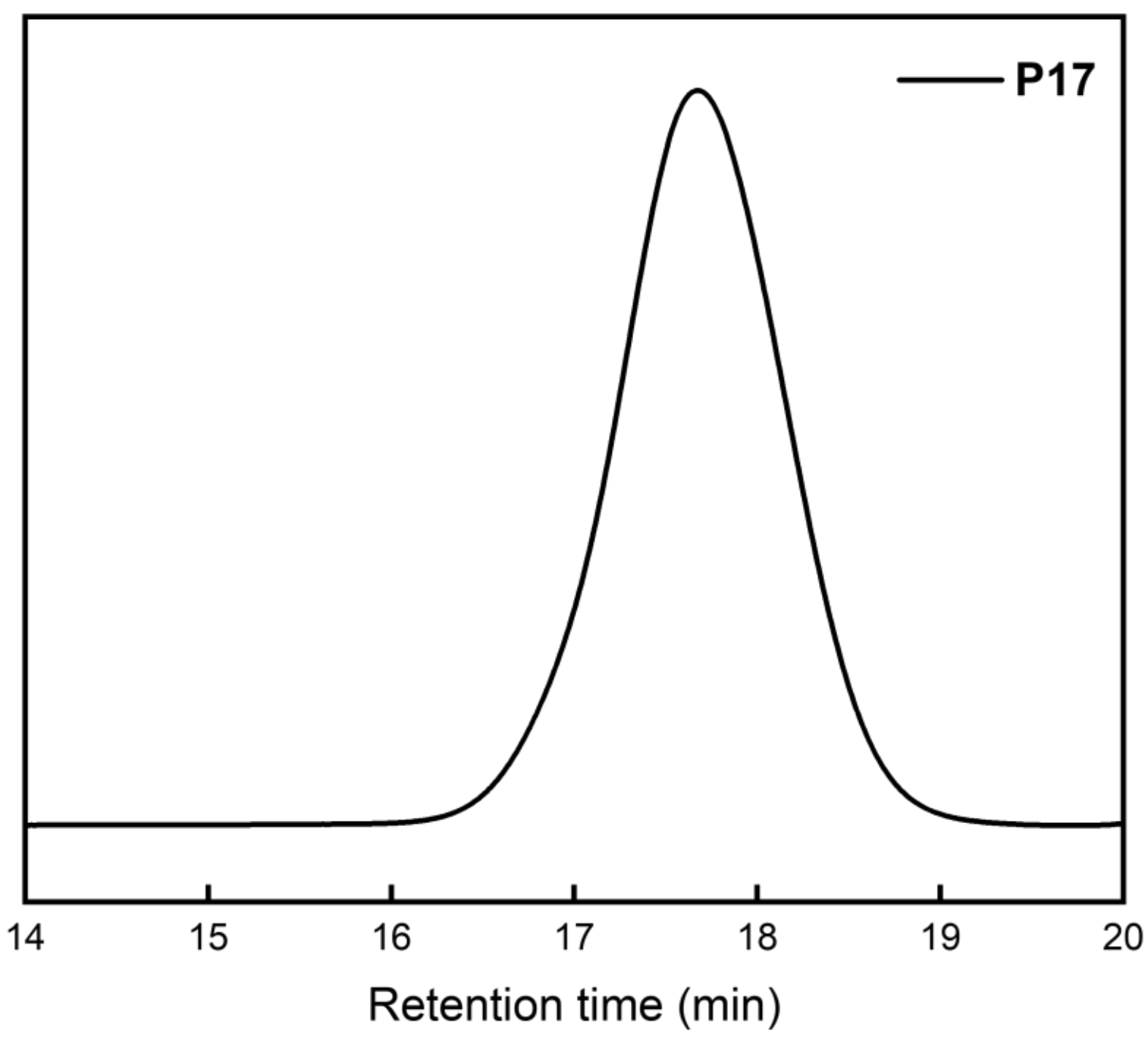

Figure S102. SEC trace (THF) of **P17**.

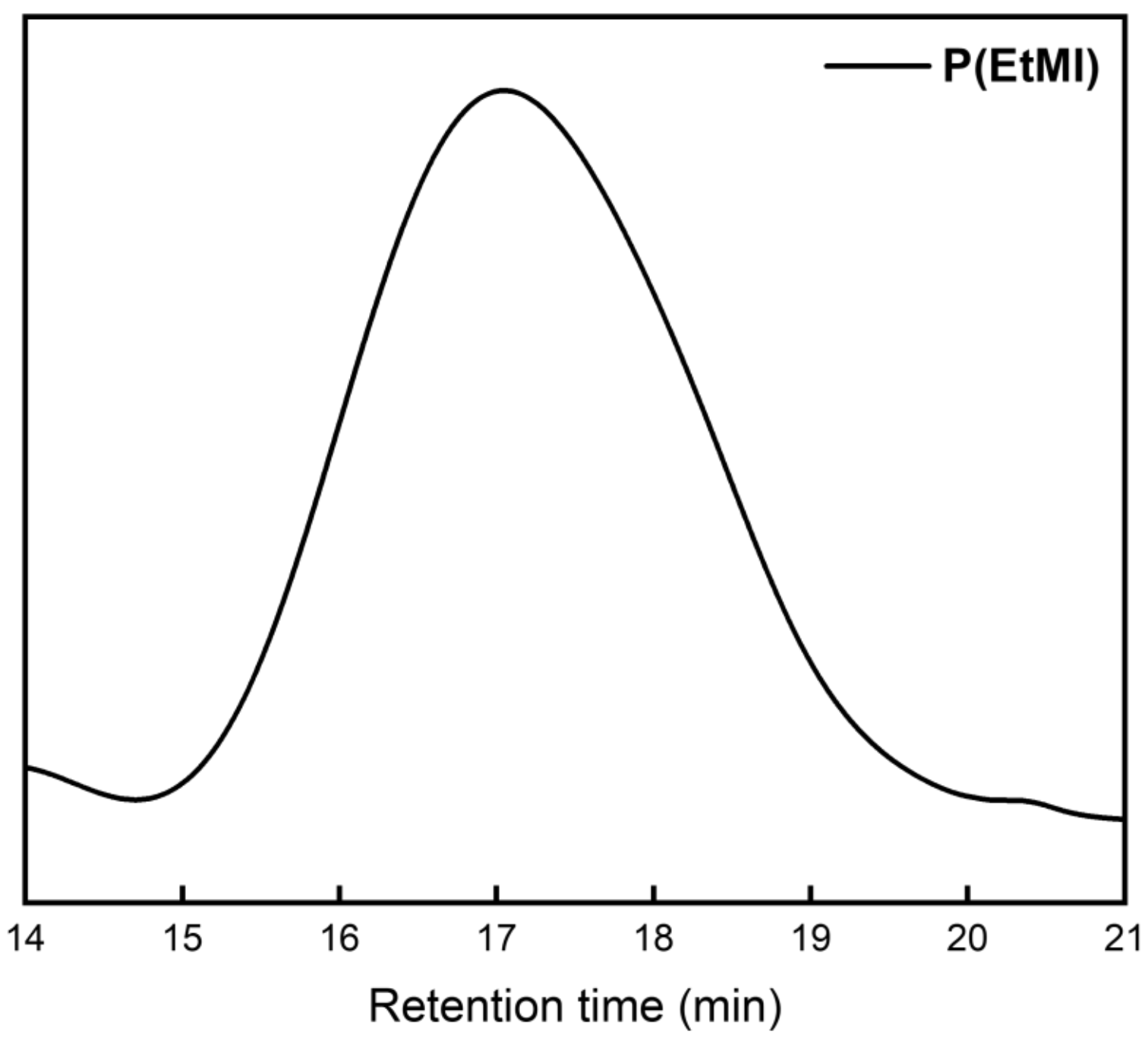

Figure S103. SEC trace (THF) of **P(EtMI)**.

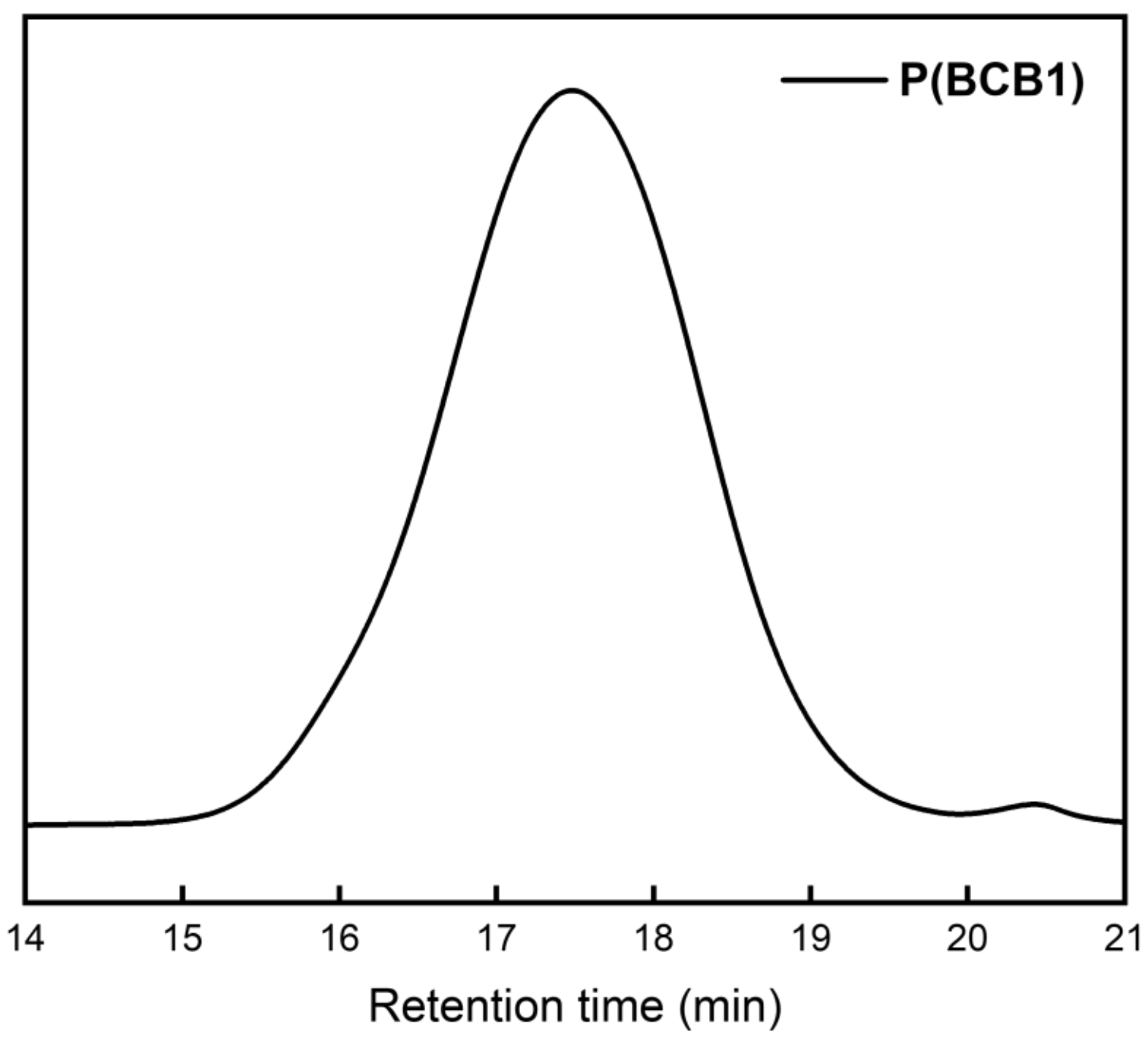

Figure S104. SEC trace (THF) of **P(BCB1)**.

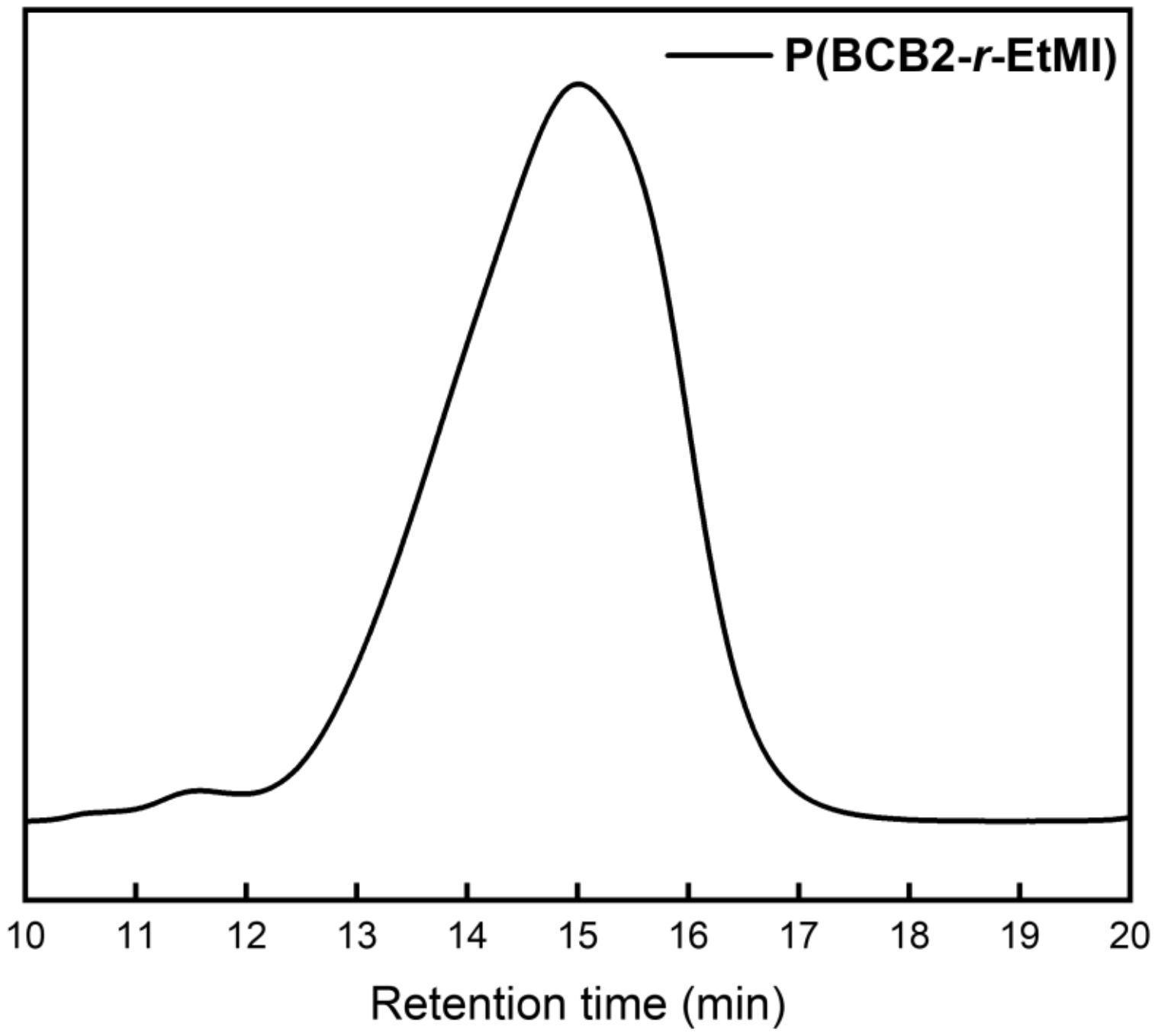

Figure S105. SEC trace (THF) of **P(BCB2-*r*-EtMI)**.

20. TGA curves

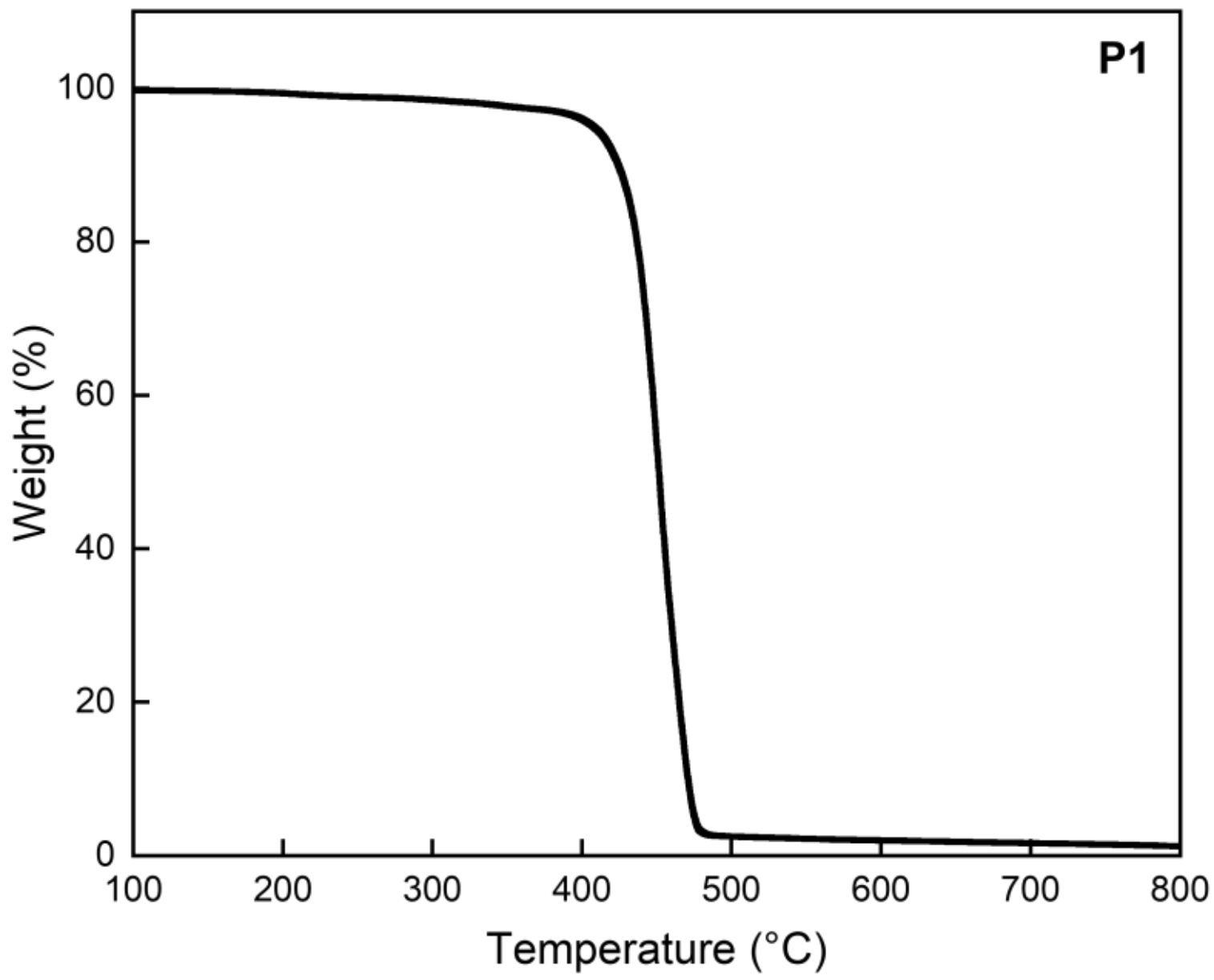

Figure S106. TGA curve of **P1**.

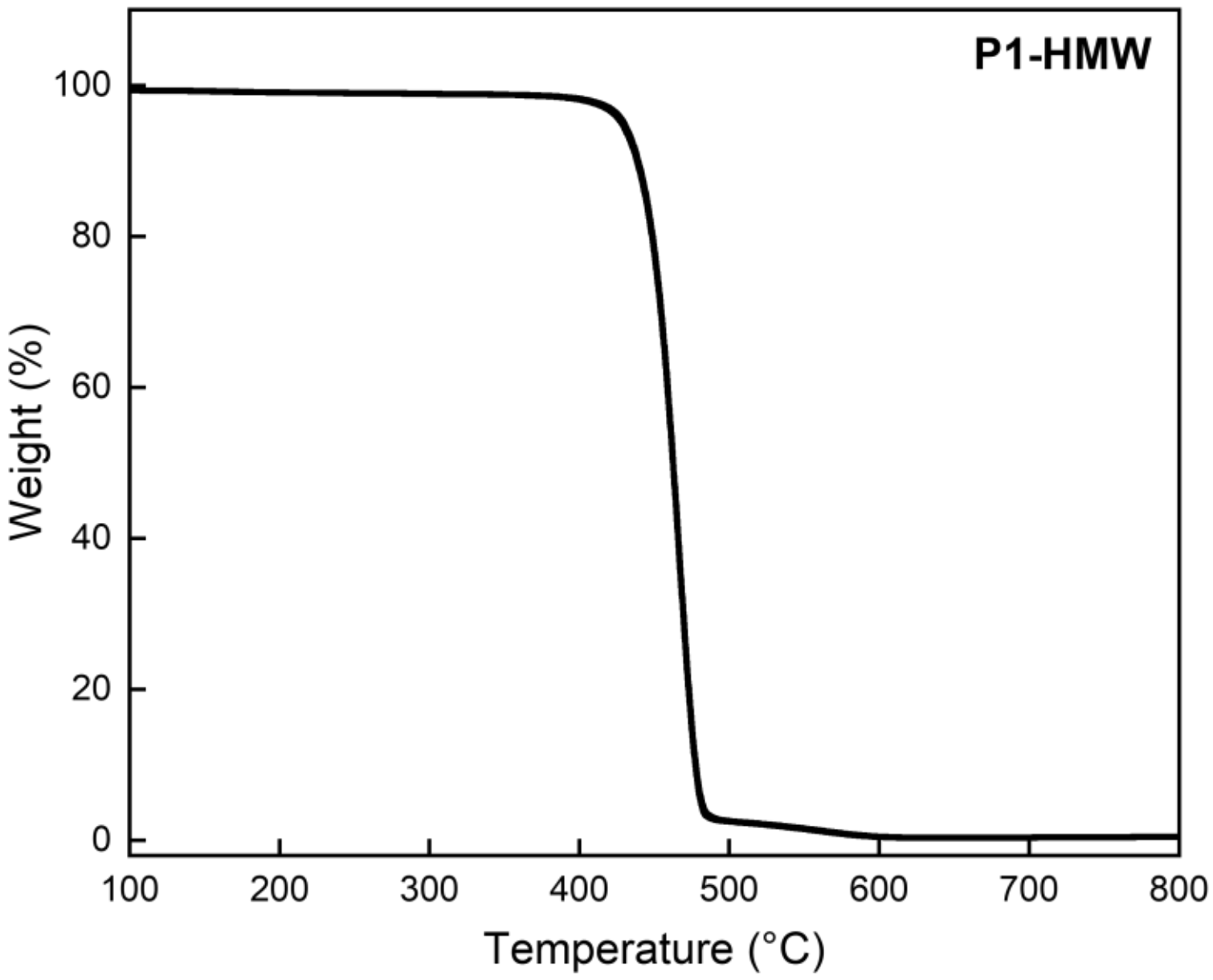

Figure S107. TGA curve of high molecular-weight copolymer **P1** (M_n= 76.6 kDa).

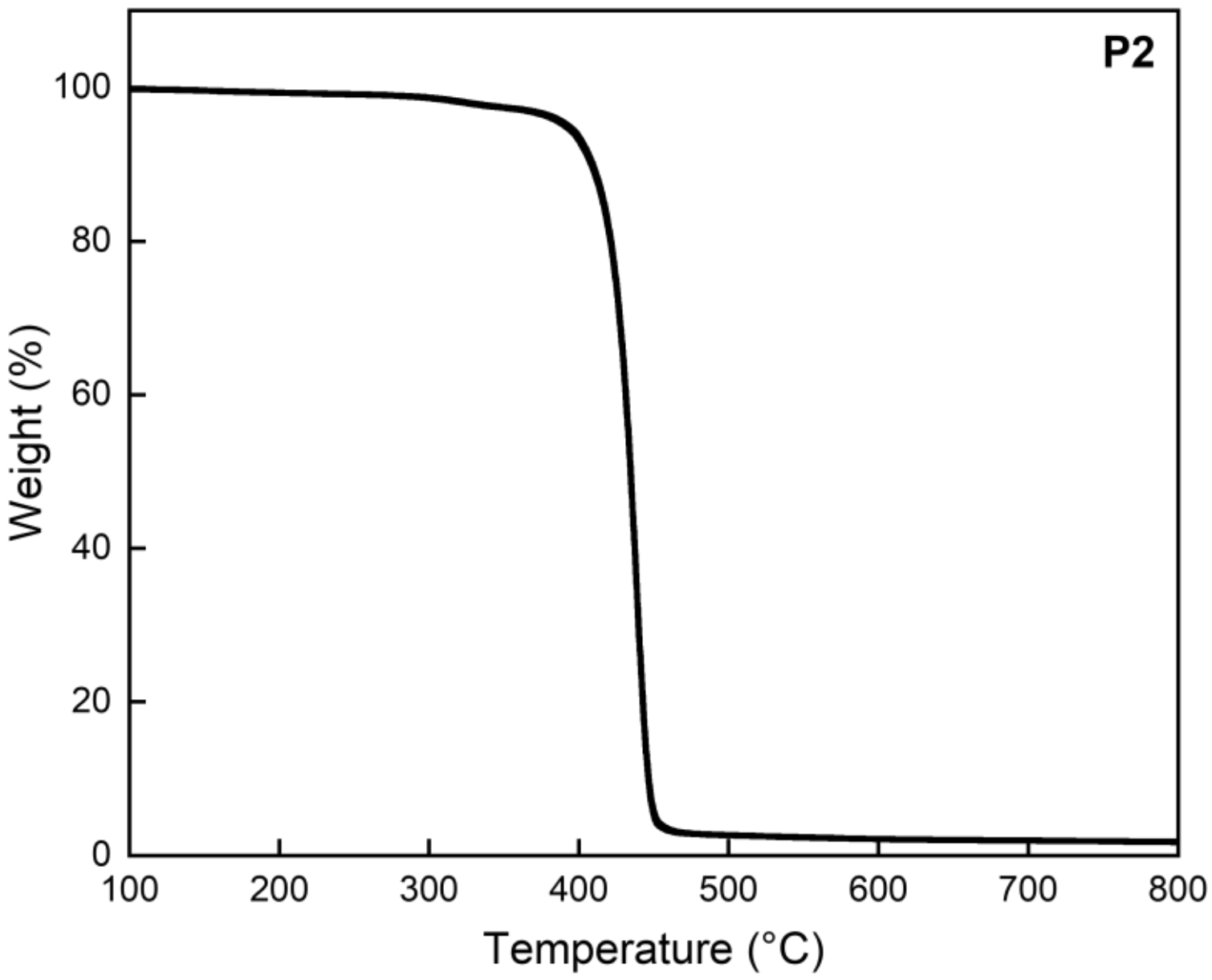

Figure S108. TGA curve of **P2**.

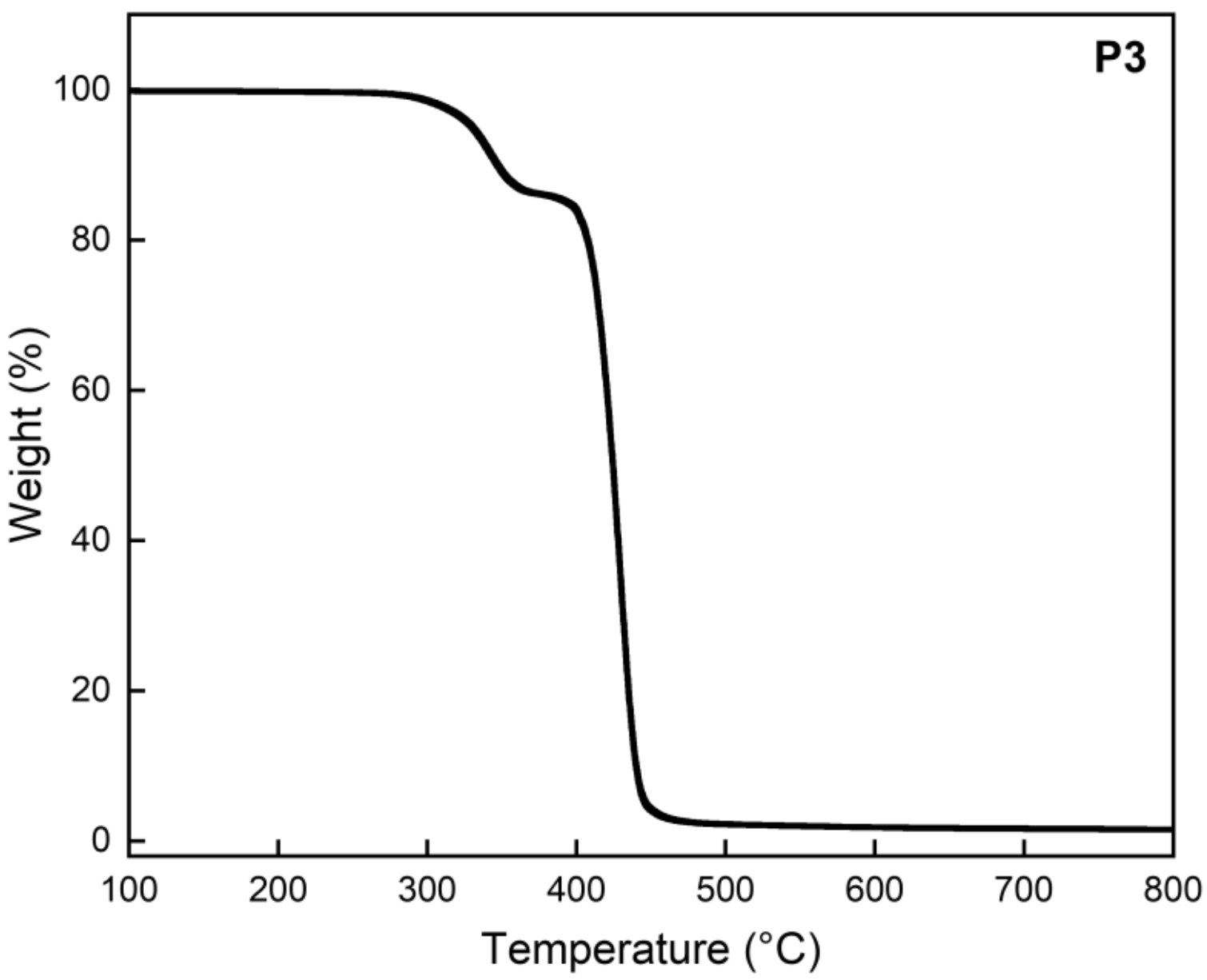

Figure S109. TGA curve of **P3**.

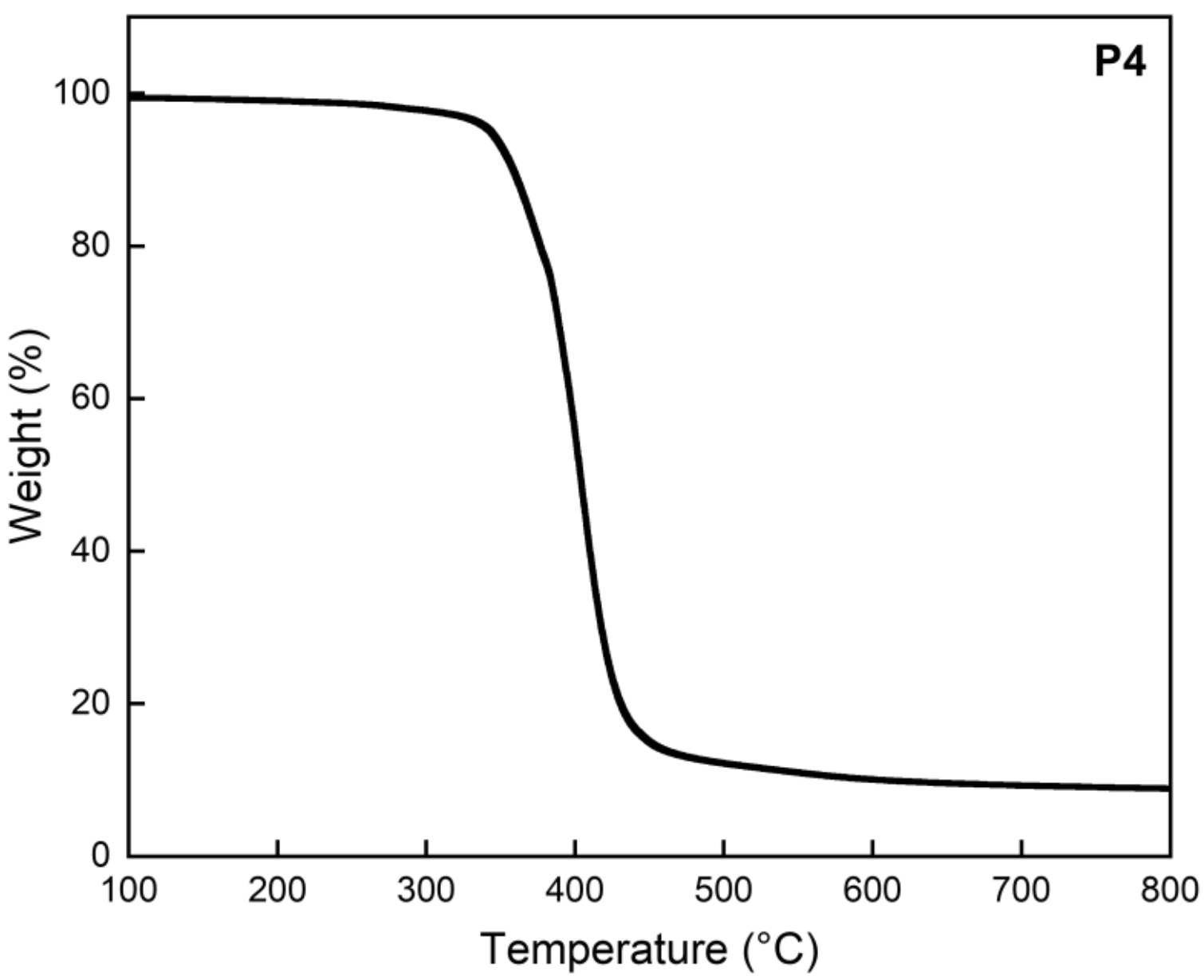

Figure S110. TGA curve of **P4**.

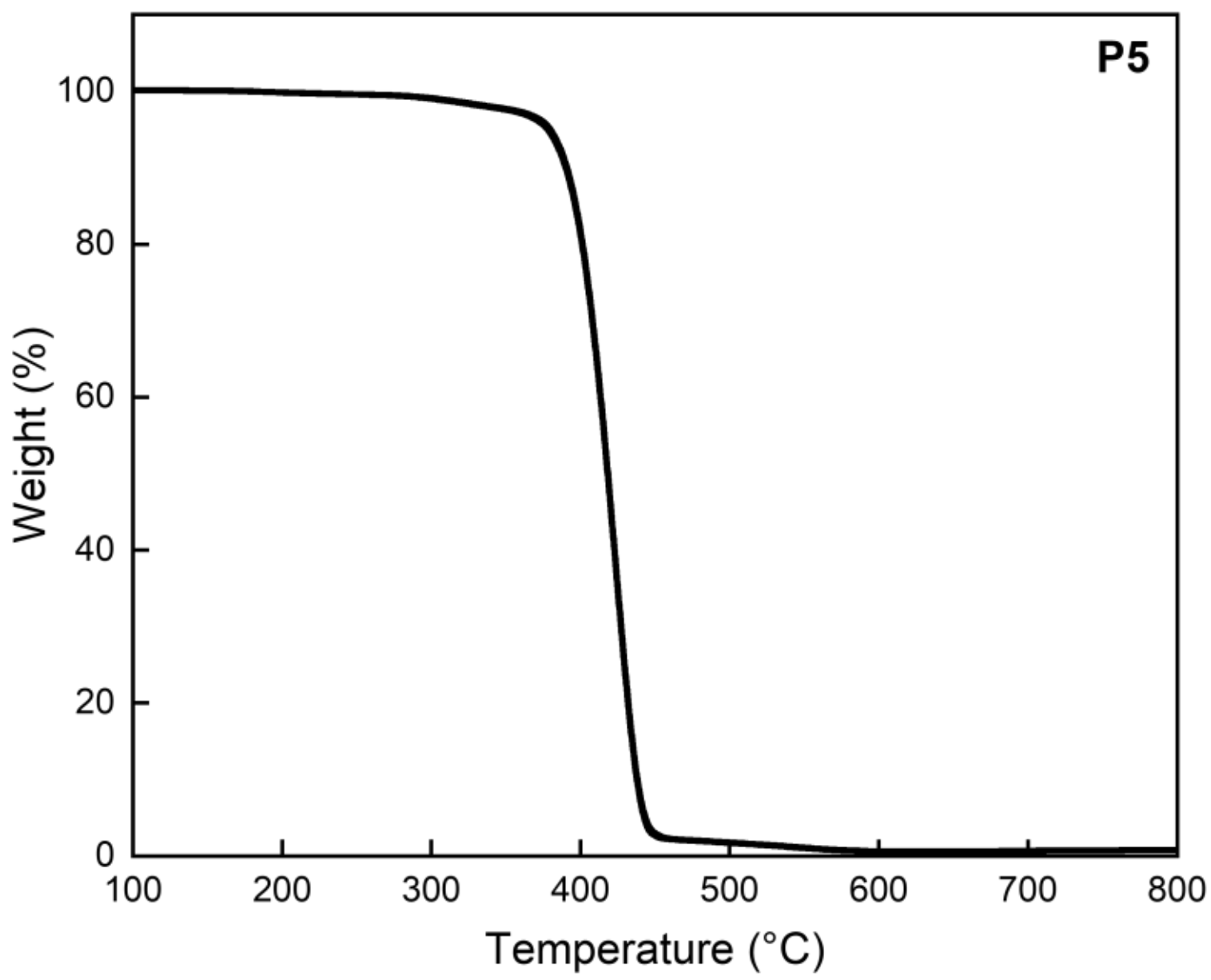

Figure S111. TGA curve of **P5**.

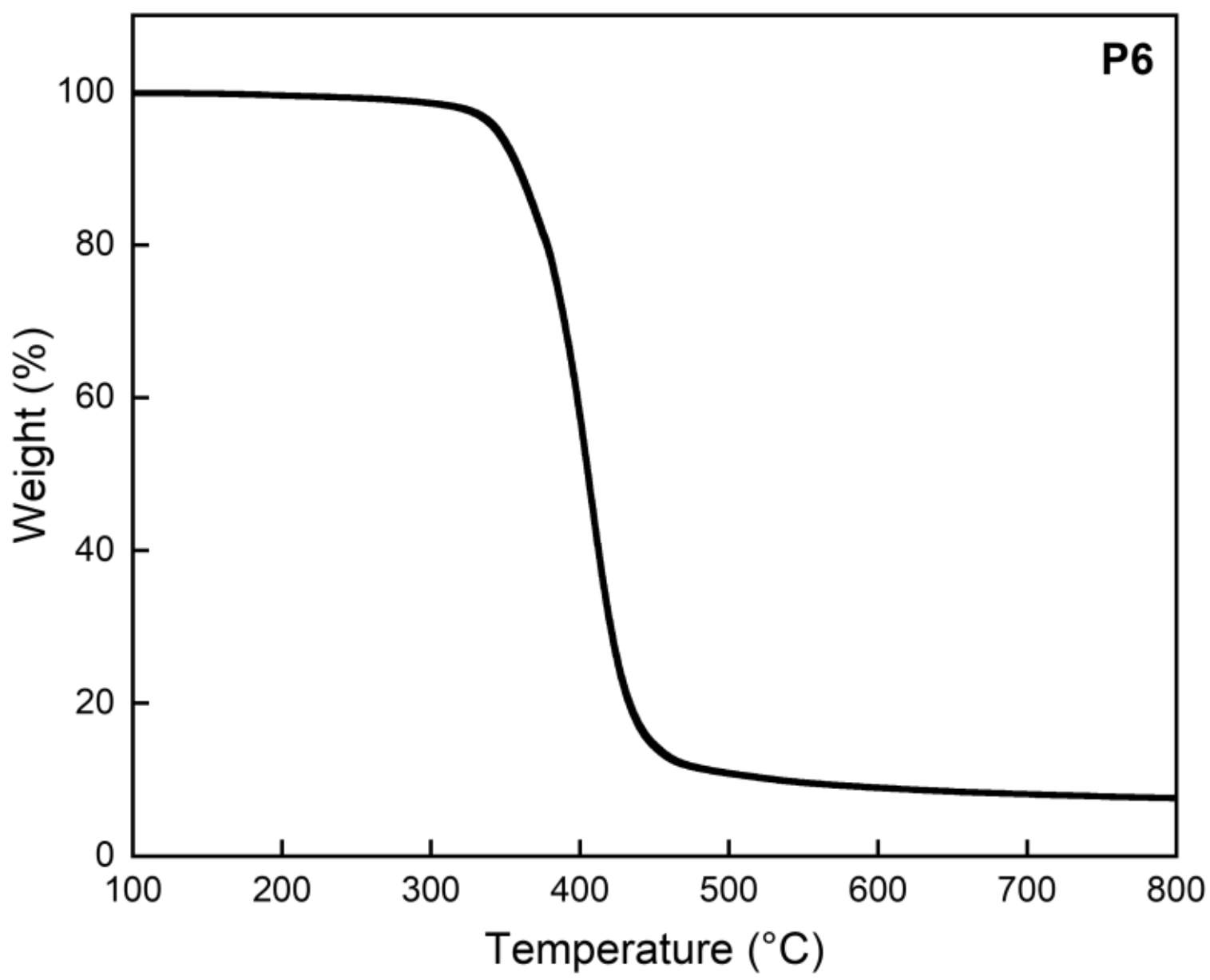

Figure S112. TGA curve of **P6**.

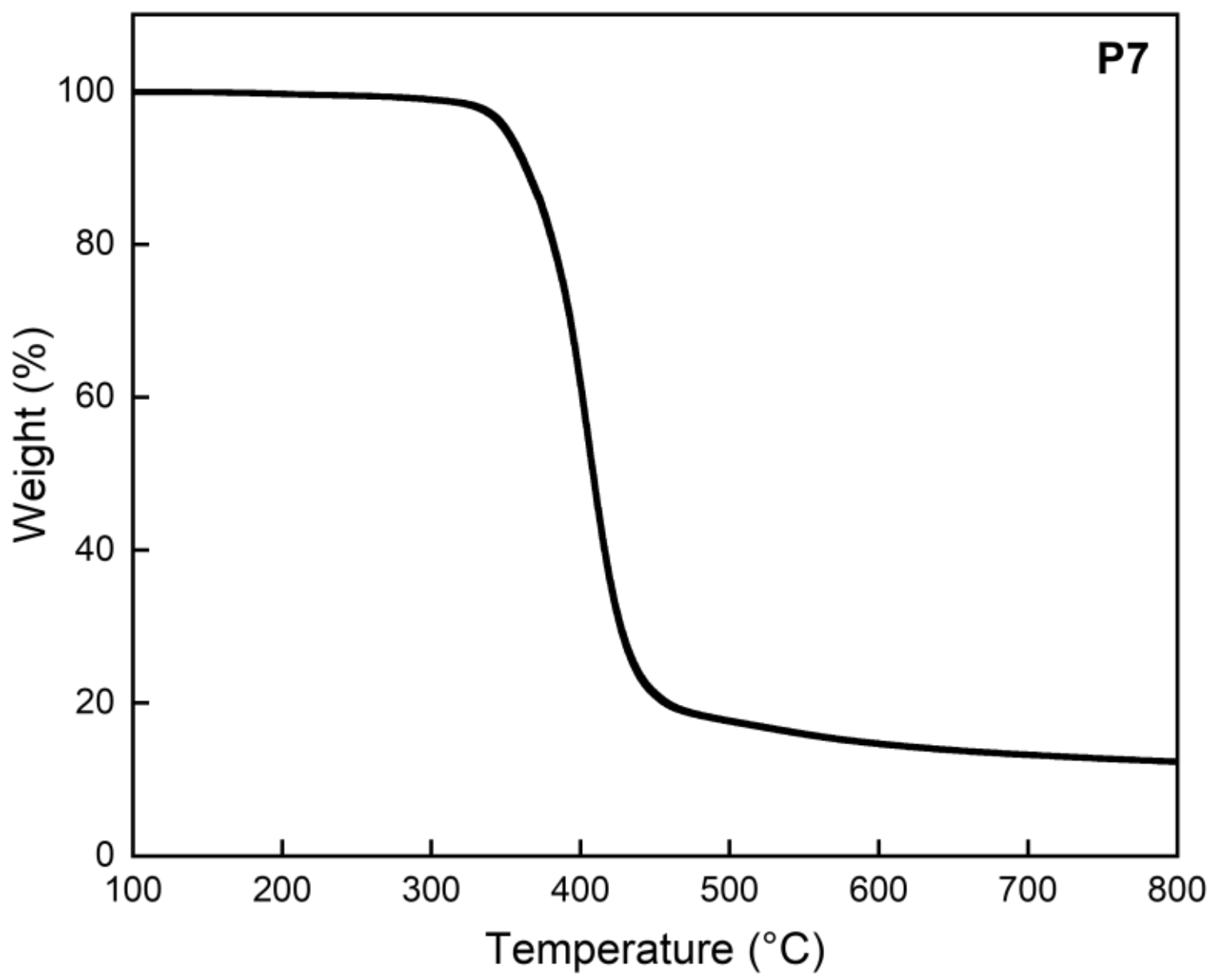

Figure S113. TGA curve of **P7**.

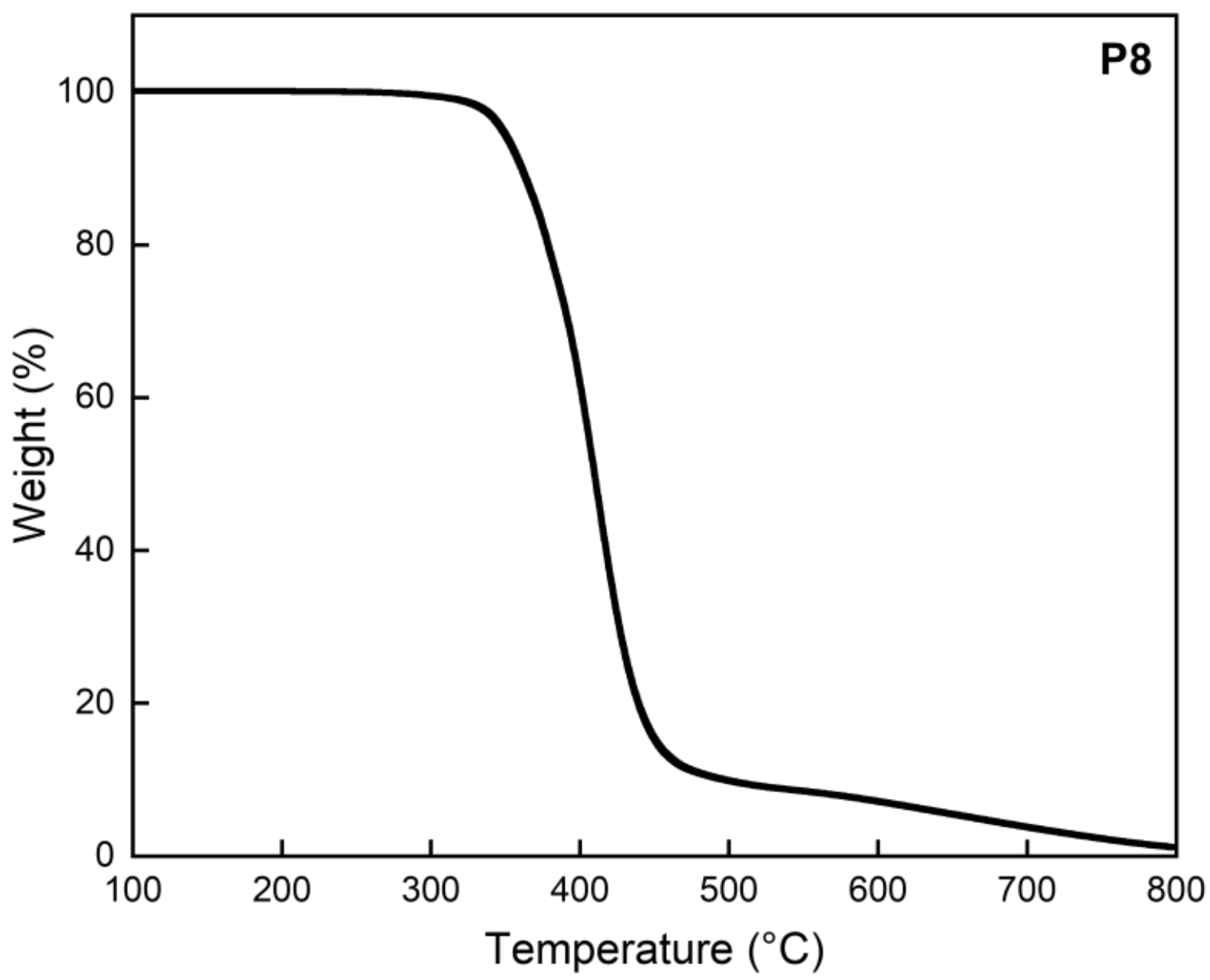

Figure S114. TGA curve of **P8**.

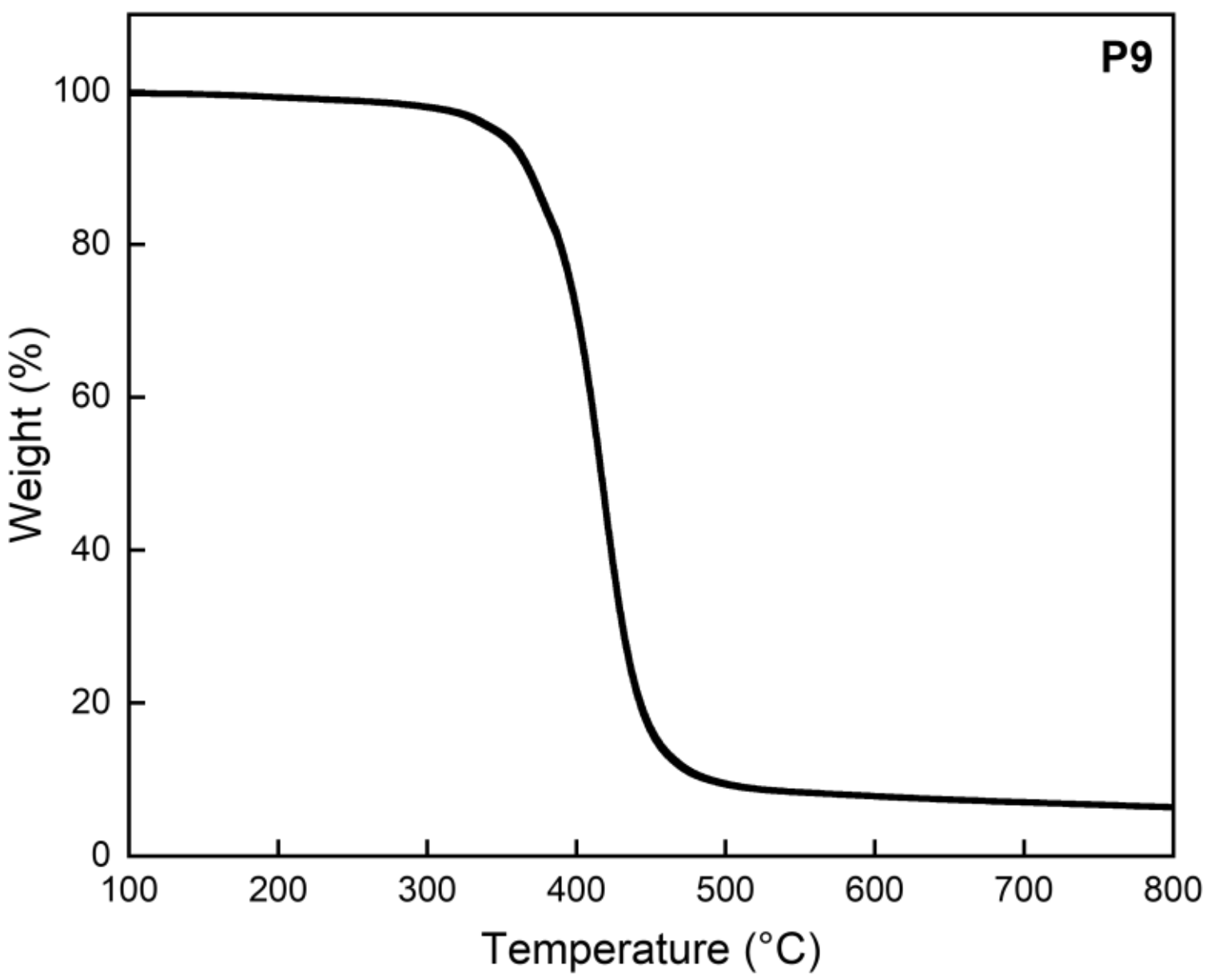

Figure S115. TGA curve of **P9**.

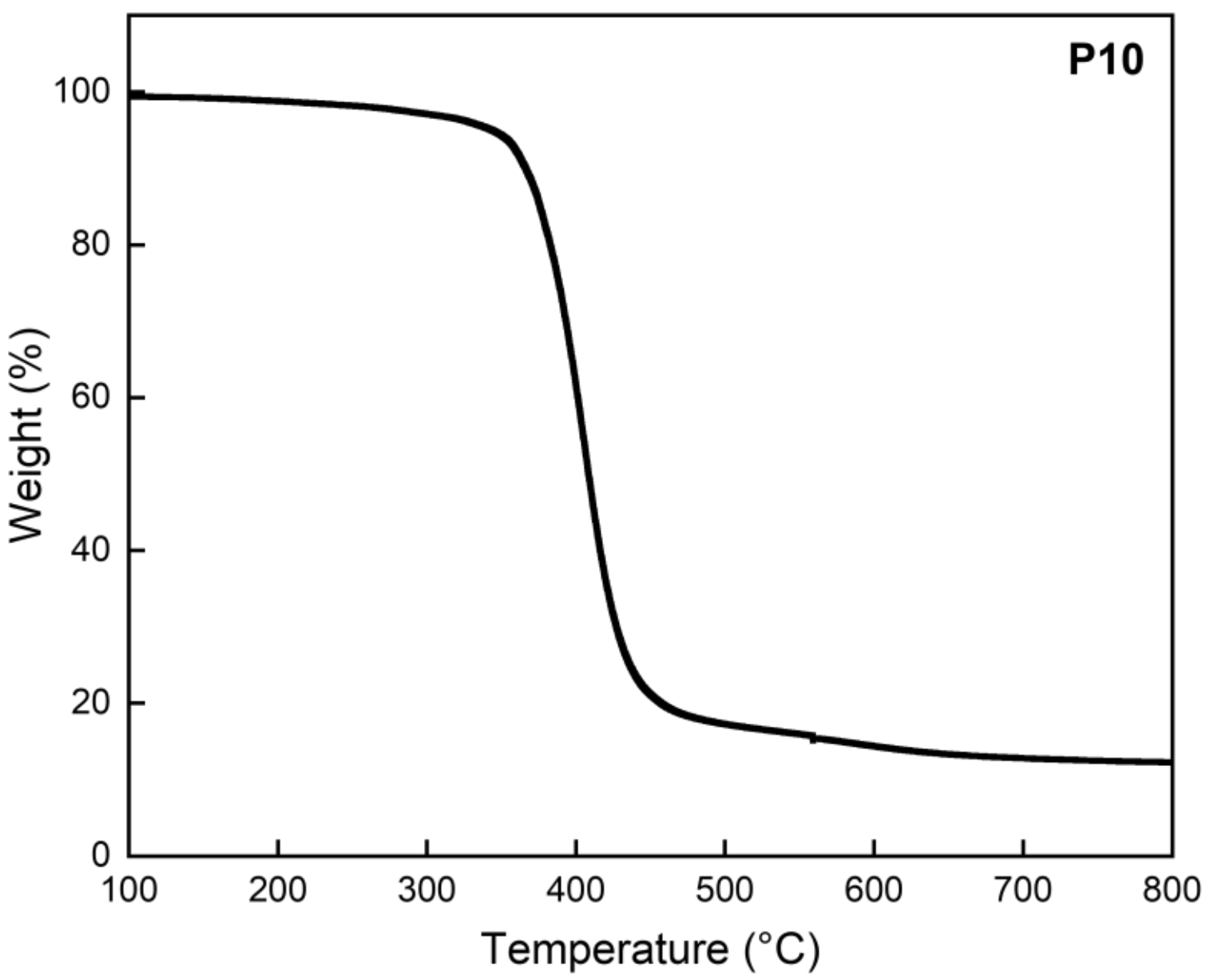

Figure S116. TGA curve of **P10**.

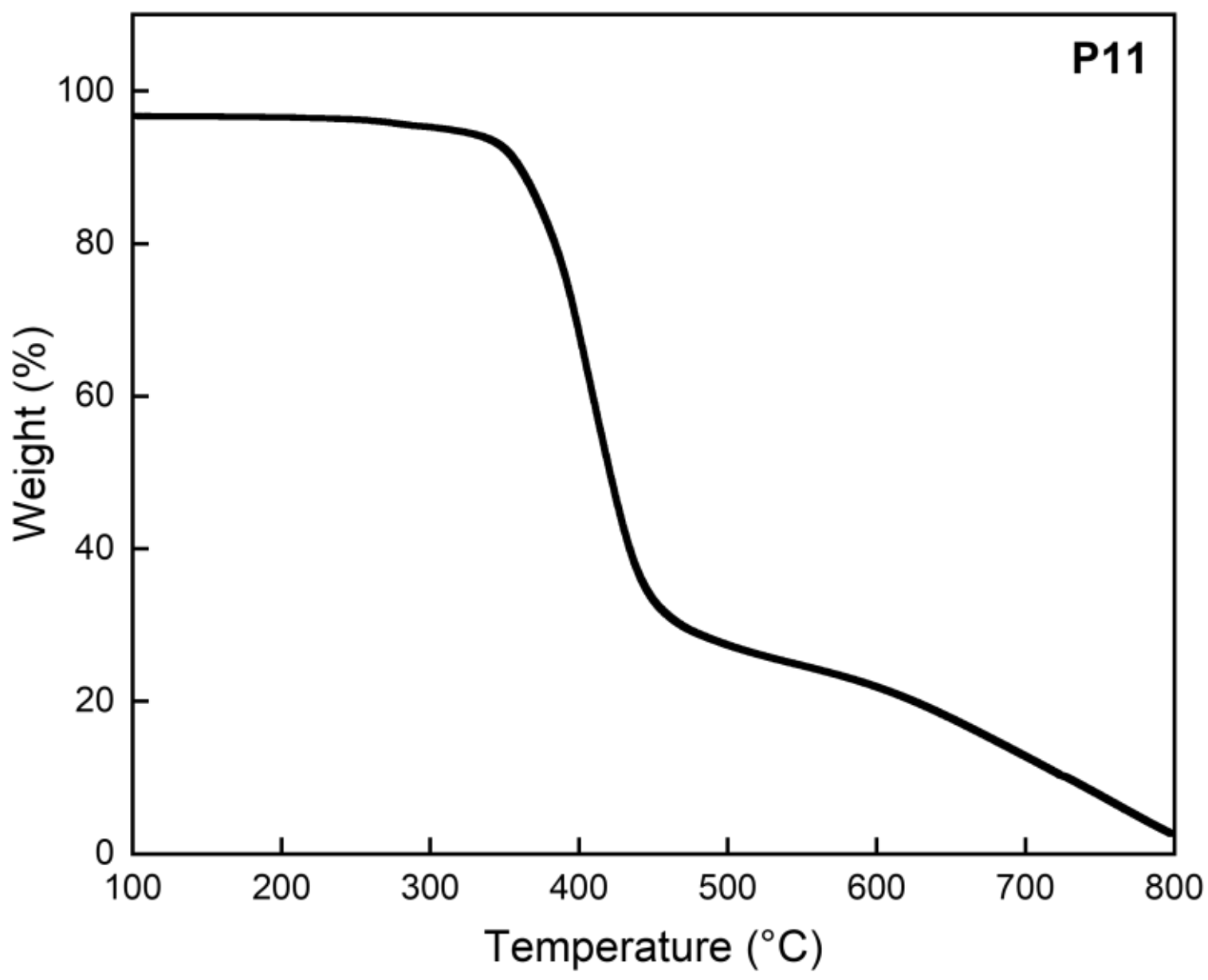

Figure S117. TGA curve of **P11**.

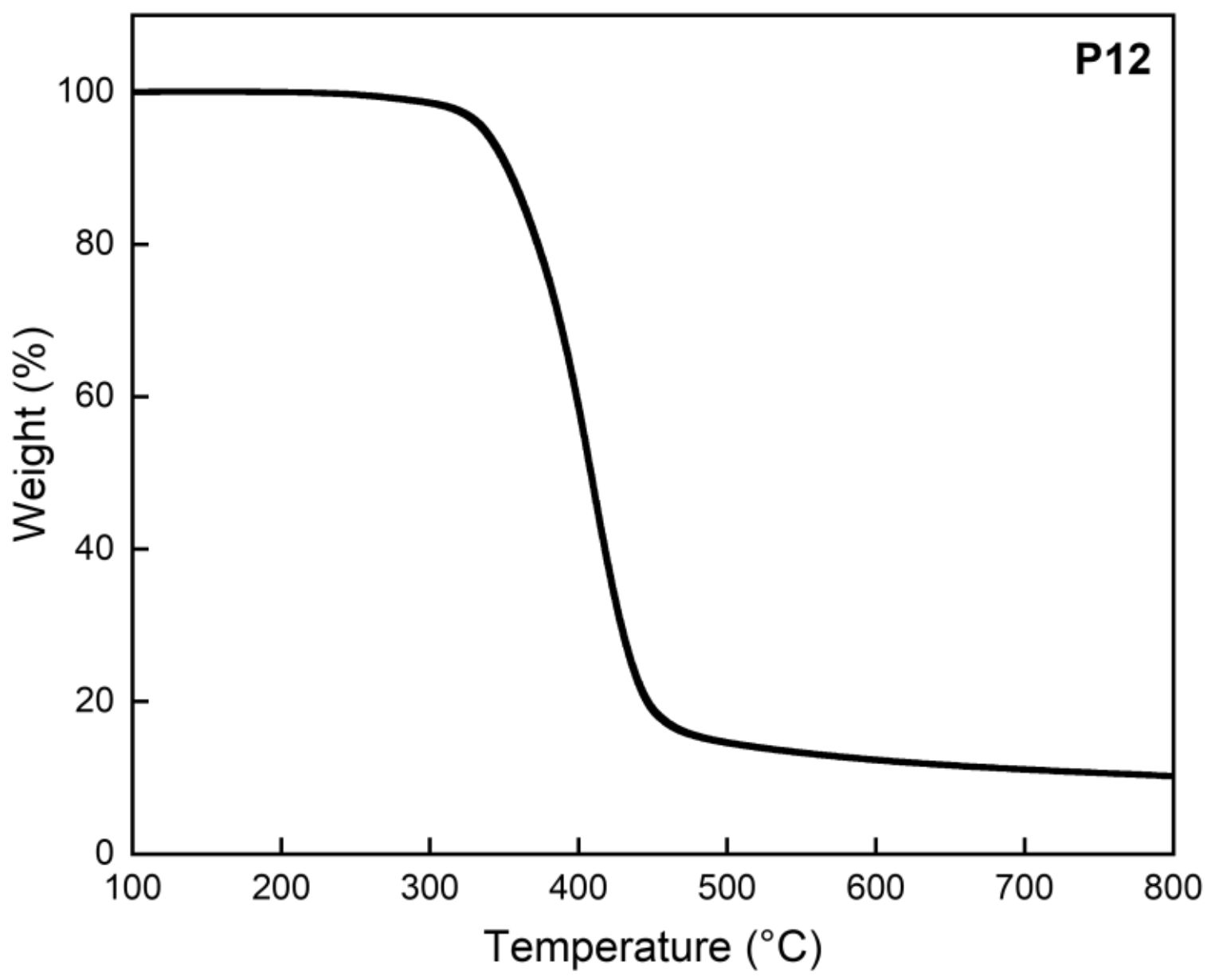

Figure S118. TGA curve of **P12**.

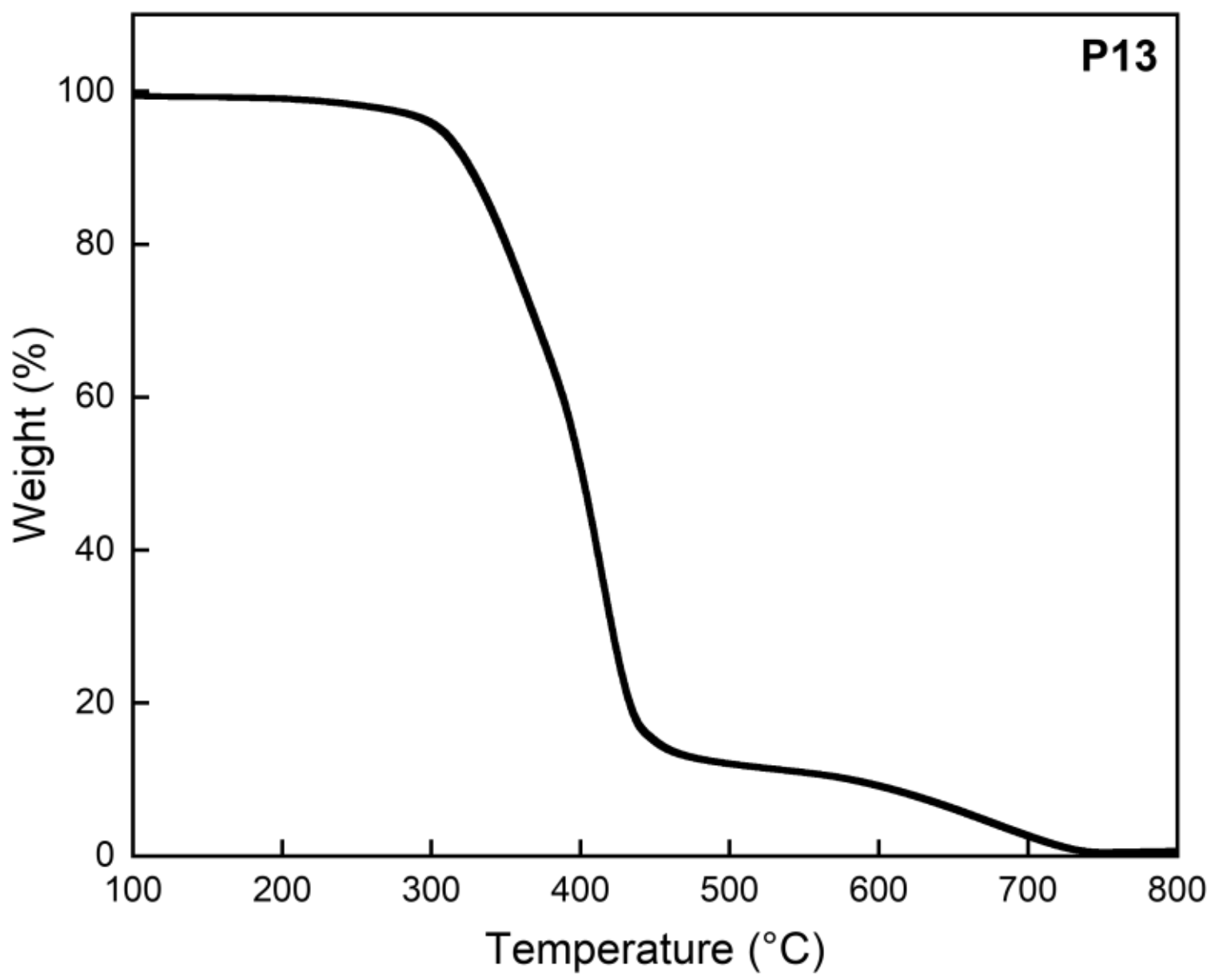

Figure S119. TGA curve of **P13**.

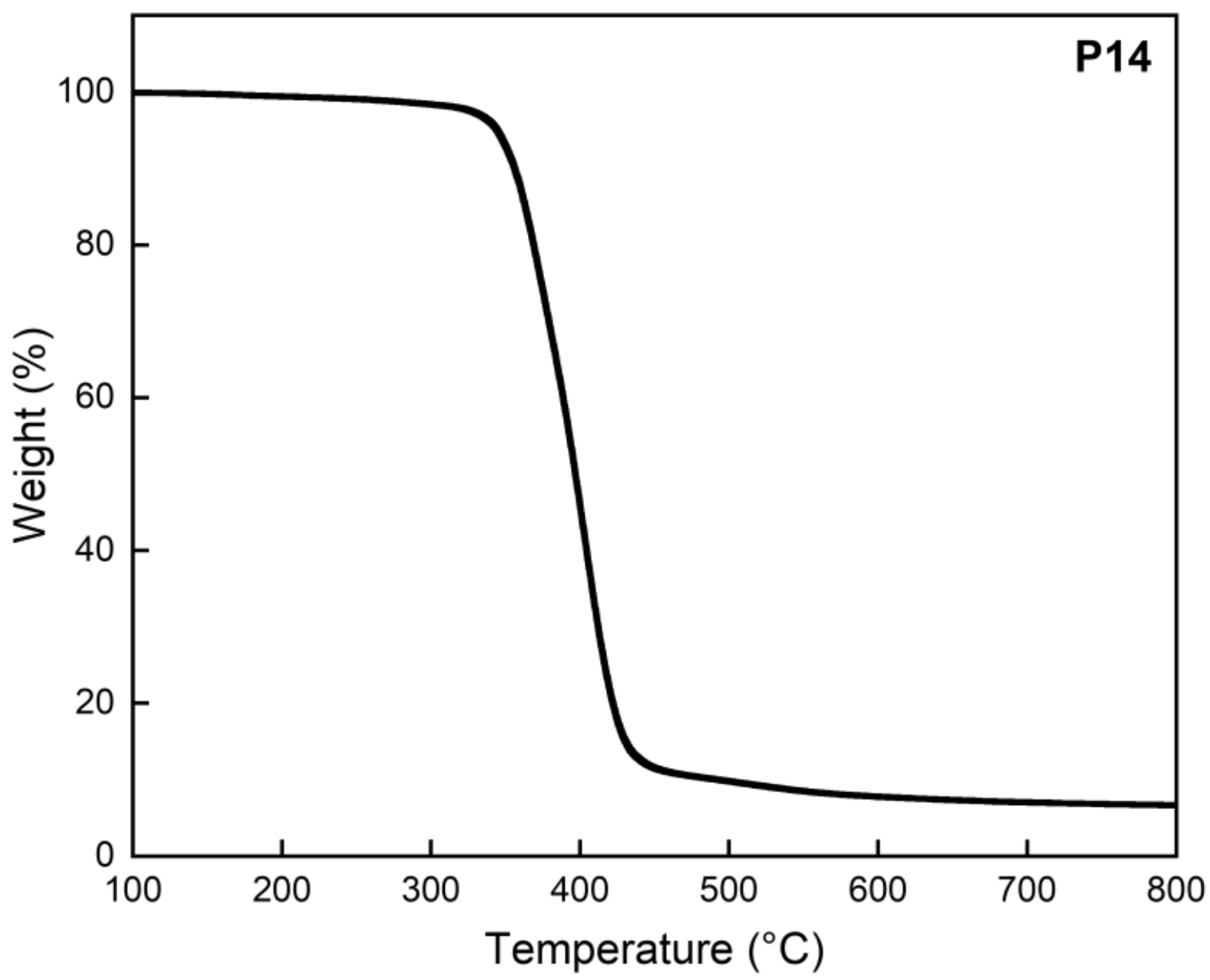

Figure S120. TGA curve of **P14**.

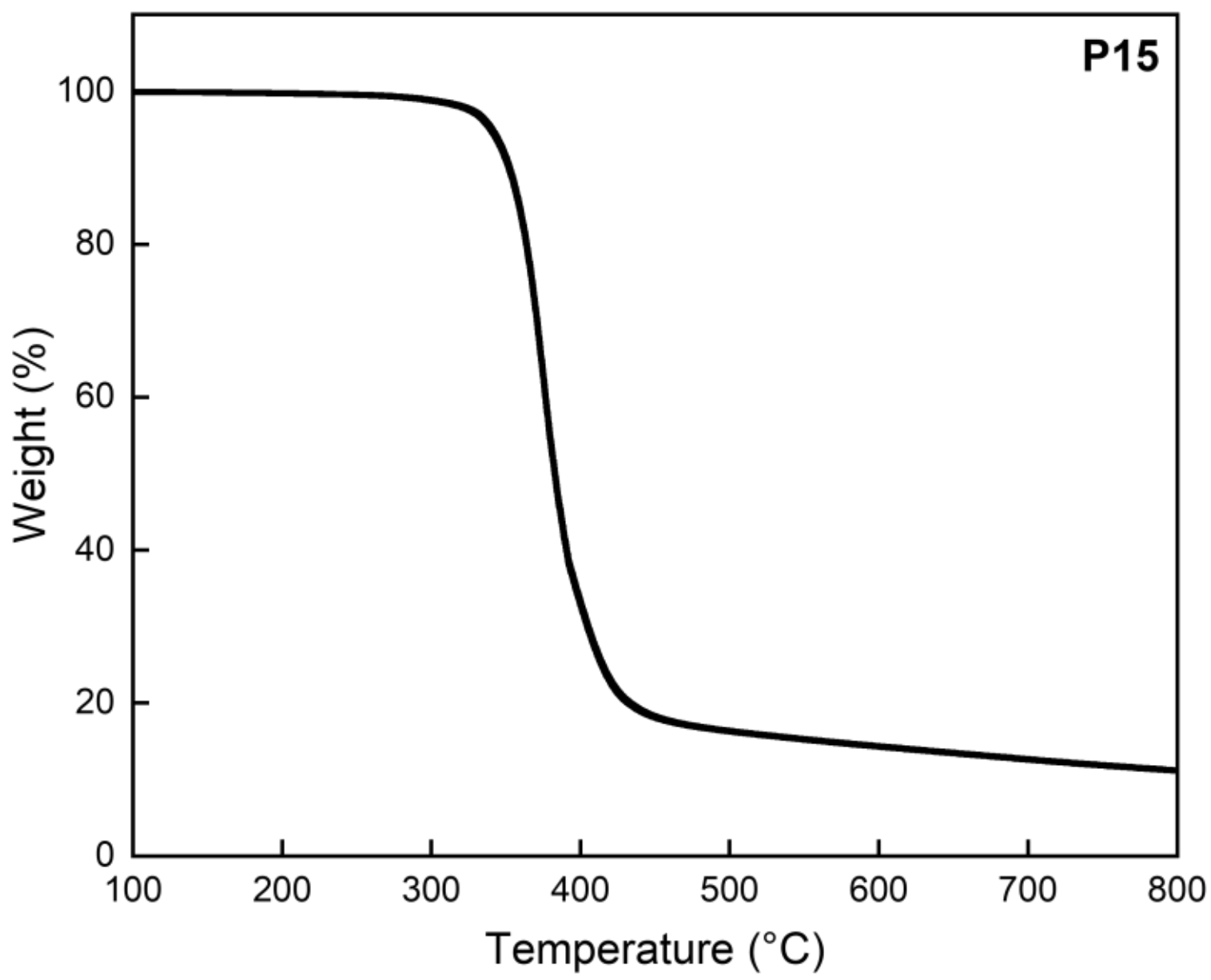

Figure S121. TGA curve of **P15**.

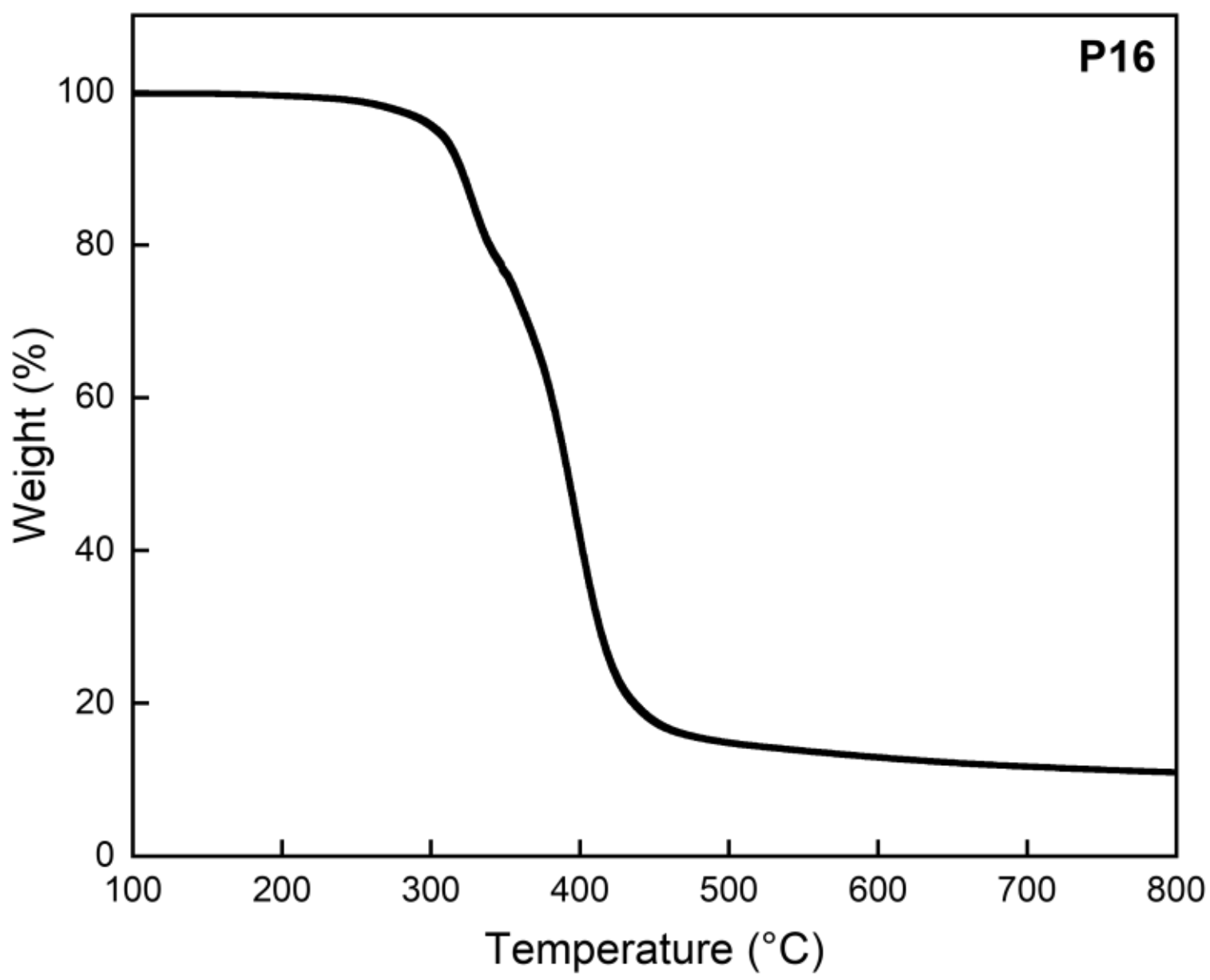

Figure S122. TGA curve of **P16**.

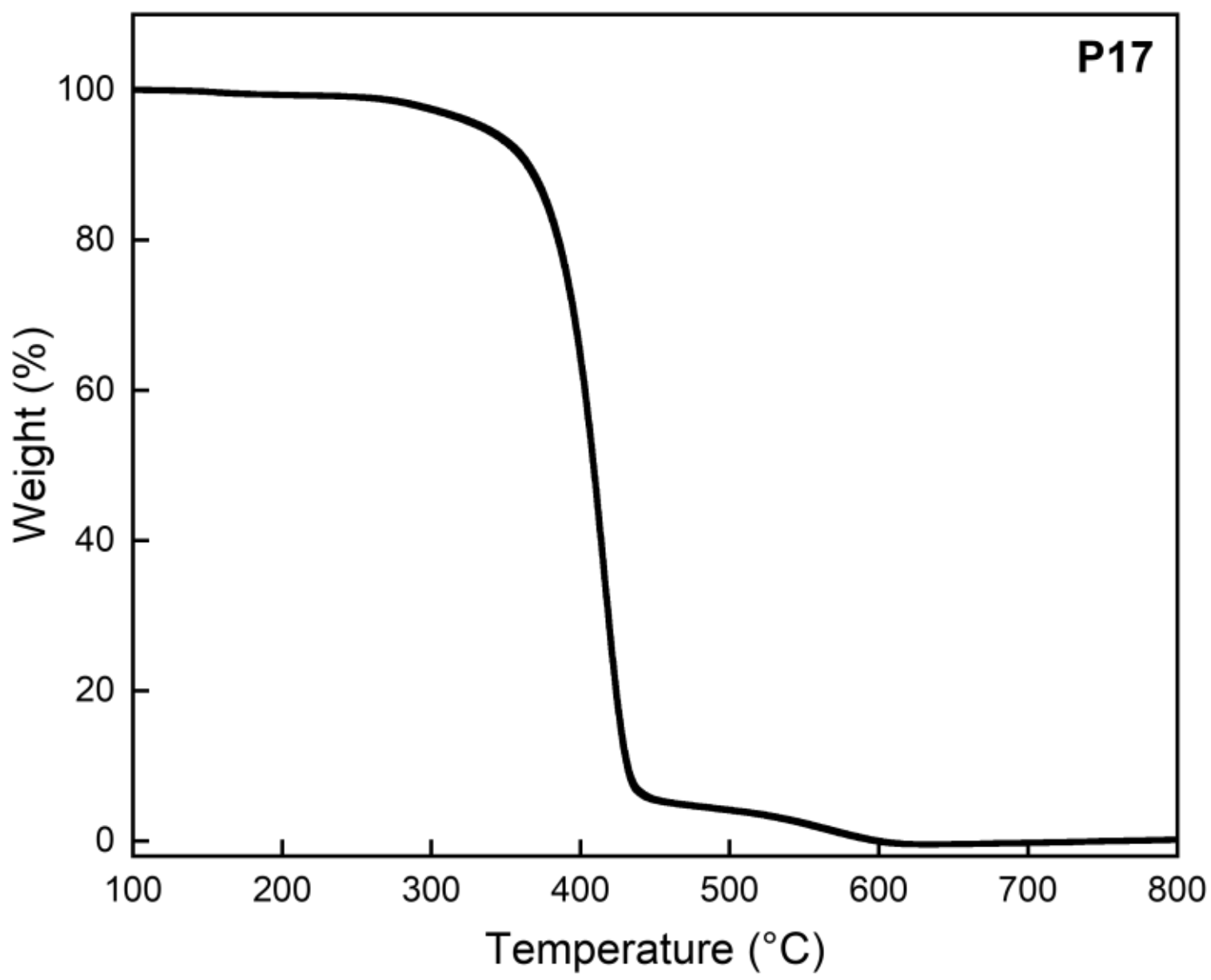

Figure S123. TGA curve of **P17**.

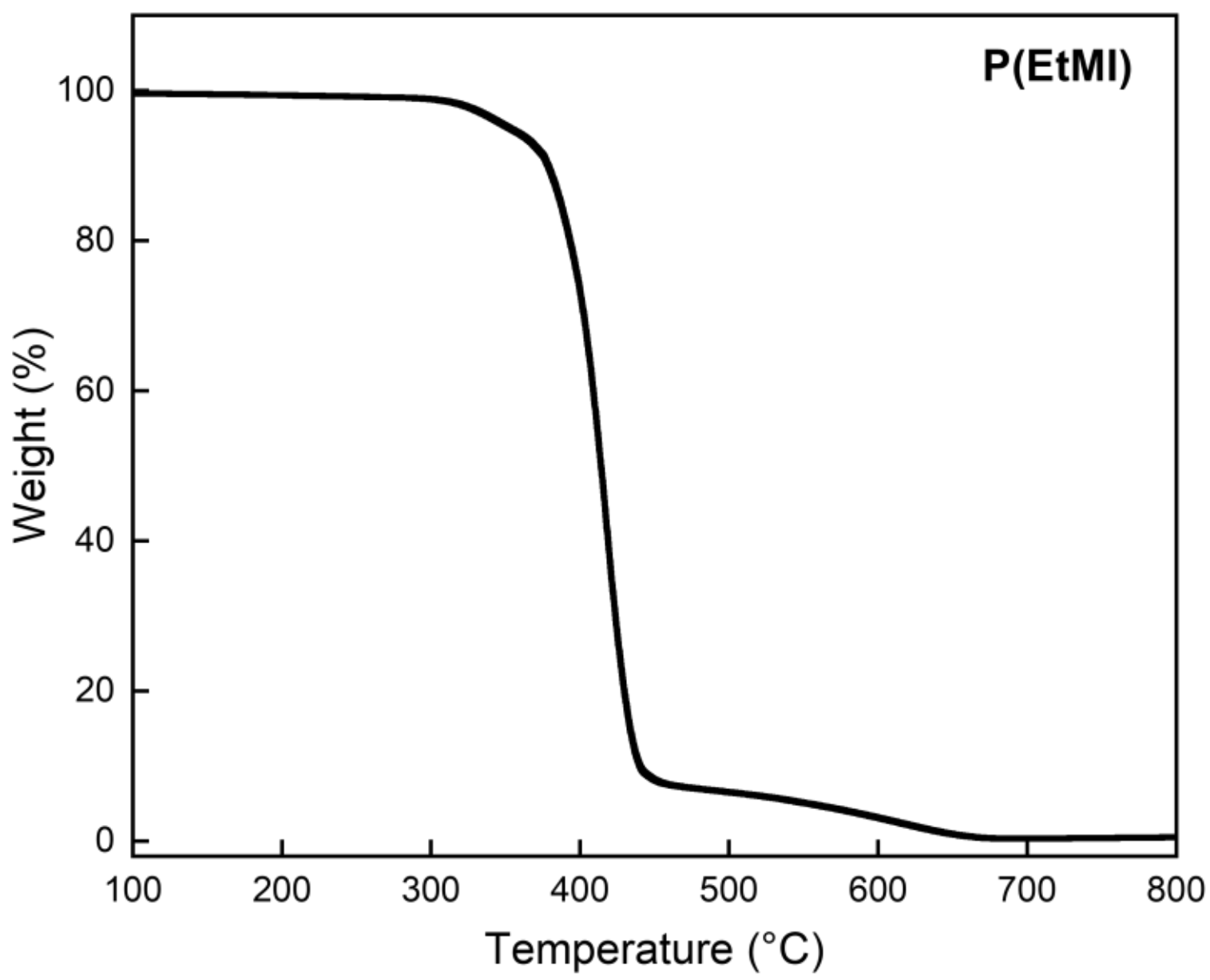

Figure S124. TGA curve of **P(EtMI)**.

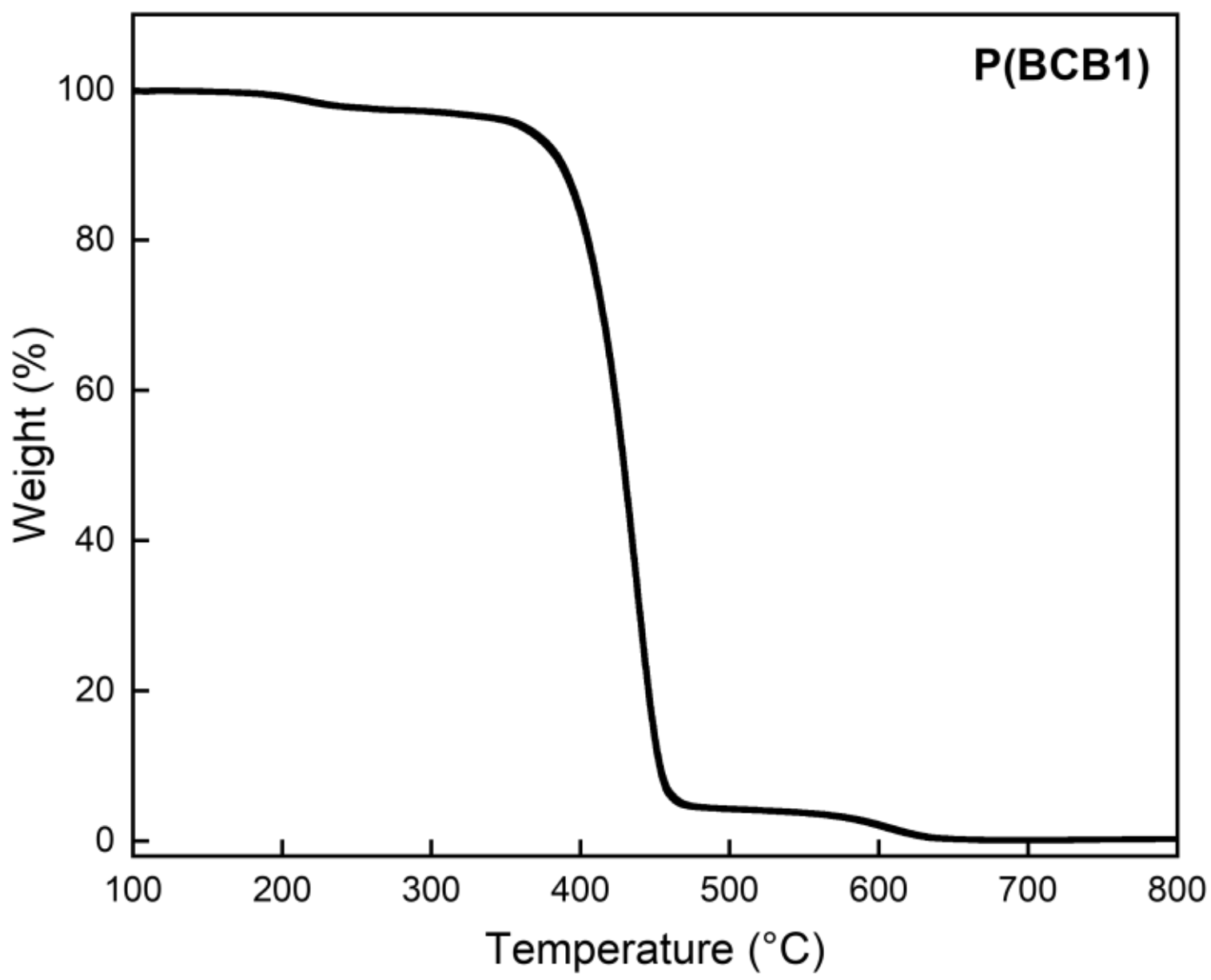

Figure S125. TGA curve of **P(BCB1)**.

21. DSC curves

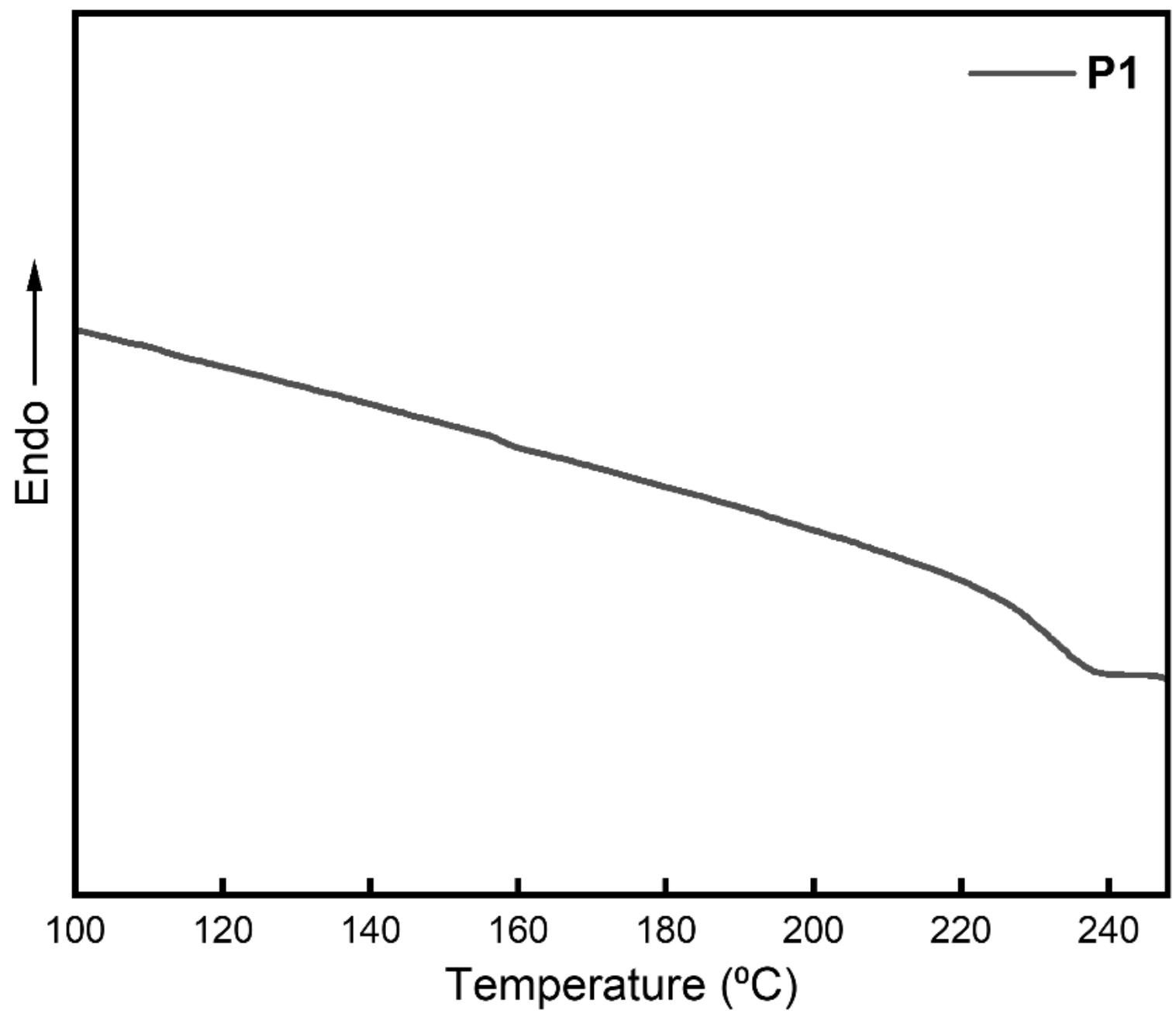

Figure S126. DSC curve of **P1**.

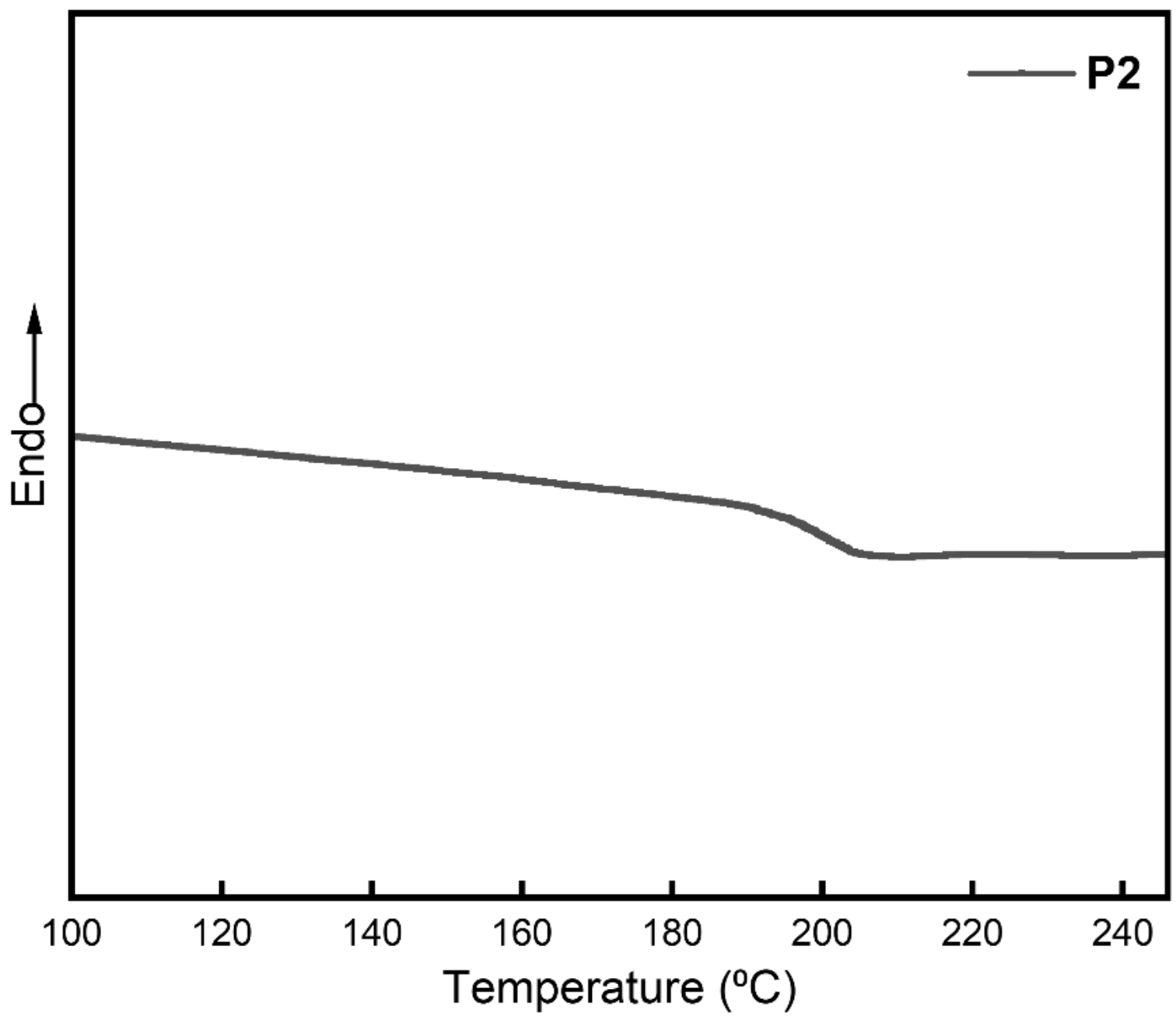

Figure S127. DSC curve of **P2**.

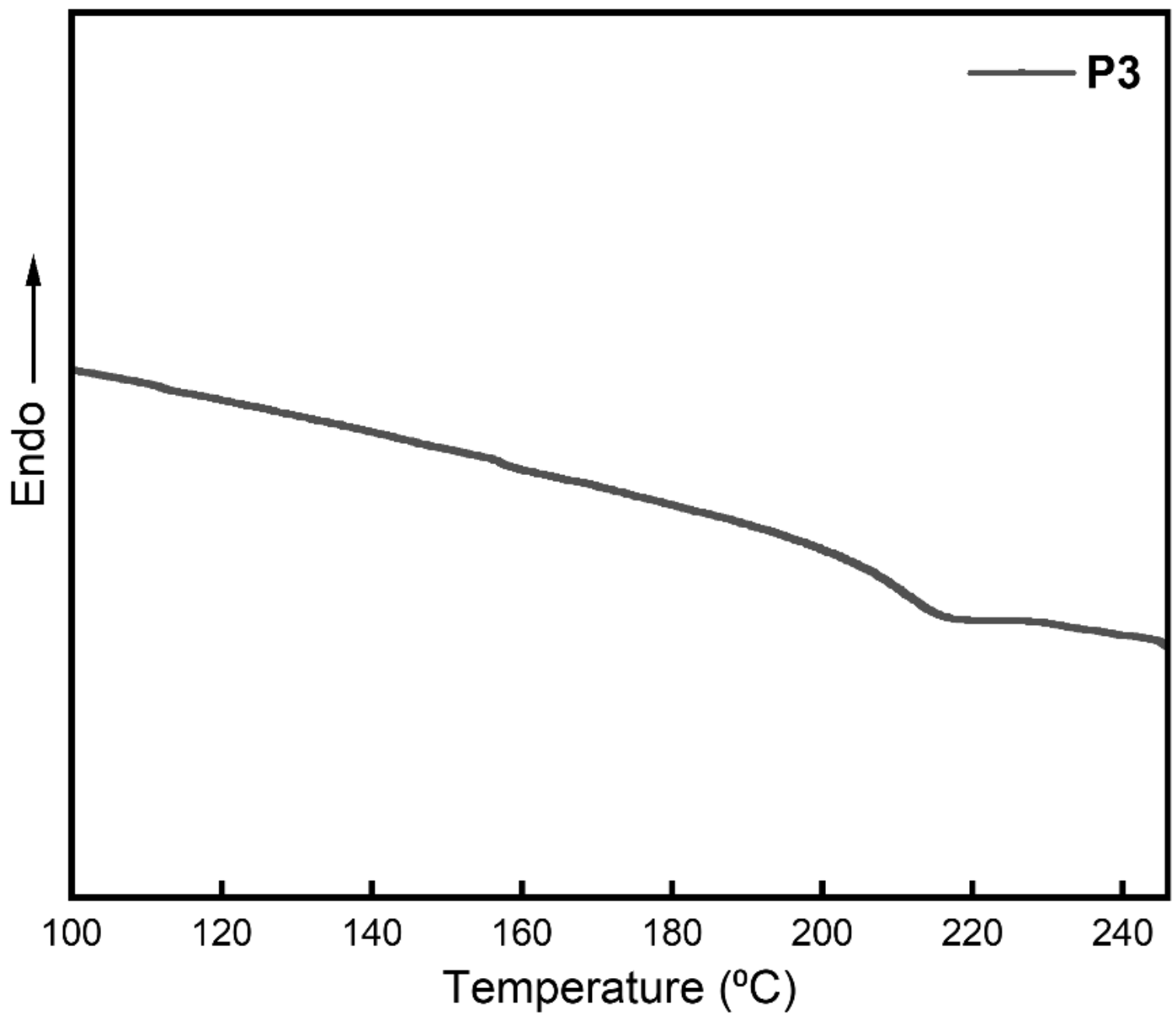

Figure S128. DSC curve of **P3**.

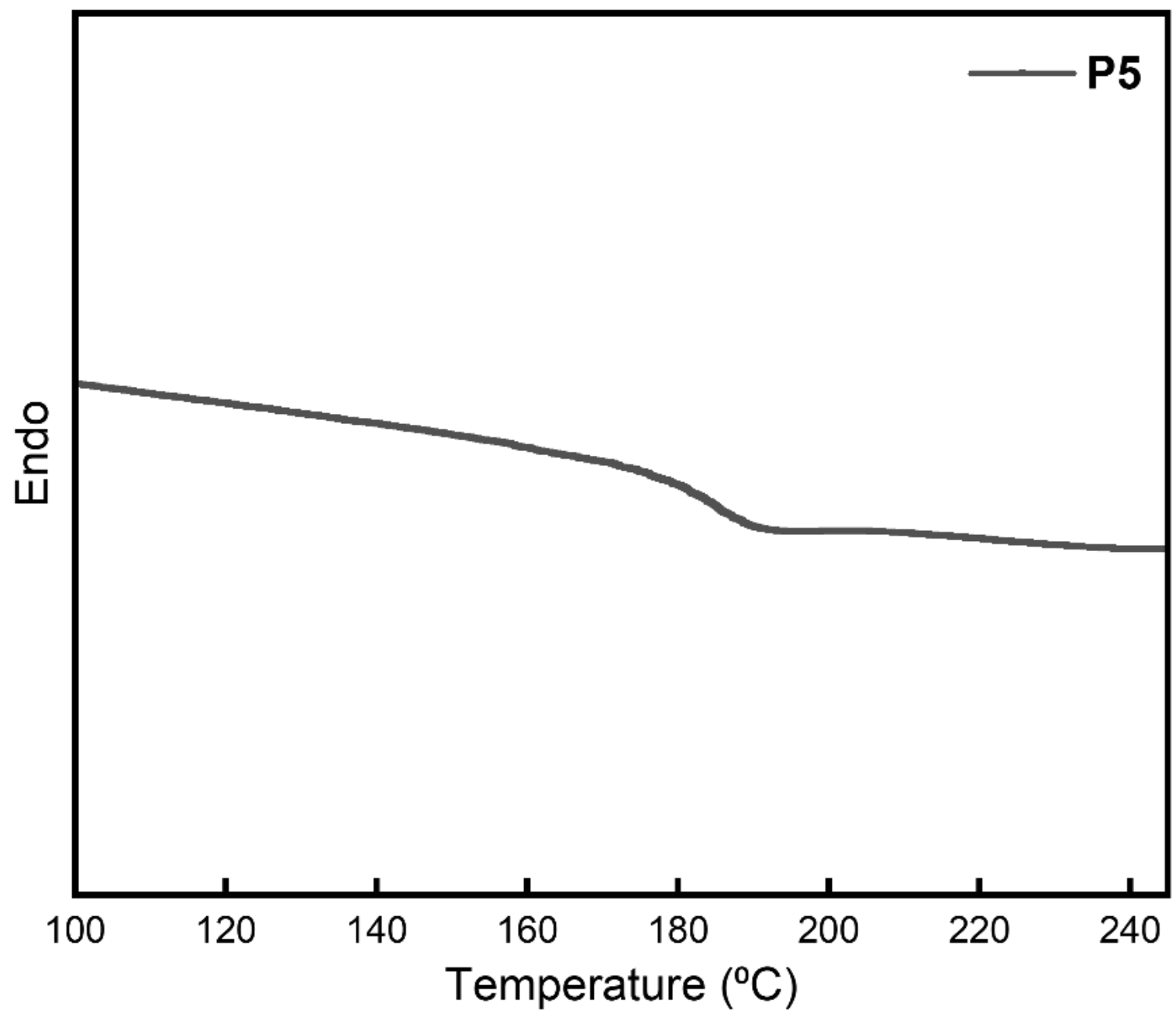

Figure S129. DSC curve of **P5**.
